# Controlling Reductive Elimination Pathways in Ti(IV) Pincer Complexes: Concerted versus Radical Mechanisms via Ligand Design

**DOI:** 10.1021/jacs.5c21215

**Published:** 2026-01-22

**Authors:** Paul Fritsche, Corinna Czernetzki, Maxi Liesa Heldner, Laura Hörlin, Ivo Krummenacher, Gabriele Hierlmeier

**Affiliations:** Julius-Maximilians-Universität Würzburg, 9190 Institute of Inorganic Chemistry, Am Hubland, Würzburg 97074, Germany

## Abstract

Oxidatively induced reductive elimination (OIRE) is a powerful strategy for the formation of carbon–carbon bonds and has been widely employed in late transition metal catalysis. In contrast, early transition metals, especially titanium, have remained largely unexplored in this context. Here, we report two classes of pyridine-based titanium complexes that are structurally similar yet electronically distinct and investigate their oxidation chemistry. Depending on the ligand, the organyl bound to titanium, and the oxidant, selective alkyl radical expulsion is demonstrated, and most notably, a rare, highly selective, and quantitative concerted reductive elimination from a titanium­(IV) complex was established. A combination of quantum chemical calculations, electrochemical measurements, and crossover experiments provided valuable insights into the reaction mechanism. These results demonstrate that appropriate ligand design, particularly the use of tridentate, redox-active ligands, can effectively suppress competing one-electron pathways and allow for selective concerted reactivity. In this way, control over this traditionally elusive elementary step in titanium chemistry was achieved, marking an advance in the development of OIRE processes for early transition metals.

## Graphical abstract

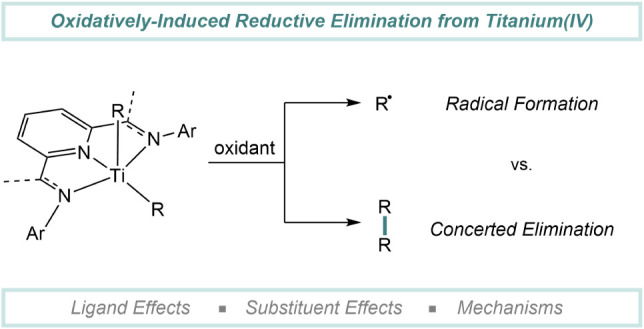

## Introduction

Reductive elimination is a fundamental transformation in organometallic chemistry, often serving as the final, product-defining, and selectivity-determining step in catalytic cycles. Its central role is evident in cross-coupling reactions, which have become essential tools in organic synthesis for the formation of carbon–carbon bonds.^1^ To enable such transformations and expand the reactivity of otherwise inert transition metal complexes, electron transfer processes are widely employed.^2^ Among these, oxidatively induced reductive elimination (OIRE) has been a particularly powerful strategy (Figure 1A). Oxidation of a metal complex, typically by one or two electrons, reduces its electron density and thereby lowers the activation barrier for reductive elimination.^3^ This enables bond-forming steps that would otherwise be energetically disfavored. While such redox-triggered eliminations have been studied in stoichiometric systems for decades,^4^ their application in homogeneous catalysis has only recently gained broader attention, with group 9 metals^5^ and Pd-based systems^6^ being particularly prominent. The vast majority of the existing literature focuses on late transition metals, for which the influence of metal oxidation state, ligand, and substituent effects is well understood. Mechanistic pathways for these metals have been investigated extensively, and a wide range of catalytic applications has been established using chemical or electrochemical oxidation.^7^ Notably, many of these electrocatalysis examples are enabled by prior insights derived from stoichiometric organometallic chemistry.

**Figure 1:**
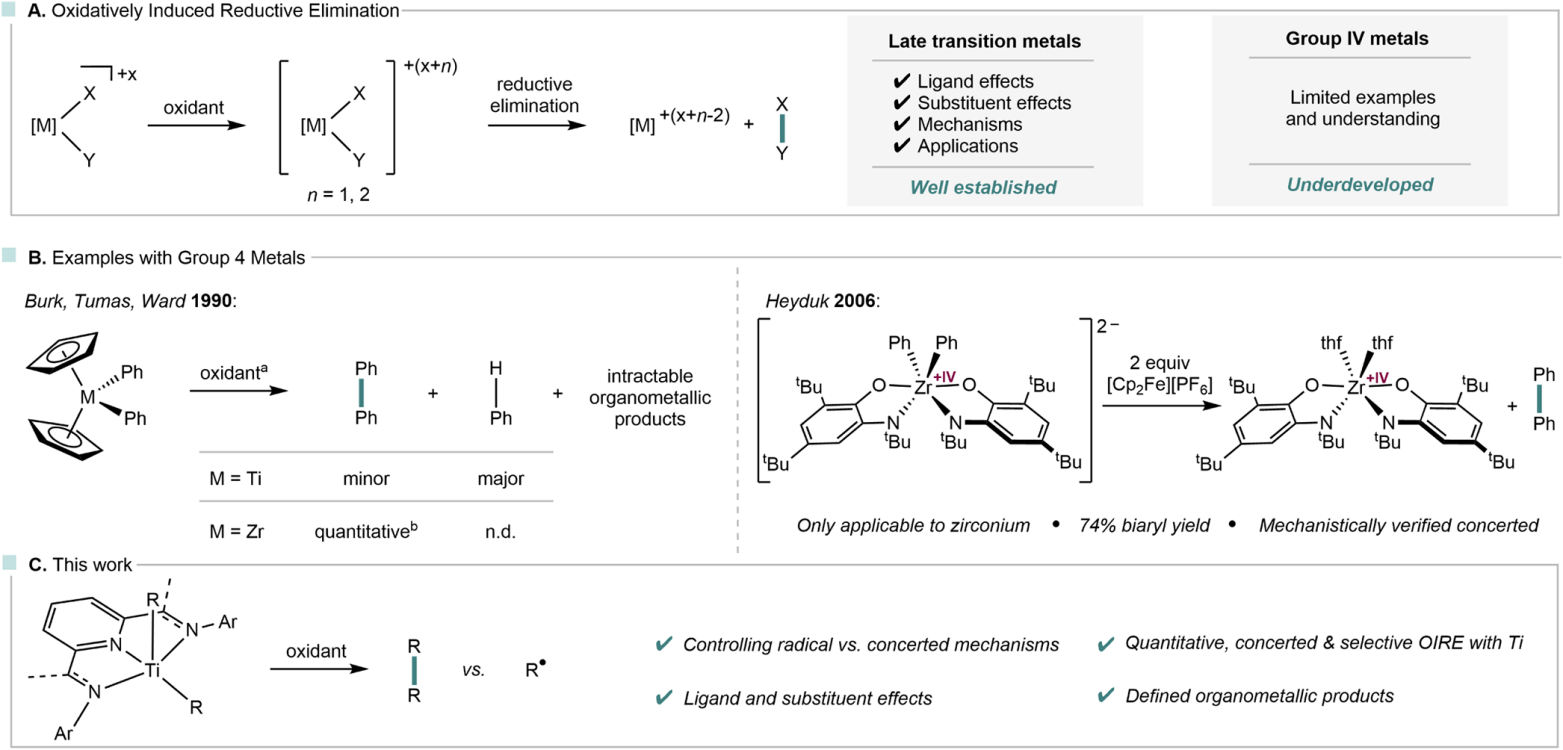
Oxidatively induced reductive elimination (OIRE) for the formation of X–Y bonds in late and early transition metal chemistry (A); examples for OIRE with group 4 metals (B) and titanium pincer-complexes in OIRE (C); ^a^ oxidants used: silver and ferrocenium salts, 7,7,8,8-tetracyanoquinodimethane, ^b^ quantitative yields were only achieved with tetrakis­(trifluoromethyl)­cyclopentadienon as oxidant; n.d. = not detected.

In contrast, the application of OIRE to early transition metals remains largely unexplored, with only a few examples reported to date.^8–10^ This lack of studies can be attributed to their fundamentally different electronic structures and redox properties.^11^ Early transition metal complexes typically exhibit limited stability in their lower oxidation states, and their electron-deficient nature poses a fundamental challenge for the design of OIRE reactions. Nevertheless, the oxidation chemistry of group 4 metallocenes has been studied, most notably by Burk, Tumas, and Ward, who investigated the oxidation of diphenyl metallocene complexes (Figure 1B). In the case of zirconium, oxidation with tetrakis­(trifluoromethyl)­cyclopentadienone led to quantitative formation of biphenyl. In contrast, oxidation of the analogous titanium complex yielded only small amounts of the C–C coupling product with benzene as the major product. The majority of these reactions produced complex mixtures of organometallic byproducts, which were not characterized.^12^ Selective OIRE examples with titanium are limited to the elimination of cyclopropane from titanacyclobutanes, a transformation that notably proceeds via a biradical rather than a concerted mechanism.^13^

A new direction for oxidatively induced reductive elimination with early transition metals has been provided by work from the Heyduk group (Figure 1B).^14^ A dianionic zirconium diphenyl complex supported by amidophenolate ligands was reacted with ferrocenium hexafluorophosphate in a two-electron oxidation, affording the corresponding biaryl in 74% yield. Mechanistic investigations confirmed that biaryl formation proceeds via a concerted elimination pathway. Although computational investigations predicted lower transition states for reductive elimination with analogous titanium complexes, this transformation remained limited to zirconium.^15^ Follow-up studies by the same group on such titanium amidophenolate complexes revealed that these bidentate ligands frequently engage in one-electron redox processes, resulting in the formation of free iminoquinones.^16^ The unselective behavior typically observed in titanium oxidation chemistry highlights a significant gap in the development of well-defined OIRE processes with this element. This limitation can be attributed to the accessibility of the Ti­(III) oxidation state, which promotes one-electron, radical-type reactivity and renders concerted reductive elimination pathways from titanium elusive.^17^

Motivated by this challenge, we aimed to address this gap through the introduction of tridentate ligands for OIRE chemistry with titanium (Figure 1C). Herein, we report the oxidation chemistry of titanium complexes containing two structurally related but electronically distinct pyridine-based pincer-type ligands, i.e., pyridinediamido and pyridinediimine. For both ligand systems, alkyl and aryl titanium complexes were synthesized and fully characterized. Their oxidation chemistry was systematically investigated and further elucidated through quantum chemical calculations, which provided insight into the electronic structures. A systematic investigation of ligand, substituent, and oxidant effects allows to achieve control over radical and concerted pathways in the reductive elimination from titanium­(IV). Specifically, the incorporation of redox-active ligands enabled a rare example of a concerted reductive elimination from titanium­(IV), resulting in the quantitative formation of the corresponding biaryl and a single, well-defined organometallic product.

## Results and Discussion

### Synthesis of Pincer-Supported Titanium Diorganyls

To investigate the potential for oxidatively induced reductive elimination in pincer-supported titanium complexes, the oxidation chemistry of complexes with two distinct ligand systems was investigated (Figure 2A). Pyridinediamido-supported complexes (^iPr^PDA)­TiR_2_ (^iPr^PDA = 2,6-(2,6-^i^Pr_2_C_6_H_3_N–CH_2_)_2_C_5_H_3_N^2–^) (**1-R**_**2**_) with R = CH_2_Ph and Ph were studied because the pyridinediamido ligand (**1)** ^**2–**^ is rarely observed in oxidation states other than −2 and is therefore not expected to undergo oxidation readily.^18^ Given the high-lying frontier orbitals of pyridinediimine (PDI) ligands, the closely related redox-active ^Et^PDI (2,6-(2,6-Et_2_C_6_H_3_N = CMe)_2_C_5_H_3_N) ligand was also investigated to explore differences in the oxidation chemistry of its titanium complexes. For both systems, alkyl- and aryl-substituted titanium complexes were chosen because C­(sp^3^)–C­(sp^3^) reductive eliminations are generally considered more challenging compared to C–C reductive eliminations of substituents with greater s-orbital content.^19^ In contrast, C­(sp^2^)–C­(sp^2^) reductive eliminations benefit from higher directionality of orbitals, reduced steric hindrance, and additional stabilizing interactions of the product’s π-system with the metal center, which lower the transition state energy. To explore these differences in well-defined systems, the pyridinediamido dibenzyl complexes **1-(CH_2_Ph)_2_** and **1-(CD**_**2**_ **Ph** **-*d*_5_)**_**2**_ were synthesized according to a previously published procedure,^20^ while **1-Ph**_**2**_ and **1-(Ph** **-*d*_5_)**_**2**_ were obtained in 25% and 56% yield, respectively, from the reaction of **1-Cl**_**2**_ with two equivalents of phenyl­(-*d*_5_) lithium (Figure 2B). Selective benzylation of (^Et^PDI)­TiCl_2_ (**2-Cl**_**2**_)^21^ was achieved with dibenzyl magnesium affording **2-(CH**_**2**_ **Ph)**_**2**_ and **2-(CD** **_2_Ph-*d*_5_)** in 82% and 81% yields, respectively, as dark green powders. Furthermore, **2-Cl**_**2**_ was treated with two equivalents of ArLi (Ar = Ph, Ph-*d*_5_, *p*-Tol), affording the corresponding diaryl complexes (^Et^PDI)­TiAr_2_ (**2-Ar**_**2**_, Figure 2B) in yields of 85% (Ar = Ph, *p*-Tol) and 40% (Ar = Ph-*d*_5_). For complexes **1-Ph**_**2**_, **2-(CH**_**2**_ **Ph)**_**2**_ and **2-(*p*-Tol)_2_**, single crystals suitable for X-ray diffraction were obtained, confirming their molecular structures in the solid state (Figure 2C). All complexes are best described as distorted square pyramids, as reflected by their τ_5_ values of 0.04 (**2-(CH**_**2**_ **Ph)**_**2**_), 0.08 (**1-Ph**_**2**_), and 0.24 (**2-(** ***p*** **-Tol)**_**2**_).^22^ **2-(CH**_**2**_ **Ph)**_**2**_ features a Ti1–C31 *ipso* interaction of 2.558(3) Å, which was not present in the corresponding PDA-complex **1-(CH**_**2**_ **Ph)**_**2**_.^20^ Similar to the PDA complex, however, the ^1^H NMR spectrum of **2-(CH**_**2**_ **Ph)**_**2**_ at ambient temperature features broad resonances for the benzyl ligands, which are resolved upon cooling to 193 K.^23^ The structures of aryl complexes **1-Ph**_**2**_ and **2-(*p*-Tol)_2_** both reveal an agostic interaction of the *ortho*-H atom of the aryl substituent with the titanium atom (**1-Ph**_**2**_: Ti1–H39: 2.520(1) Å, Ti1–H39–C39: 90.35(1)°; **2** **-(*p*-Tol)_2_**: Ti1–H31A: 2.259 Å, Ti1–H31A–C31A: 95.2(2)°).^24^ Comparable Ti–H bond distances were observed in Cp^R^TiPh_3_ (R = (CH_2_)_2_OCH_3_; Ti–H = 2.65 Å, Ti–H–C: 87.9(2)°).^25,26^ Despite the presence of this agostic interaction in the solid state, solutions of **1-Ph**_**2**_ and **2-(*p*-Tol)**_**2**_ in benzene-*d*_6_ exhibit a single set of signals for the aryl substituents in the ^1^H NMR spectrum even at 193 K, indicating rapid interchange.^27^

**Figure 2:**
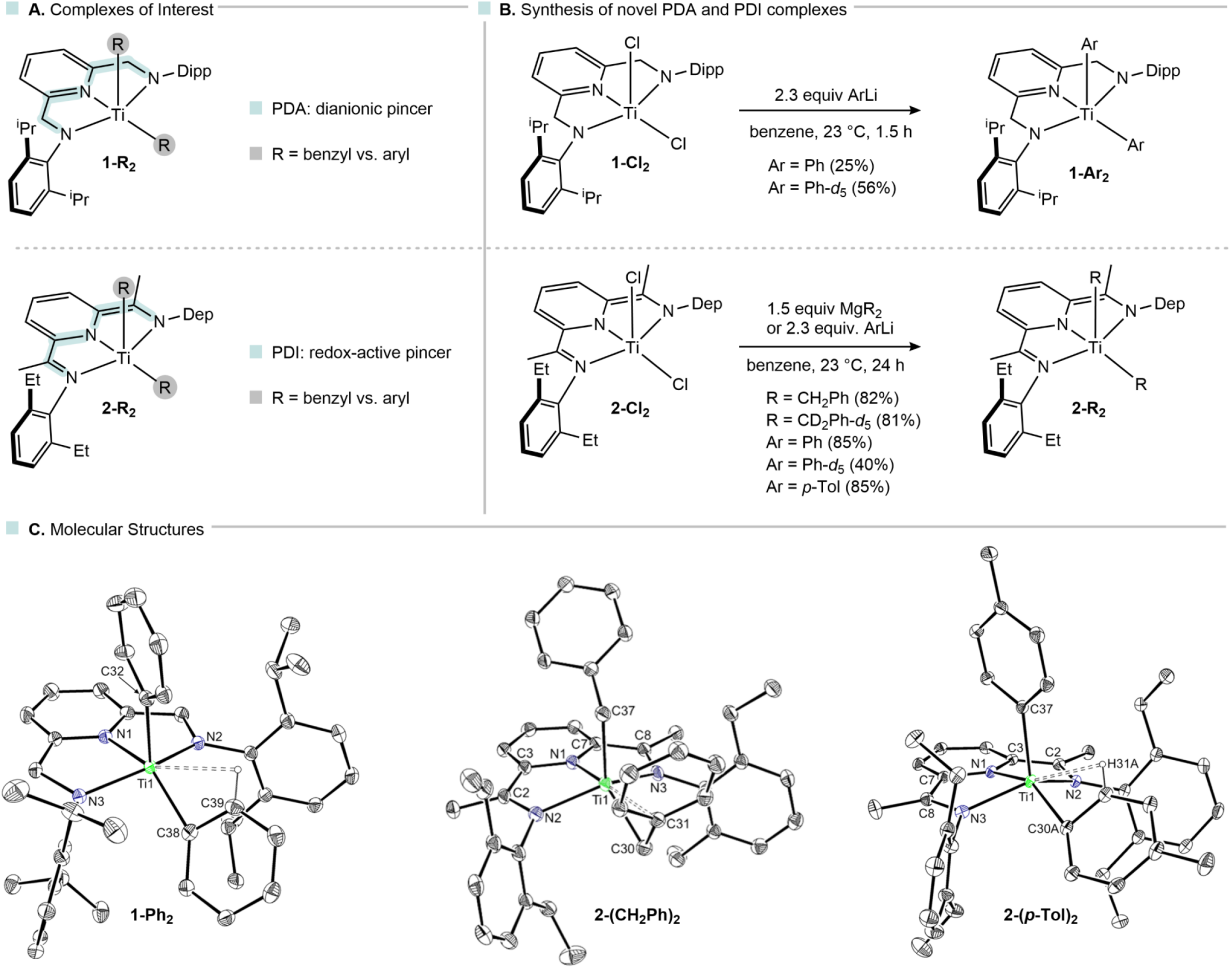
Pincer-supported titanium complexes: complexes of interest (A), synthesis of novel titanium dialkyl and diaryl complexes (B), molecular structures of **1-Ph**_**2,**_ **2-(CH**_**2**_ **Ph)**_**2**_ and **2-(*p*-Tol)_2_** in the solid state (C); molecular structures are shown with 30% probability ellipsoids, and hydrogen atoms (except for those with Ti interactions) are omitted for clarity.

To assess the ligand oxidation state in the novel PDI complexes, the Δ-value, introduced by Wieghardt and coworkers,^28^ was calculated. This value gives an estimate of the ligand oxidation state by taking bond elongation and bond contraction upon occupying antibonding π*-orbitals of the ligand into account. The Δ-values for **2-R**_**2**_ were determined to be 0.046 Å (R = CH_2_Ph) and 0.047 Å (R = *p*-Tol), respectively, which is comparable to that in [(PDI)^2–^Zn­(4-NMe_2_-pyr)_2_] (0.065 Å) and markedly lower than the values reported for zinc complexes bearing monoanionic PDI ligands (Δ = 0.120 ± 0.02 Å), supporting the assignment of a doubly reduced (PDI)^2–^ ligand.^29^ To further probe the electronic structure, DFT calculations were performed at the TPSSh-D4/def2-TZVP level of theory. Both singlet and triplet states were evaluated to distinguish between a closed-shell (PDI)^2–^ dianion and an open-shell dianion^–^ for **2-R**_**2**_ (R = CH_2_Ph, *p*-Tol). The singlet state was found to be 4.1 kcal·mol^–1^ (Gibbs free energy, R = CH_2_Ph) and 2.5 kcal·mol^–1^ (R = *p*-Tol) more stable than the triplet, and better reproduced the bond lengths (see Table S6 in SI). Broken symmetry calculations (BS­(1,1)) also converged to closed-shell singlet solutions in both cases.

### Electrochemical Studies

Electrochemical studies of complexes **1-R**_**2**_ (R = CH_2_Ph, Ph) and **2-R**_**2**_ (R = CH_2_Ph, *p*-Tol) were performed in order to provide further insights into their redox behavior (Figure 3). The cyclic voltammogram of **1-(CH**_**2**_ **Ph)**_**2**_ in THF using [^n^Bu_4_N]­[BAr^F^_4_] (BAr^F^_4_ = tetrakis­(3,5-bis­(trifluoromethyl)­phenyl)­borate) as electrolyte revealed one oxidation at *E* ^p^ = 1.1 V (vs Cp_2_Fe^+/0^, Figure 3A) when a scan rate (υ) of 50 mV·s^–1^ was employed. At higher scan rates, no evidence of a quasi-reversible process was observed; however, a second, poorly defined oxidation event emerged. Similarly, electrochemical analysis of **1-Ph**_**2**_ by cyclic voltammetry revealed one irreversible oxidation at *E* ^p^ = 1.0 V (υ = 50 mV·s^–1^). In this case, the peak potential was located for different scan rates, and plotting the log­(υ) *versus E* ^p^ afforded a linear relationship with a slope of 55 mV, indicating an irreversible electron transfer event (see Figure S82 in SI).^30^ The cyclic voltammogram of **2-(CH**_**2**_ **Ph)**_**2**_ recorded in THF shows a quasi-reversible oxidation event at −0.4 V (Figure 3B, scan rate υ = 50 mV·s^–1^) and an ill-defined second oxidation at 1.1 V (vs Cp_2_Fe^+/0^, see Supporting Information), indicating a primary electrochemical reaction followed by a chemical reaction (EC mechanism).

**Figure 3:**
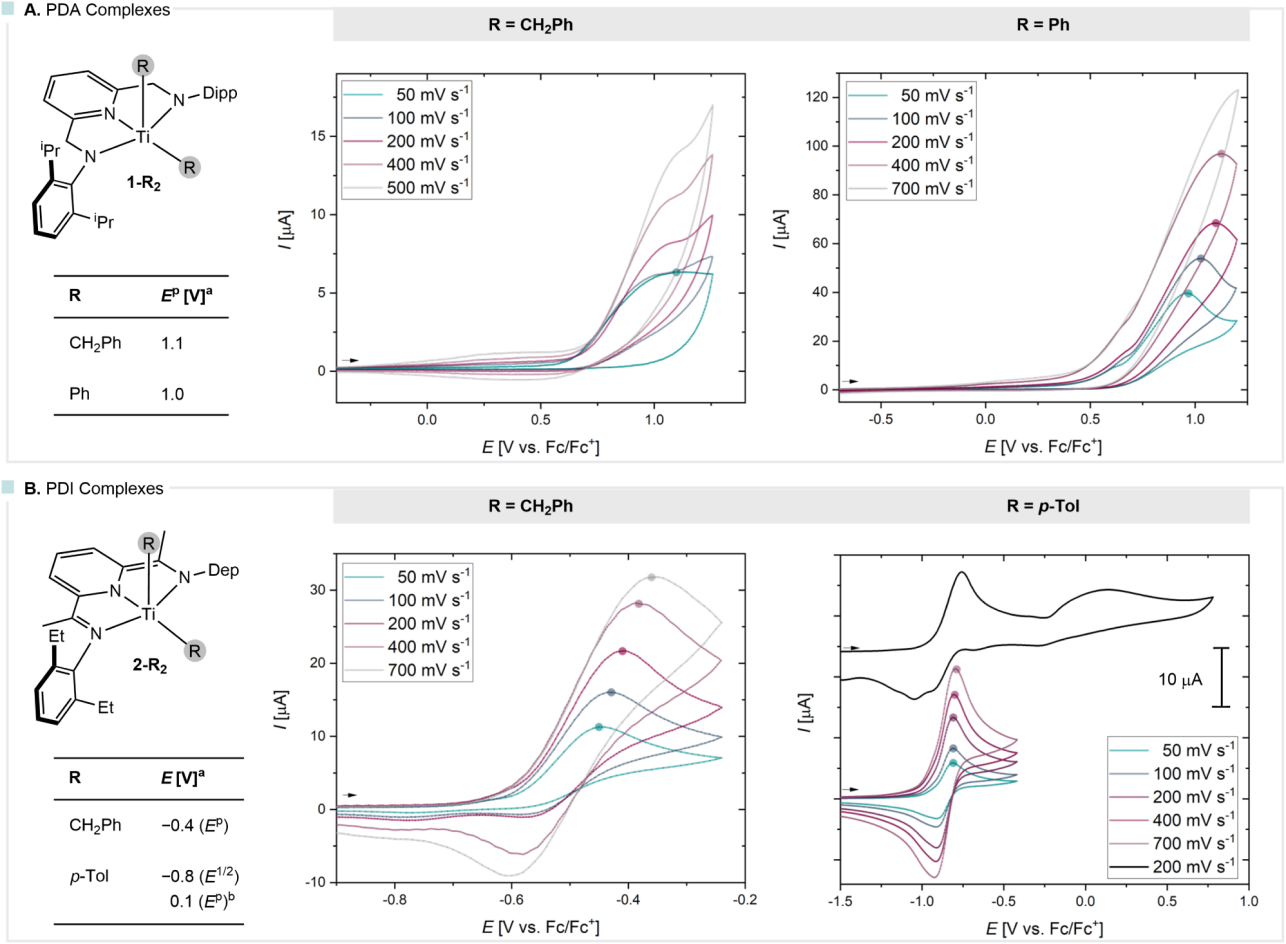
Cyclic voltammetry of PDA (A) and PDI (B) titanium complexes with benzyl and aryl substituents recorded in THF using [^n^Bu_4_N]­[BAr^F^_4_] as electrolyte; ^a^ peak potential is given at a scan rate of 50 mV·s^–1^; ^b^ peak potential at a scan rate of 200 mV·s^–1^.

Lastly, the cyclic voltammogram of **2-(*p*-Tol)**_**2**_ (Figure 3B) revealed an oxidation event at *E* ^1/2^ = −0.8 V that appeared reversible, as indicated by scan rate studies. At higher potentials, a second irreversible oxidation event (*E* ^p^ = 0.1 V) emerges. Differential pulse voltammetry (DPV) was used to determine whether the observed redox events correspond to one- or two-electron processes. Comparison with DPV traces of equimolar decamethylferrocene indicated that the events represent two sequential one-electron oxidations (Figure S84 in the SI). In summary, the oxidation chemistry of PDA complexes is marked by irreversible oxidations at 1.1 V, indicating the need for strong oxidants in stoichiometric transformations. In contrast, related PDI complexes exhibit much lower oxidation potentials (−0.4 V for R = CH_2_Ph, −0.8 V for R = *p*-Tol) with (quasi)-reversible behavior, although the **2-(*p*-Tol)**_**2**_ complex undergoes an additional irreversible oxidation.

### Electronic Structure Analysis

To gain further insight into the electronic structures, DFT calculations were performed at the TPSSh-D4 def2-TZVP level of theory. For **1-(CH**_**2**_ **Ph)**_**2**_, the HOMO is predominantly localized on the Ti–C bonds of the benzyl groups, with minor contributions from the C­(sp^2^) atoms of the benzyl substituents (Figure 4). In contrast, the HOMO of the related phenyl complex **1-Ph**_**2**_ is primarily localized on the amido nitrogen atoms of the ligand. Analysis of the analogous PDI complexes **2-R**_**2**_ (R = CH_2_Ph, *p*-Tol) revealed pronounced differences. The HOMO of **2-(CH**_**2**_ **Ph)**_**2**_ is largely ligand-centered, with negligible electron density on the Ti–C bonds, in stark contrast to the pronounced Ti–C character observed in **1-(CH**_**2**_ **Ph)**_**2**_. Consistent with this trend, **2-(*p*-Tol)**_**2**_ also features a ligand-centered HOMO, resembling that of **2-(CH**_**2**_ **Ph)**_**2**_. These observations highlight the profound influence of the redox-active ligand on the electronic structure, which directly correlates with the divergent reactivity of the two systems (*vide infra*).

**Figure 4:**
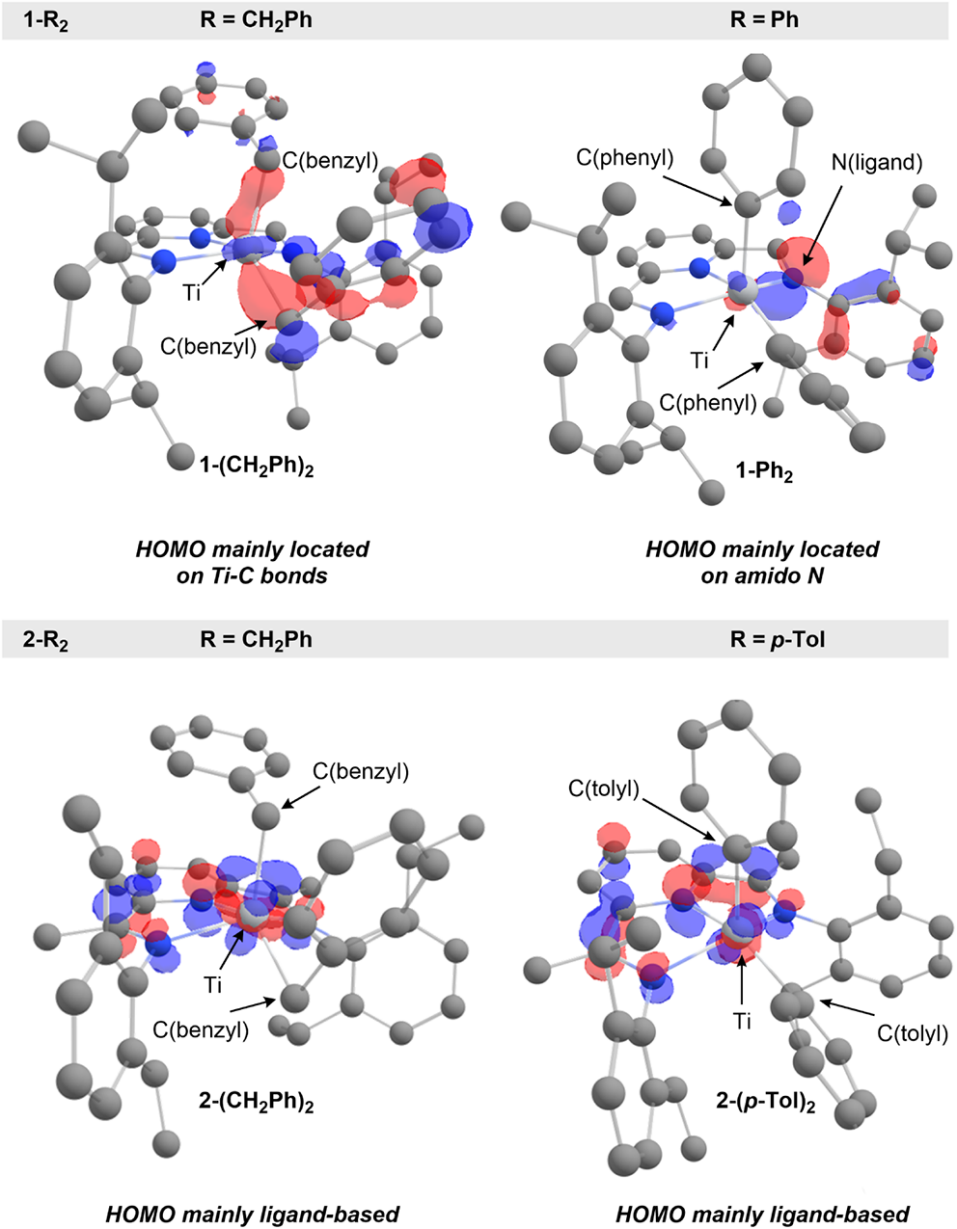
Plots of the HOMO of complexes **1-R**_**2**_ (R = CH_2_Ph, Ph) and **2-R**_**2**_ (R = CH_2_Ph, *p*-Tol) with isosurface contour values of 0.06; hydrogen atoms and methyl groups on the *p*-Tol substituent in **2-(*p*-Tol)**_**2**_ are omitted for clarity.

### Chemical Oxidation of PDA Complexes

Based on the computational and electrochemical findings, chemical oxidation of complexes **1-R**_**2**_ (R = CH_2_Ph, Ph) was studied. Given the rather high oxidation potentials, silver salts were selected as appropriate oxidants.^31^ Treatment of **1-(CH**_**2**_ **Ph)**_**2**_ with one equivalent of silver­(I) trifluoromethanesulfonate in benzene-*d*_6_ resulted in the formation of 1,2-diphenylethane (bibenzyl) in 74% yield (100% yield based on elimination of one benzyl substituent from **1-(CH**_**2**_ **Ph)**_**2**_, Figure 5A). The ^19^F NMR spectrum of the reaction features a singlet at −77.61 ppm, accounting for over 90% of all signals in intensity, which is consistent with the incorporation of a triflate group in the titanium complex. Single crystals suitable for X-ray diffraction were obtained from a concentrated solution of benzene at ambient temperature, confirming the formation of (^iPr^PDA)­Ti­(CH_2_Ph)­(OSO_2_CF_3_) (**1-(CH**_**2**_ **Ph)­(OTf)**, Figure 5B). Complex **1-(CH**_**2**_ **Ph)­(OTf)** was isolated in 31% yield following recrystallization from THF. To probe the mechanism of this reaction and distinguish between a radical expulsion or a concerted bimetallic pathway, the oxidation of **1-(CH**_**2**_ **Ph)**_**2**_ with silver triflate was performed in the presence of different radical traps. The reaction with TEMPO [(2,2,6,6-tetramethylpiperidin-1-yl)­oxyl] revealed the formation of TEMPO–CH_2_Ph in quantitative yields (>95%), indicating interception of a benzyl radical.^32^ Oxidation of **1-(CH**_**2**_ **Ph)**_**2**_ in the presence of further radical traps including phenothiazine (PTZ) and Gomberg’s dimer also afforded the corresponding trapping products.^33^ Similarly, in the presence of the spin traps DMPO (5,5-dimethyl-1-pyrroline *N*-oxide) and PBN (*N*-*tert*-butyl-α-phenylnitrone) the corresponding benzyl-substituted radicals were detected by EPR spectroscopy. The difference in the locations of the HOMOs prompted further studies on the oxidation chemistry of **1-Ph**_**2**_ with AgOTf, in analogy to **1-(CH**_**2**_ **Ph)**_**2**_. Unlike its benzyl-substituted analogue, oxidation of **1-Ph**_**2**_ with three equivalents of AgOTf yielded only 18% of biphenyl and benzene as the major organic product as detected by ^1^H NMR spectroscopy and mass spectrometry (Figure 5C). A mixture of organometallic products was formed, among which (^iPr^PDA)­TiPh­(OTf) (**1-Ph­(OTf)**) was identified by crystallization from the reaction mixture, indicating radical processes upon phenyl ligand expulsion. Finally, crossover experiments using equimolar mixtures of **1-(CH**_**2**_ **Ph)**_**2**_/**1-(CD**_**2**_ **Ph** **-*d*_5_)_2_** and **1-Ph**_**2**_/**1-** **(Ph-*d*_5_)_2_**, respectively, afforded a mixture of the corresponding isotopologues containing the radical crossover products as established by mass spectrometry (Figure 5D). Previous studies have demonstrated that transmetalation does not occur between these complexes.^20^ Overall, the oxidation of both the benzyl- and phenyl-substituted pyridinediamido titanium complexes produces the C–C coupled products via radical processes. In case of **1-(CH**_**2**_ **Ph)**_**2**_, oxidation is selective and affords good yields of bibenzyl or corresponding trapping products, which can be rationalized by the high contributions of the Ti–C bonds to the HOMO. Notably, related reactivity has been reported by Jordan and coworkers for dibenzyl titanocene, where oxidation with silver tetraphenylborate led to bibenzyl formation through radical expulsion, albeit with lower selectivity and formation of three distinct organometallic products.^9^ In contrast, a single, well-defined organometallic product was isolated after oxidation of pyridinediamido complex **1-(CH**_**2**_ **Ph)**_**2**_, highlighting a more selective radical expulsion pathway via oxidation of the Ti–C bonds. Controlled radical generation plays an important role in synthetic chemistry, enabling, e.g., hydrogen atom transfer reactivity, catalysis, and the generation of novel structural motifs.^34–36^

**Figure 5:**
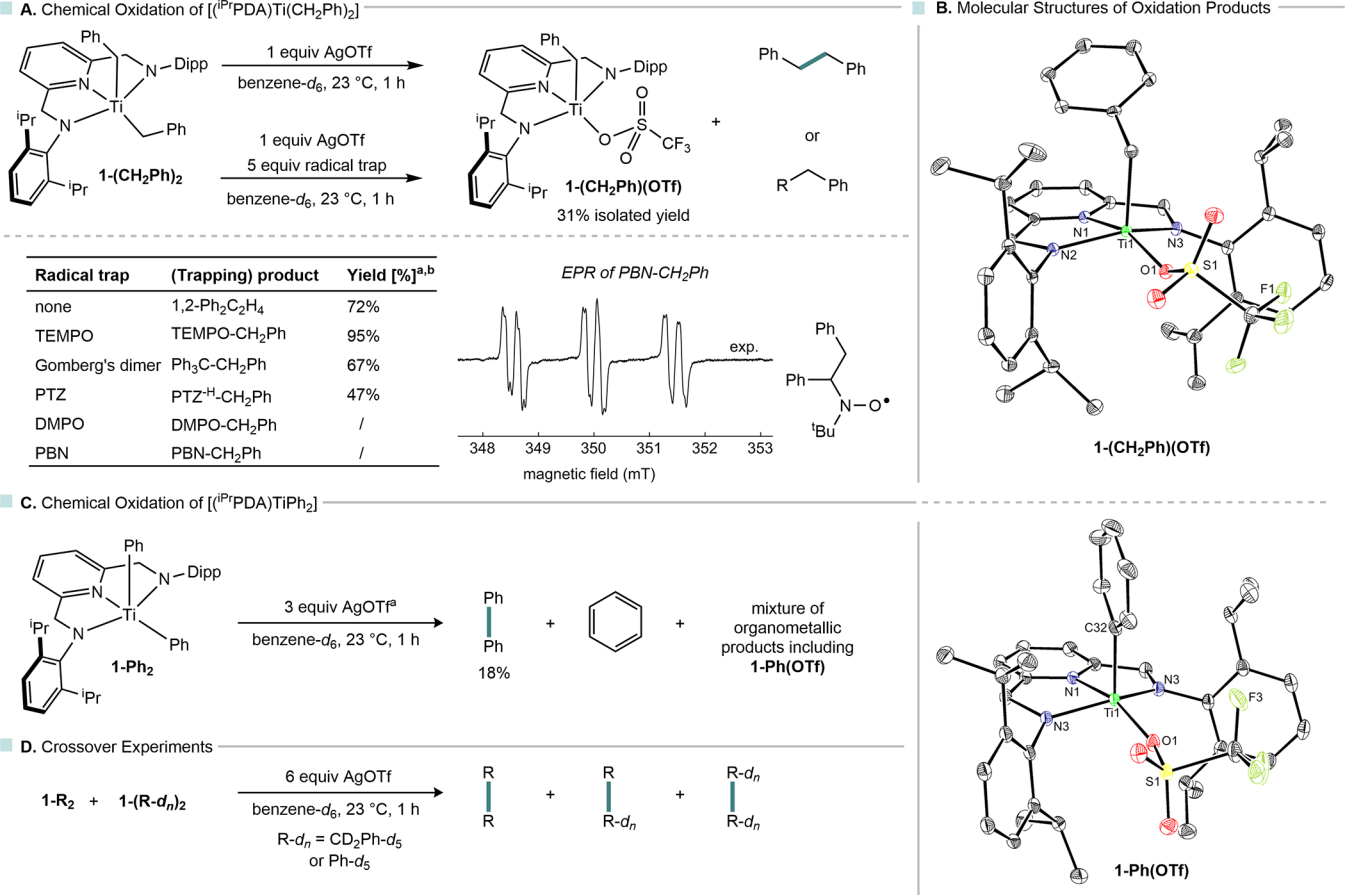
Chemical oxidation of (^iPr^PDA)­TiR_2_: oxidation results for R = CH_2_Ph (A), molecular structure of oxidation product in the solid state (B), chemical oxidation results for R = Ph (C), and crossover experiments (D). Molecular structures are shown with 30% probability ellipsoids, and hydrogen atoms are omitted for clarity; ^a^ yields were determined by ^1^H NMR spectroscopy using HDMSO as internal standard; ^b^ the maximum yield of 100% is based on the elimination of one benzyl substituent from **1-(CH**_**2**_ **Ph)**_**2**_.

### Chemical Oxidation of PDI Complexes

The ligand-based HOMO in **2-(CH**_**2**_ **Ph)**_**2**_, together with its distinct electrochemistry, motivated further studies of its oxidation chemistry. Based on the low oxidation potential of −0.4 V, chemical oxidation reactions with a series of oxidants were conducted (Figure 6A). Initial experiments varying the stoichiometry of the oxidant revealed that, in general, the use of two equivalents led to higher yields of organic products. A screening of different oxidizing agents, including both outer- and inner-sphere oxidants, showed that ferrocenium salts, such as those with tetrafluoroborate or the weakly coordinating BAr^F^_4_^–^ anions, produced no detectable amounts of bibenzyl even though full consumption of **2-(CH**_**2**_ **Ph)**_**2**_ was shown by ^1^H NMR spectroscopy. Interestingly, very weakly oxidizing tropylium salts also led to complete consumption of **2-(CH**_**2**_ **Ph)**_**2**_, but again, no bibenzyl was detected. Among the halogen-based oxidants, both I_2_ and PhICl_2_ yielded low amounts of bibenzyl (16%), whereas silver triflate (AgOTf) afforded 30% bibenzyl and 10% toluene. To gain mechanistic insight, the isotopologue **2-(CD**_**2**_ **Ph** **-*d*_5_)_2_** was prepared and subjected, together with **2-(CH**_**2**_ **Ph)**_**2**_, to AgOTf in a crossover experiment (Figure 6B). Product analysis by ^1^H NMR and mass spectrometry revealed bibenzyl-*d*_7_, which is consistent with a radical mechanism. Given the full consumption of the starting material and the modest yields of bibenzyl and toluene, the reaction with iodine was selected for closer examination. Crystallization of the product mixture yielded a single crystal of a titanium diiodido complex bearing a PDI ligand substituted with benzyl groups (**2** ^**Bn**^ **-I**_**2**_, Figure 6B). This suggests that oxidation generates benzyl radicals, which subsequently undergo addition to the PDI ligand. Further radical trapping experiments using AgOTf in the presence of different radical traps confirmed the formation of the corresponding adduct for TEMPO and Gomberg’s dimer (*vide supra*). The overall yield of organic benzyl-containing products amounts to 68% and 92%, respectively (100% yield based on elimination of both benzyl substituents), suggesting that both benzyl substituents of **2-(CH**_**2**_ **Ph)**_**2**_ are eliminated. To probe the generality of this alkyl radical expulsion, the dimethyl complex **2-Me**_**2**_ was synthesized according to a literature procedure and reacted with iodine.^21a^ Similar to **2-(CH**_**2**_ **Ph)**_**2**_, the major product was the corresponding alkyl iodide (MeI) as evidenced by a singlet in the ^1^H NMR spectrum at 1.43 ppm. In contrast to the reaction with **2-(CH**_**2**_ **Ph)**_**2**_, small amounts of methane rather than ethane were detected in the reaction mixture. Overall, the oxidation chemistry of **2-(CH**_**2**_ **Ph)**_**2**_ indicates that, similar to **1-(CH**_**2**_ **Ph)**_**2**_, the complex undergoes alkyl radical expulsion, but this is induced by oxidation of the ligand rather than the Ti–C bonds. However, in the case of **2-(CH**_**2**_ **Ph)**_**2**_, the redox-active PDI ligand can trap the expelled radicals via addition at the imine and 4-position of the pyridine ring.

**Figure 6:**
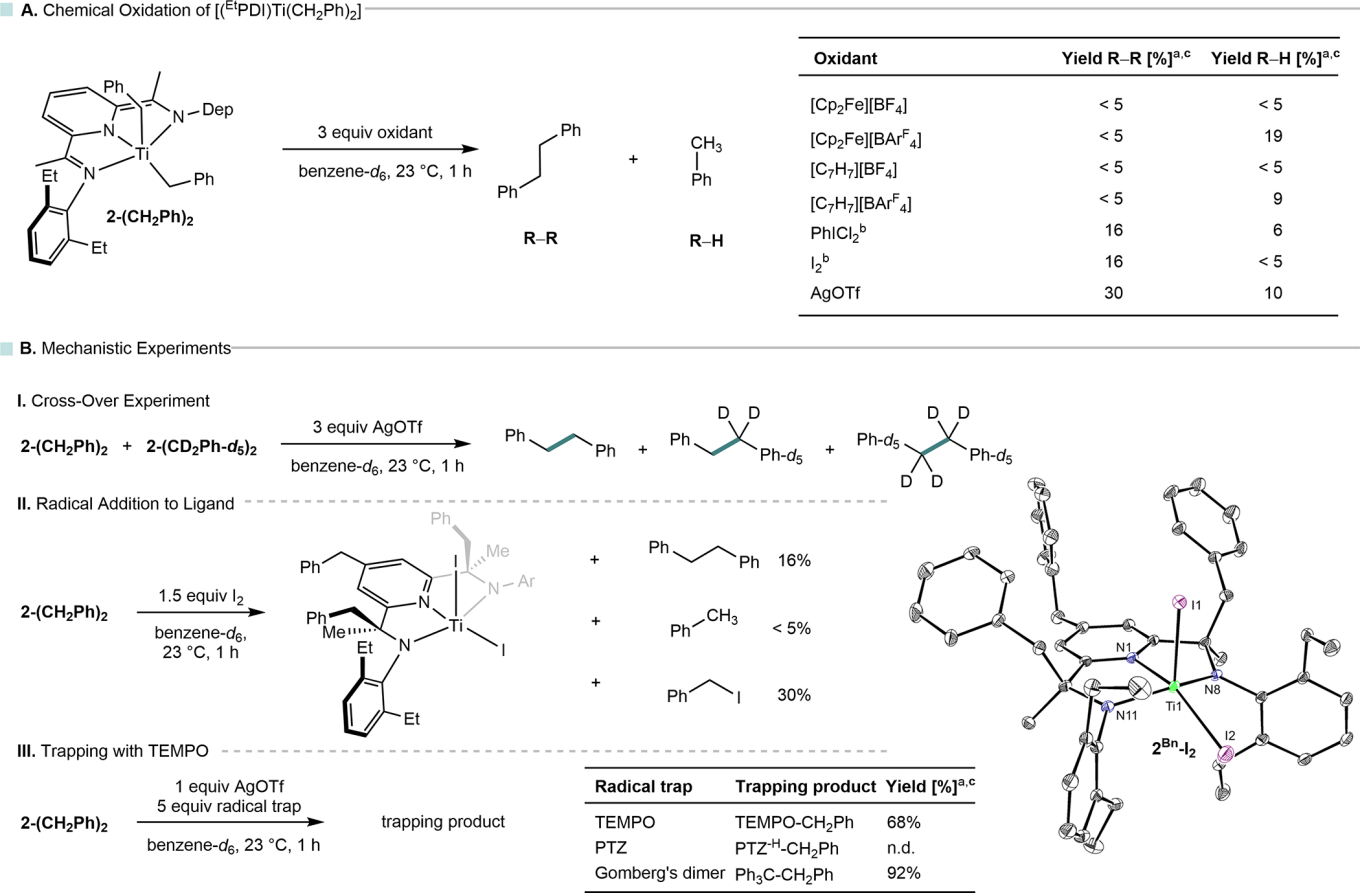
Oxidation chemistry of **2-(CH**_**2**_ **Ph)**_**2**_ including chemical oxidation results (A) and mechanistic experiments (B); molecular structure in the solid state with 30% probability ellipsoids; hydrogen atoms and the minor component that does not show benzyl substitution on the pyridine are omitted for clarity; ^a^ yields were determined by ^1^H NMR spectroscopy using HMDSO as internal standard; ^b^ in reactions with I_2_ and PhICl_2_, only 1.5 instead of 3.0 equiv were used; ^c^ maximum yield of 100% is based on elimination of two benzyl substituents from **2-(CH**_**2**_ **Ph)**_**2**_; n.d. = not detected.

Finally, the oxidation chemistry of biaryl complexes **2-Ar**_**2**_ was explored. In analogy to **2-(CH**_**2**_ **Ph)**_**2**_, chemical oxidations were screened with a series of different oxidants (Figure 7A). For this purpose, **2-(*p*-Tol)**_**2**_ was selected, as both the biaryl and the arene can conveniently be detected via their methyl resonances in the ^1^H NMR spectrum. Reactions with ferrocenium and tropylium salts afforded good yields of the corresponding biaryl, i.e., 74% for [Cp_2_Fe]^+^ and 69% for [C_7_H_7_]^+^, when BF_4_^–^ was used as the counterion. Notably, in the presence of pyridine, [BF_3_·pyr] was detected as a byproduct in the ^11^B (δ = 1.24 ppm) and ^19^F NMR spectra (δ = −150.5 ppm), suggesting fluoride-ion transfer to titanium. In contrast, when the same reactions were carried out using the noncoordinating BAr^F^_4_^–^ anion, significantly lower yields of bis­(*para*-tolyl) were obtained (48% for [Cp_2_Fe]­[BAr^F^_4_] and 35% for [C_7_H_7_]­[BAr^F^_4_]) accompanied by increased amounts of toluene (37% and 33%, respectively). These results indicate that noncoordinating anions promote C–H elimination pathways, possibly due to the generation of a highly reactive, coordinatively unsaturated titanium intermediate. Remarkably, when PhICl_2_ was used as the oxidant, a high yield of bis­(*para*-tolyl) (92%) and only 9% toluene were observed. Most notably, quantitative formation of the biaryl with no detectable toluene was achieved when silver triflate or iodine were used as oxidants. Furthermore, electrochemical oxidation of solutions of **2-(*p*-Tol)_2_** in THF with [^n^Bu_4_N]­[BAr^F^_4_] as the electrolyte afforded the biaryl in good yields of 65%, showing the potential of this method in electrocatalysis. To further assess the suitability for (catalytic) C–C bond formation, a synthetic cycle involving sequential addition of ArLi (Ar = Ph, *p*-Tol) followed by iodine oxidation was carried out (see Figures S1 and S2 in the Supporting Information). Remarkably, the yield of biphenyl formation remained at 80% after five cycles and above 40% after ten cycles, demonstrating the selectivity and the potential of this strategy for future applications in catalysis.

**Figure 7:**
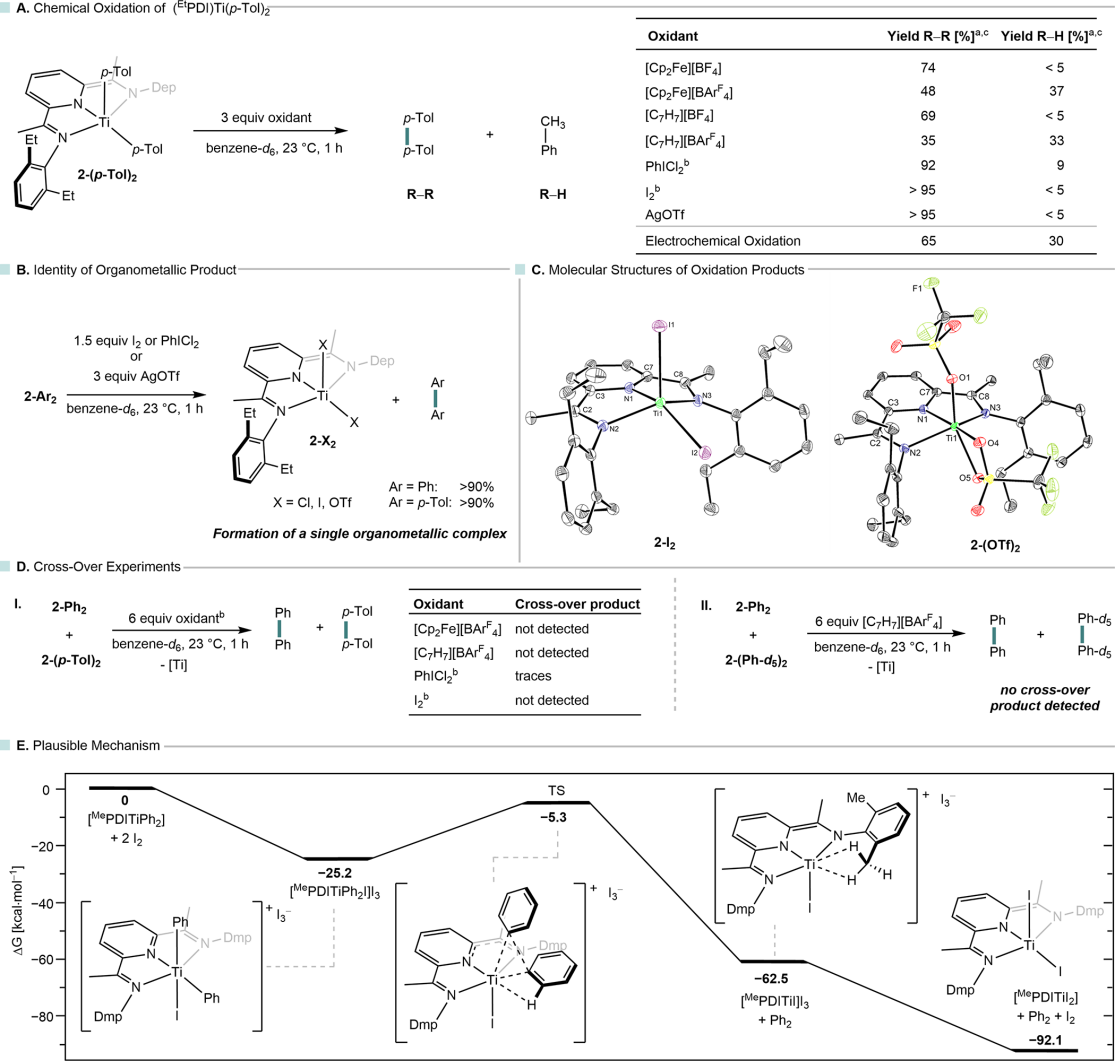
Oxidation chemistry of **2-(*p*-Tol)_2_**: chemical oxidation results (A), identity of the organometallic product (B), and their molecular structures in the solid state with 30% probability ellipsoids (C); crossover experiments (D); hydrogen atoms are omitted for clarity; plausible mechanism calculated by DFT (E, PBE0-D4/def2-TZVPP//PBE0-D4/def2-TZVP); ^a^ yields were determined by ^1^H NMR spectroscopy using HMDSO as the internal standard and 100% yield corresponds to elimination of both aryl substituents; ^b^ in reactions with I_2_ and PhICl_2_, only 1.5 instead of 3.0 equiv were used.

Closer inspection of the reactions affording high biaryl yields using PhICl_2_, I_2_ and AgOTf revealed that in all cases, (^Et^PDI)­TiX_2_ (**2-X**_**2**_, X = Cl,^21a^ I, OTf) was formed as the organometallic product (Figure 7B). The formation of **2-X**_**2**_ was confirmed by single-crystal X-ray diffraction (X = OTf, I; Figure 7C), NMR spectroscopy (X = Cl, I), and independent syntheses starting from **2-Cl**_**2**_ (for **2-I**_**2**_). Notably, for X = I, complex **2-I**_**2**_ is formed quantitatively. While **2-(OTf)**_**2**_ was unambiguously identified by SCXRD, exposure to AgOTf leads to further oxidation, giving rise to paramagnetic **2-(OTf)**_**3**_ (see the SI). The structural parameters of **2-I**_**2**_ resemble those of the previously reported dichlorido complex **2-Cl**_**2**_,^21a^ particularly in the Δ-value of 0.055 (**2-Cl**_**2**_: Δ = 0.055). Similarly, triflate complex **2-(OTf)**_**2**_ has a Δ-value of 0.064.

Having established that **2-Ar**_**2**_ complexes undergo clean, selective biaryl elimination upon oxidation with several oxidants, we sought mechanistic insight through crossover experiments to differentiate between radical and concerted pathways (Figure 7D). Treatment of equimolar mixtures of **2-Ph**_**2**_ and **2-(*p*-Tol)**_**2**_ with iodine, PhICl_2_, [Cp_2_Fe]­[BAr^F^_4_] or [C_7_H_7_]­[BAr^F^_4_] yielded only biphenyl and *p,p’*-bitolyl with only trace or nondetectable amounts of the crossover product as evidenced by ^1^H NMR spectroscopy, GC-MS, and HRMS (Figure 7D,I). Similarly, the oxidation of a mixture of **2-Ph**_**2**_ and **2-(Ph-*d*_5_)_2_** produced only biphenyl and biphenyl-*d*_10_, with no detectable crossover product in high-resolution mass spectrometry (Figure 7D,II). These findings strongly support concerted C­(sp^2^)–C­(sp^2^) reductive elimination from a Ti­(IV) intermediate induced by oxidation. The observation of a concerted C–C reductive elimination from Ti­(IV) upon oxidation is highly unusual and, to date, largely undocumented, highlighting the potential of tridentate redox-active ligands to unlock new elementary steps in titanium chemistry.^37^

Given the electrochemically observed reversible first oxidation, attempts were made to isolate a cationic intermediate using the one-electron oxidants AgOTf and [Cp_2_Fe]­X (X = BF_4_^–^, BArF_4_^–^). However, the addition of only one equivalent of oxidant to **2-Ar**_**2**_ (Ar = Ph, *p*-Tol) consistently led to incomplete consumption of the starting material and yields of the biaryl product of ca. 50%. Attempts to detect such a short-lived species by EPR spectroscopy at low temperatures were also unsuccessful (see SI). Hence, for one-electron oxidants, the data support a mechanism involving an initial reversible ligand oxidation, followed by an irreversible second oxidation that induces concerted reductive elimination. Computational analysis of the one- and two-electron oxidized complexes [**2-(** *p* **-Tol)**_**2**_]^**+** *n*^ (*n = 1, 2*) revealed a shortening of both Ti–C bond lengths upon oxidation and similarly a decreasing C···C distance (x = 0: 3.672, X = 1: 3.558, X = 2: 3.423 Å). To gain deeper insight into the oxidation mechanism with two-electron oxidants, quantum chemical calculations were performed for the reaction of the truncated model complex (^Me^PDI)­TiPh_2_ with iodine (PBE0-D4/def2-TZVPP//PBE0-D4/def2-TZVP level of theory; CPCM benzene).^38^ The initial two-electron ligand oxidation affords the cationic octahedral complex [(^Me^PDI)­Ti­(Ph)_2_I]­[I]. Subsequent reaction with I_2_ gives [(^Me^PDI)­Ti­(Ph)_2_I]­[I_3_] with a total Gibbs free energy change of −25.2 kcal·mol^–1^. A three-membered transition state was located for the concerted elimination process, featuring an activation barrier of ΔG^‡^ = 19.9 kcal·mol^–1^ and the bond metric data for this structure indicates a monoanionic ^Me^PDI^–^ ligand, which is consistent with metal-ligand cooperativity in the reductive elimination process. Reductive elimination affords [(^Me^PDI)­TiI]­[I_3_], in which the titanium center is stabilized by two agostic interactions. Finally, coordination of I^–^ affords [(^Me^PDI)­TiI_2_] with an overall reaction free energy of −92.1 kcal·mol^–1^.^39,40^

## Conclusions

In summary, control over the reductive-elimination reactivity of titanium­(IV) dialkyl and diaryl complexes was achieved through three key parameters (Figure 8):

**Figure 8:**
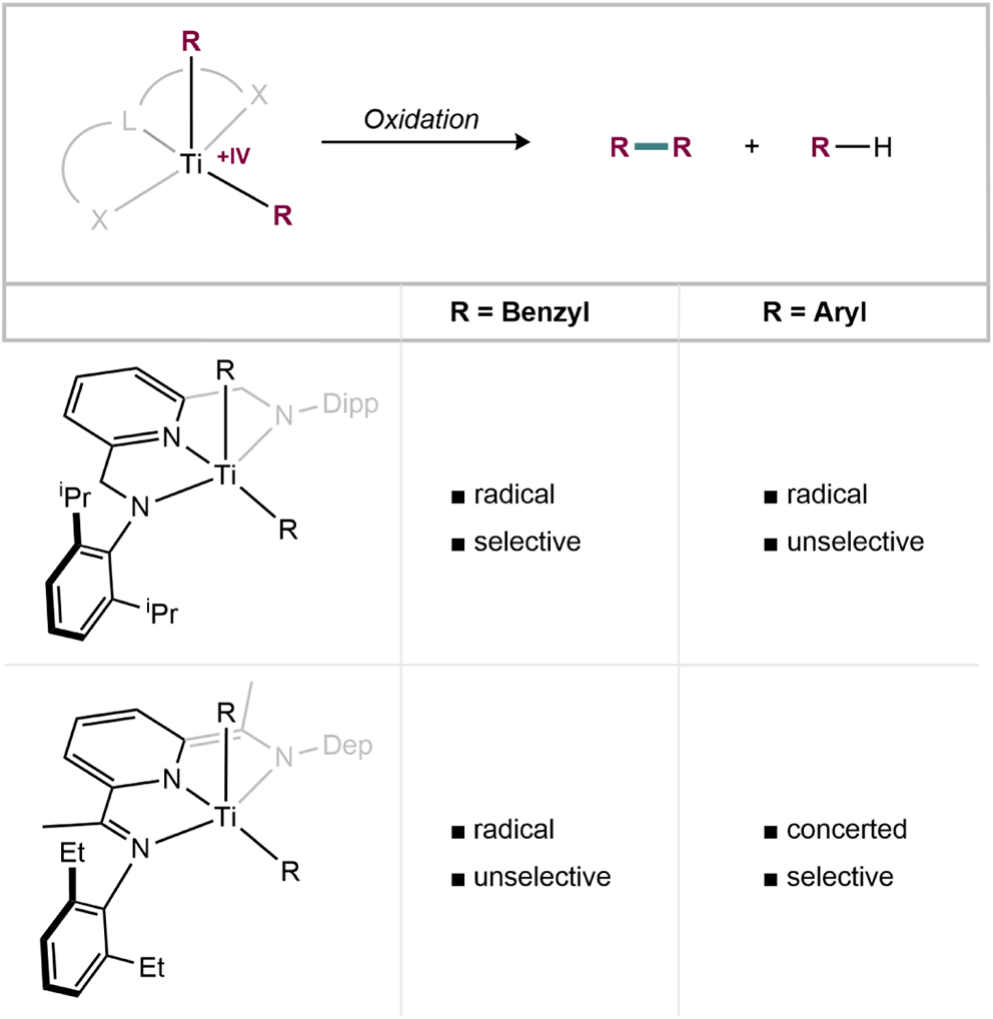
Summary of the ligand and substituent effects on oxidatively induced reductive elimination in titanium pincer complexes.

**1.** Ligand effects: The choice of a rigid pincer ligand proved decisive for steering one- versus two-electron pathways. Pyridinediamido (PDA) complexes exhibit HOMOs localized on Ti–C σ-bonds or nitrogen atoms, favoring radical formation upon oxidation. In contrast, the introduction of a redox-active pyridinediimine (PDI) ligand fundamentally alters the electronic structure to ligand-centered and delocalized HOMOs, enabling a rare, concerted C­(sp^2^)–C­(sp^2^) reductive elimination from Ti­(IV) with high selectivity and quantitative biaryl formation.

**2.** Substituent effects (C­(sp^3^) vs C­(sp^2^)): Oxidation of the dibenzyl PDA complex **1-(CH**_**2**_ **Ph)**_**2**_ results in selective benzyl-radical generation, whereas the analogous diphenyl complex **1-Ph**_**2**_ shows unselective oxidation chemistry. In contrast, the PDI-supported diaryl complexes **2-Ar**_**2**_ (Ar = Ph, *p*-Tol) undergo concerted biaryl reductive elimination, while the PDI dibenzyl complex releases benzyl radicals. These results highlight how substituent hybridization strongly influences whether radical expulsion or concerted C–C coupling is favored.

**3.** Oxidant effects: The nature of the oxidant further controls the reaction outcomes. Inner-sphere oxidants with coordinating anions promote selective reactions, enabling either controlled radical generation or concerted reductive elimination (e.g., AgOTf).

Finally, the potential of these transformations for catalysis was demonstrated by achieving reductive elimination under electrochemical conditions and a synthetic cycle for biaryl formation. Together, these results show that ligand design, substituent choice, and oxidant are handles for directing reductive elimination pathways in titanium chemistry and open new avenues toward catalytic C–C bond-forming processes. Ongoing studies aim to explore the potential of these reactions in (electro)­catalysis.

## Supplementary Material



## Notes

Experimental details, NMR spectra, cyclic voltammetry data, single-crystal X-ray diffraction data, and computational details (PDF)

## References

[1] Miyaura N., Suzuki A. (1995). Palladium-Catalyzed
Cross-Coupling
Reactions of Organoboron Compounds. Chem. Rev..

[2] Levin M. D., Kim S., Toste F. D. (2016). Photoredox Catalysis Unlocks Single-Electron Elementary
Steps in Transition Metal Catalyzed Corss-Coupling. ACS Cent. Sci..

[3] Qiao B., Lin F.-Y., Fu D., Li S.-J., Zhang T., Lan Y. (2024). Mechanistic insights
into facilitating
reductive elimination from Ni­(II) species. Chem.
Commun..

[4] Tsou T. T., Kochi J. K. (1978). Reductive coupling
of organometals induced by oxidation. Detection of metastable paramagnetic
intermediates. J. Am. Chem. Soc..

[5] Li L., Brennessel W. W., Jones W. D. (2008). An efficient low-temperature
route to polycyclic isoquinoline salt synthesis via C-H activation
with [Cp*MCl_2_]_2_ (M = Rh, Ir). J. Am. Chem. Soc..

[6] Kalyani D., Deprez N. R., Desai L. V., Sanford M. S. (2005). Oxidative C-H activation/C-C bond forming reactions:
synthetic scope and mechanistic insights. J.
Am. Chem. Soc..

[7] Lagueux-Tremblay P.-L., Tam K. M., Jiang M., Arndtsen B. A. (2025). Electrifying Redox-Neutral
Palladium-Catalyzed Carbonylations: Multielectron Transfer as a Catalyst
Driving Force. J. Am. Chem. Soc..

[8] Burk M. J., Tumas W., Ward M. D., Wheeler D. R. (1990). Oxidation
chemistry of d^0^ organometallic complexes. J. Am. Chem. Soc..

[9] Borkowsky S. L., Baenziger N. C., Jordan R. F. (1993). Generation and reactivity
of titanocene benzyl Cp_2_Ti­(CH_2_Ph)­(L)^+^ complexes. Oxidation and protonolysis chemistry of Cp_2_Ti­(CH_2_Ph)_2_. Organometallics.

[10] Zheng L., Cui Y.-S., Chen D.-P., Li G.-M., Liu F., Zhai D.-D., Shi Z.-J. (2025). Terminal
Vanadium Hydride through
Oxidative C-H Cleavage and Its Application in Reduction of O_2_. J. Am. Chem. Soc..

[11] Beaumier E. P., Pearce A. J., See X. Y., Tonks I. A. (2019). Modern applications
of low-valent early transition metals in synthesis and catalysis. Nat. Rev. Chem..

[12] A rare example of an OIRE reaction with group IV metallocenes that proceeds selectively and where the oxidation product was characterized is described in reference 8.

[13] Ho S. C. H., Straus D. A., Grubbs R. H. (1984). An alternate path
to reductive elimination for Group IVB metals: mechanism of cyclopropane
formation from titanacyclobutanes. J. Am. Chem.
Soc..

[14] Haneline M. R., Heyduk A. F. (2006). C-C bond-forming
reductive elimination from a zirconium­(IV)
redox-active ligand complex. J. Am. Chem. Soc..

[15] Ashley D. C., Baik M.-H. (2015). How a Redox-Innocent
Metal Promotes the Formal Reductive
Elimination of Biphenyl Using Redox-Active Ligand. Chem. - Eur. J..

[16] Blackmore K. J., Sly M. B., Haneline M. R., Ziller J. W., Heyduk A. F. (2008). Group IV
imino-semiquinone complexes obtained by oxidative addition of halogens. Inorg. Chem..

[17] McCallum T., Wu X., Lin S. (2019). Recent Advances
in Titanium Radical Redox Catalysis. J. Org.
Chem..

[18] Guérin F., McConville D. H., Payne N. C. (1996). Conformationally Rigid Diamide Complexes: Synthesis
and Structure of Titanium­(IV) Alkyl Derivatives. Organometallics.

[19] Low J. J., Goddard W. A. (1986). Theoretical studies of oxidative
addition and reductive
elimination. 3. Carbon-hydrogen and carbon-carbon reductive coupling
from palladium and platinum bis­(phosphine) complexes. J. Am. Chem. Soc..

[20] Fritsche P., Geyer L., Czernetzki C., Hierlmeier G. (2024). Coordination-induced
reductive elimination from a titanium­(IV) complex. Chem. Commun..

[21] Rahimi N., de Bruin B., Budzelaar P. H. M. (2017). Balance
between Metal and Ligand Reduction in Diiminepyridine Complexes of
Ti. Organometallics.

[22] Addison A. W., Rao T. N., Reedijk J., van Rijn J., Verschoor G. C. (1984). Synthesis, structure, and spectroscopic
properties of copper­(II) compounds containing nitrogen–sulphur
donor ligands; the crystal and molecular structure of aqua­[1,7-bis­(N-methylbenzimidazol-2′-yl)-2,6-dithiaheptane]­copper­(II)
perchlorate. J. Chem. Soc., Dalton. Trans.

[23] Agapie T., Henling L. M., DiPasquale A. G., Rheingold A. L., Bercaw J. E. (2008). Zirconium and Titanium Complexes Supported by Tridentate
LX_2_ Ligands Having Two Phenolates Linked to Furan, Thiophene,
and Pyridine Donors: Precatalysts for Propylene Polymerization and
Oligomerization. Organometallics.

[24] Brookhart M., Green M. L. H., Parkin G. (2007). Agostic interactions
in transition
metal compounds. Proc. Natl. Acad. Sci..

[25] Esteruelas M. A., López A. M., Mateo A. C., Oñate E. (2006). New Half-Sandwich
Alkyl, Aryl, Aryloxide, and Propargyloxide Titanium­(IV) Complexes
Containing a Cyclopentadienyl Ligand with a Pendant Ether Substituent:
Behavior and Influence in the Hydroamination of Alkynes of the Ether
Group. Organometallics.

[26] Olthof G. J., van Bolhuis F. (1976). Structure of (2,6-dimethylphenyl)­dicyclopentadienyltitanium­(III). J. Organomet. Chem.

[27] Buchwald S. L., Nielsen R. B. (1988). Group 4 metal complexes of benzynes,
cycloalkynes, acyclic alkynes, and alkenes. Chem. Rev..

[28] Römelt C., Weyhermüller T., Wieghardt K. (2019). Structural characteristics of redox-active
pyridine-1,6-diimine complexes: Electronic structures and ligand oxidation
levels. Coord. Chem. Rev..

[29] Chu T., Belding L., Poddutoori P. K., van der Est A., Dudding T., Korobkov I., Nikonov G. I. (2016). Unique
molecular geometries of reduced 4- and 5-coordinate zinc complexes
stabilised by diiminopyridine ligand. Dalton
Trans..

[30] Laviron E. (1979). General expression
of the linear potential sweep voltammogram in the case of diffusionless
electrochemical systems. J. Electroanal. Chem..

[31] Connelly N. G., Geiger W. E. (1996). Chemical Redox Agents
for Organometallic Chemistry. Chem. Rev..

[32] Yasu Y., Koike T., Akita M. (2012). Visible Light-Induced Selective Generation
of Radicals from Organoborates by Photoredox Catalysis. Adv. Synth. Catal..

[33] Kleeberg C. (2013). On the structural diversity of K­(18-crown-6)­EPh_3_ complexes (E = C, Si, Ge, Sn, Pb): synthesis, crystal structures
and NOESY NMR study. Dalton Trans..

[34] Mörsdorf J.-M., Ballmann J. (2023). Coordination-Induced Radical Generation:
Selective
Hydrogen Atom Abstraction via Controlled Ti-C σ-Bond Homolysis. J. Am. Chem. Soc..

[35] Basuli F., Bailey B. C., Tomaszewski J., Huffman J. C., Mindiola D. J. (2003). A Terminal and Four-Coordinate Titanium
Alkylidene Prepared by Oxidatively Induced α-Hydrogen Abstraction. J. Am. Chem. Soc..

[36] Zhang Y., Petersen J. L., Milsmann C. (2018). Photochemical
C–C Bond Formation in Luminescent Zirconium Complexes with
CNN Pincer Ligands. Organometallics.

[37] Szigethy G., Heyduk A. F. (2011). Steric and electronic consequences
of flexibility in a tetradentate redox-active ligand: Ti­(IV) and Zr­(IV)
complexes. Inorg. Chem..

[38] Gossage R. A., Ryabov A. D., Spek A. L., Stufkens D. J., van Beek J. A. M., van Eldik R., van Koten G. (1999). Models for the Initial Stages of Oxidative Addition.
Synthesis, Characterization, and Mechanistic Investigation of η^1^-I_2_ Organometallic “Pincer” Complexes
of Platinum. X-ray Crystal Structures of [PtI­(C_6_H_3_{CH_2_NMe_2_}­2–2,6)­(η^1^-I_2_)] and exo-meso-[Pt­(η^1^-I_3_)­(η^1^-I_2_)­(C_6_H_3_{CH_2_N­(t-Bu)­Me}­2–2,6)]. J. Am. Chem. Soc..

[39] Notably, LOBA calculations (see SI) showed an oxidation state of +4 throughout the reductive elimination reaction for all intermediates.

[40] Alternatively, a mechanism where two aryl radicals are ejected and recombine in the coordination sphere is possible here. While we cannot exclude such a scenario, a concerted mechanism is viable according to our DFT calculations.

