# Supplementary material for: Controlling Reductive Elimination Pathways in Ti(IV) Pincer Complexes: Concerted versus Radical Mechanisms via Ligand Design
Source: J Am Chem Soc. 2026 Jan 22;148(4):4694–705. doi: 10.1021/jacs.5c21215 (PMC12879738; doi:10.1021/jacs.5c21215)
Supplement: Supplementary file 1 [file ja5c21215_si_001.pdf]

Supplementary Information for

Controlling Reductive Elimination Pathways in Ti(IV) Pincer Complexes: Concerted versus Radical Mechanisms via Ligand Design

Paul Fritsche, Corinna Czernetzki, Maxi Liesa Heldner, Laura Hörlin, Ivo Krummenacher, Gabriele Hierlmeier*

Institute for Inorganic Chemistry, Julius-Maximilians-Universität Würzburg, Am Hubland, 97074 Würzburg, Germany

E-mail: gabriele.hierlmeier@uni-wuerzburg.de

Table of Contents

1. Methods and materials ... 2
2. Synthetic procedures for novel compounds ... 4
3. Synthetic procedures for reactivity & mechanistic studies ... 13
4. NMR spectra of isolated compounds ... 18
5. NMR spectra of OIRE and trapping reactions ... 28
6. EPR spectra of trapping reactions ... 43
7. GC and MS Data ... 44
8. X-ray crystallographic details ... 58
9. Cyclic and Differential Pulse Voltammetry ... 62
10. Quantum Chemical Calculations ... 64
11. References ... 87

1. Methods and materials

All manipulations were performed either under an atmosphere of dry argon or *in vacuo* using standard Schlenk line or glovebox techniques. Deuterated solvents were dried over molecular sieves and degassed by three freeze-pump-thaw cycles prior to use. All other solvents were distilled and degassed from appropriate drying agents. Both deuterated and non-deuterated solvents were stored under argon over activated 4 Å molecular sieves.

NMR spectra were acquired either on a Bruker Avance 600 (operating at 600 MHz for ^{1}H and 151 MHz for ^{13}C), a Bruker Avance 500 (operating at 500 MHz for ^{1}H, 126 MHz for ^{13}C, and 471 MHz for ^{19}F) or a Bruker Avance 400 NMR (operating at 400 MHz for ^{1}H and 100 MHz for ^{13}C). Chemical shifts (δ) are given in ppm and internally referenced to the carbon nuclei (^{13}C) or residual protons (^{1}H) of the solvent or externally for ^{19}F using CCl_3F.

EPR measurements at X-band (9.83 GHz) were carried out using a Bruker ELEXSYS E580 CW EPR spectrometer. The spectral simulations were performed using MATLAB 24.2.0.2773142 (R2024b) and the EasySpin 6.0.10 toolbox.[1] Experiments were conducted with following parameters: microwave frequency = 9.83 GHz; microwave power = 2 mW; modulation amplitude = 0.2 G; conversion time = 60 ms; modulation frequency = 100 kHz.

HRMS spectra were acquired using an Exactive Plus mass spectrometer with an Orbitrap analyser (Thermo Scientific). Ionisation was achieved using the APCI method.

GCMS data were acquired using an Agilent 7890A gas chromatograph with an Agilent 5975C inert XL EI/CI mass selective detector.

Microanalyses (C, H, N, S) were performed on an Elementar Vario MICRO cube elemental analyzer at the Julius-Maximilians-University Würzburg and the University of Regensburg.

Electrochemical reactions were carried out using an undivided cell setup with two graphite electrodes (rod with ⌀ = 4.0 mm, ECO quality, purchased from graphite24.com) and a Rohde & Schwarz NGM 7.5/4 laboratory power supply (output voltage 0−7.5 V, output current 0−4 A DC).

(^{iPr}PDA)Ti(CH_2Ph)$_2$,[2] (^{iPr}PDA)Ti(CD_2Ph-*d*$_5$)$_2$,[2] (^{iPr}PDA)$TiCl_2$,[3] (^{Et}PDI)$TiCl_2$,[4] PhLi,[5] Ph-*d*$_5$Li,[5] *p*-TolLi,[5] Mg(CH_2Ph)$_2$,[6] Mg(CD_2Ph-*d*$_5$)$_2$,[6] [C_7H_7][BAr$^F_4$],[7] [Cp_2Fe][BAr$^F_4$],[8] and $PhICl_2$[9] were prepared following literature procedures. All other solid reagents were dried under reduced pressure and transferred into a glovebox prior to use. Liquid

reagents were degassed and dried over activated 4 Å molecular sieves and distilled under reduced pressure or filtered prior to use.

2. Synthetic procedures for novel compounds

(^{iPr}PDA)Ti(CH_2Ph)(OTf)

In a 20 mL vial, a suspension of AgOTf (23 mg, 87 µmol, 1.00 equiv.) in benzene (2 mL) was added to suspension of (^{iPr}PDA)Ti(CH_2Ph)$_2$ (60 mg, 87 µmol, 1.00 equiv.) at ambient temperature, affording a brown suspension. After stirring the reaction mixture for 1 hour, the solvent was removed *in vacuo*. The dark red residue was washed with benzene (3×1 mL) and the remaining solid was extracted with THF. Red crystals of (^{iPr}PDA)Ti(CH_2Ph)(OTf) were obtained after storing the THF solution at ambient temperature for one week and were dried *in vacuo*. Single crystals suitable for X-ray analysis were grown by storing a saturated solution of (^{iPr}PDA)Ti(CH_2Ph)(OTf) in THF at ambient temperature.

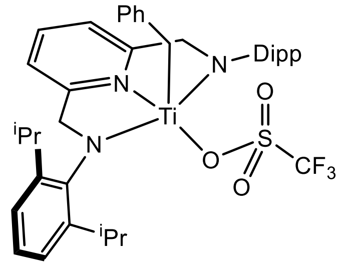

$C_{39}H_{48}F_3N_3O_3STi$, 743.75 g/mol

Yield: 20 mg (31%)

^{1}H NMR (500 MHz, 298 K, benzene-*d*$_6$): δ = 0.93 (d, ^3J = 6.8 Hz, 6H, C*H*$_3$), 1.38 (d, ^3J = 6.9 Hz, 6H, C*H*$_3$), 1.47 (d, ^3J = 6.9 Hz, 6H, C*H*$_3$), 1.63 (d, ^3J = 6.9 Hz, 6H, C*H*$_3$), 2.78 (ps. sept, ^3J = 6.9 Hz, 2H, C*H*(CH_3)$_2$), 3.61 (s, 2H, C*H*$_2$Ph), 4.47 (ps. sept, ^3J = 6.9 Hz, 2H, C*H*(CH_3)$_2$), 4.64 (d, ^3J = 21.5 Hz, 2H, C*H*$_2$N), 5.40 (d, ^3J = 21.7 Hz, 2H, C*H*$_2$N), 6.17 (d, ^3J = 7.7 Hz, 2H, *m*-C*H*$_{Pyr}$), 6.55–6.60 (m, 1H, *p*-CH$_{Ph}$), 6.68 (t, ^3J = 7.7 Hz, 1H, *p*-C*H*$_{pyr}$), 6.77–6.83 (m, 2H, *m*-C*H*$_{Ph}$), 6.88–6.93 (m, 2H, *o*-C*H*$_{Ph}$), 7.24 (dd, *J* = 2.0, 7.7 Hz, 2H, *m*-C*H*$_{Dipp}$), 7.27 (t, ^3J = 7.5 Hz, 2H, *p*-C*H*$_{Dipp}$), 7.32 (dd, ^3J = 2.0, 7.4 Hz, 2H, *m*-C*H*$_{Dipp}$) ppm.

^{13}C{^{1}H} NMR (126 MHz, 298 K, benzene-*d*$_6$): δ = 24.1 (s, *C*H$_3$), 24.2 (s, *C*H$_3$), 26.4 (s, *C*H$_3$), 26.5 (s, *C*H$_3$), 28.3 (s, *C*H(CH_3)$_2$), 29.7 (s, *C*H(CH_3)$_2$), 69.1 (s, *C*H$_2$N), 84.4 (s, *C*H$_2$Ti), 117.3 (s, *m*-*C*H$_{Pyr}$), 119.4 (q, $^1J_{CF}$ = 319.6 Hz, *C*F$_3$), 122.6 (s, *p*-*C*H$_{Ph}$), 124.7 (s, *m*-*C*H$_{Dipp}$), 125.3 (s, *m*-*C*H$_{Dipp}$), 125.4 (s, *o*-*C*H$_{Ph}$), 127.6 (s, *m*-*C*H$_{Ph}$), 128.0 (s,

p-*C*H$_{Dipp}$, overlapping with benzene-*d*$_6$ signal), 138.8 (s, *p*-*C*H$_{Pyr}$), 142.4 (s, *o*-*C*H$_{Dipp}$), 144.3 (s, *o*-*C*H$_{Dipp}$), 149.1 (s, *i*-*C*H$_{Ph}$), 150.3 (s, *i*-*C*H$_{Dipp}$), 161.6 (s, *o*-*C*H$_{Pyr}$) ppm.
^{19}F{^{1}H} NMR (471 MHz, 298 K, benzene-*d*$_6$): δ = −77.61(s, CF_3) ppm.
Elemental analysis (%) calc. for $C_{39}H_{48}F_3N_3O_3STi$ [743.75 g mol^{-1}]: C 62.98, H 6.51, N 5.65; S 4.31 found: C 63.33, H 6.47, N 5.88 S 4.14.

(^{iPr}PDA)TiPh$_2$

In a 5 mL vial, a suspension of (^{iPr}PDA)TiCl$_2$ (150 mg, 261 µmol, 1.0 equiv.) and PhLi (61 mg, 723 µmol, 2.8 equiv.) in benzene (5 mL) was stirred at ambient temperature for 90 min, affording a golden-brown suspension. Note that longer reaction times and using PhLi in excess were found to lead to decomposition. Subsequently, the solvent was removed *in vacuo*, and the dark brown residue was washed with *n*−pentane. Extraction of the residue with benzene followed by removal of the solvent *in vacuo* afforded the product as a dark brown powder. Crystals suitable for SCXRD were obtained by cooling a saturated *n*-hexane solution of (^{iPr}PDA)TiPh$_2$ to −35 °C.

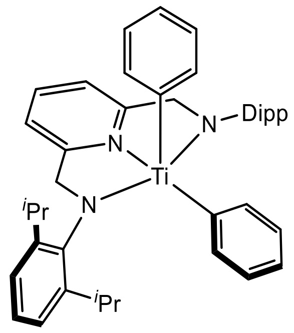

$C_{43}H_{51}N_3Ti$, 657.77 g/mol

Yield: 42.8 mg (25%)

^{1}H NMR (400 MHz, benzene-*d*$_6$) δ = 0.88 (d, ^3J = 6.8 Hz, 12H, CH(C*H*$_3$)$_2$), 1.13 (d, ^3J = 6.8 Hz, 12H, CH(C*H*$_3$)$_2$), 3.51 (ps. sept., ^3J = 6.8 Hz, 4H, C*H*(CH$_3$)$_2$), 5.00 (s, 4H, C*H*$_2$N), 6.48 (d, ^3J = 7.8 Hz, 2H, *m*-C*H*$_{py}$), 6.91 − 6.94 (m, 6H, C*H*$_{Ph/Dipp}$), 6.96 (t, ^3J = 7.8 Hz, 1H, *p*-C*H*$_{py}$), 7.11−7.14 (m, 3H, C*H*$_{Ph/Dipp}$), 7.18−7.23 (m, 5H, C*H*$_{Ph/Dipp}$) ppm.

Two aromatic proton signals were not detected in benzene-*d*$_6$ at 298 K. Due to the limited stability of the complex at ambient temperature, full characterisation by NMR was conducted at 193 K.

^{1}H NMR (600 MHz, 193 K, toluene-d_8) δ = 0.86−0.96 (m, 12H, CH(C*H*$_3$)$_2$), 1.17−1.25 (m, 12H, CH(C*H*$_3$)$_2$), 3.49−3.63 (m, 4H, C*H*(CH$_3$)$_2$), 4.91 (brs, 4H, C*H*$_2$N), 6.17 (d, ^3J = 7.8 Hz, 2H, *m*-C*H*$_{py}$), 6.77 (t, ^3J = 7.7 Hz, 1H, *p*-C*H*$_{py}$), 6.93−6.98 (m, 4H, *m*-C*H*$_{Ph}$), 7.10 (d, ^3J = 7.6 Hz, 4H, *m*-C*H*$_{Dipp}$), 7.13−7.15 (m, 6H, *o*/*p*-C*H*$_{Ph}$), 7.24 (t, ^3J = 7.5 Hz, 2H, *p*-C*H*$_{Dipp}$) ppm.
^{13}C{^{1}H} NMR (151 MHz, 193 K, toluene-d_8) δ = 23.51 (s, CH(*C*H$_3$)$_2$), 26.37 (s, CH(*C*H$_3$)$_2$), 27.94 (s, *C*H(CH$_3$)$_2$), 69.11 (s, *C*H$_2$N), 117.30 (s, *m*-*C*H$_{py}$), 124.16 (s, *m*-*C*H$_{Dipp}$), 125.88 (s, *m*-*C*H$_{Ph}$), 125.98 (s, *p*-*C*H$_{Dipp}$), 128.52 (s, *o*/*p* -*C*H$_{Ph}$), 138.31 (s, *p*-*C*H$_{py}$), 143.56 (s, *o*-*C*$_{Dipp}$), 154.04 (s, *i*-*C*$_{Dipp}$), 161.55 (s, *o*-*C*$_{py}$), 198.85 (s, *i*-*C*$_{Ph}$) ppm.
Elemental analysis (%) calc. for $C_{43}H_{51}N_3Ti$ [657.77 g mol^{-1}]: C 78.52, H 7.82, N 6.39; found: C 77.64, H 8.10, N 5.95.

(^{iPr}PDA)Ti(Ph-d_5)$_2$

In a 5 mL vial, a suspension of (^{iPr}PDA)TiCl$_2$ (20 mg, 35 µmol, 1.00 equiv.) and Ph-d_5Li (13 mg, 146 µmol, 4.2 equiv.) in benzene (1 mL) was stirred at ambient temperature for 30 min, affording a brown suspension. Note that longer reaction times and using Ph-d_5Li in excess were found to lead to decomposition. Subsequently, the solvent was removed *in vacuo*, and the dark brown residue was washed with cold (−35 °) *n*−pentane. Extraction of the residue with benzene followed by removal of the solvent *in vacuo* afforded the product as a dark brown powder.

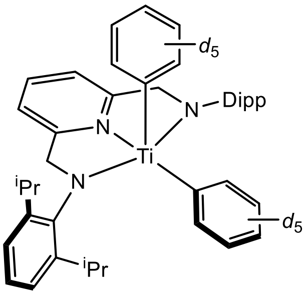

$C_{43}H_{41}D_{10}N_3Ti$, 667.83 g/mol

Yield: 13 mg (56%)

^{1}H NMR (400 MHz, 298 K, benzene-d_6) δ = 0.88 (d, ^3J = 6.8 Hz, 12H, CH(C*H*$_3$)$_2$), 1.13 (d, ^3J = 6.8 Hz, 12H, CH(C*H*$_3$)$_2$), 3.51 (ps. sept., ^3J = 6.8 Hz, 4H, C*H*(CH$_3$)$_2$), 5.00 (s, 4H, C*H*$_2$N), 6.47 (d, ^3J = 7.8 Hz, 2H, *m*-C*H*$_{py}$), 6.96 (t, ^3J = 7.8 Hz, 1H, *p*-C*H*$_{py}$), 7.11−7.14 (m, 4H, *m*-C*H*$_{Dipp}$), 7.18−7.23 (m, 2H, *p*-C*H*$_{Dipp}$) ppm.

(^{Et}PDI)Ti($CH_2Ph)_2$

In a 20 mL vial, a suspension of (^{Et}PDI)$TiCl_2$ (200 mg, 367 µmol, 1.00 equiv.) and $Mg(CH_2Ph)_2$ (127 mg, 615 µmol, 1.67 equiv.) in benzene (7 mL) was stirred at ambient temperature for 2 h. Subsequently, suspended solids were removed *via* filtration over Celite, and the solvent subsequently removed *in vacuo* to afford (^{Et}PDI)Ti($CH_2Ph)_2$ as a black powder which was used without further purification. Single crystals suitable for X-ray diffraction were grown by cooling a saturated solution of (^{Et}PDI)Ti($CH_2Ph)_2$ in *n*-hexane from ambient temperature to −35 °C.

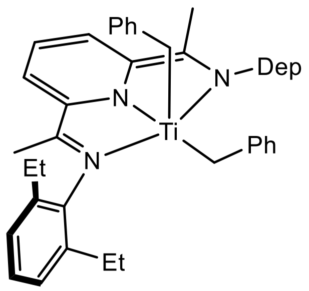

$C_{43}H_{49}N_3Ti$, 655.75 g/mol

Yield: 198 mg (82%)

^{1}H NMR (400 MHz, 298 K, benzene-d_6): δ = 1.10 (t, ^3J = 7.5 Hz, 12H, CH_2CH_3), 1.36 (s, 6H, C(N)CH_3), 2.05−2.25 (brs, 4H, CH_2CH_3), 2.31−2.65 (brs, 4H, CH_2CH_3), 5.42 (t, ^3J = 7.7 Hz, 1H, *p*-CH_{py}), 6.06 (d, ^3J = 7.7 Hz, 2H, *m*-CH_{py}), 7.11−7.13 (brs, 6H, *m*/*p*-CH_{Dep}) ppm.

Due to rapid exchange of the benzyl substituents of the complex in solution, the methylene signals of the benzyl ligand are not observed at room temperature.

^{1}H NMR (500 MHz, 193 K, toluene-d_8): δ = 0.96 (t, ^3J = 7.3 Hz, 6H, CH_2CH_3), 1.15 (s, 2H, CH_2Ph), 1.16 (s, 6H, C(N)CH_3), 1.23 (t, ^3J = 7.3 Hz, 6H, CH_3), 1.46–1.56 (m, 2H, CH_2CH_3), 1.59–1.71 (m, 2H, CH_2CH_3), 2.35–2.46 (m, 2H, CH_2CH_3), 3.19–3.31 (m, 2H, CH_2CH_3), 4.45 (s, 2H, CH_2Ph), 5.38 (t, ^3J = 7.6 Hz, 1H, *p*-CH_{py}), 5.90 (d, ^3J = 7.6 Hz, 2H, *m*-CH_{py}), 5.90 (d, ^3J = 7.0 Hz, 2H, CH_{Ph}), 6.78−6.82 (m, 2H, CH_{Ph}), 6.94 (d, ^3J = 7.7 Hz, 2H, *m*-CH_{Dep}), 7.06−7.08 (m, 2H, CH_{Ph}), 7.08−7.11 (m, 2H, *m*-CH_{Dep}), 7.18 (t, 2H, ^3J = 7.1 Hz, *p*-CH_{Dep}), 7.53−7.60 (m, 4H, CH_{Ph}) ppm.

^{13}C{^{1}H} NMR (125.8 MHz, 193 K, toluene-d8): δ = 12.76 (s, C($CH_3)_2$), 13.72 (s, C($CH_3)_2$), 14.91 (s, C(N)CH_3), 23.45 (s, CH_2CH_3), 23.70 (s, CH_2CH_3), 81.12 (s,

CH_2Ph), 81.29 (s, *C*H_2Ph), 119.31 (s, *p*-*C*H_{Pyr}), 120.43 (s, *p*-*C*H_{Ph}), 124.28 (s, *C*H_{Ph}), 124.68 (s, *C*H_{Ph}), 125.95 (s, *C*H_{Ph}), 126.15 (s, *m*-*C*H_{Dep}), 127.05 (s, *C*H_{Ph}), 127.94 (*overlapping with solvent signal assigned via HSQC and HMBC*, s, *m*-*C*H_{Pyr}), 128.20 (*overlapping with solvent signal assigned via HSQC and HMBC*, s, *C*H_{Ph}), 130.28 (s, *C*H_{Ph}), 133.51 (s, C_{Dep}), 139.21 (s,*C*(N)CH_3), 141.95 (s, C_{Ph}), 147.95 (s, C_{Dep}), 151.43 (s, C_{Ph}), 156.53 (s, *o*-C_{Pyr}) ppm.
Elemental analysis (%) calc. for $C_{43}H_{49}N_3Ti$ [655.75 g mol^{-1}]: C 78.76, H 7.53, N 6.41; found: C 77.32, H 7.65, N 6.26.

(^{Et}PDI)Ti(CD_2Ph-*d*$_5$)$_2$

In a 20 mL vial, a suspension of (^{Et}PDI)$TiCl_2$ (30 mg, 55 μmol, 1.00 equiv.) and Mg(CD_2Ph-*d*$_5$)$_2$ (19 mg, 83 μmol, 1.5 equiv.) in benzene (1 mL) was stirred at ambient temperature for 2 h. Subsequently, suspended solids were removed *via* filtration over Celite, and the solvent subsequently removed *in vacuo* to afford (^{Et}PDI)Ti(CD_2Ph-*d*$_5$)$_2$ as a black powder which was used without further purification.

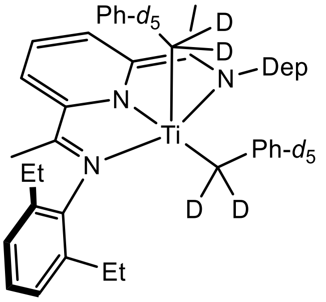

$C_{43}H_{35}D_{14}N_3Ti$, 669.84 g/mol

Yield: 30 mg (81%)

^{1}H NMR (400 MHz, 298 K, benzene-*d*$_6$): δ = 1.09 (t, ^3J = 7.5 Hz, 12H, CH_2CH_3), 1.36 (s, 6H, C(N)C*H*$_3$), 2.05−2.25 (brs, 4H, C*H*$_2$CH$_3$), 2.31−2.65 (brs, 4H, C*H*$_2$CH$_3$), 5.42 (t, ^3J = 7.7 Hz, 1H, *p*-C*H*$_{py}$), 6.07 (d, ^3J = 7.7 Hz, 2H, *m*-C*H*$_{py}$), 7.11−7.13 (brs, 6H, *m*/*p*-C*H*$_{Dep}$) ppm.

(^{Et}PDI)TiPh$_2$

In a 20 mL vial, a suspension of (^{Et}PDI)$TiCl_2$ (150 mg, 276 μmol, 1.00 equiv.) and PhLi (53 mg, 625 μmol, 2.26 equiv.) in benzene (5 mL) was stirred at ambient temperature

for 1 h. Subsequently, suspended solids were removed *via* centrifugation (2000 rpm, 5 min) followed by filtration of the solution over Celite. Removal of the solvent afforded (^{Et}PDI)TiPh$_2$ as a black powder.

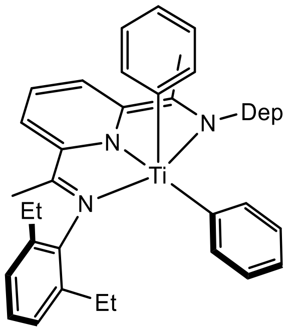

$C_{41}H_{45}N_3Ti$, 627.70 g/mol

Yield: 147 mg (85%)

^{1}H-NMR (500 MHz, 298 K, benzene-*d*$_6$) δ = 0.91 (t, ^3J = 7.9 Hz, 12H, CH$_2$C*H*$_3$), 1.33 (s, 6H, C(N)C*H*$_3$), 1.98–2.20 (m, 4H, C*H*$_2$CH$_3$), 2.25–2.39 (m, 4H, C*H*$_2$CH$_3$), 5.25 (t, ^3J = 7.5 Hz, 1H, *p*-C*H*$_{Pyr}$), 5.94 (d, ^3J = 7.6 Hz, 2H, *m*-C*H*$_{Pyr}$), 6.88 (d, ^3J = 7.6 Hz, 4H, *m*-C*H*$_{Dep}$), 6.96 (dd, ^3J = 8.1, 7.1 Hz, 2H, *p*-C*H*$_{Dep}$), 7.10 (t, ^3J = 7.3 Hz, 2H, *p*-C*H*$_{Ph}$), 7.19 (d, ^3J = 6.9 Hz, 4H, *m*-C*H*$_{Ph}$), 8.01 (d, ^3J = 6.7 Hz, 4H, *o*-C*H*$_{Ph}$) ppm.
^{13}C{^{1}H} NMR (500 MHz, 298 K, benzene-*d*$_6$) δ = 13.6 (s, CH$_2$*C*H$_3$), 14.8 (s, C(N)*C*H$_3$), 24.1 (s, *C*H$_2$CH$_3$), 119.7 (s, *p*-*C*$_{Pyr}$), 125.7 (s, *m*-*C*$_{Dep}$), 126.4 (s, *p*-*C*$_{Dep}$), 126.5 (s, *m*-*C*$_{Ph}$), 127.5 (s, *m*-*C*$_{Pyr}$), 129.0 (s, *p*-*C*$_{Ph}$), 131.5 (s, *o*-*C*$_{Ph}$), 135.5 (s, *o*-*C*$_{Dep}$), 142.0 (s, *o*-*C*$_{Pyr}$), 148.9 (s, i-*C*$_{Dep}$), 157.6 (s, *C*(N)CH$_3$), 199.7 (s, *i*-*C*$_{Ph}$) ppm.
Elemental analysis: (%) calc. for $C_{41}H_{45}N_3Ti$ [627.70 g mol^{-1}]: C, 78.45; H, 7.23; N, 6.69. **Found**: C, 76.97; H, 7.82; N, 6.16.

(^{Et}PDI)Ti(Ph-*d*$_5$)$_2$

In a 20 mL vial, a suspension of (^{Et}PDI)TiCl$_2$ (100 mg, 184 µmol, 1.00 equiv.) and Ph-*d*$_5$Li (36 mg, 404 µmol, 2.20 equiv.) in benzene (5 mL) was stirred at ambient temperature for 1 h. Subsequently, suspended solids were removed *via* filtration of the suspension over Celite. Removal of the solvent afforded (^{Et}PDI)Ti(Ph-*d*$_5$)$_2$ as a black powder.

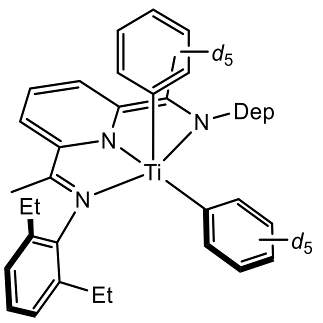

$C_{41}H_{35}D_{10}N_3Ti$, 637.76 g/mol

Yield: 47 mg (40%)

^{1}H-NMR (500 MHz, 298 K, benzene-*d*6) δ = 0.91 (t, ^3J = 7.9 Hz, 12H, CH_2CH_3), 1.33 (s, 6H, C(N)C*H3*), 1.98–2.20 (m, 4H, CH_2CH_3), 2.25–2.39 (m, 4H, CH_2CH_3), 5.25 (t, ^3J = 7.5 Hz, 1H, *p*-CH_{Pyr}), 5.94 (d, ^3J = 7.6 Hz, 2H, *m*-CH_{Pyr}), 6.88 (d, ^3J = 7.6, 4H, *m*-CH_{Dep}), 6.96 (m, 2H, *p*-CH_{Dep}) ppm.

(^{Et}PDI)Ti(*p*-Tol)$_2$

In a 20 mL vial, a suspension of (^{Et}PDI)$TiCl_2$ (480 mg, 882 µmol, 1.00 equiv.) and *p*-TolLi (195 mg, 1.99 mmol, 2.25 equiv.) in benzene (10 mL) was stirred at ambient temperature for 5 h. Suspended solids were then removed *via* centrifugation (2000 rpm, 5 min) followed by filtration over Celite. Removal of the solvent *in vacuo* afforded (^{Et}PDI)Ti(*p*-Tol)$_2$ as a black powder. Crystals suitable for SCXRD were obtained by cooling a saturated *n*-hexane solution of (^{Et}PDI)Ti(*p*-Tol)$_2$ from ambient temperature to −35 °C.

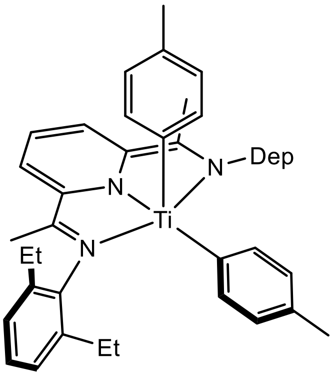

$C_{43}H_{49}N_3Ti$, 655.75 g/mol

Yield: 494 mg (85%)

^{1}H NMR (500 MHz, benzene-*d*6) δ = 0.94 (t, ^3J = 7.5 Hz, 12H, CH_2CH_3), 1.34 (s, 6H, C(N)CH_3), 2.08 (ps. sext., ^3J = 7.6 Hz, 4H, CH_2CH_3), 2.09 (s, 6H, *p*-*T*ol-CH_3), 2.93 (ps.

sext., ^3J = 7.6 Hz, 4H, CH_2CH$_3$), 5.28 (t, ^3J = 7.7 Hz, 1H, *p*-CH_{py}), 5.99 (d, ^3J = 7.7 Hz, 2H, *m*-CH$_{py}$), 6.89−6.92 (m, 4H, *m*-CH_{Dep}), 6.98−7.01 (m, 6H, *p*-CH_{Dep}/*m*-C$H_{p\text{-Tol}}$), 7.93−7.96 (m, 4H, *o*-C$H_{p\text{-Tol}}$) ppm.

^{13}C{^{1}H} NMR (126 MHz, benzene-d_6) δ = 13.63 (s, CH$_2$*C*H$_3$), 14.88 (s, C(N)*C*H$_3$), 21.73 (s, *p*-Tol-*C*H$_3$), 24.08 (s, *C*H$_2$CH$_3$), 119.24 (s, *p*-*C*H$_{py}$), 125.62(s, *m*-*C*H$_{Dep}$), 126.17 (s, *p*-*C*H$_{Dep}$), 127.03 (s, *m*-*C*H$_{p\text{-Tol}}$), 127.56 (s, *m*-*C*H$_{py}$), 131.74 (s, *o*-*C*H$_{p\text{-Tol}}$), 135.53 (s, *o*-*C*$_{Dep}$), 138.21 (s, *p*-*C*$_{p\text{-Tol}}$), 142.12 (s, *o*-*C*$_{py}$), 149.34 (s, *i*-*C*$_{Dep}$), 157.23 (s, *C*(N)CH$_3$), 197.82 (s, *i*-*C*$_{p\text{-Tol}}$) ppm.

Elemental analysis: (%) calc. for $C_{43}H_{49}N_3Ti$ [655.75 g mol^{-1}]: C, 78.76; H, 7.53; N, 6.41. **Found**: C, 76.97; H, 7.82; N, 6.16.

(^{Et}PDI)TiI$_2$

In a 50 mL Schlenk tube, (^{Et}PDI)TiCl$_2$ (100 mg, 184 µmol, 1.00 equiv.) was suspended in benzene (4 mL). TMSI (110 mg, 78.6 µL, 552 µmol, 3.00 equiv.) was added *via* syringe and the reaction mixture was heated to 80 °C for 21 h, which resulted in a color change from dark green to rust red. Subsequently, volatiles were removed *in vacuo*, and the residue resuspended in benzene (4 mL). A second portion of TMSI (55 mg, 38.3 µL, 276 mmol, 1.50 equiv.) was added and the mixture was heated to 80 °C for 19 h. Volatiles were again removed *in vacuo* and the residual solid washed with *n*-pentane (2×4 mL). Subsequent drying *in vacuo* afforded (^{Et}PDI)TiI$_2$ as a dark brown powder. Crystals suitable for SCXRD were obtained by slow diffusion of *n*-pentane into a saturated toluene solution of (^{Et}PDI)TiI$_2$ at ambient temperature from an OIRE reaction (see Chapter 3).

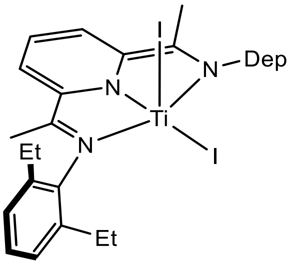

$C_{29}H_{35}I_2N_3Ti$, 727.30 g/mol

Yield: 113 mg (85%)

^{1}H NMR (500 MHz, benzene-d_6) δ = 1.17 (t, ^3J = 7.5 Hz, 12H, CH$_2$CH_3), 1.39 (s, 6H, C(N)CH_3), 2.31 (ps. sext., ^3J = 7.5 Hz, 4H, CH_2CH$_3$), 2.91 (ps. sext., ^3J = 7.5 Hz, 4H,

CH_2CH_3), 5.26 (t, ^3J = 7.7 Hz, 2H, *p*-CH_{py}), 6.01 (d, ^3J = 7.7 Hz, 2H, *m*-CH_{py}), 7.15−7.17 (m, 4H, *m*-CH_{Dep}), (dd, ^3J = 8.6, ^3J = 6.6 Hz, 2H, *p*-CH_{Dep}) ppm.

^{13}C{^{1}H} NMR (126 MHz, benzene-d_6) δ = 14.09 (s, CH_2CH_3), 16.45 (s, C(N)CH_3), 25.22 (s, CH_2CH_3), 126.22 (s, *p*-CH_{py}), 126.68 (s, *m*-CH_{py}), 126.74 (s, *m*-CH_{Dep}), 128.16 (s, *p*-CH_{Dep}), 134.68 (s, *o*-C_{Dep}), 143.84 (s, *o*-C_{py}), 150.75 (s, *i*-C_{Dep}), 170.10 (s, *C*(N)CH_3) ppm.

Elemental analysis (%) calc. for $C_{29}H_{35}I_2Ti$ [727.30 g mol^{-1}]: C, 47.89; H, 4.85; N, 5.78. **Found**: C, 48.30; H, 5.12; N, 5.73.

3. Synthetic procedures for reactivity & mechanistic studies

General procedure for OIRE reactions

In a J. Young NMR tube, approximately 10 mg (1.0 equiv.) of (^{Et}PDI)TiR_2 (R = CH_2Ph, Ph, *p*-Tol, Me) or (^{iPr}PDA)TiR_2 (R = CH_2Ph, Ph)were weighed out and dissolved in benzene-d_6 (0.4 mL). HMDSO (10.0 µL) was then added *via* Hamilton syringe and the exact amount of complex determined *via* ^{1}H NMR spectroscopy. The complex resonance of the CH_2CH_3 groups around 0.9 ppm (for (^{Et}PDI)TiR_2) and those for the $CH(CH_3)_2$ groups at 0.89 ppm (for (^{iPr}PDA)TiR_2) were used for integration against the HMDSO signal. Subsequently, 3 equivalents of the oxidant were added to the reaction mixture. The sample was sonicated for 3 min and subsequently mixed at ambient temperature for 1 h. Any suspended solids were then filtered off using a filter pipette, and the resulting solution investigated *via* ^{1}H NMR spectroscopy. Amounts of toluene and bitolyl were calculated by integration of the ArCH_3 signals. For biphenyl, the *o*-CH_{Ph} resonance at 7.45 ppm was used. Benzene was not quantified due to overlap with the residual solvent signal of benzene-d_6. Organic benzyl compounds resulting from OIRE with (^{Et}PDI)Ti(CH_2Ph)$_2$ were quantified *via* integration of their respective Ph$CH_{2/3}$ resonances (2.11 (PhCH_3), 2.73 ((PhCH_2)$_2$), 2.77−2.79 ([C_7H_7]CH_2Ph), 3.89 (PhCH_2I), 4.90 (PhCH_2−TEMPO) ppm). Further reaction details are listed in Table S1.

Table S1. Summary of reaction details of the conducted oxidation reactions.

Complex	Oxidant	equiv.	R−R yield [%]	R−H yield [%]
(^{Et}PDI)Ti(*p*-Tol)$_2$	[Fc][BF_4]	3.0	74	3
(^{Et}PDI)Ti(*p*-Tol)$_2$	[Fc][BAr^F_4]	3.0	48	37
(^{Et}PDI)Ti(*p*-Tol)$_2$	[C_7H_7][BF_4]	3.0	69	4
(^{Et}PDI)Ti(*p*-Tol)$_2$	[C_7H_7][BAr^F_4]	3.0	35	33
(^{Et}PDI)Ti(*p*-Tol)$_2$	AgOTf	3.0	98	4
(^{Et}PDI)Ti(*p*-Tol)$_2$	I_2	1.5	98	3
(^{Et}PDI)Ti(*p*-Tol)$_2$	PhICl_2	1.5	92	9
(^{Et}PDI)Ti(CH_2Ph)$_2$	[Fc][BF_4]	3.0	< 1	1
(^{Et}PDI)Ti(CH_2Ph)$_2$	[Fc][BAr^F_4]	3.0	< 1	19
(^{Et}PDI)Ti(CH_2Ph)$_2$	[C_7H_7][BF_4]	3.0	< 1	< 1
(^{Et}PDI)Ti(CH_2Ph)$_2$	[C_7H_7][BAr^F_4]	3.0	1	9
(^{Et}PDI)Ti(CH_2Ph)$_2$	AgOTf	3.0	30	10
(^{Et}PDI)Ti(CH_2Ph)$_2$	I_2	1.5	16	2
(^{Et}PDI)Ti(CH_2Ph)$_2$	PhICl_2	1.5	16	6
(^{iPr}PDA)Ti(Ph)$_2$	AgOTf	3.0	18	n. d.
(^{iPr}PDA)Ti(CH_2Ph)$_2$	AgOTf	1.0	75	5

In some cases, the organometallic species of the reaction was identified by crystallization and analysis of single crystals by SCXRD.

(^{iPr}PDA)Ti(CH_2Ph)(OTf): Crystals were obtained from a saturated solution of the compound in THF at ambient temperature.

(^{iPr}PDA)Ti(Ph)(OTf): Crystals were obtained by slow diffusion of *n*-pentane into a saturated THF solution of the compound at ambient temperature.

(^{Et}PDI^{Bn})TiI_2: Crystals were obtained from toluene at ambient temperature.

(^{Et}PDI)TiI_2: Crystals were obtained by slow diffusion of *n*-pentane into a saturated toluene solution of the compound at ambient temperature.

(^{Et}PDI)Ti$(OTf)_2$: Crystals were obtained by cooling a saturated solution of the compound in *n*-pentane from ambient temperature to −35 °C. Attempts were made to isolate this compound from the reaction of (^{Et}PDI)$TiCl_2$ with two equivalents of AgOTf. However, these reactions led to mixtures of (^{Et}PDI)Ti$(OTf)_2$ and (^{Et}PDI)Ti$(OTf)_3$ (overoxidation), see Figure **S47**.

General procedure for cross-over reactions

In a J. Young NMR Tube, approximately 10 mg of (^{Et}PDI)TiPh_2 (0.5 equiv.) and 10 mg of (^{Et}PDI)Ti(*p*-Tol)$_2$ (0.5 equiv.) were dissolved in benzene-d_6 (0.4 mL). HMDSO (10.0 µL) was then added via Hamilton syringe and the exact amount of each complex determined *via* ^{1}H NMR spectroscopy. Subsequently, the oxidant was added to the solution. The reaction mixture was mixed at ambient temperature for one hour and subsequently analysed *via* ^{1}H NMR spectroscopy. The solution was then exposed to air, filtered and transferred to a GC vial for further analysis by HRMS and GCMS. More reaction details are listed in Table S2.

Experiments with PDA complexes, i.e. (^{iPr}PDA)Ti$(CH_2Ph)_2$ and (^{iPr}PDA)Ti$(CD_2Ph\text{-}d_5)_2$ or (^{iPr}PDA)TiPh_2 and (^{iPr}PDA)Ti(Ph-d_5)$_2$ were performed analogously, but without prior quantification of the complex in solution due to limited solubility in benzene.

Table S2. Details on cross-over reactions of TiR_2 and TiR'_2 and findings. Equivalents of oxidant are given per [Ti].

Ligand	R	R'	Oxidant	Equiv.	Reaction time [h]	R_2 / R_2'	R-R'
^{Et}PDI	*p*-Tol	Ph	[Fc][BAr$^F_4$]	1.2	2.5	Major	Not detected
^{Et}PDI	*p*-Tol	Ph	[C_7H_7][BAr$^F_4$]	4.0	4 h	Major	Not detected
^{Et}PDI	*p*-Tol	Ph	I_2	1.5	1.5	Major	Not detected
^{Et}PDI	*p*-Tol	Ph	$PhICl_2$	1.5	1 h	Major	Traces
^{Et}PDI	Ph	Ph-*d*$_5$	[C_7H_7][BAr$^F_4$]	3.0	1 h	Major	Not detected
^{iPr}PDA	CH_2Ph	CD_2Ph-*d*$_5$	AgOTf	3.0	1 h	Minor	Major
^{iPr}PDA	Ph	Ph-*d*$_5$	AgOTf	3.0	1 h	Minor	Major

General procedure for trapping reactions

In a J. Young NMR Tube, approximately 10 mg of (^{Et}PDI)Ti(CH_2Ph)$_2$ or 30 mg of (^{iPr}PDA)Ti(CH_2Ph)$_2$ were weighed out and dissolved in benzene-*d*$_6$ (0.4 mL). HMDSO (10.0 µL) was then added via Hamilton syringe and the exact amount of complex determined *via* ^{1}H NMR spectroscopy (only for PDI complexes). 5.0 equivalents of TEMPO (PDI and PDA) or phenothiazine (only PDA) or 2.5 equivalents of Gomberg's dimer (only PDA) were then added followed by AgOTf (1.0 equiv. for PDA, 3.0 equiv. for PDI). The reaction mixture was sonicated for 3 min and subsequently mixed at ambient temperature for 1 h. Suspended Ag^0 was filtered off, and the reaction mixture analysed *via* ^{1}H NMR spectroscopy. The solution was then exposed to air, filtered and transferred to a 2 mL vial for further analysis by GCMS.

General procedure for spin trapping reactions

In a J. Young NMR Tube, approximately 15 mg of (^{iPr}PDA)Ti(CH_2Ph)$_2$ (1.0 equiv.) and DMPO or PBN (8.5 equiv.) were weighed out and suspended in benzene (0.4 mL). AgOTf (1.0 equiv.) was then added and the sample mixed for 1 h at ambient temperature. EPR measurements were then conducted at ambient temperature to confirm the formation of benzyl-DMPO or benzyl-PBN, respectively.

General procedure for synthetic cycles with (^{Et}PDI)TiAr$_2$

In a J. Young NMR Tube, 10 mg of (^{Et}PDI)TiCl$_2$ (1.0 equiv.) were suspended in benzene-*d*$_6$ (0.4 mL) and 2.0 equivalents of a titrated solution of PhLi (1.13 M in *n*-Bu_2O) or *p*-TolLi (941 mM in Et_2O) added *via* syringe. The resulting dark green solution was mixed at ambient temperature for 5 min and the exact amount of resulting (^{Et}PDI)TiAr$_2$ (Ar = Ph, *p*-Tol) then determined by quantification with HMDSO

(10.0 µL) *via* ^{1}H NMR. 1.0 equivalent of a titrated solution of I_2 in benzene-d_6 (465 mM) was then added, resulting in immediate precipitation of a dark solid and a colour change to rust red. The following addition of 2.0 equivalents of PhLi or *p*-TolLi solution caused a colour change back to dark green. The cycles were then continued as described and the yield of biaryl determined after each addition of ArLi (Ar = Ph, *p*-Tol). Yields were calculated relative to the initial amount of (^{Et}PDI)TiAr$_2$ (Ar = Ph, *p*-Tol).

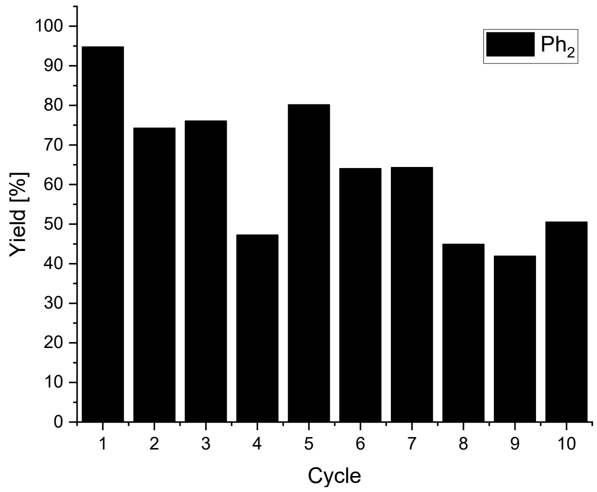

Figure S1. Distribution of aryl-based organic products observed in the synthetic cycle of the coupling reaction of PhLi with (^{Et}PDI)TiPh$_2$.

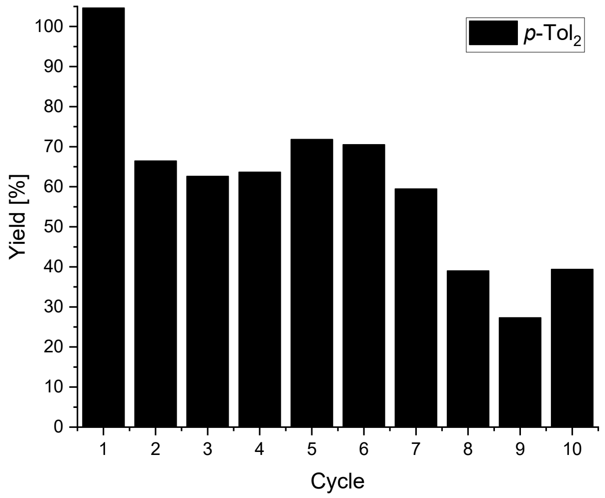

Figure S2. Distribution of aryl-based organic products observed in the synthetic cycle of the coupling reaction of PhLi with (^{Et}PDI)TiPh$_2$.

Procedure for the electrochemical oxidation of (^{Et}PDI)Ti(*p*-Tol)$_2$

In an argon-filled glovebox, a 10 mL reaction vessel with a stir bar was charged with 20 mg of (^{Et}PDI)Ti(*p*-Tol)$_2$, [*n*-Bu$_4$N][BAr^F$_4$] (50 mM) and THF (6 mL). HMDSO (20.0 µL) was added and the exact amount of (^{Et}PDI)Ti(*p*-Tol)$_2$ determined *via* ^{1}H NMR

spectroscopy. Electrodes were then inserted to the cell and the reaction vessel sealed with a rubber septum. The electrodes were then connected to the power supply and a constant current of 3.5 mA applied while stirring at ambient temperature. The reaction progress was monitored *via* ^{1}H NMR after 1 h. A conversion of around 90% was observed along with 65% of the biaryl and 30% of toluene. The formation of *p*-Tol_2 was further confirmed by GCMS measurements.

Procedure for EPR-spectroscopic

A J. Young NMR tube was charged with 15 mg of (^{Et}PDI)$TiPh_2$ and [Fc][BAr^F_4] (0.8 equiv.). After cooling the solids to −70 °C, toluene was added *via* condensation under static vacuum and the suspension was mixed for 2 h. The dark green sample was then transferred to a pre-cooled EPR spectrometer and monitored while warming to ambient temperature. After the measurement, a brown colour associated with ferrocene was observed, indicating successful oxidation. No evidence for an intermediate Ti(III)-species was found.

4. NMR spectra of isolated compounds

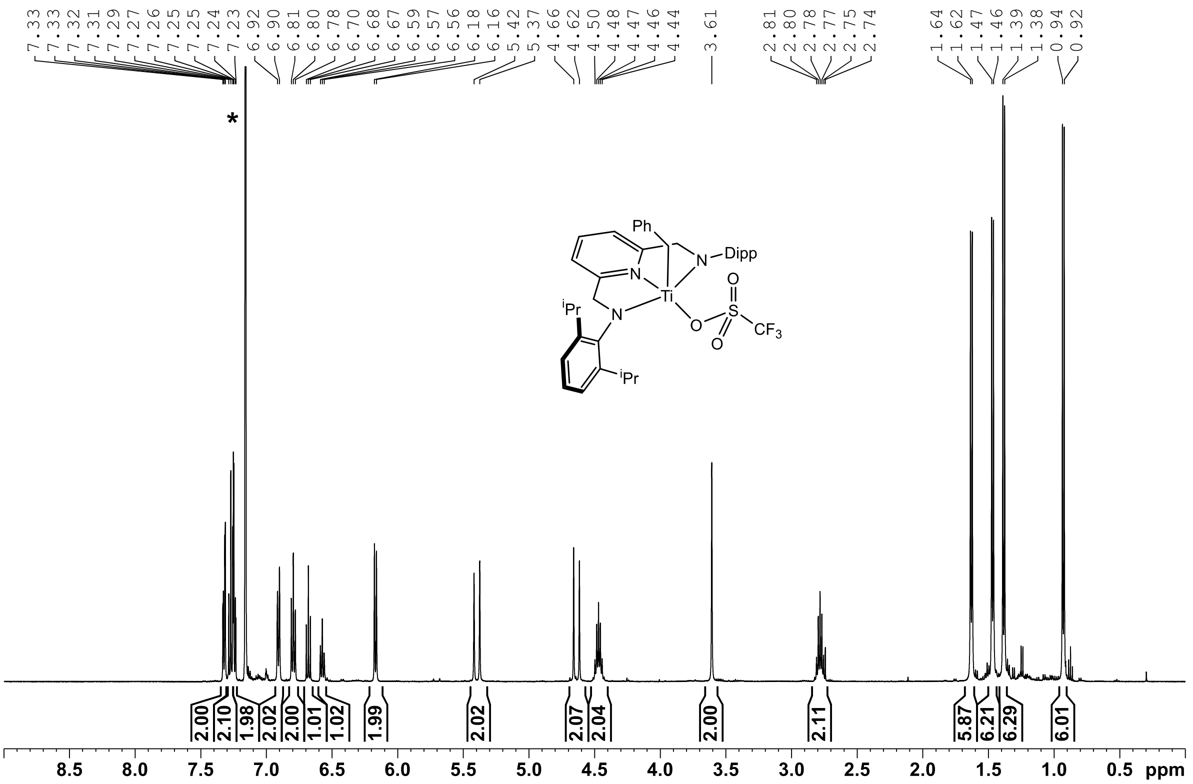

Figure S3. ^{1}H NMR spectrum of (^{iPr}PDA)Ti(CH_2Ph)(OTf) (500 MHz, 298 K, benzene-d_6). Residual signal of deuterated solvent is marked with an asterisk (*).

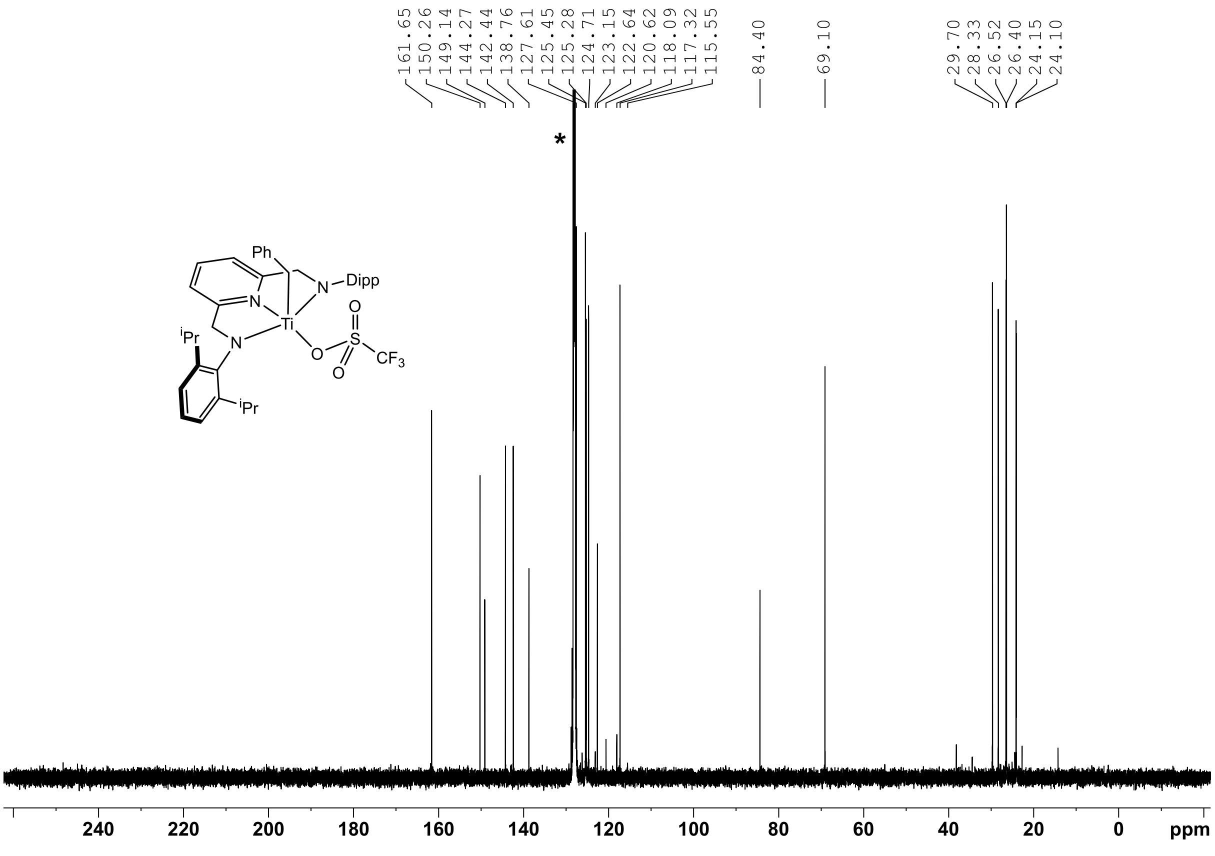

Figure S4. ^{13}C {^{1}H} NMR spectrum of (^{iPr}PDA)Ti(CH_2Ph)(OTf) (126 MHz, 298 K, benzene-d_6). Residual signal of deuterated solvent is marked with an asterisk (*).

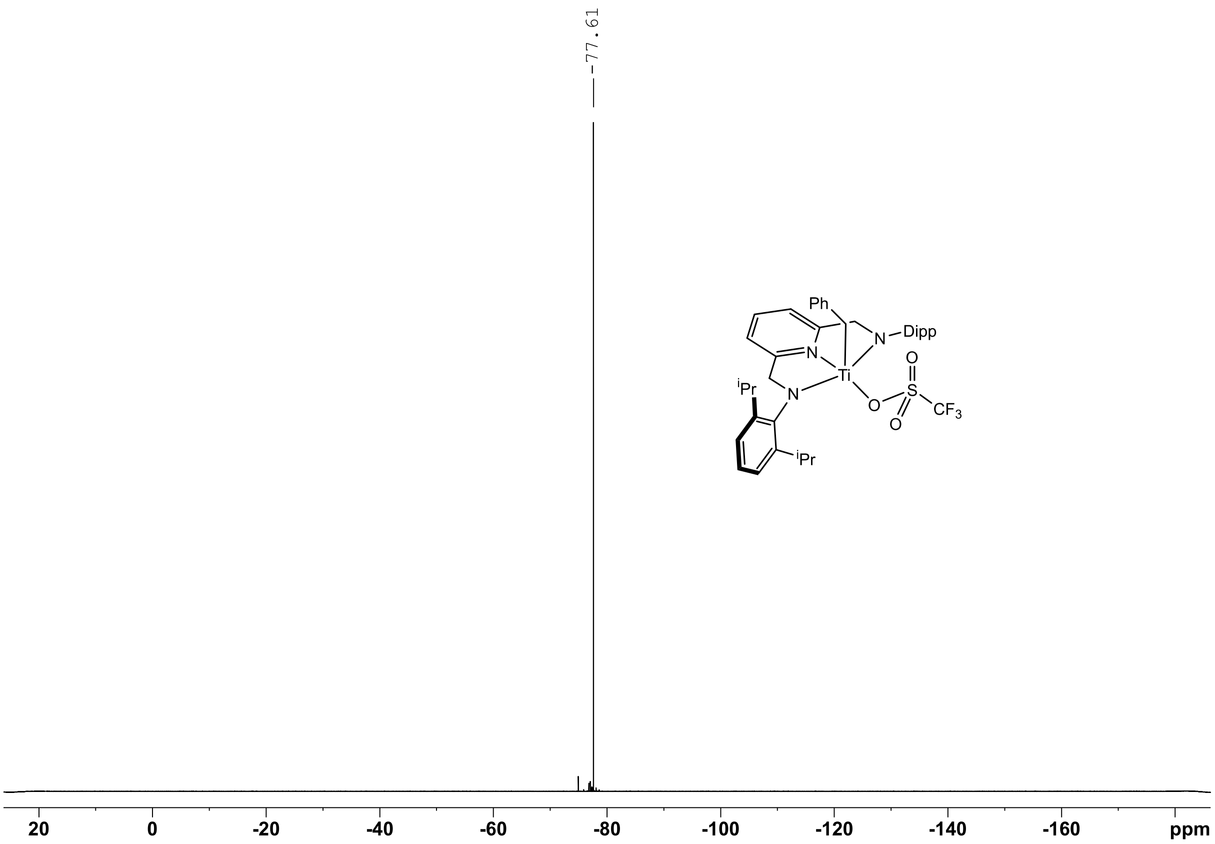

Figure S5. ^{19}F NMR spectrum of (^{iPr}PDA)Ti(CH_2Ph)(OTf) (471 MHz, 298 K, benzene-d_6).

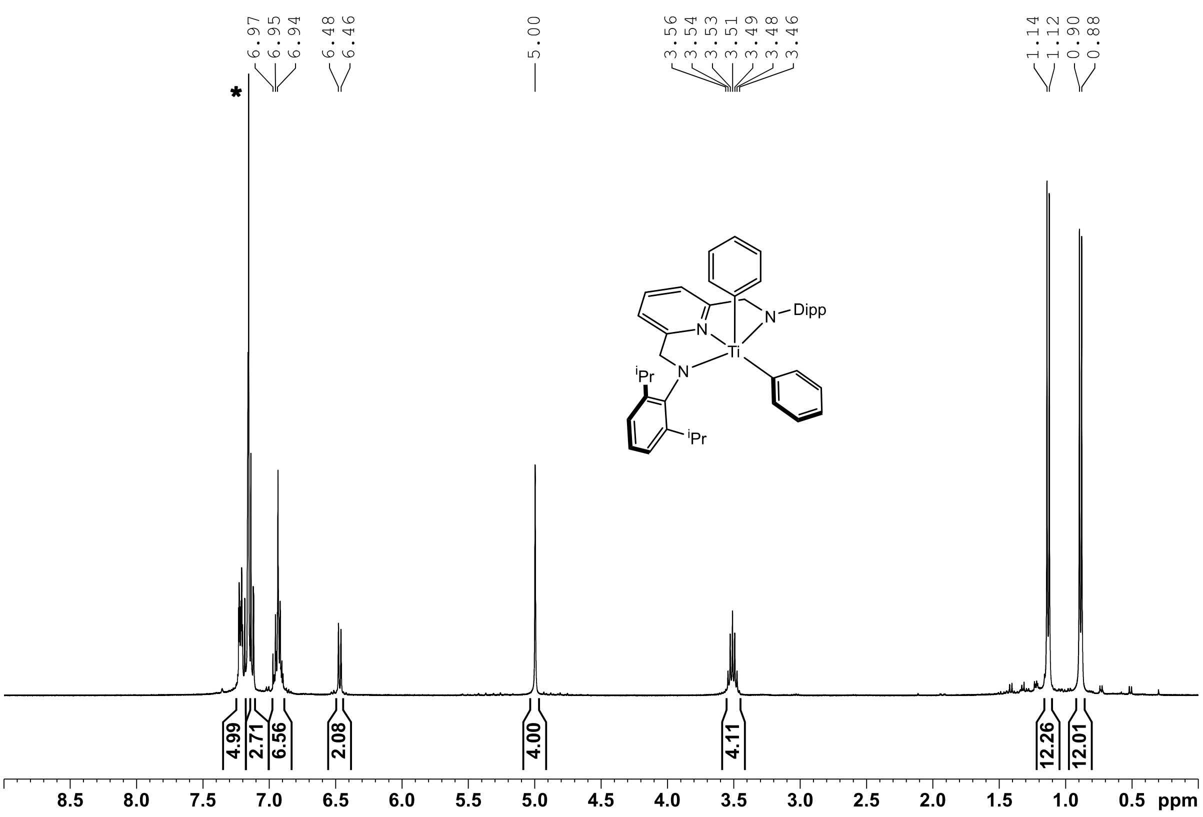

Figure S6. ^{1}H NMR spectrum of (^{iPr}PDA)$TiPh_2$ (400 MHz, 298 K, benzene-d_6). Residual signal of deuterated solvent is marked with an asterisk (*).

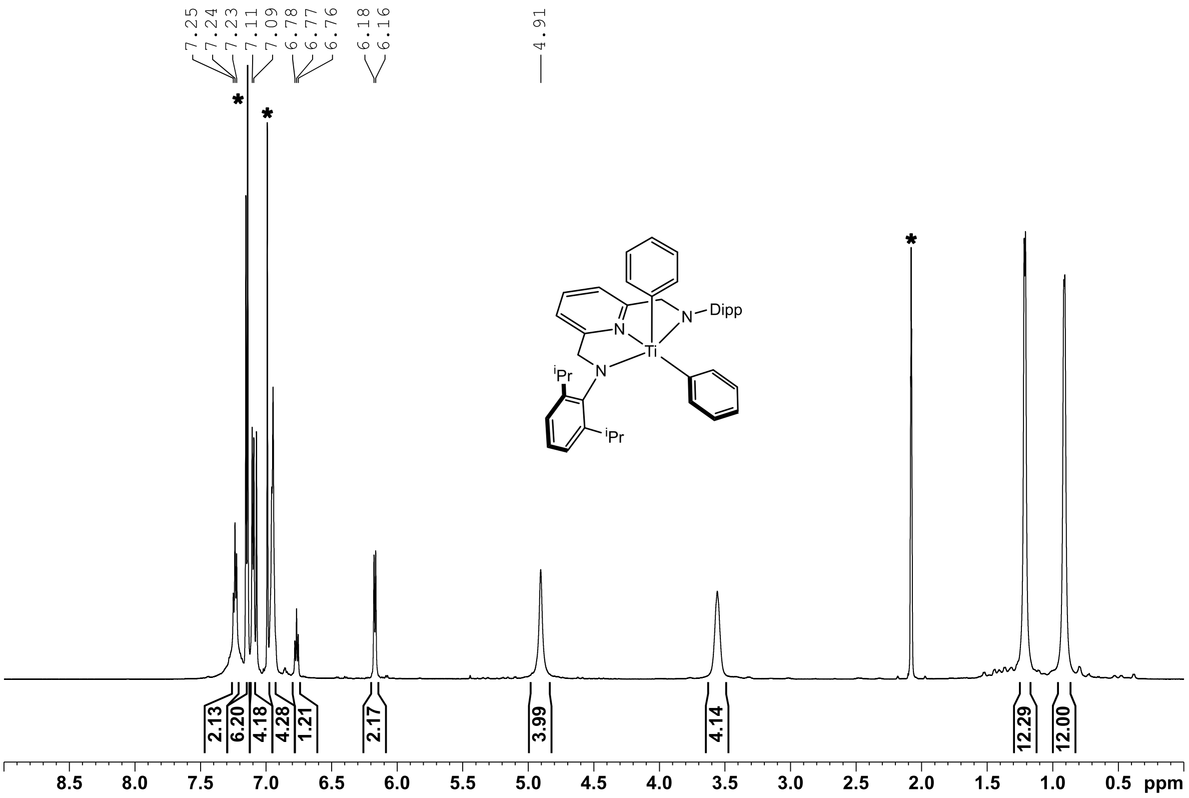

Figure S7. ^{1}H NMR spectrum of (^{iPr}PDA)TiPh$_2$ (600 MHz, 193 K, toluene-d_8). Residual signal of deuterated solvent is marked with an asterisk (*).

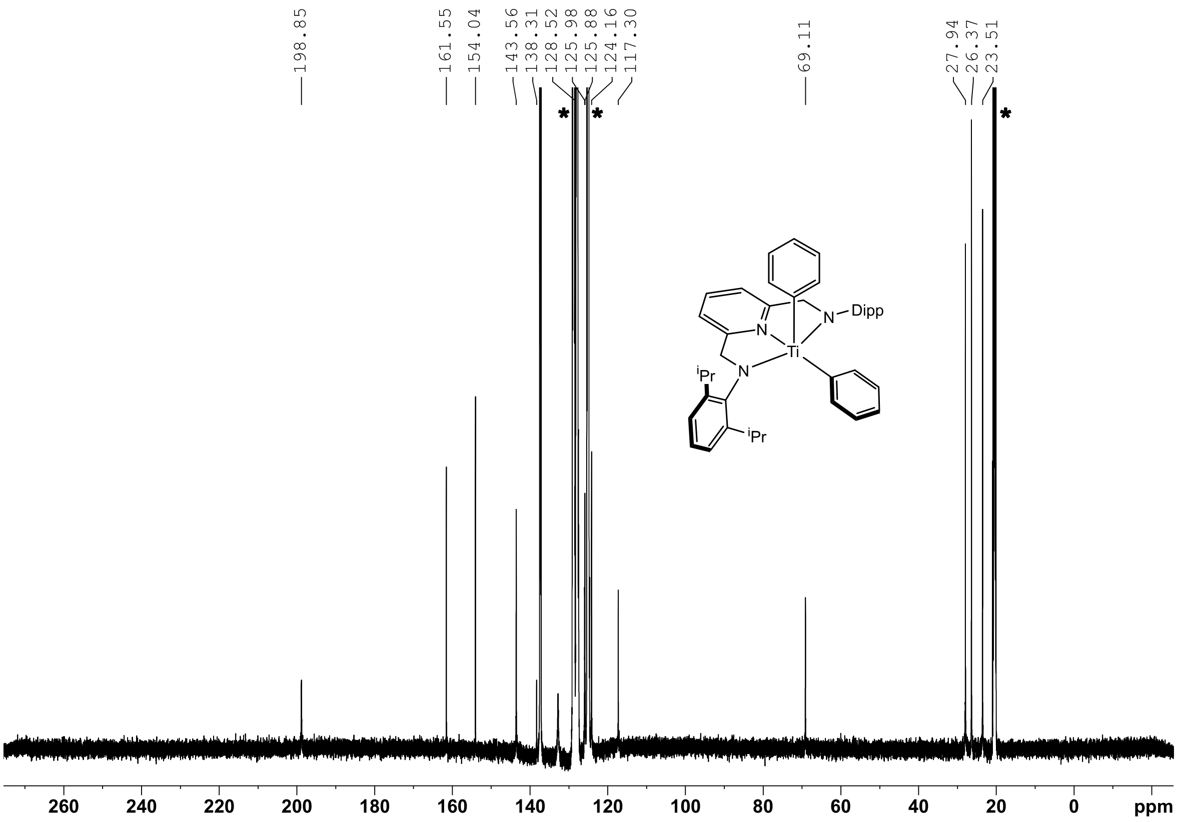

Figure S8. ^{13}C {^{1}H} NMR spectrum of (^{iPr}PDA)TiPh$_2$ (151 MHz, 193 K, toluene-d_8). Residual signal of deuterated solvent is marked with an asterisk (*).

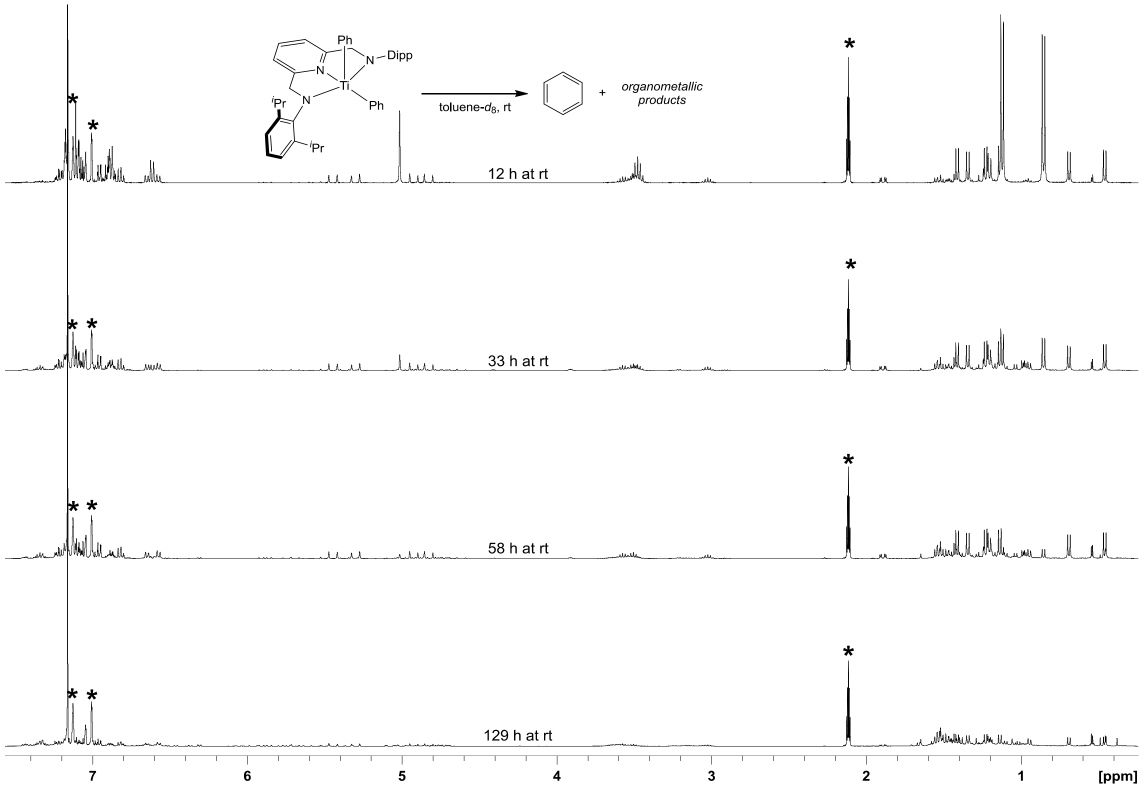

Figure S9. ^{1}H NMR spectra (400 MHz, 298 K, toluene-d_8) of the thermal decomposition of (^{iPr}PDA)$TiPh_2$ at ambient temperature. Following signals are marked: Residual solvent signal is marked with an asterisk (*).

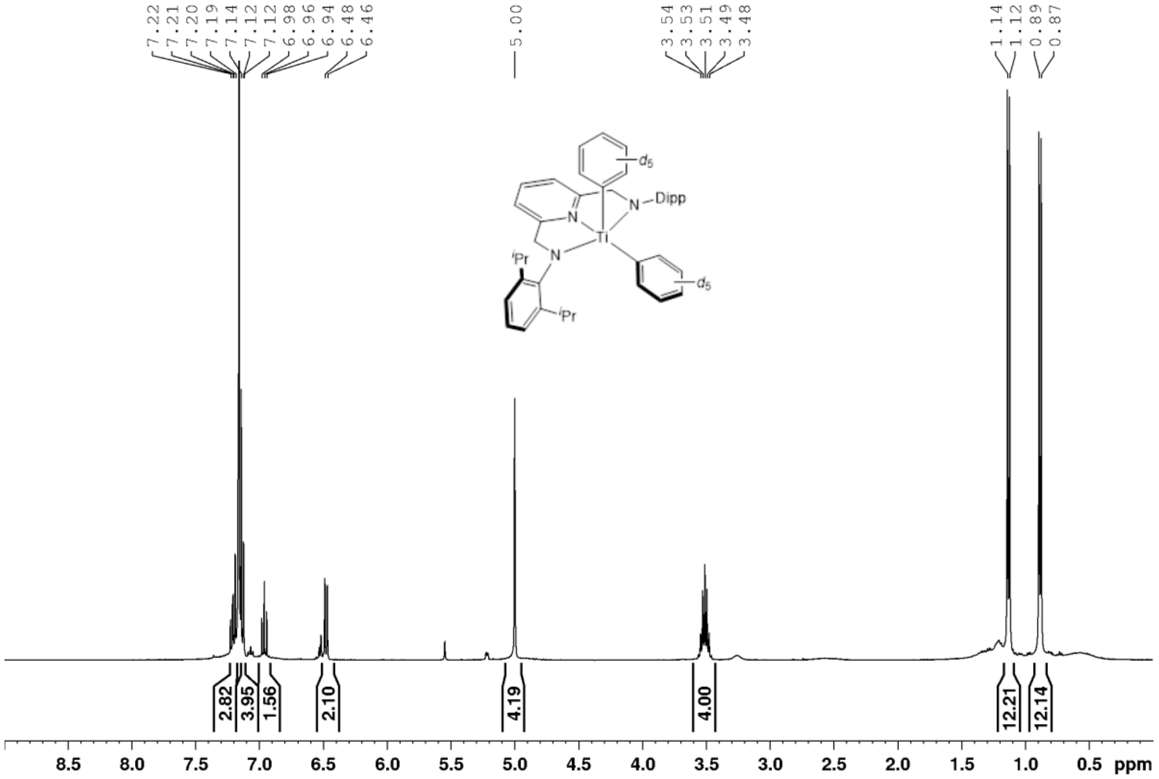

Figure S10. ^{1}H NMR spectrum of (^{iPr}PDA)Ti(Ph-d_5)$_2$ (400 MHz, 298 K, benzene-d_6). The residual solvent signal is marked with an asterisk (*). Due to overlap with the residual solvent signal, the integral between δ = 7.18 and 7.23 ppm is larger than expected.

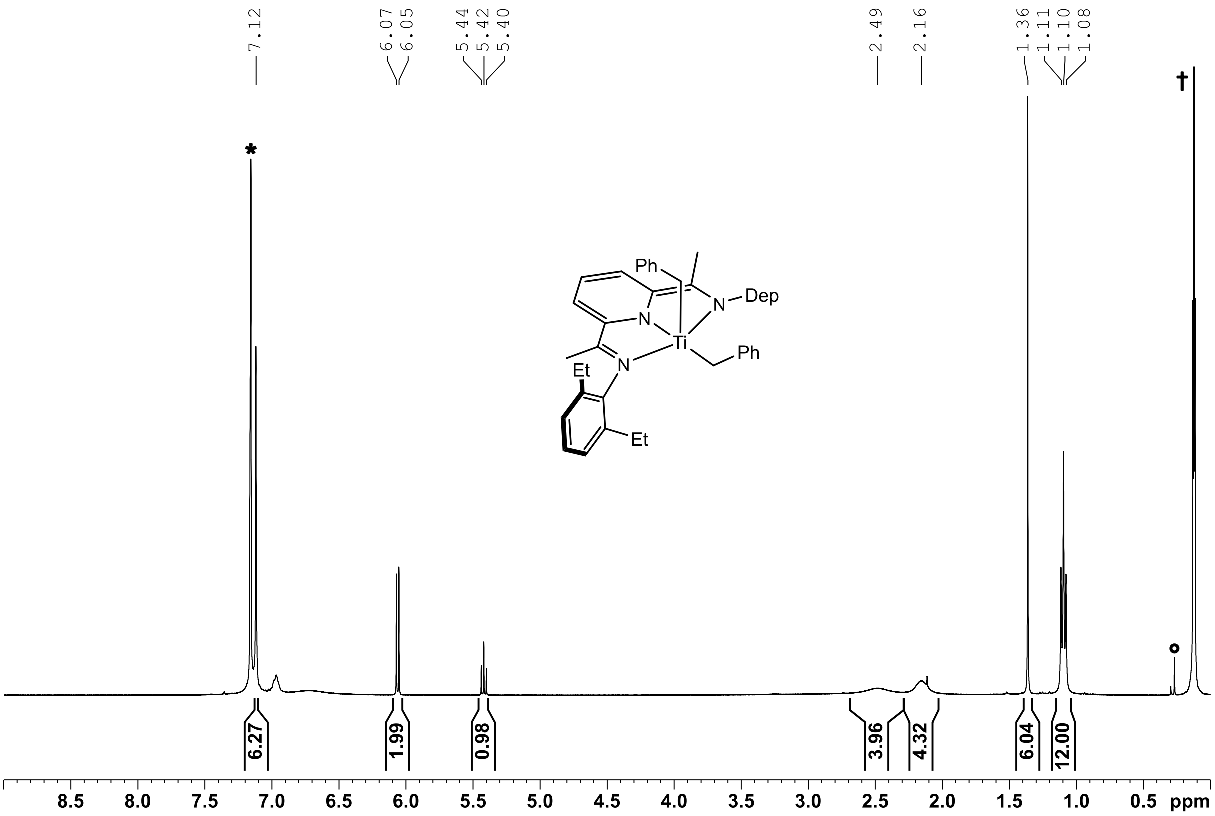

Figure S11. ^{1}H NMR spectrum of (^{Et}PDI)Ti(CH_2Ph)$_2$ (400 MHz, 298 K, benzene-d_6). Following signals are marked: Residual solvent signal (*), HMDSO from quantification (†), silicon grease (°).

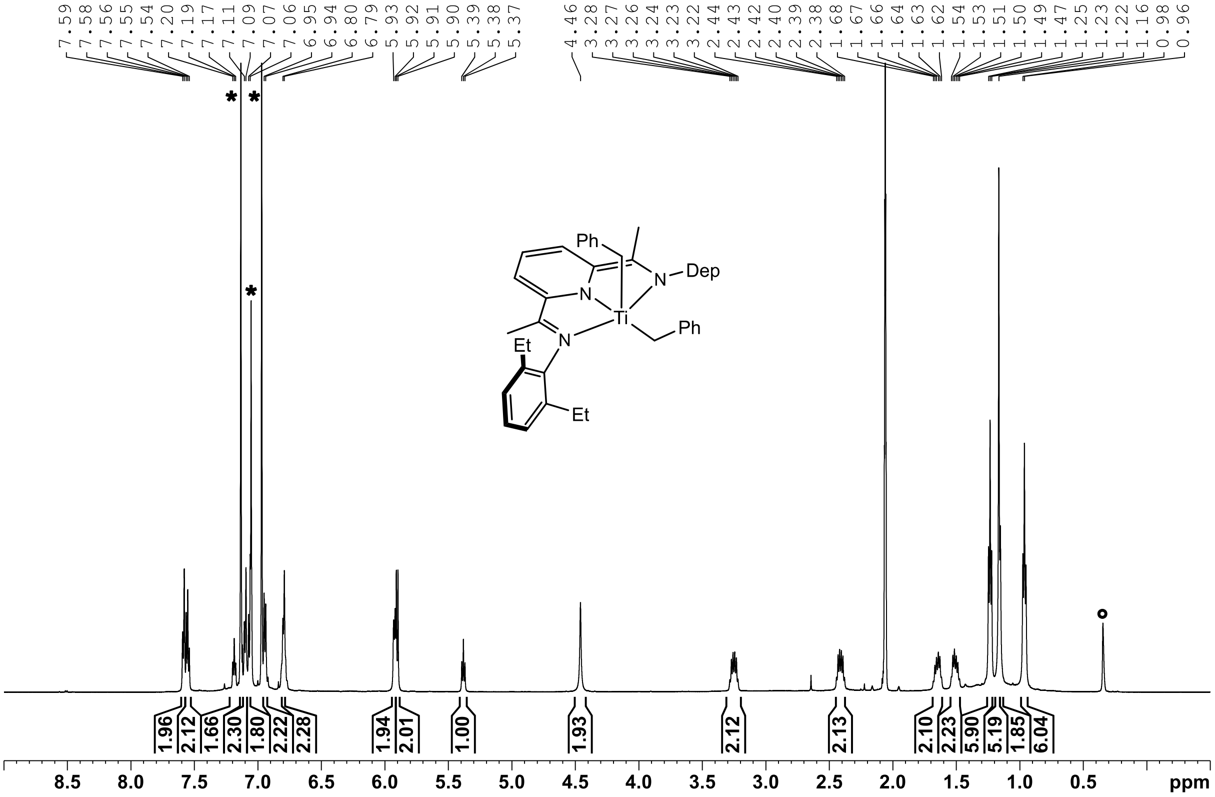

Figure S12. ^{1}H NMR spectrum of (^{Et}PDI)Ti(CH_2Ph)$_2$ (600 MHz, 193 K, toluene-d_8). Following signals are marked: Residual solvent signal (*), silicon grease (°).

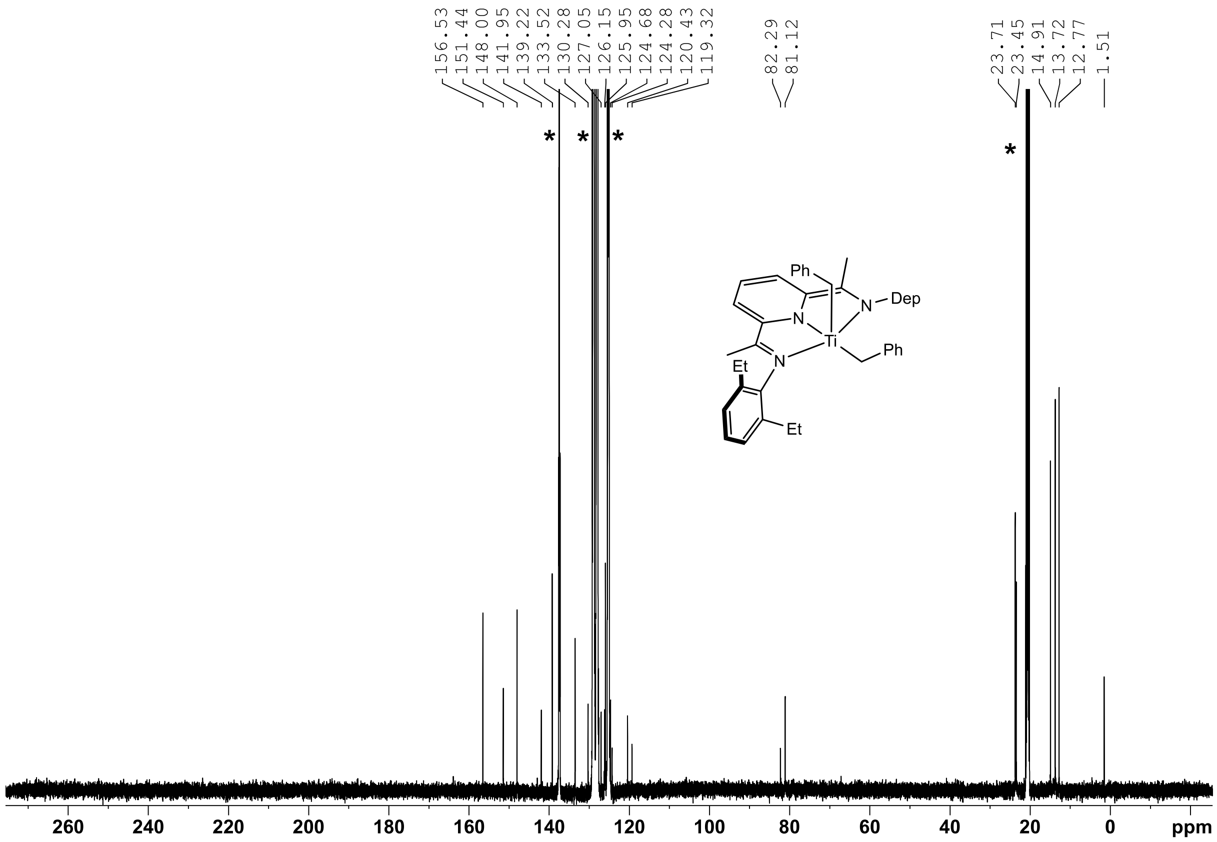

Figure S13. ^{13}C {^1H} NMR spectrum of (^{Et}PDI)Ti(CH_2Ph)$_2$ (151 MHz, 193 K, toluene-d_8). Residual signal of deuterated solvent is marked with an asterisk (*).

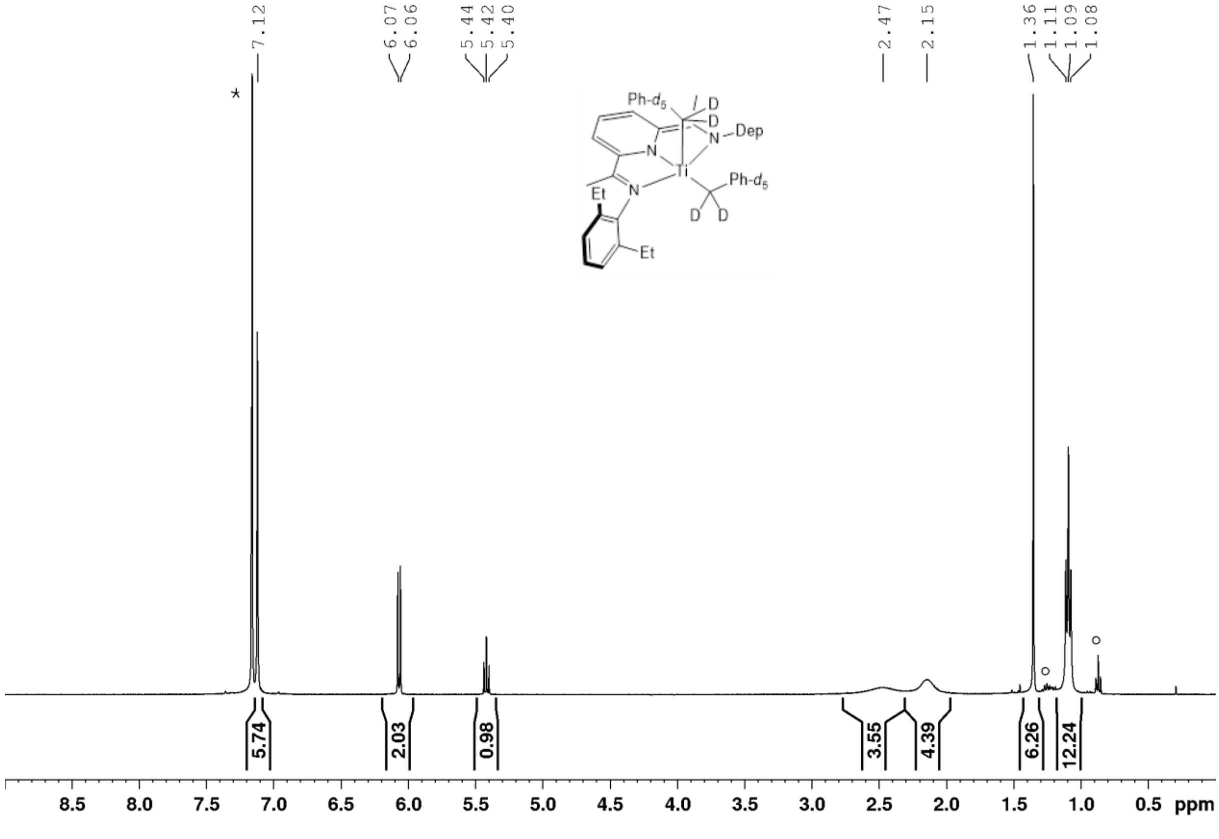

Figure S14. ^1H NMR spectrum of (^{Et}PDI)Ti(CD_2Ph-d_5)$_2$ (500 MHz, 298 K, benzene-d_6). Residual signal of deuterated solvent (*); *n*-pentane (°).

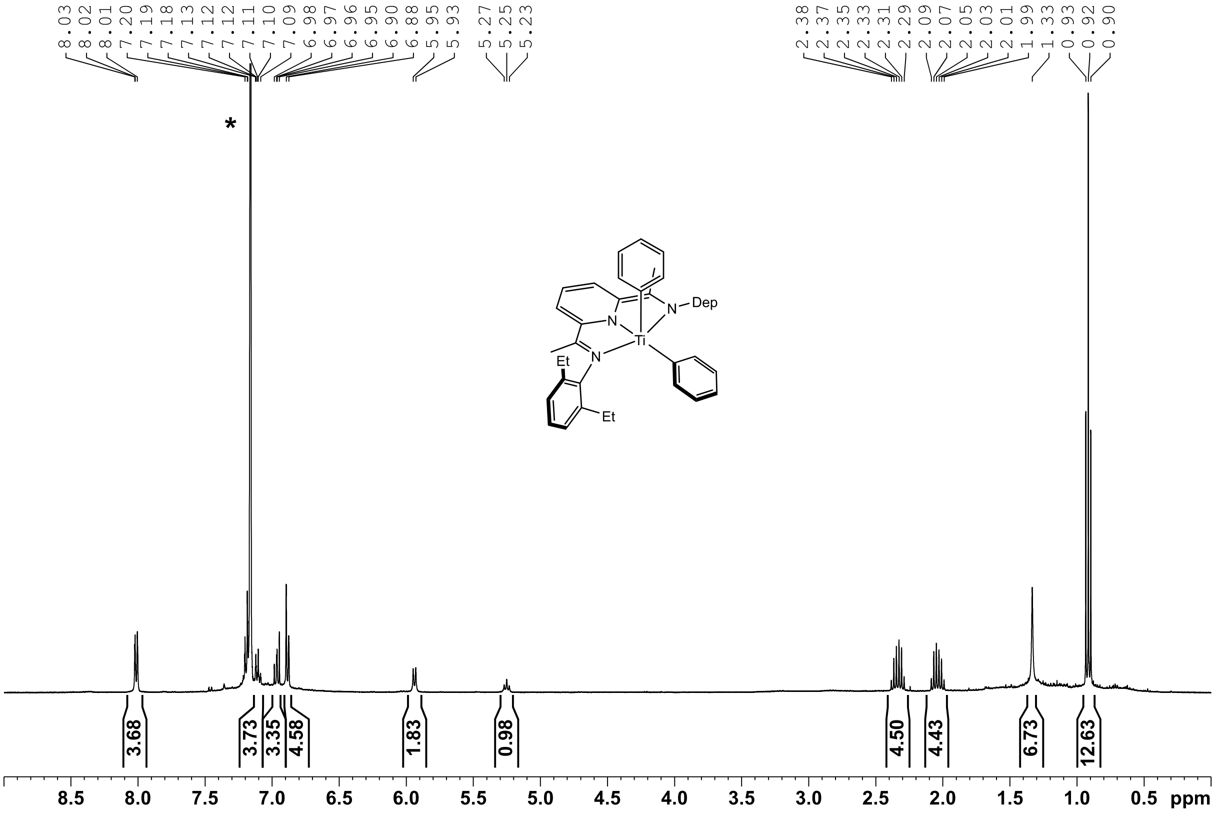

Figure S15. ^{1}H NMR spectrum of (^{Et}PDI)TiPh$_2$ (600 MHz, 298 K, toluene-d_8). Residual signal of deuterated solvent is marked with an asterisk (*).

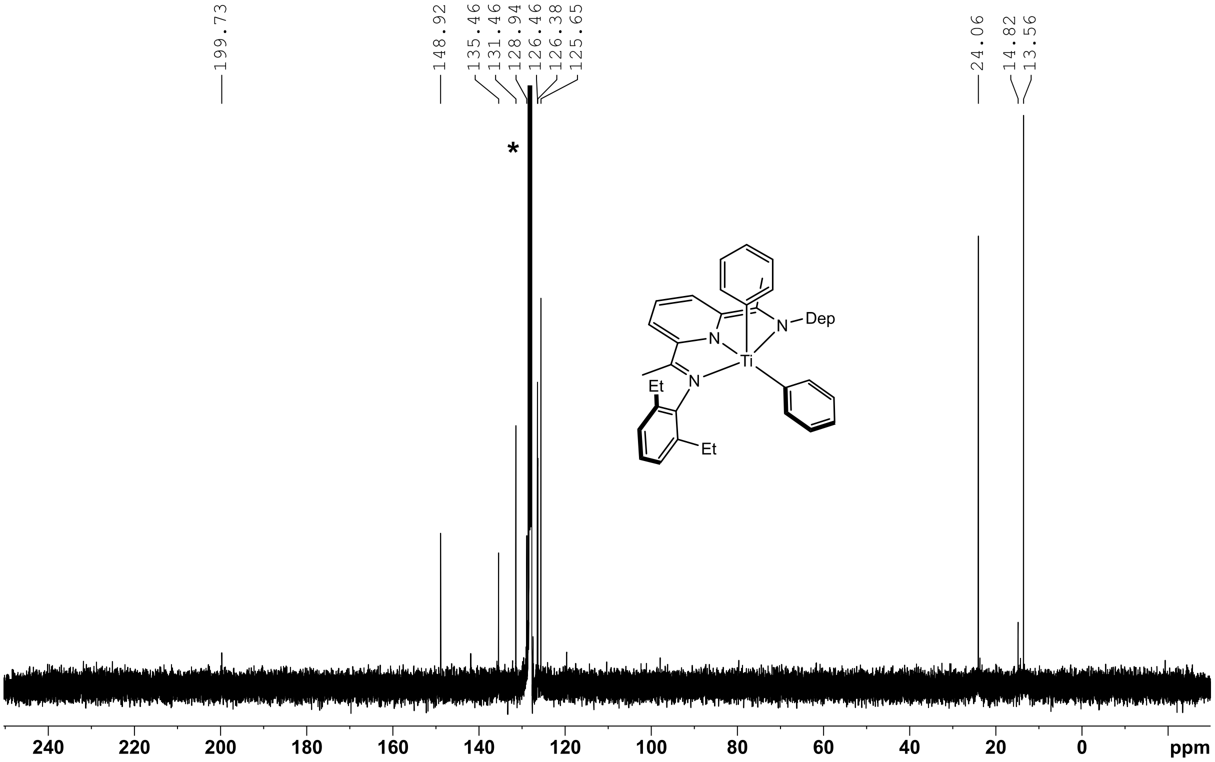

Figure S16. ^{13}C {^{1}H} NMR spectrum of (^{Et}PDI)TiPh$_2$ (600 MHz, 298 K, toluene-d_8). Residual signal of deuterated solvent is marked with an asterisk (*).

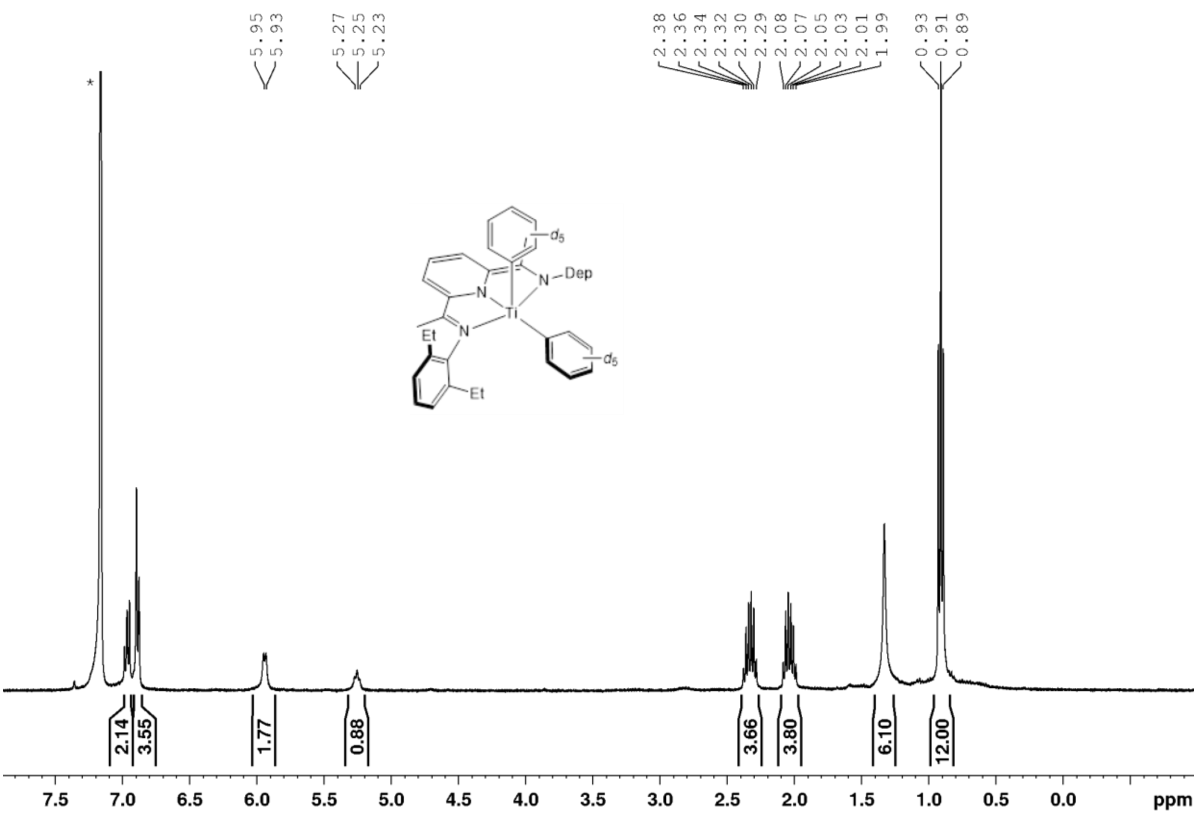

Figure S17. ^{1}H NMR spectrum of (^{Et}PDI)Ti(Ph-d_5)$_2$ (600 MHz, 298 K, benzene-d_6). Residual signal of deuterated solvent is marked with an asterisk (*).

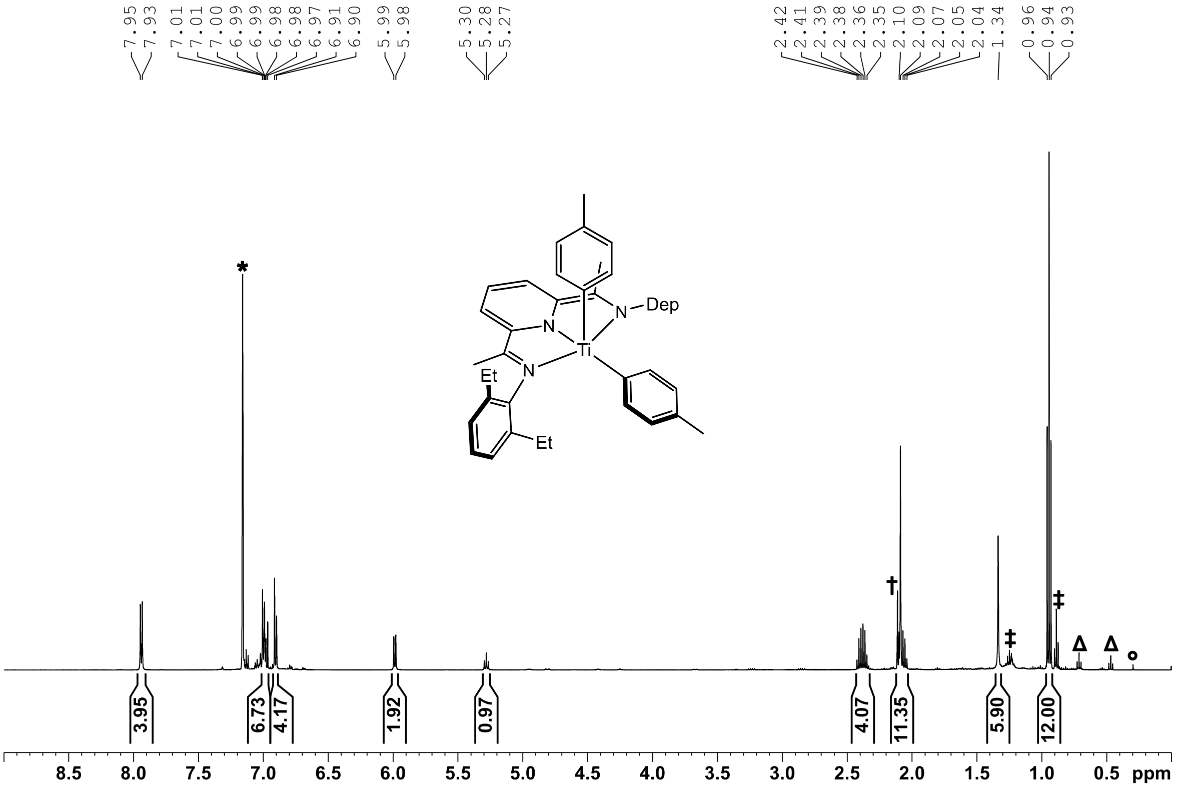

Figure S18. ^{1}H NMR spectrum of (^{Et}PDI)Ti(*p*-Tol)$_2$ (500 MHz, 298 K, benzene-d_6). Following signals are marked: Residual solvent signal (*), *n*-pentane (‡), toluene (†) and minor amounts of decomposition product (< 10%, $^{\Delta}$), silicon grease (°).

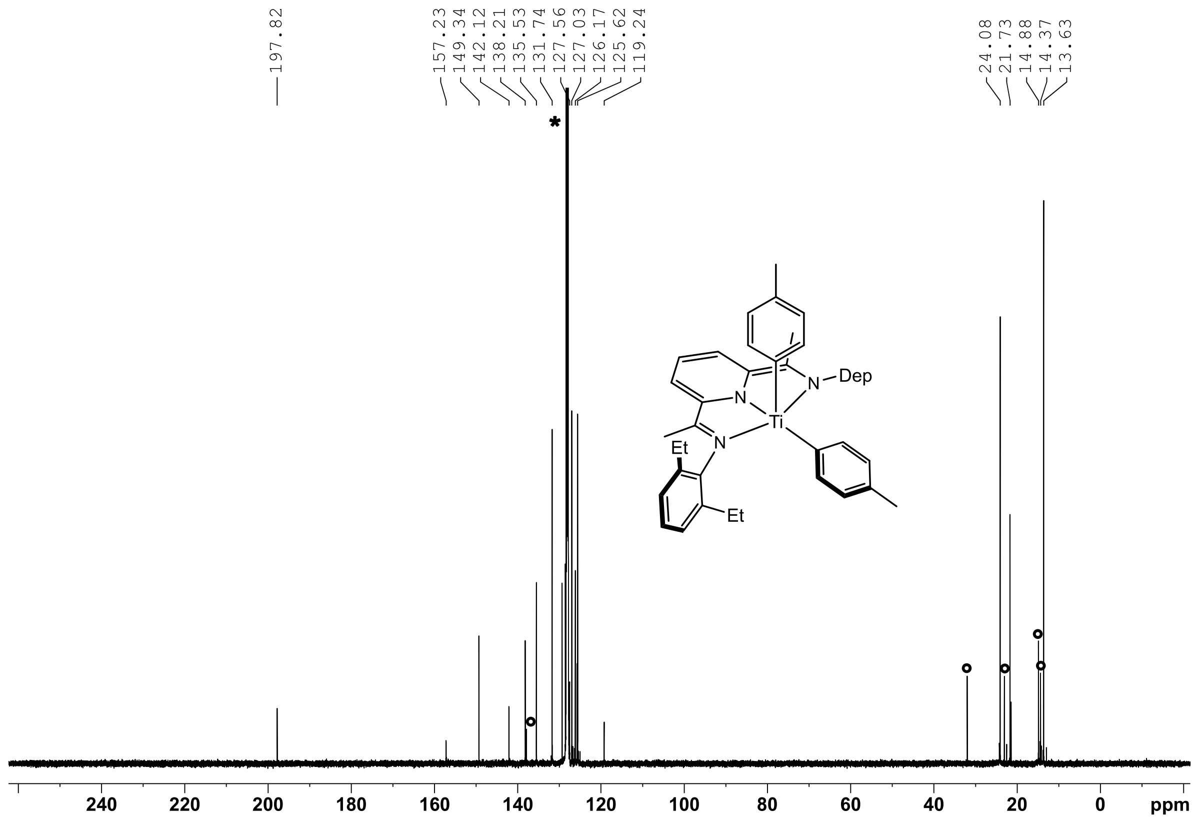

Figure S19. ^{13}C NMR spectrum of (^{Et}PDI)Ti(p-Tol)$_2$ (126 MHz, 298 K, benzene-d_6). Following signals are marked: Residual solvent signal (*), minor amounts of decomposition (°).

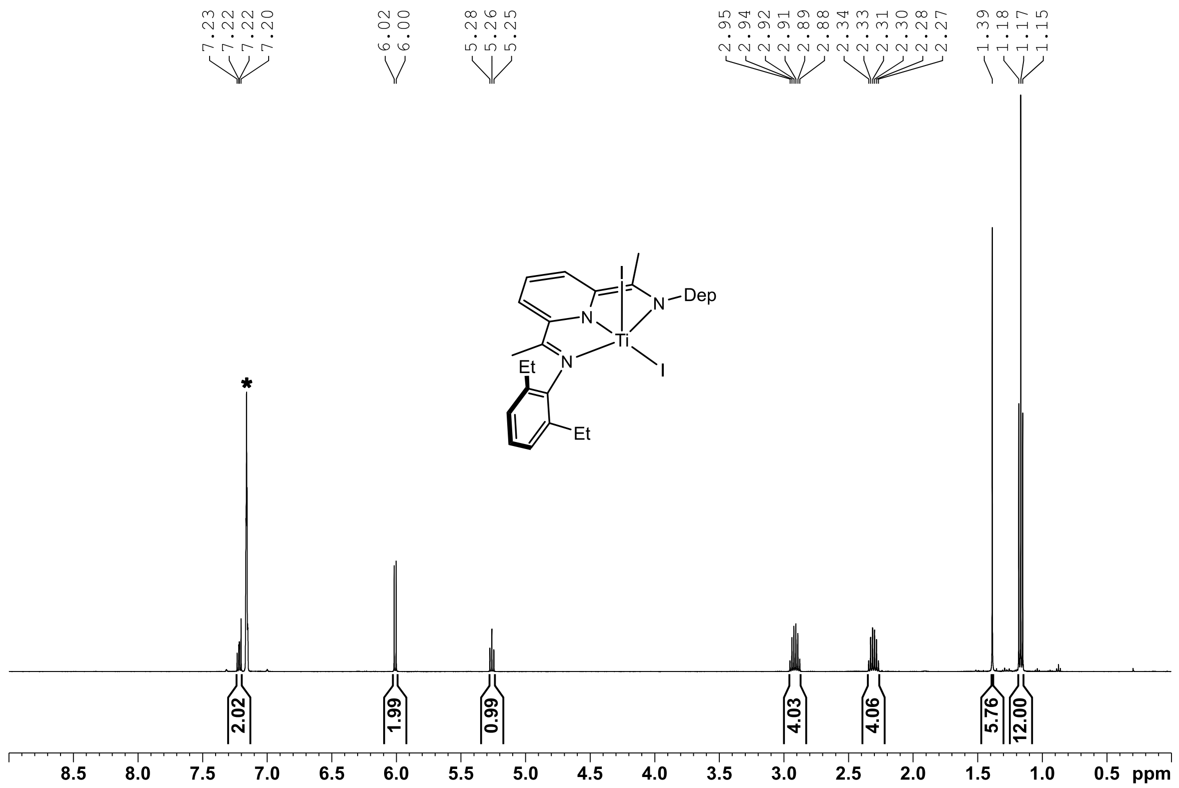

Figure S20. ^{1}H NMR spectrum of (^{Et}PDI)TiI$_2$ (500 MHz, 298 K, benzene-d_6). Residual solvent signal is marked with an asterisk (*).

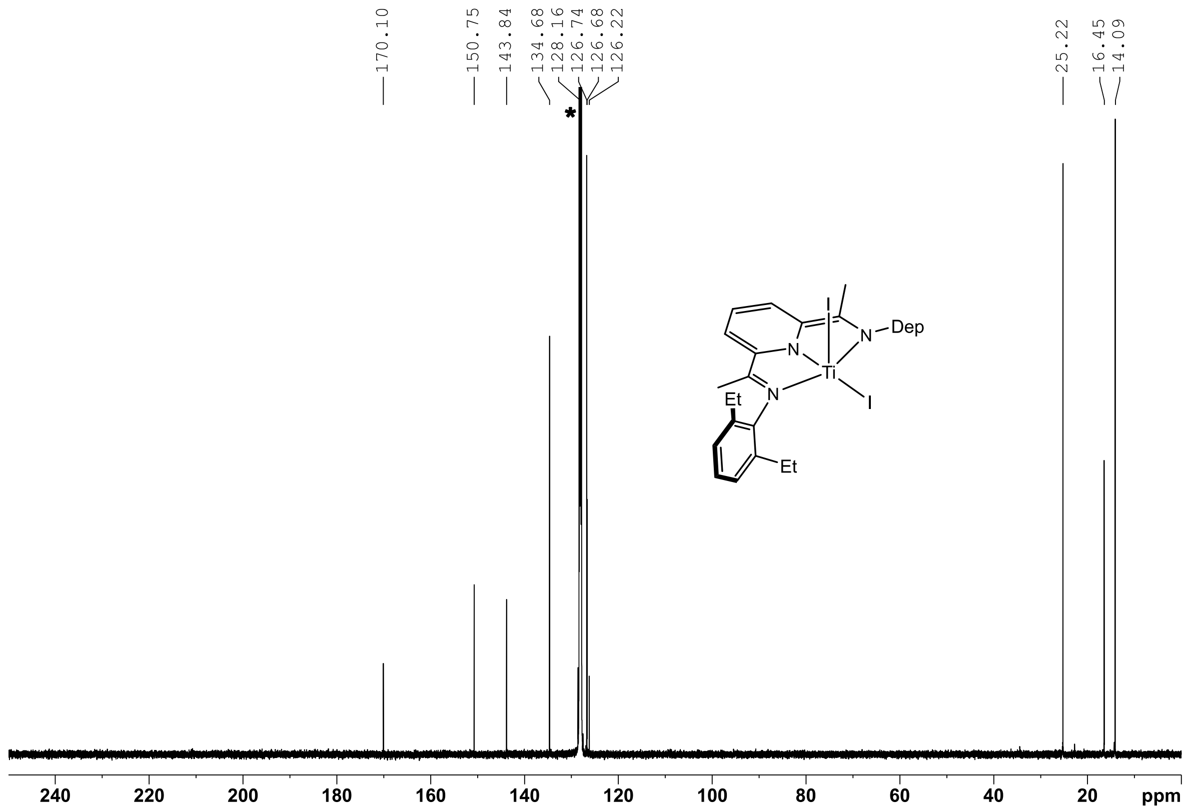

Figure S21. ^{13}C NMR spectrum of (^{Et}PDI)TiI_2 (126 MHz, 298 K, benzene-d_6). Residual solvent signal is marked with an asterisk (*).

5. NMR spectra of OIRE and trapping reactions

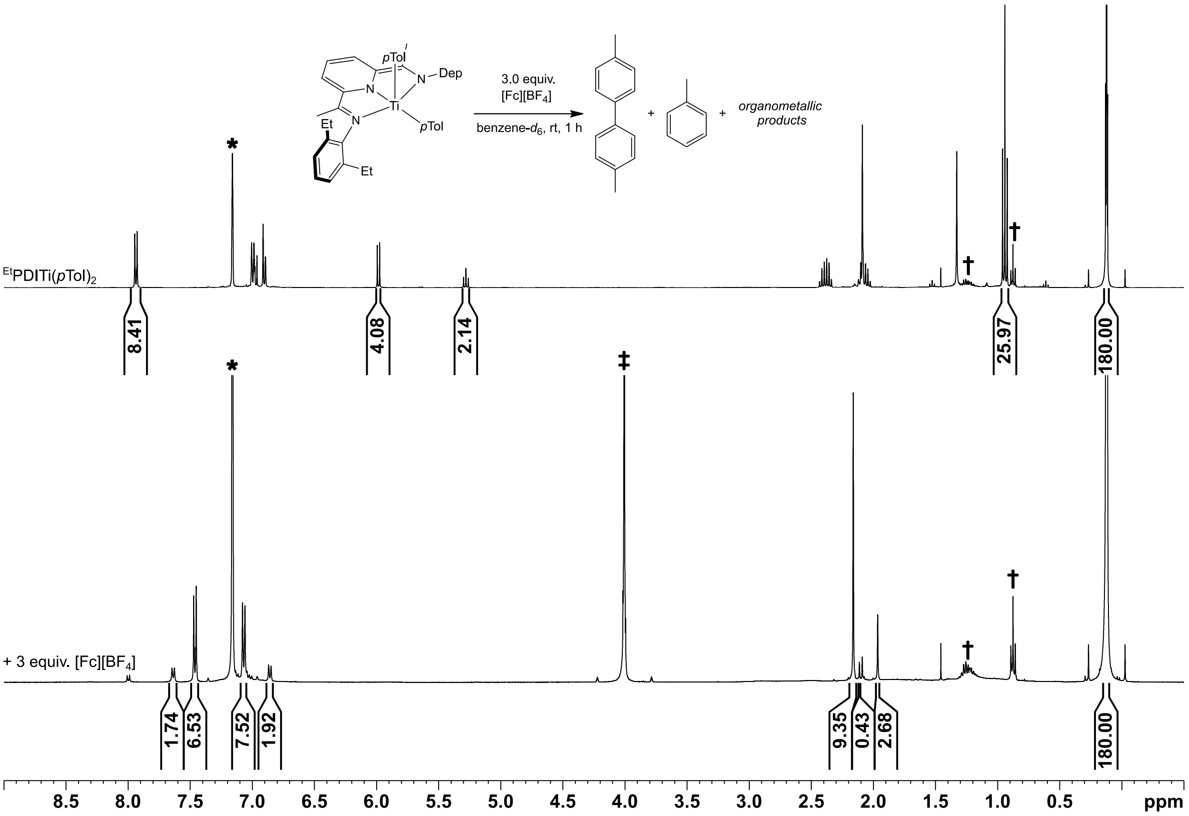

Figure S22. ^{1}H NMR spectra (400 MHz, 298 K, benzene-d_6) of the OIRE reaction of (^{Et}PDI)Ti(p-Tol)$_2$ with [Fc][BF$_4$]. Following signals are marked: Residual solvent signal (*), *n*-pentane (†), ferrocene (‡).

a)

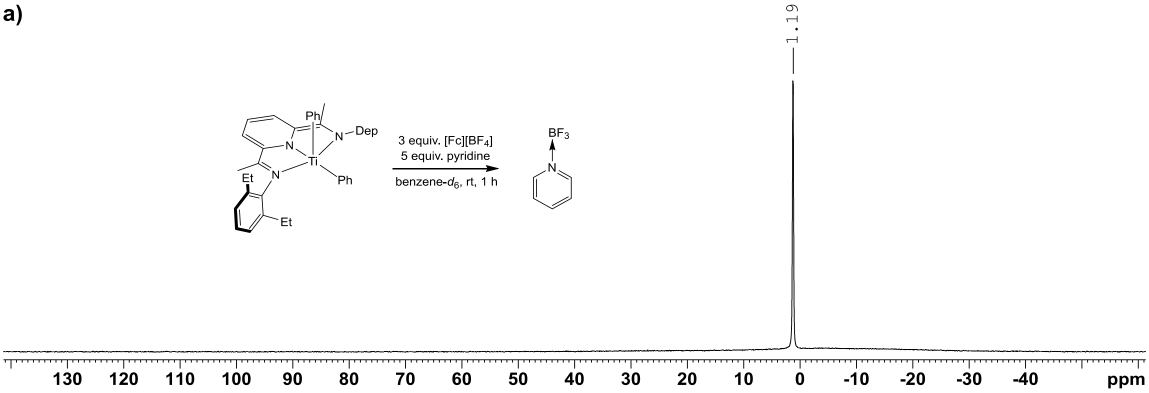

b)

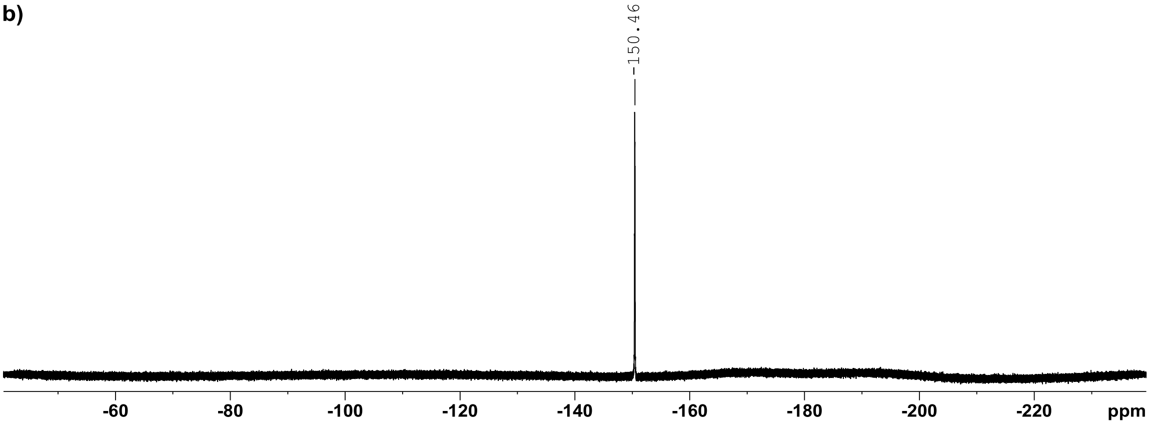

Figure S23. a) ^{11}B and **b)** ^{19}F NMR spectra (128 MHz for ^{11}B and 377 MHz for ^{19}F, 298 K, benzene-d_6) of the reaction mixture obtained by oxidation of complex **4** with [Fc][BF$_4$] in the presence of pyridine.

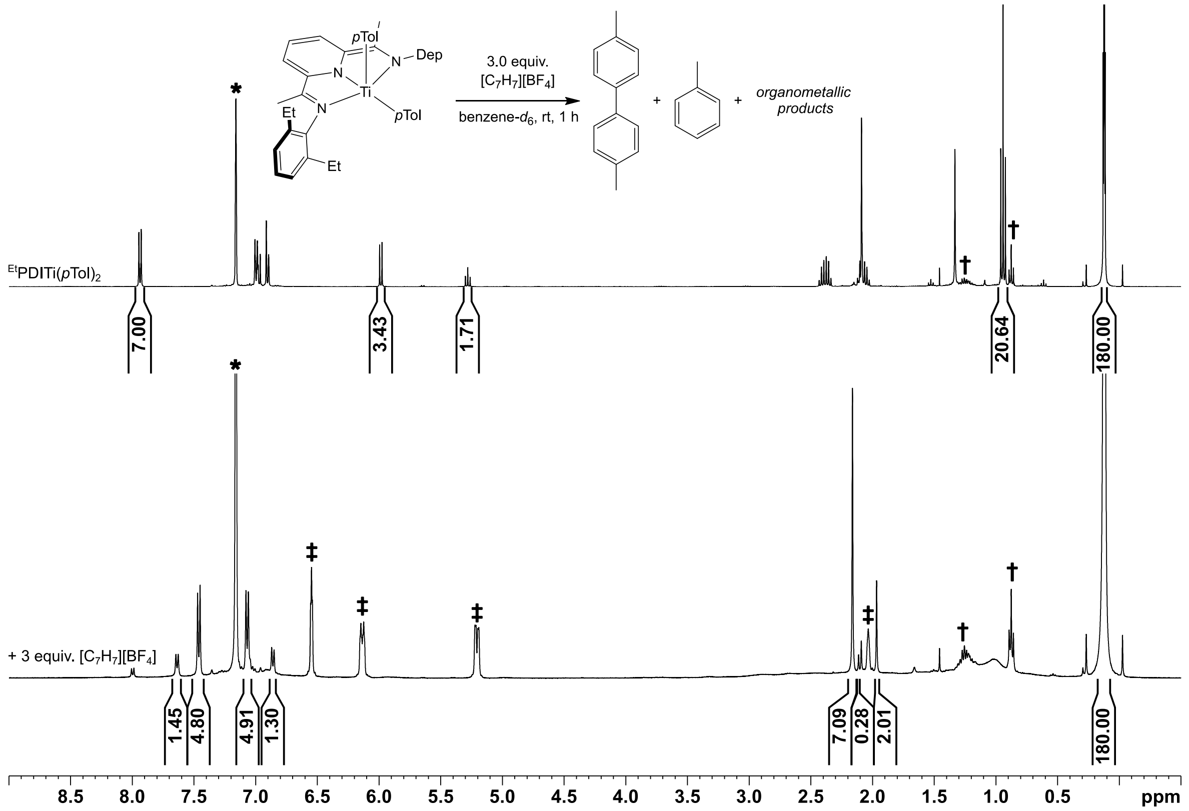

Figure S24. ^{1}H NMR spectra (400 MHz, 298 K, benzene-d_6) of the OIRE reaction of (^{Et}PDI)Ti(p-Tol)$_2$ with [C_7H_7][BF_4]. Following signals are marked: Residual solvent signal (*), *n*-pentane (†), bitropyl (‡).

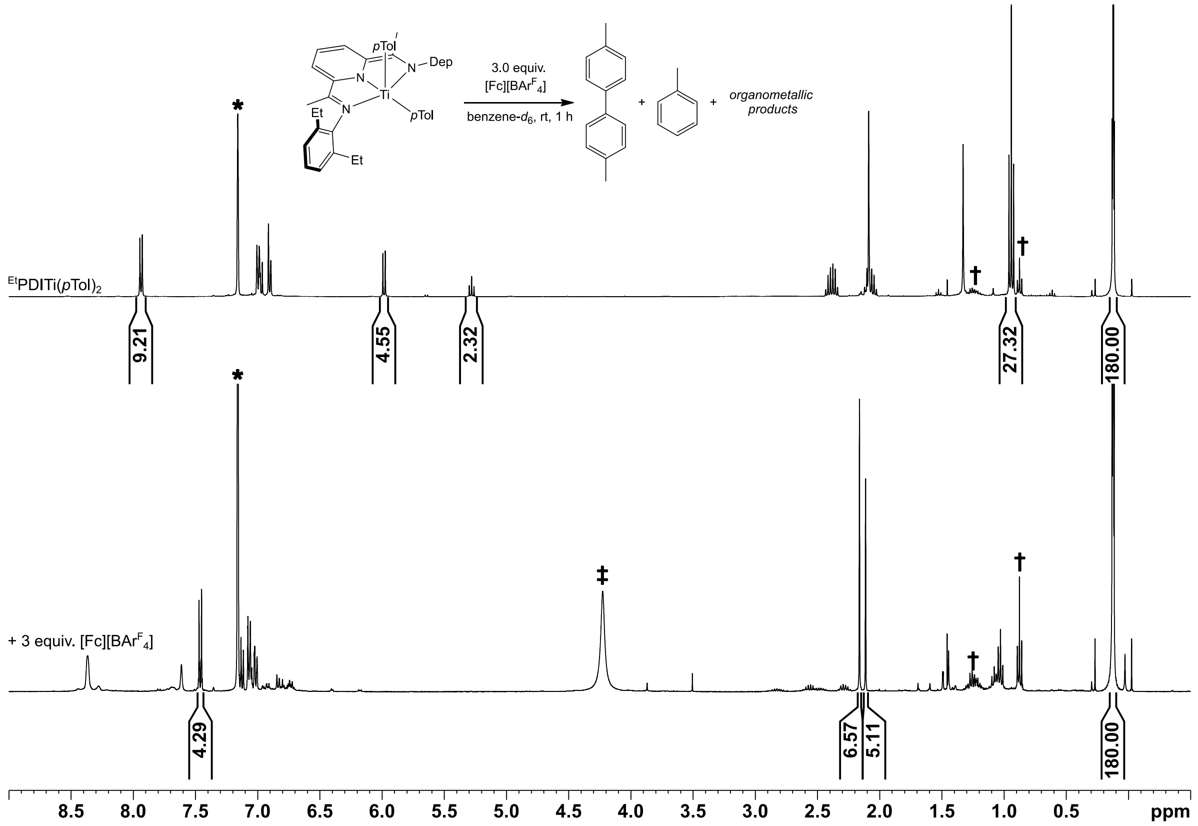

Figure S25. ^{1}H NMR spectra (400 MHz, 298 K, benzene-d_6) of the OIRE reaction of (^{Et}PDI)Ti(p-Tol)$_2$ with [Fc][$BAr^F{}_4$]. Following signals are marked: Residual solvent signal (*), *n*-pentane (†), ferrocienium (‡).

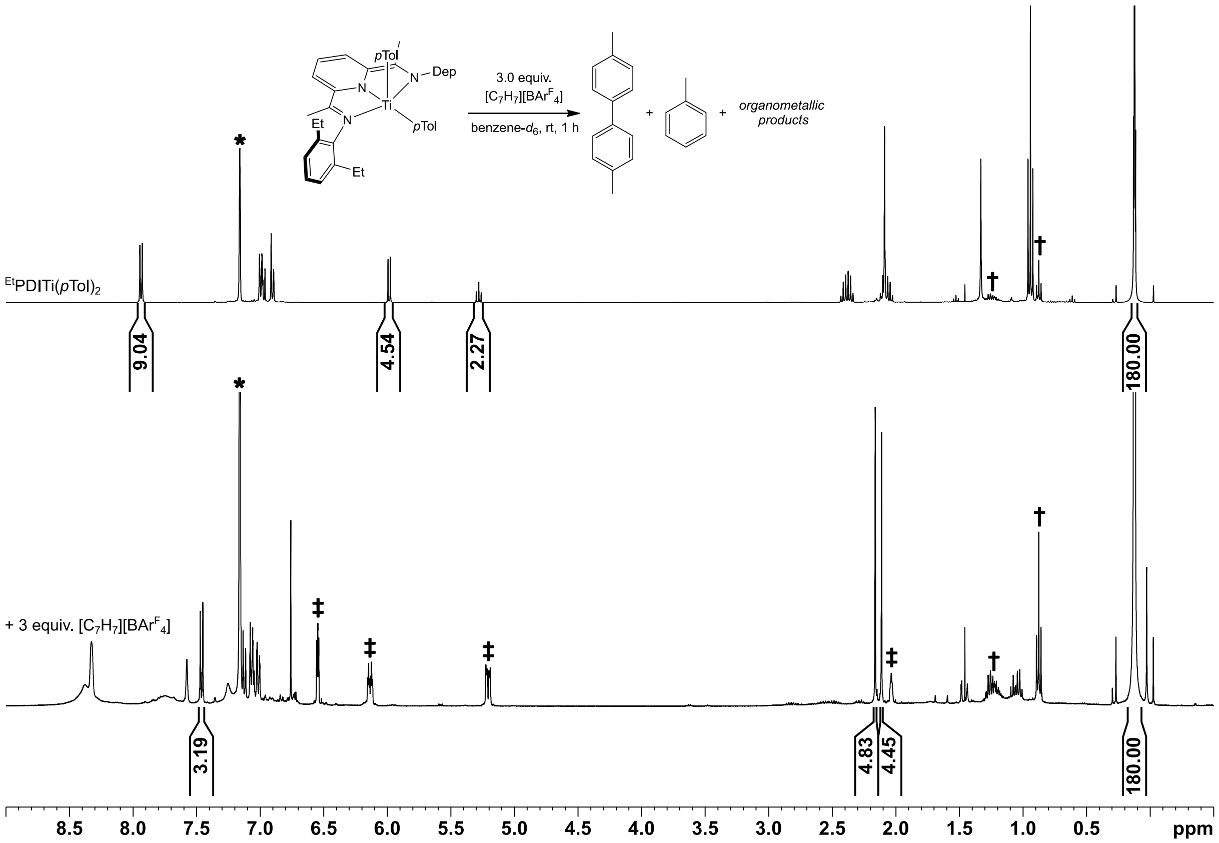

Figure S26. ^{1}H NMR spectra (400 MHz, 298 K, benzene-d_6) of the OIRE reaction of (^{Et}PDI)Ti(p-Tol)$_2$ with [C_7H_7][$BAr^F{}_4$]. Following signals are marked: Residual solvent signal (*), *n*-pentane (†), bitropyl (‡).

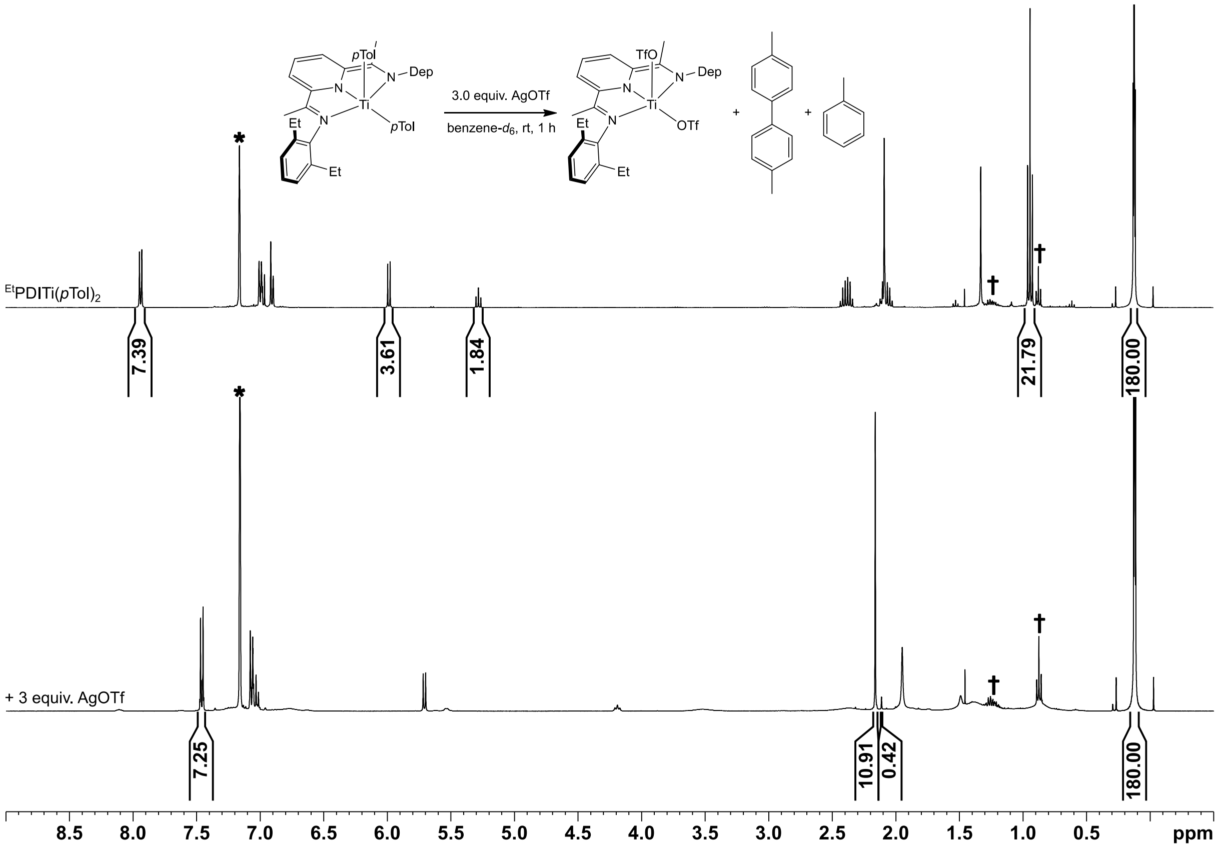

Figure S27. ^{1}H NMR spectra (400 MHz, 298 K, benzene-d_6) of the OIRE reaction of (^{Et}PDI)Ti(p-Tol)$_2$ with AgOTf. Following signals are marked: Residual solvent signal (*), *n*-pentane (†).

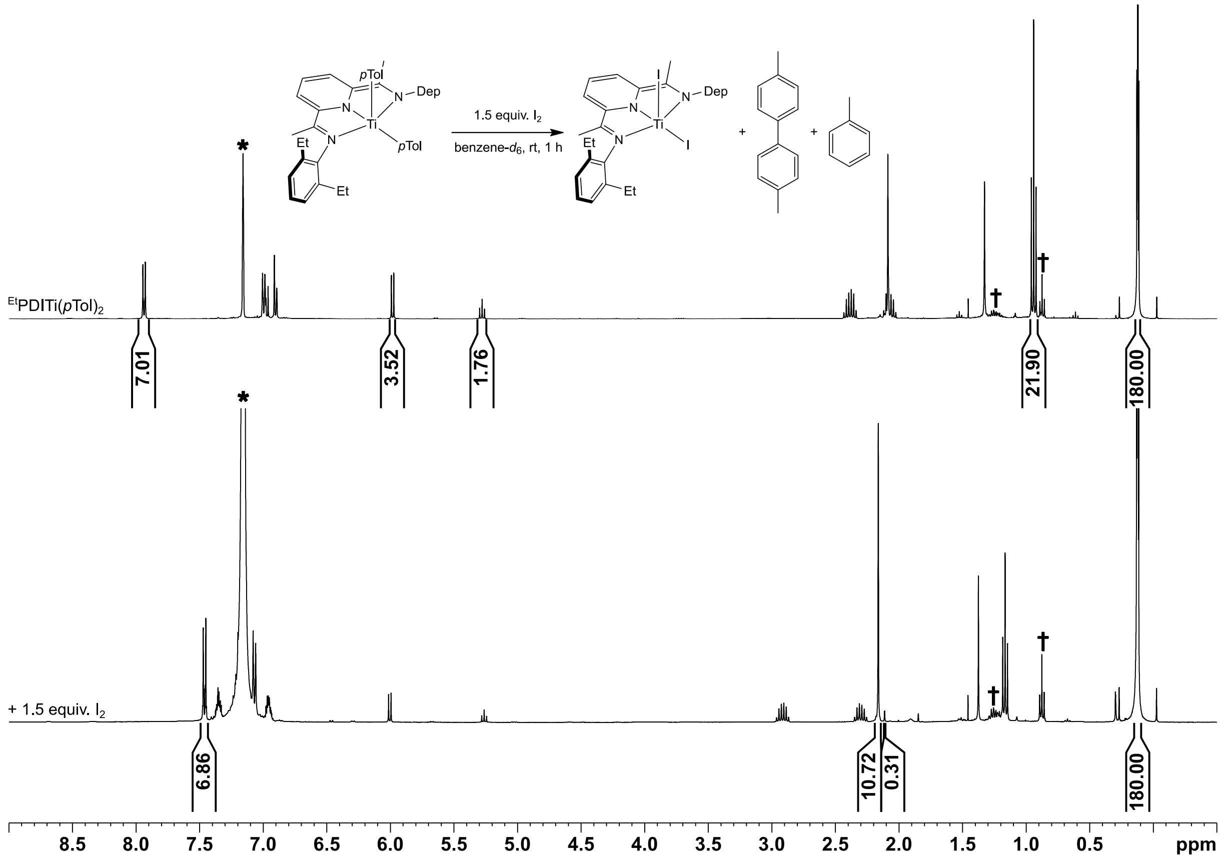

Figure S28. ^{1}H NMR spectra (400 MHz, 298 K, benzene-*d*$_6$) of the OIRE reaction of (^{Et}PDI)Ti(*p*-Tol)$_2$ with I_2. Following signals are marked: Residual solvent signal (*), *n*-pentane (†).

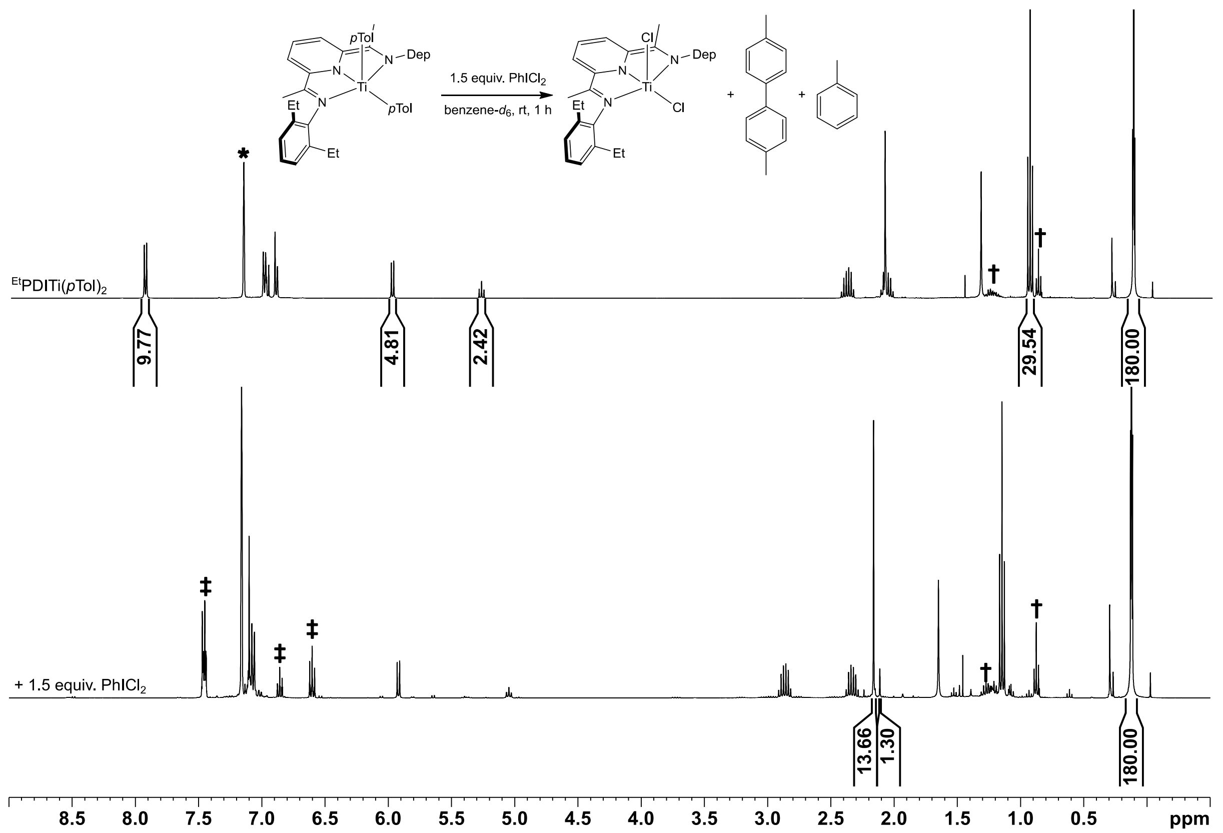

Figure S29. ^{1}H NMR spectra (400 MHz, 298 K, benzene-*d*$_6$) of the OIRE reaction of (^{Et}PDI)Ti(*p*-Tol)$_2$ with $PhICl_2$. Following signals are marked: Residual solvent signal (*), *n*-pentane (†), iodobenzene (‡).

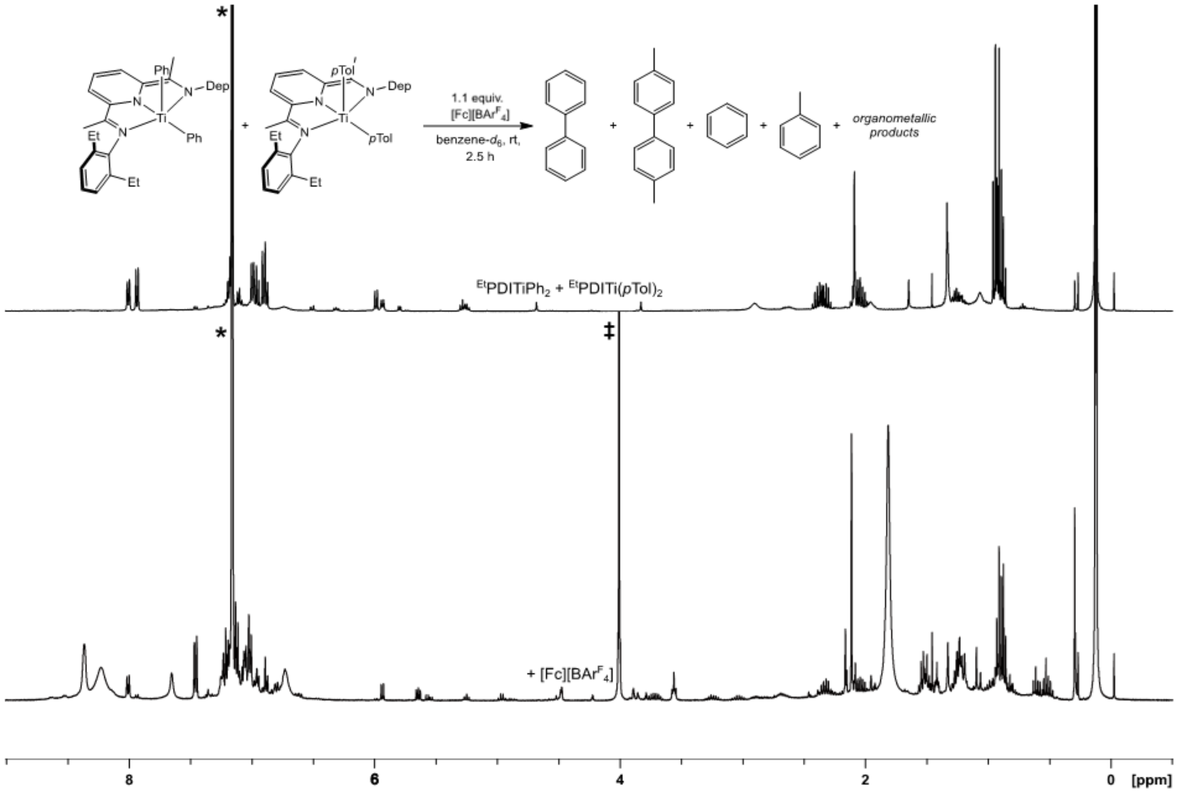

Figure S30. ^{1}H NMR spectra (400 MHz, 298 K, benzene-d_6) of the cross-over experiment using (^{Et}PDI)Ti(p-Tol)$_2$ and (^{Et}PDI)TiPh$_2$ and [Fc][BAr$^F_4$]. Following signals are marked: Residual solvent signal (*), ferrocene (‡).

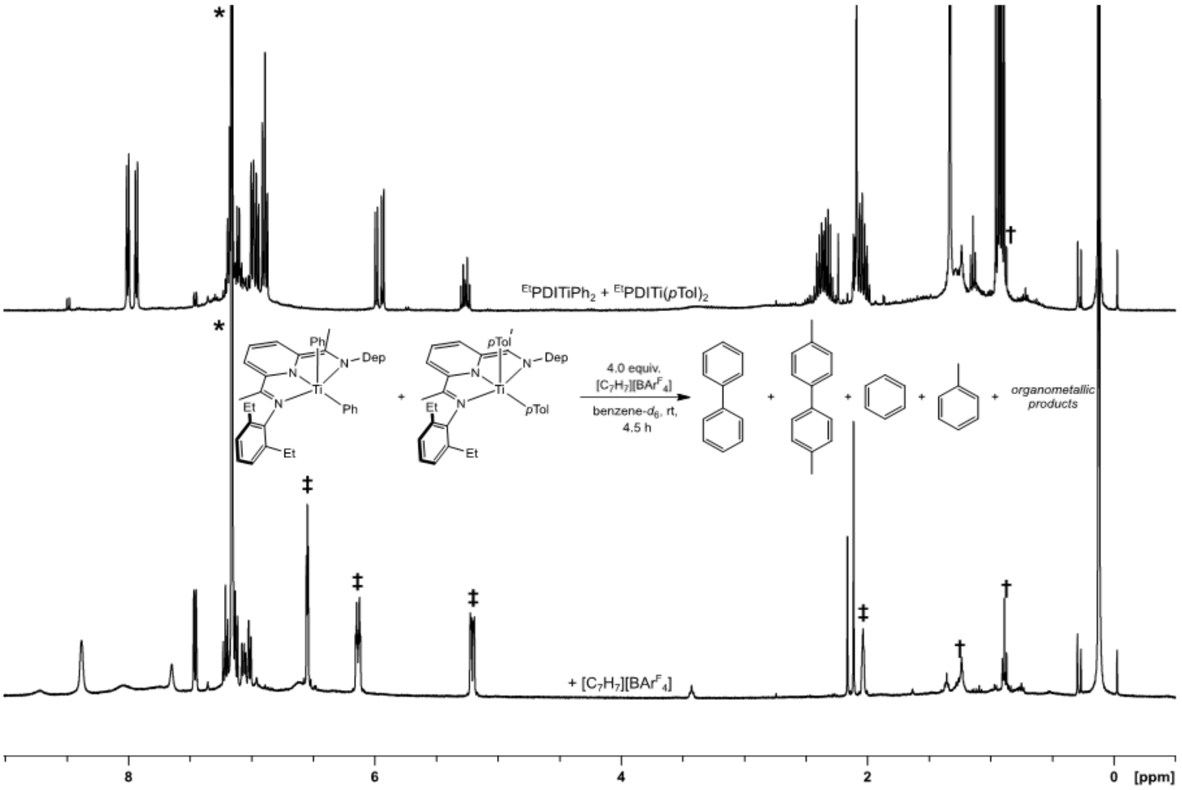

Figure S31. ^{1}H NMR spectra (400 MHz, 298 K, benzene-d_6) of the cross-over experiment using (^{Et}PDI)Ti(p-Tol)$_2$ and (^{Et}PDI)TiPh$_2$ and [C_7H_7][BAr$^F_4$]. Following signals are marked: Residual solvent signal (*), *n*-pentane (†), bitropyl (‡).a

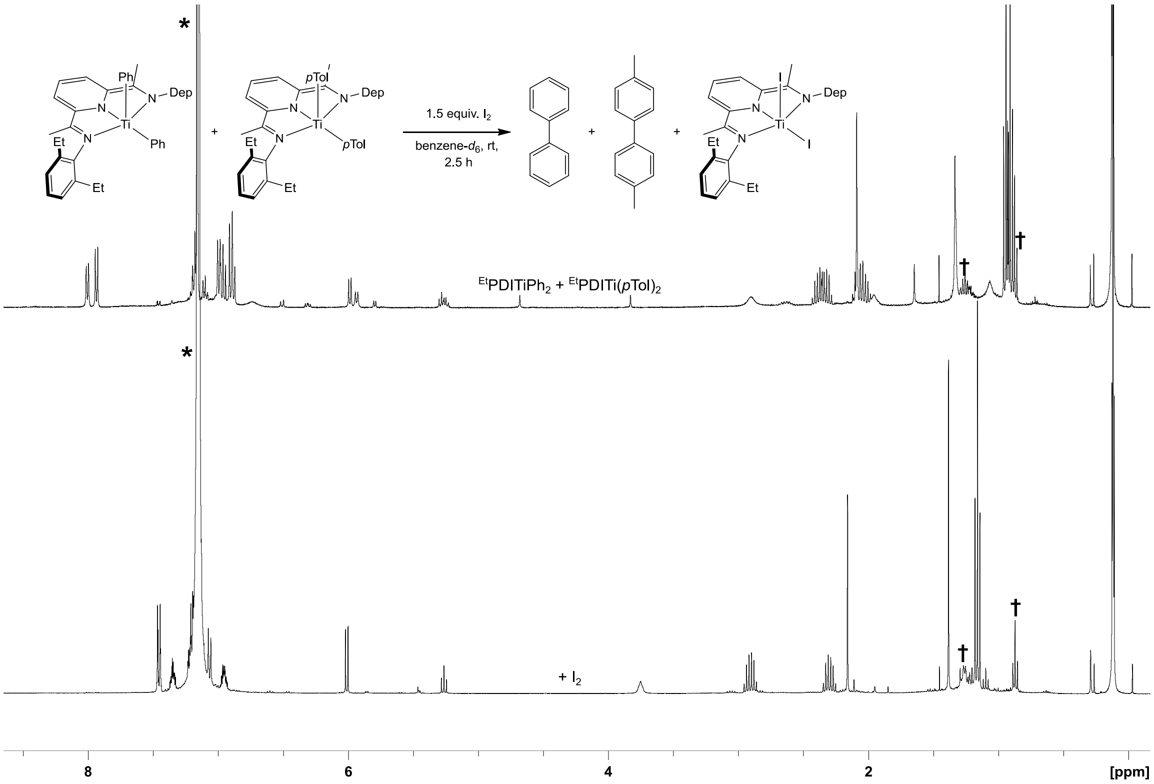

Figure S32. ^{1}H NMR spectra (400 MHz, 298 K, benzene-d_6) of the cross-over experiment using (^{Et}PDI)Ti(p-Tol)$_2$ and (^{Et}PDI)TiPh$_2$ and I_2. Following signals are marked: Residual solvent signal (*), *n*-pentane (†).

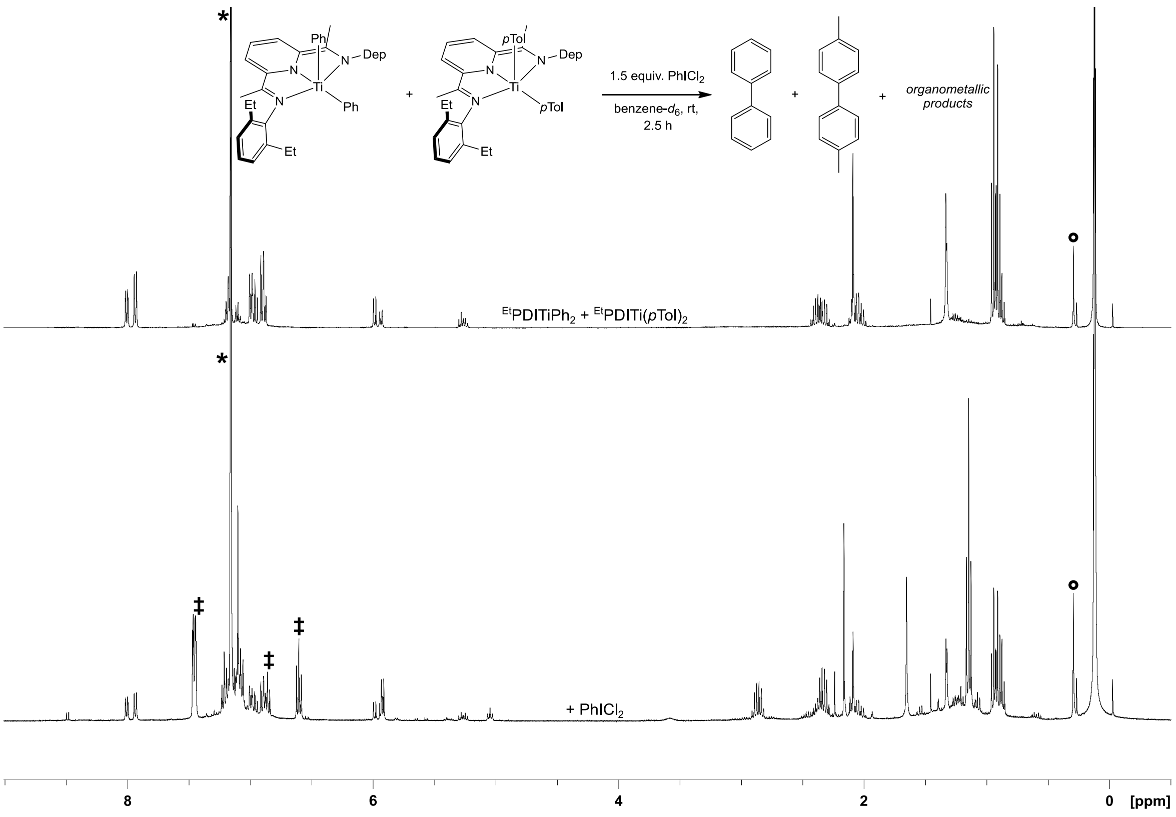

Figure S33. ^{1}H NMR spectra (400 MHz, 298 K, benzene-d_6) of the cross-over experiment using (^{Et}PDI)Ti(p-Tol)$_2$ and (^{Et}PDI)TiPh$_2$ and PhICl$_2$. Following signals are marked: Residual solvent signal (*), iodobenzene (‡), silicon grease (°).

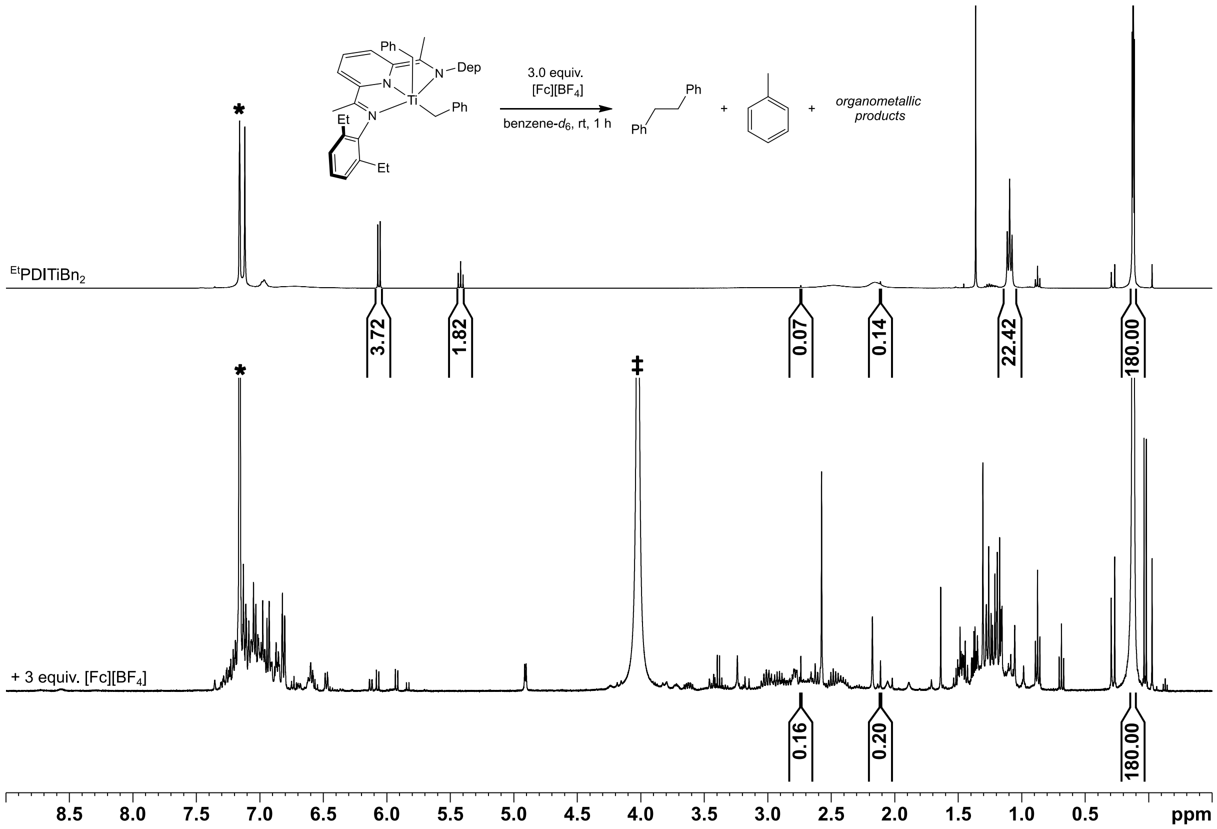

Figure S34. ^{1}H NMR spectra (400 MHz, 298 K, benzene-d_6) of the OIRE reaction of (^{Et}PDI)Ti(CH_2Ph)$_2$ with [Fc][BF_4]. Following signals are marked: Residual solvent signal (*), ferrocenium (‡).

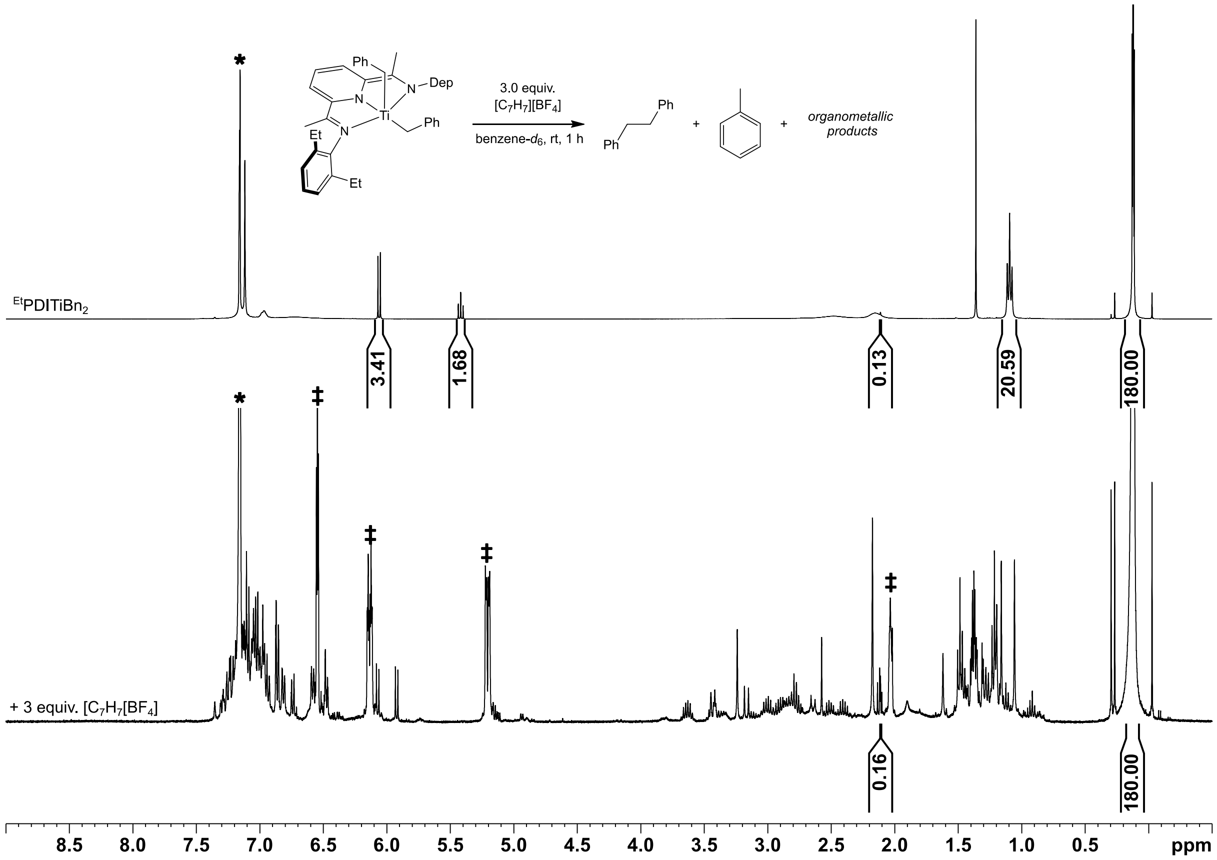

Figure S35. ^{1}H NMR spectra (400 MHz, 298 K, benzene-d_6) of the OIRE reaction of (^{Et}PDI)Ti(CH_2Ph)$_2$ with [C_7H_7][BF_4]. Following signals are marked: Residual solvent signal (*), bitropyl (‡).

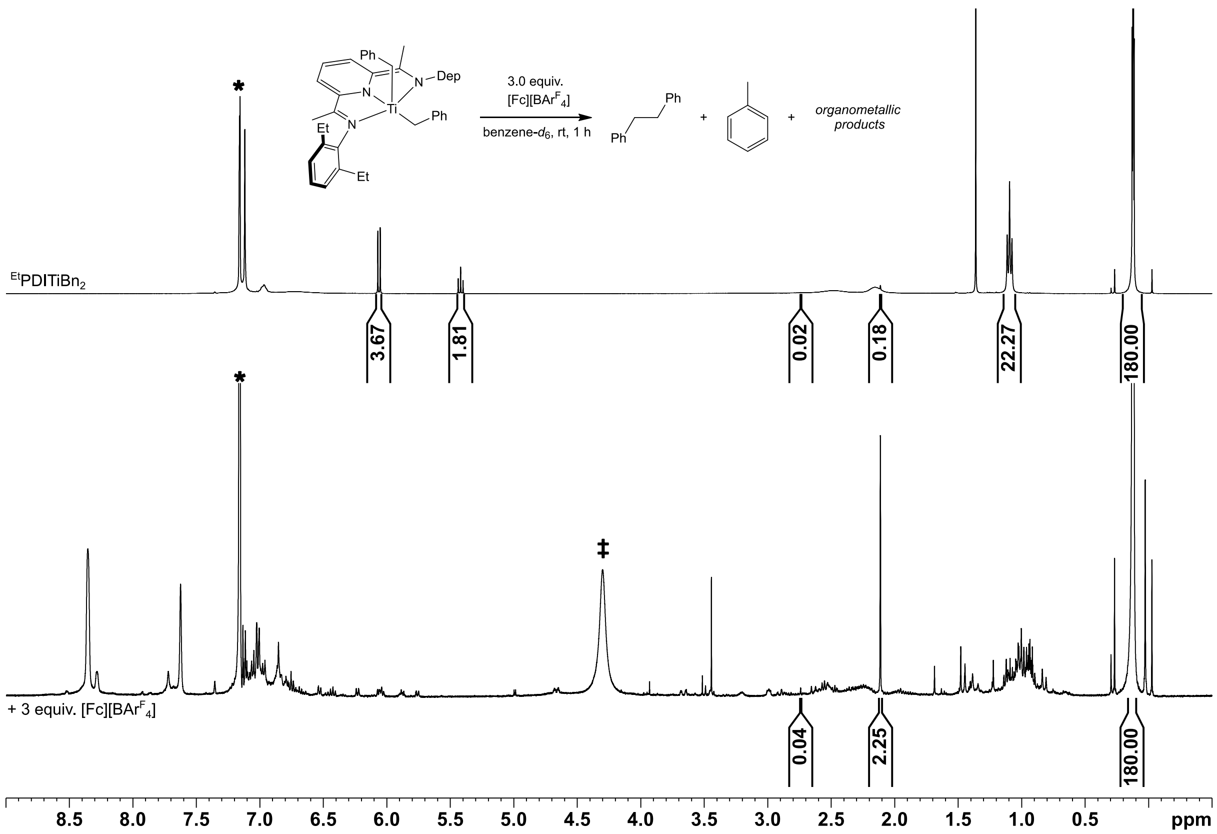

Figure S36. ^{1}H NMR spectra (400 MHz, 298 K, benzene-*d*$_6$) of the OIRE reaction of (^{Et}PDI)Ti($CH_2Ph)_2$ with [Fc][BAr^F$_4$]. Following signals are marked: Residual solvent signal (*), ferrocenium (‡).

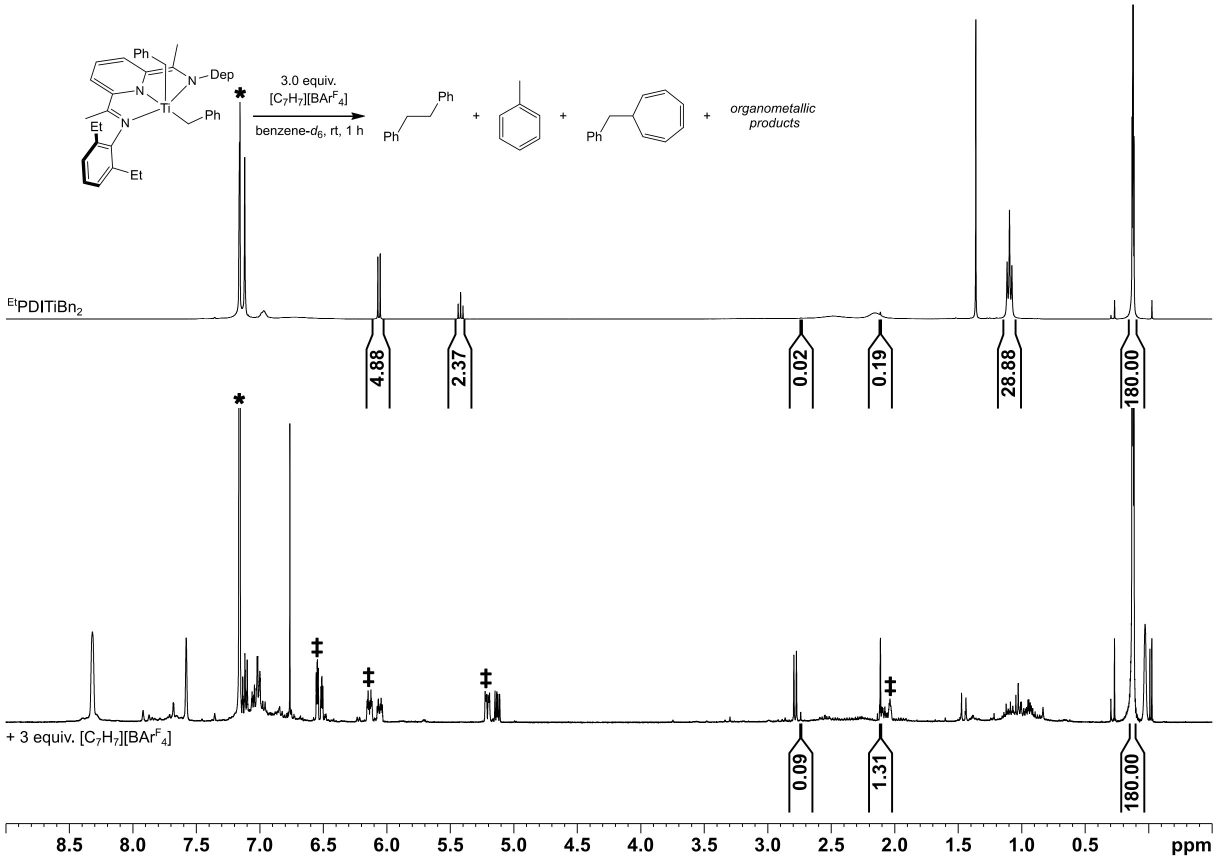

Figure S37. ^{1}H NMR spectra (400 MHz, 298 K, benzene-*d*$_6$) of the OIRE reaction of (^{Et}PDI)Ti($CH_2Ph)_2$ with [C_7H_7][BAr^F$_4$]. Following signals are marked: Residual solvent signal (*), bitropyl (‡).

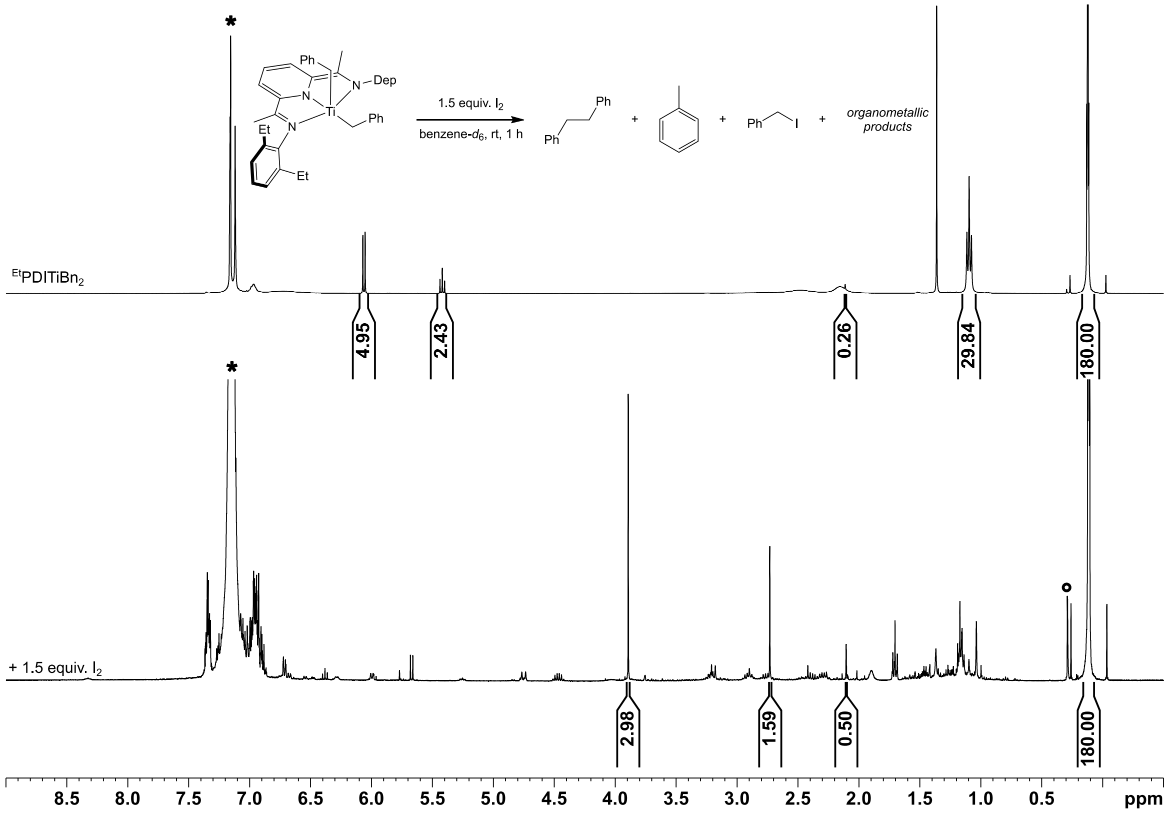

Figure S38. ^{1}H NMR spectra (400 MHz, 298 K, benzene-*d*$_6$) of the OIRE reaction of (^{Et}PDI)Ti(CH$_2$Ph)$_2$ with I$_2$. Following signals are marked: Residual solvent signal (*), silicon grease (°).

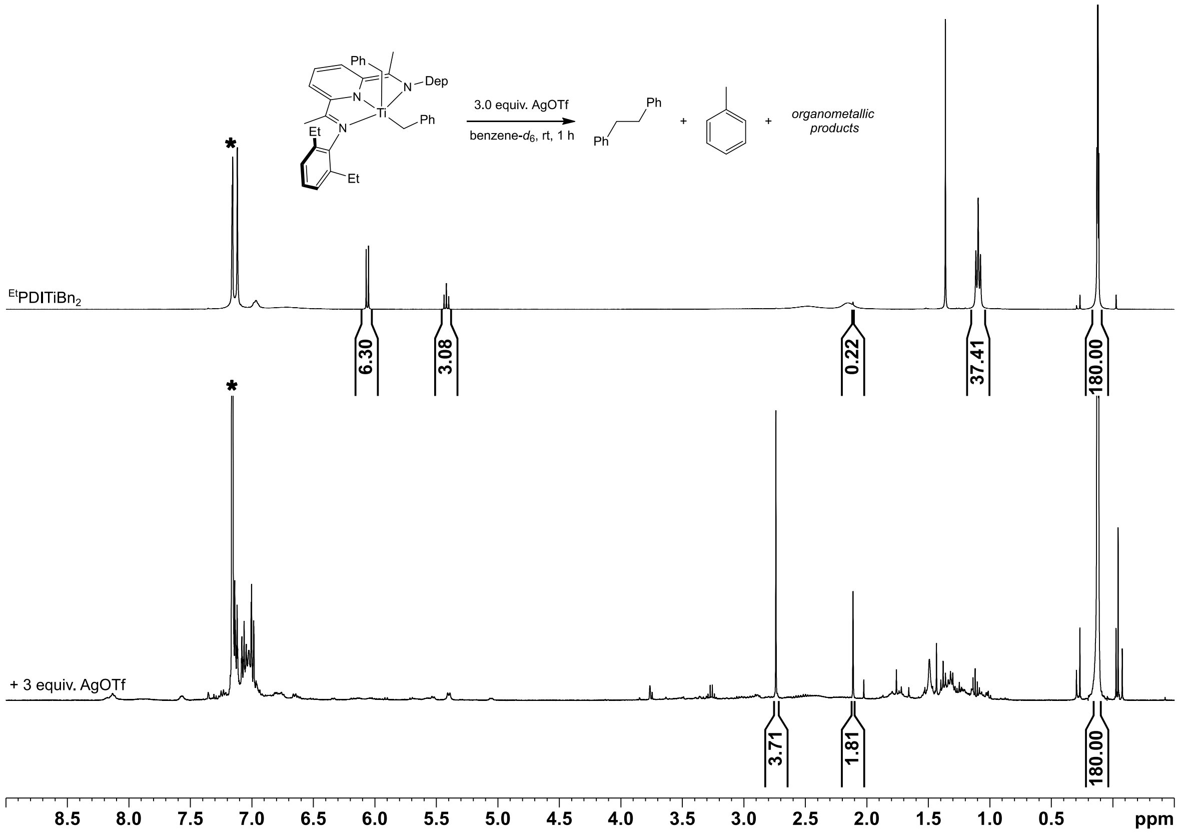

Figure S39. ^{1}H NMR spectra (400 MHz, 298 K, benzene-*d*$_6$) of the OIRE reaction of (^{Et}PDI)Ti(CH$_2$Ph)$_2$ with AgOTf. Following signals are marked: The residual signal of deuterated solvent is marked with an asterisk (*).

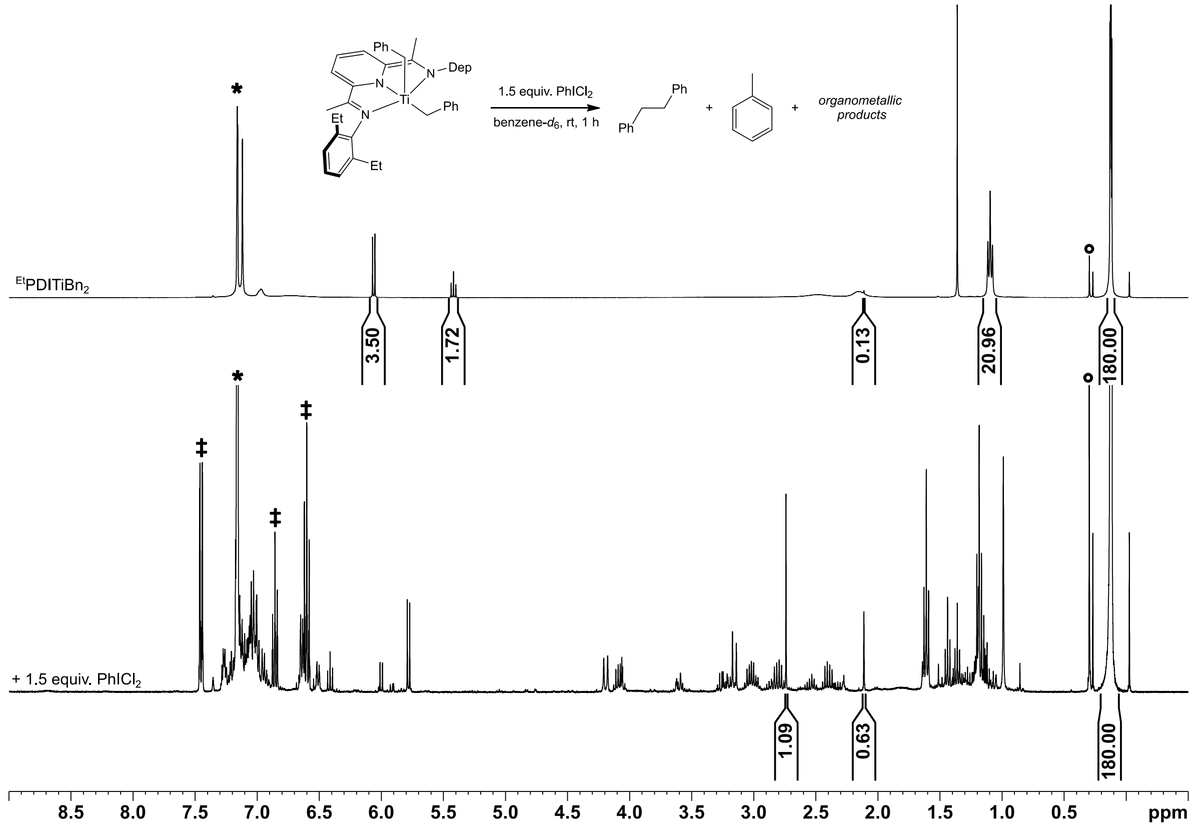

Figure S40. ^{1}H NMR spectra (400 MHz, 298 K, benzene-d_6) of the OIRE reaction of (^{Et}PDI)Ti(CH_2Ph)$_2$ with $PhICl_2$. Following signals are marked: Residual solvent signal (*), iodobenzene (‡), silicon grease (°).

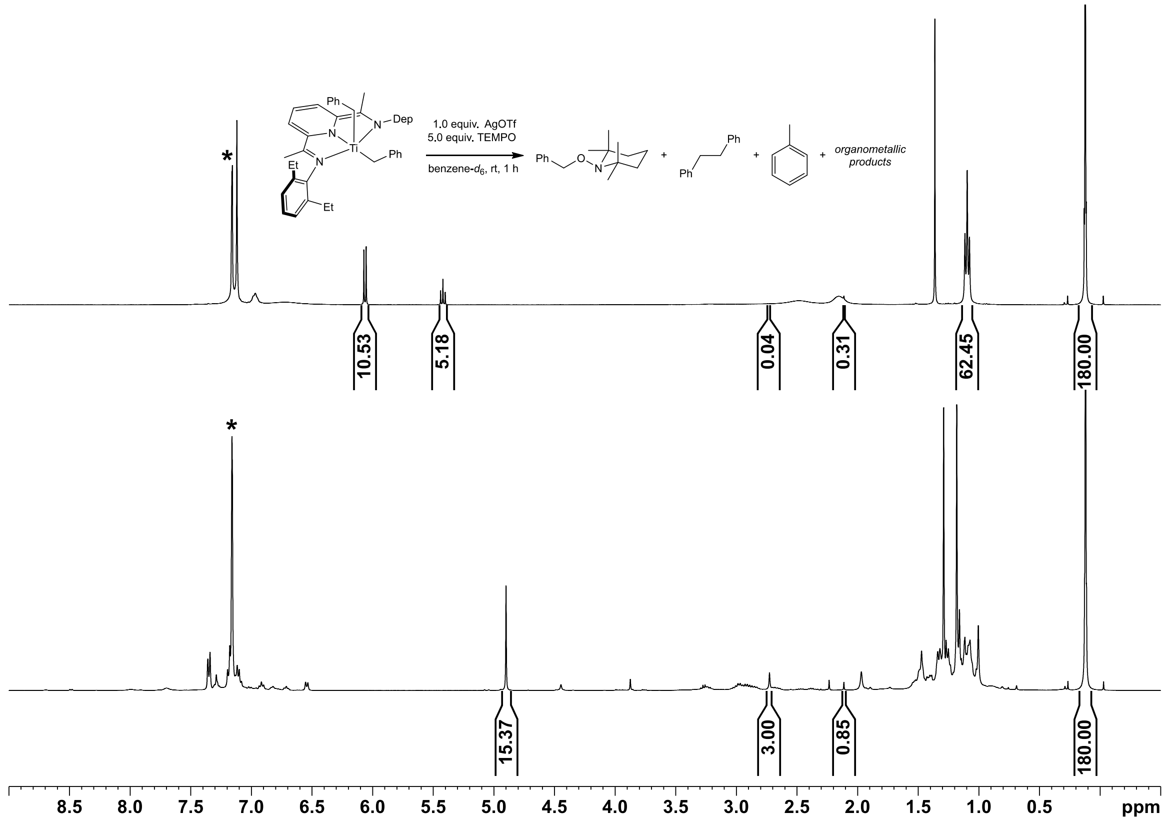

Figure S41. ^{1}H NMR spectra (400 MHz, 298 K, benzene-d_6) of the oxidation of (^{Et}PDI)Ti(CH_2Ph)$_2$ with AgOTf in the presence of TEMPO. Residual signal of deuterated solvent is marked with an asterisk (*).

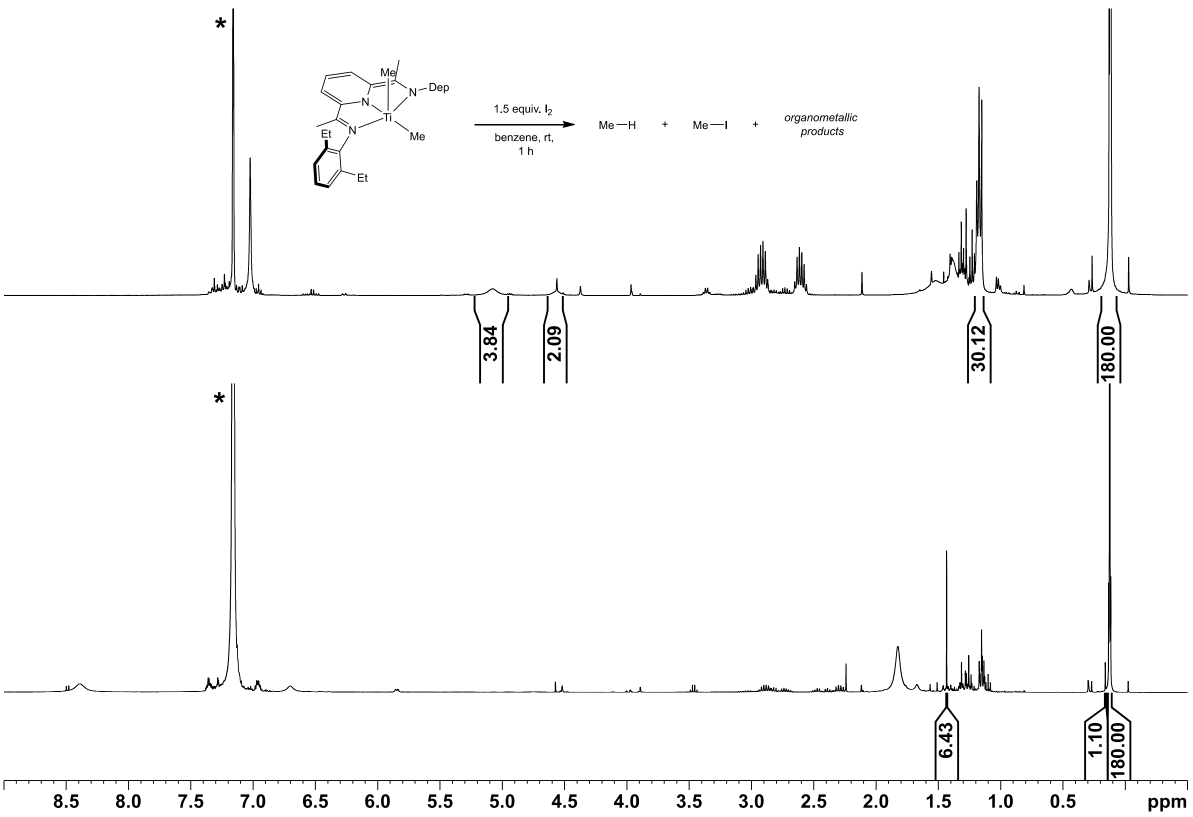

Figure S42. ^{1}H NMR spectra (400 MHz, 298 K, benzene-d_6) of the oxidation of (^{Et}PDI)TiMe$_2$ with I$_2$. The residual signal of deuterated solvent is marked with an asterisk (*).

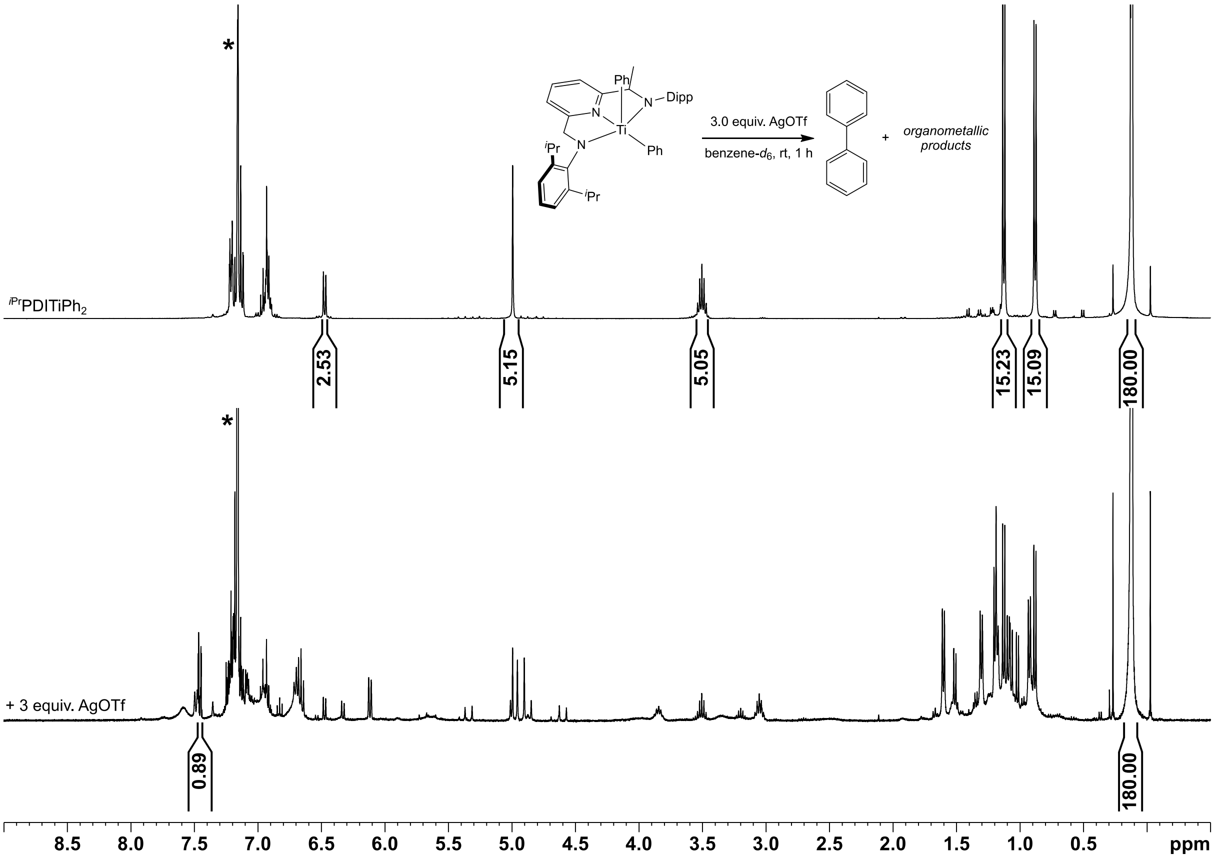

Figure S43. ^{1}H NMR spectra (400 MHz, 298 K, benzene-d_6) of the oxidation of (^{iPr}PDA)TiPh$_2$ with AgOTf. The residual signal of deuterated solvent is marked with an asterisk (*).

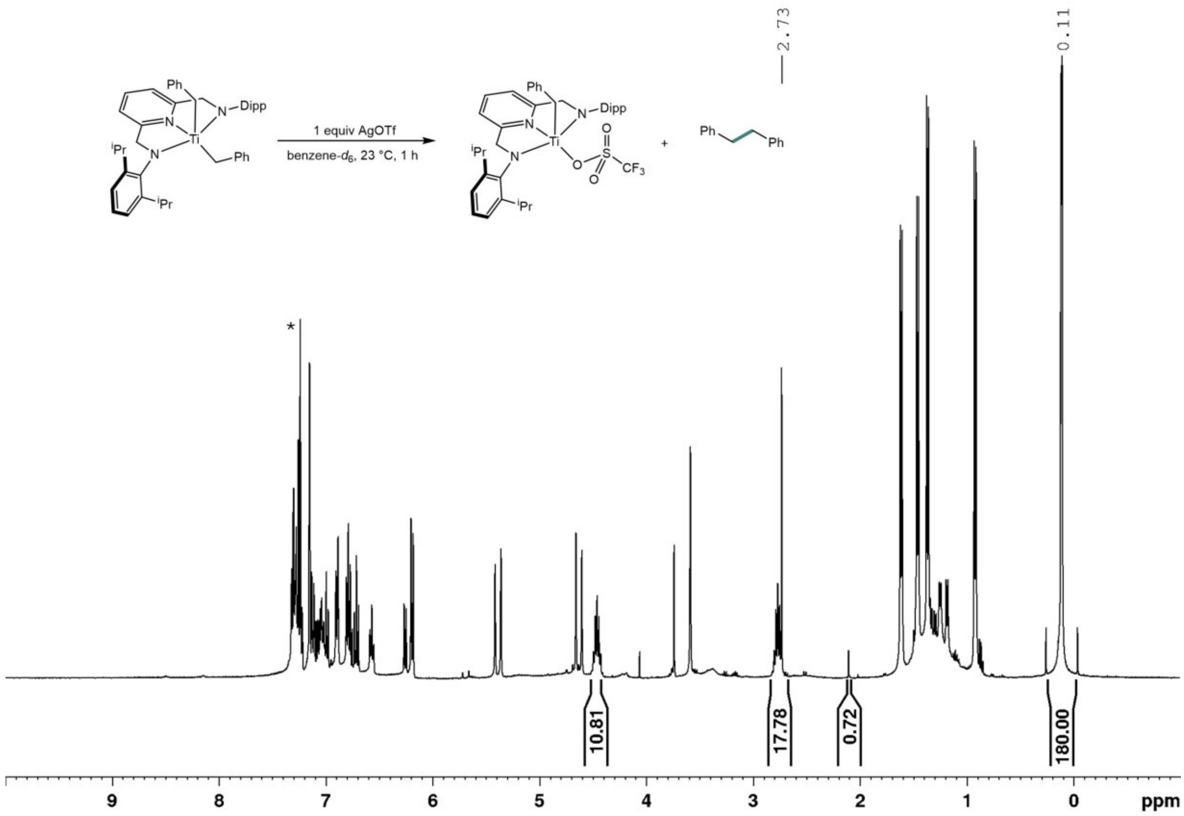

Figure S44. ^{1}H NMR spectrum (400 MHz, 298 K, benzene-d_6) of the oxidation of (^{iPr}PDA)Ti(CH_2Ph)$_2$ with AgOTf. The residual signal of deuterated solvent is marked with an asterisk (*).

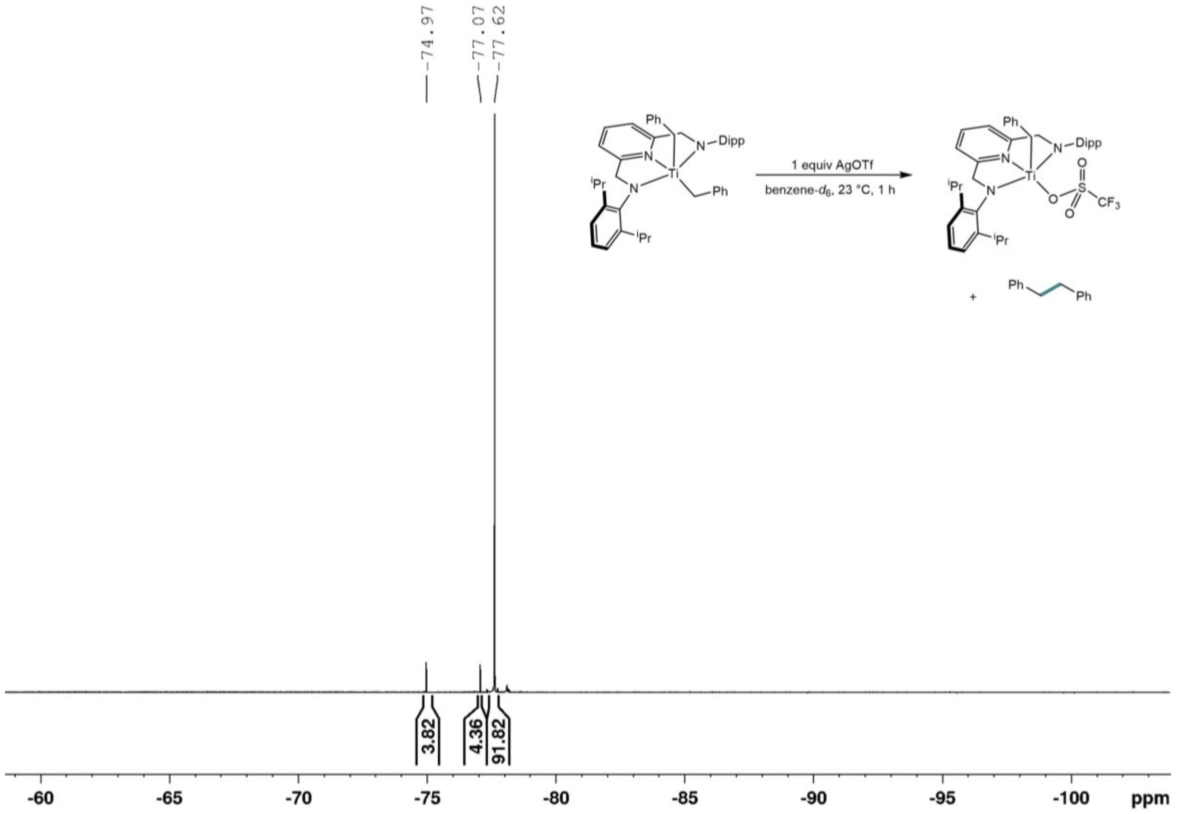

Figure S45. ^{19}F NMR spectrum (377 MHz, 298 K, benzene-d_6) of the oxidation of (^{iPr}PDA)Ti(CH_2Ph)$_2$ with AgOTf showing that >90% of fluorine-containing species are (^{iPr}PDA)Ti(CH_2Ph)(OTf).

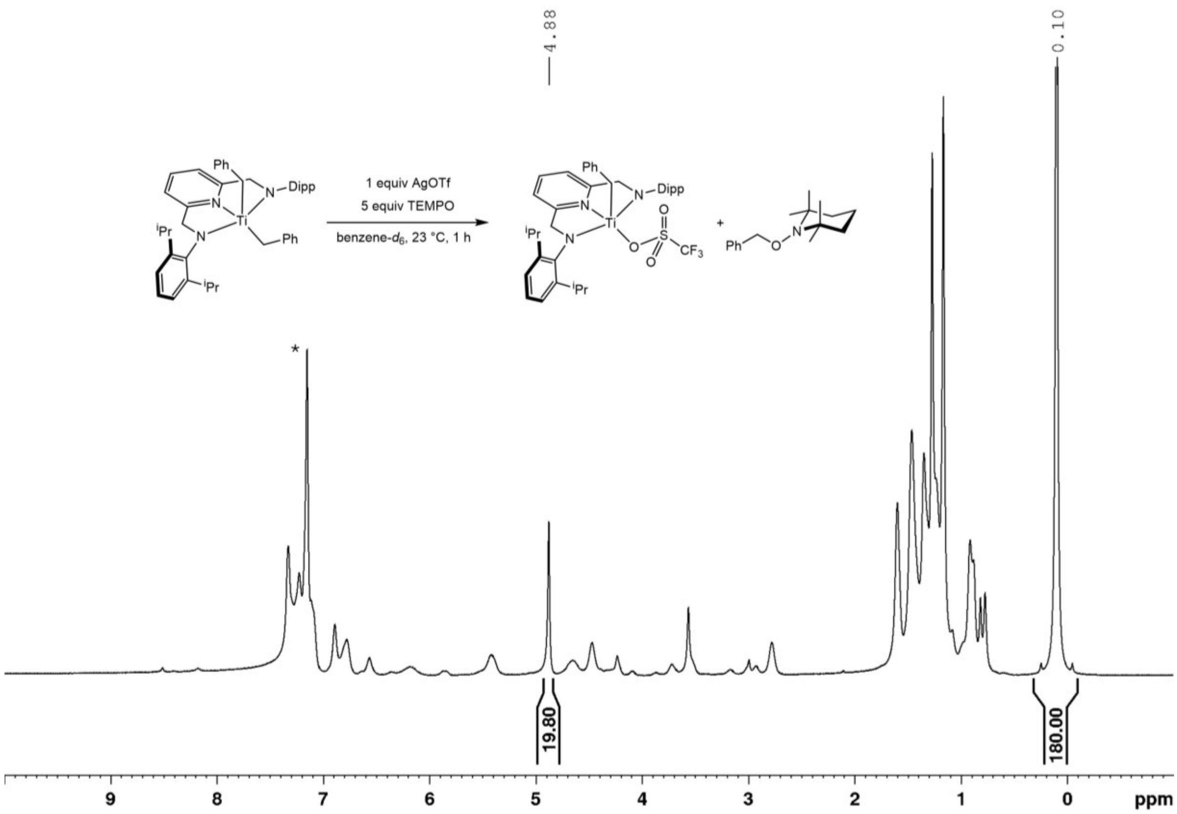

Figure S46. ^{1}H NMR spectrum (400 MHz, 298 K, benzene-d_6) of the oxidation of (^{iPr}PDA)Ti(CH_2Ph)$_2$ with AgOTf in the presence of 5 equivalents of TEMPO. The residual signal of deuterated solvent is marked with an asterisk (*).

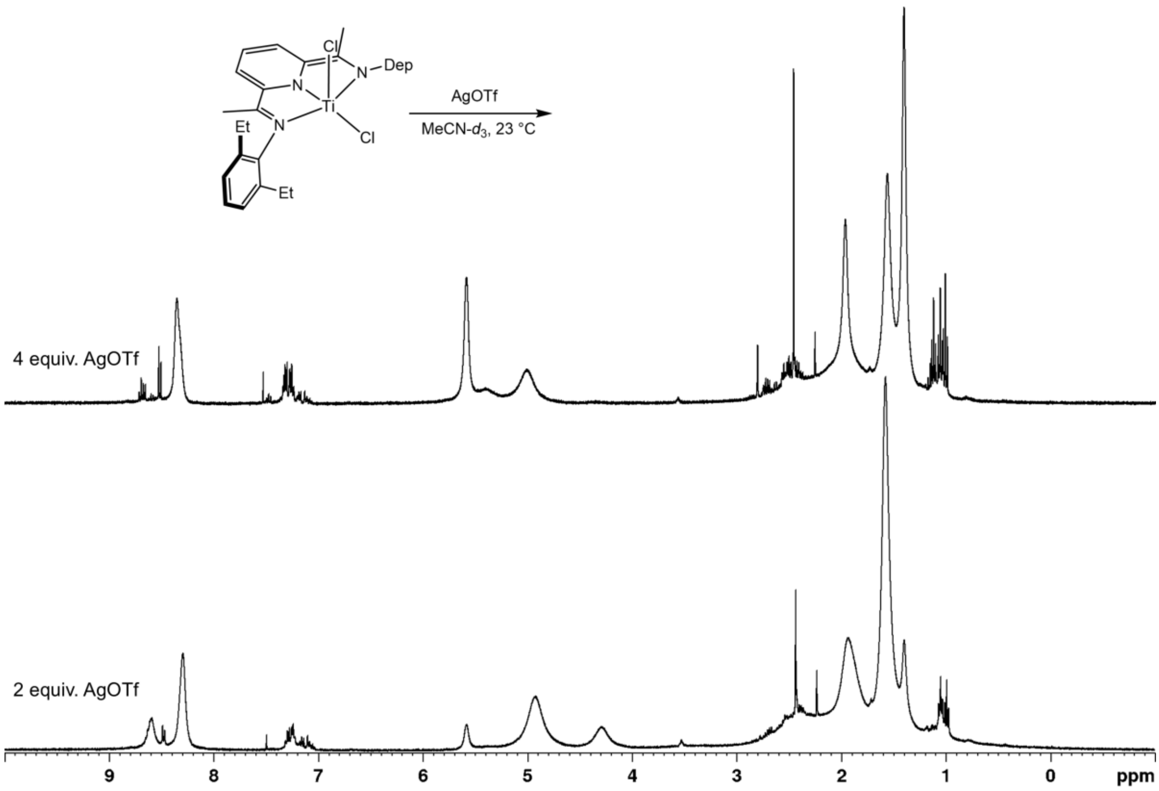

Figure S47. ^{1}H NMR spectra (400 MHz, 298 K, benzene-d_6) of the reaction of (^{Et}PDI)$TiCl_2$ with 2 and 4 equivalents of AgOTf in MeCN-d_3.

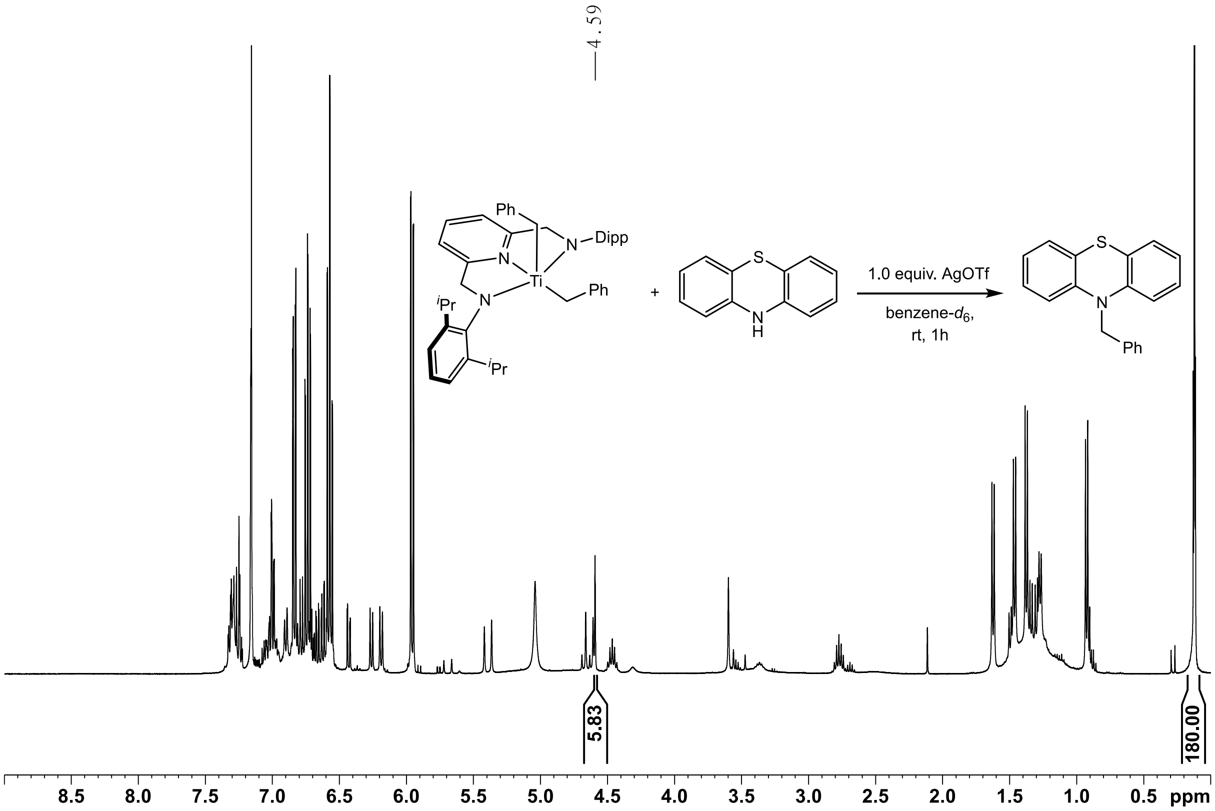

Figure S48. ^{1}H NMR spectrum (400 MHz, 298 K, benzene-d_6) of the trapping reaction with (^{iPr}PDA)Ti(CH_2Ph)$_2$ and phenothiazine.

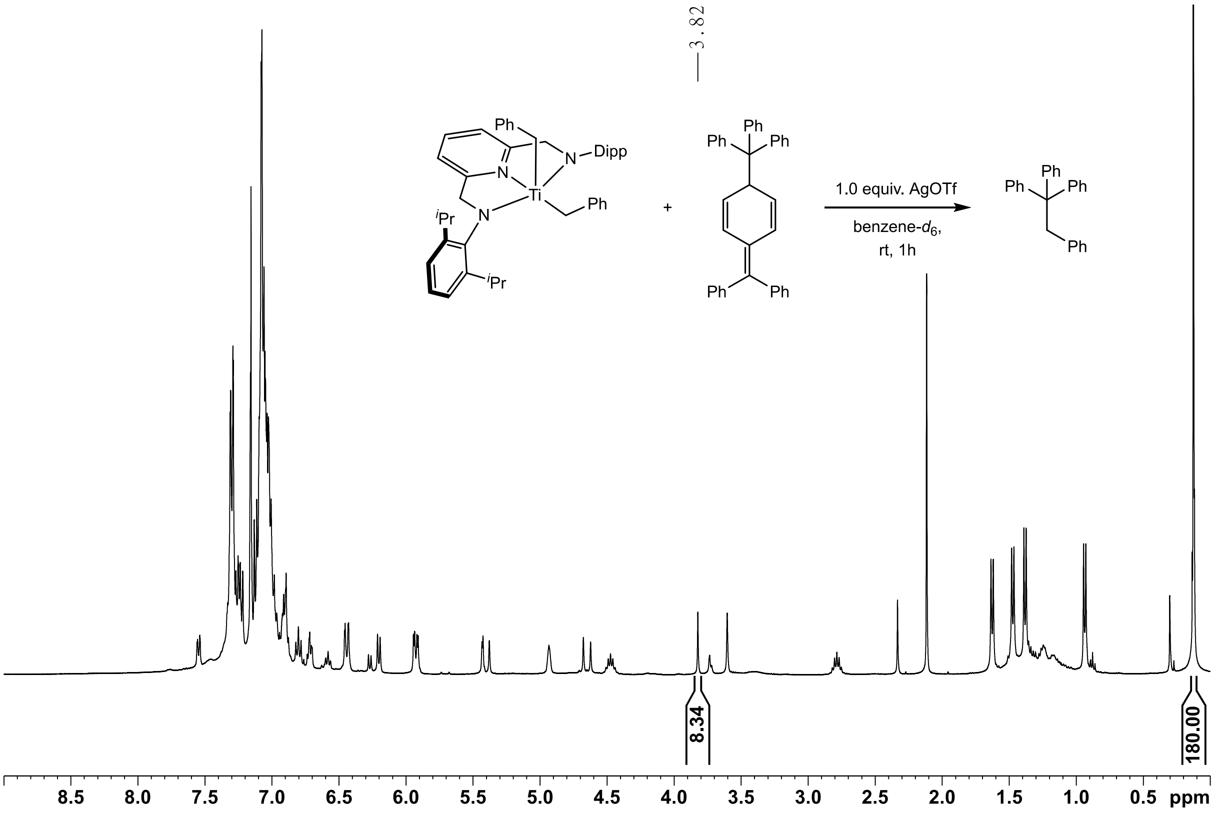

Figure S49. ^{1}H NMR spectrum (400 MHz, 298 K, benzene-d_6) of the trapping reaction with (^{iPr}PDA)Ti(CH_2Ph)$_2$ and Gomberg's dimer.

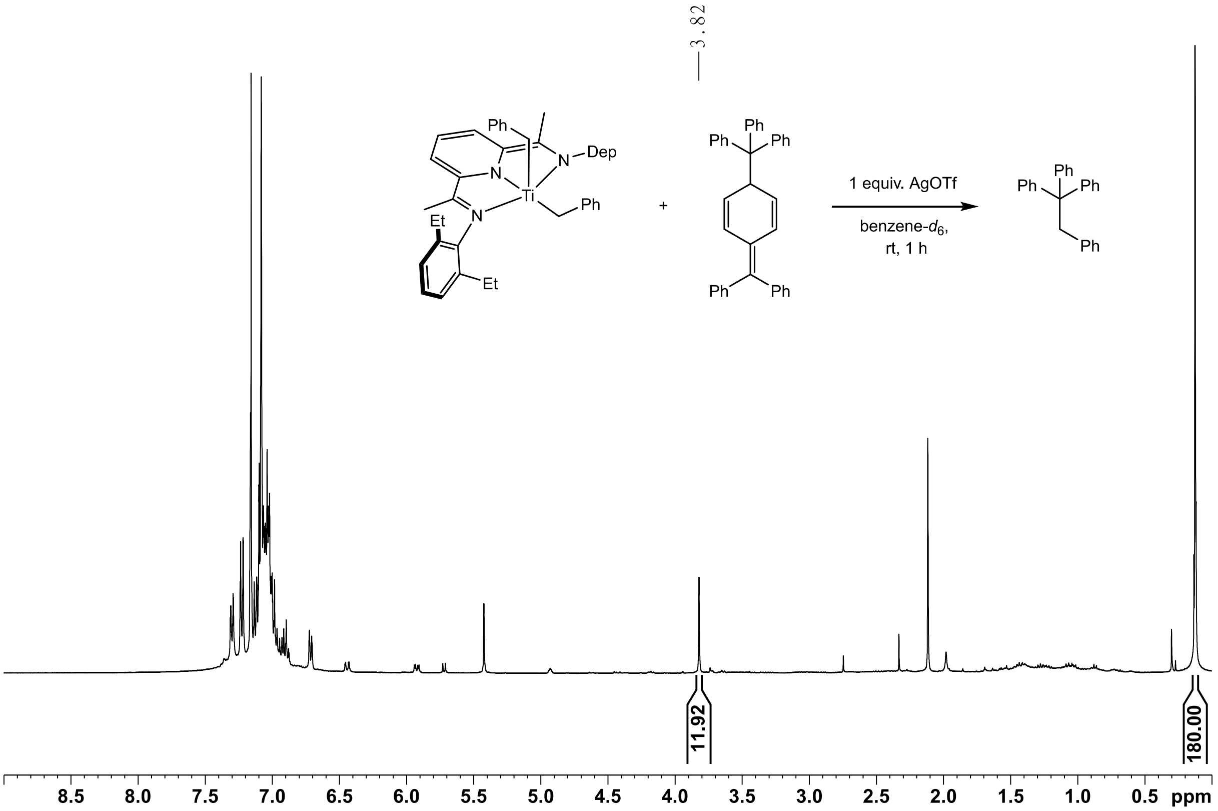

Figure S50. ^{1}H NMR spectrum (400 MHz, 298 K, benzene-d_6) of the trapping reaction with (^{Et}PDT)Ti(CH_2Ph)$_2$ and Gomberg's dimer.

6. EPR spectra of trapping reactions

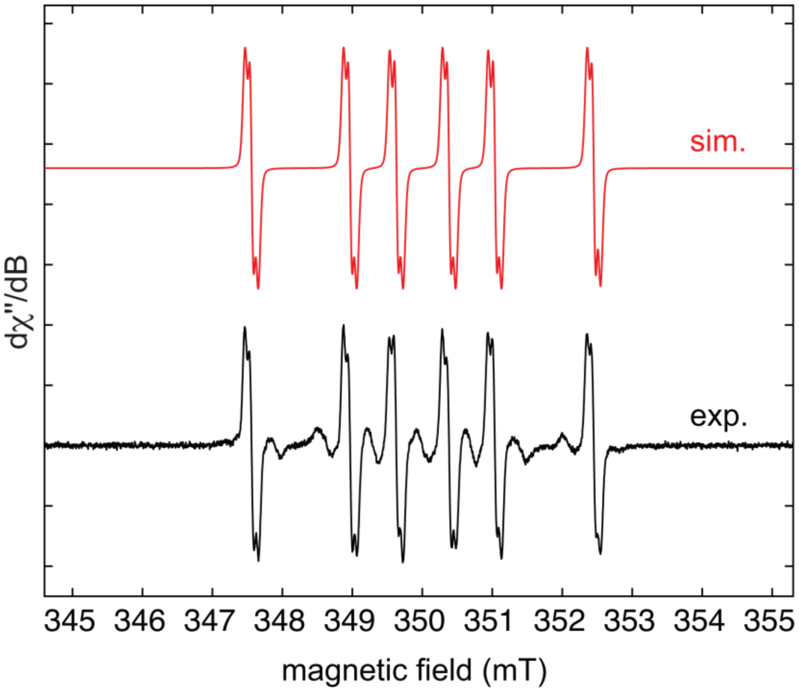

Figure S51. Experimental (black) and simulated (red) continuous-wave (CW) X-band EPR spectra of the reaction of (^{iPr}PDA)Ti(CH_2Ph)$_2$ with AgOTf in the presence of DMPO (8.5 equiv.) in benzene at ambient temperature. The best-fit simulation parameters are: g_{iso} = 2.0061, a(^{14}N) = 39.6 MHz (14.1 G), a(^{1}H) = 58.1 MHz (20.7 G), and a(^{1}H, 2H) = 1.9 MHz (0.7 G).

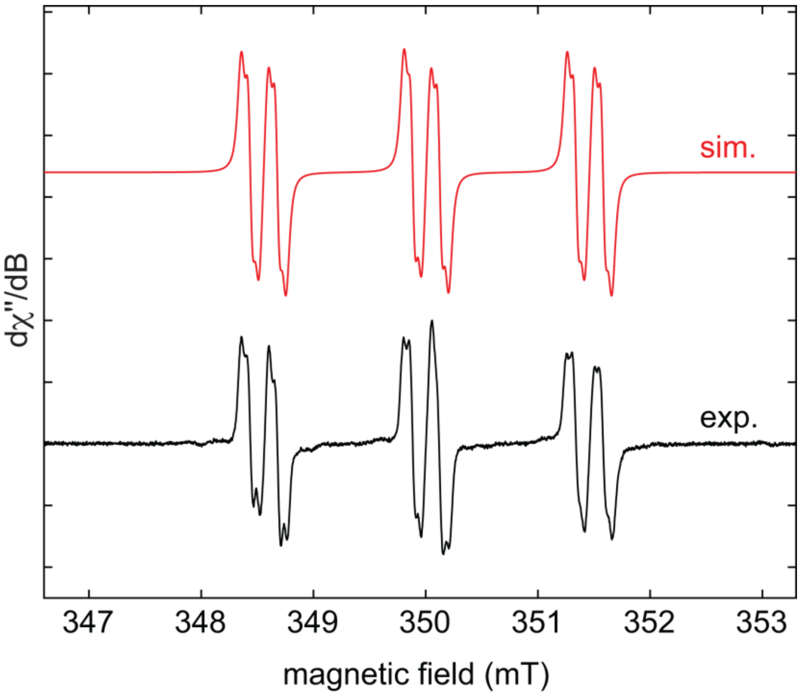

Figure S52. Experimental (black) and simulated (red) continuous-wave (CW) X-band EPR spectra of the reaction of (^{iPr}PDA)Ti(CH_2Ph)$_2$ with AgOTf in the presence of PBN (8.5 equiv.) in benzene at ambient temperature. The best-fit simulation parameters are: g_{iso} = 2.0063, a(^{14}N) = 40.8 MHz (14.5 G), a(^{1}H) = 6.8 MHz (2.4 G), and a(^{1}H, 2H) = 1.6 MHz (0.6 G).

7. GC and MS Data

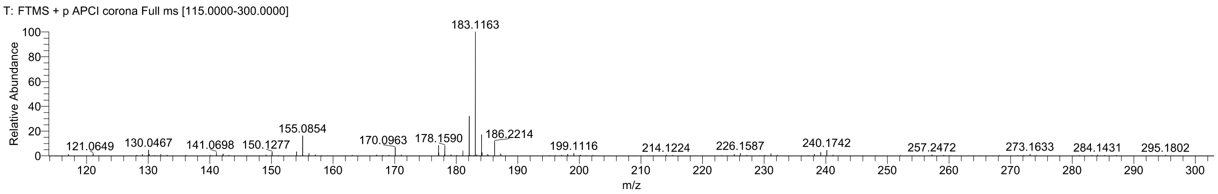

Figure S53. HRMS spectrum of the cross-over experiment of (^{Et}PDI)TiPh$_2$ and (^{Et}PDI)Ti(*p*-Tol)$_2$ with [C_7H_7][BAr^F$_4$]. Calculated for [$C_{12}H_{10}$ + H]$^+$: 155.0855 m/z; [$C_{13}H_{12}$ + H]$^+$: 169.1012 m/z; [$C_{14}H_{14}$ + H]$^+$: 183.1168 m/z.

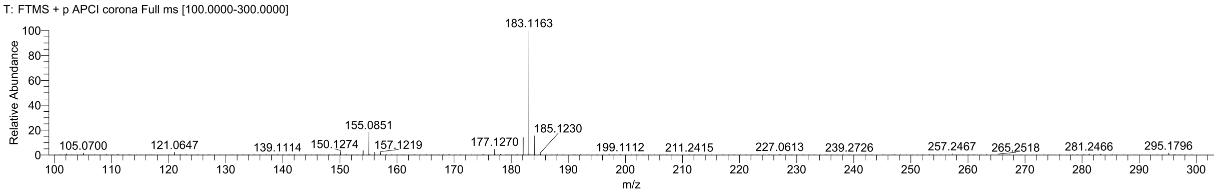

Figure S54. HRMS spectrum of the cross-over experiment of (^{Et}PDI)TiPh$_2$ and (^{Et}PDI)Ti(*p*-Tol)$_2$ with I_2. Calculated for [$C_{12}H_{10}$ + H]$^+$: 155.0855 m/z; [$C_{13}H_{12}$ + H]$^+$: 169.1012 m/z; [$C_{14}H_{14}$ + H]$^+$: 183.1168 m/z.

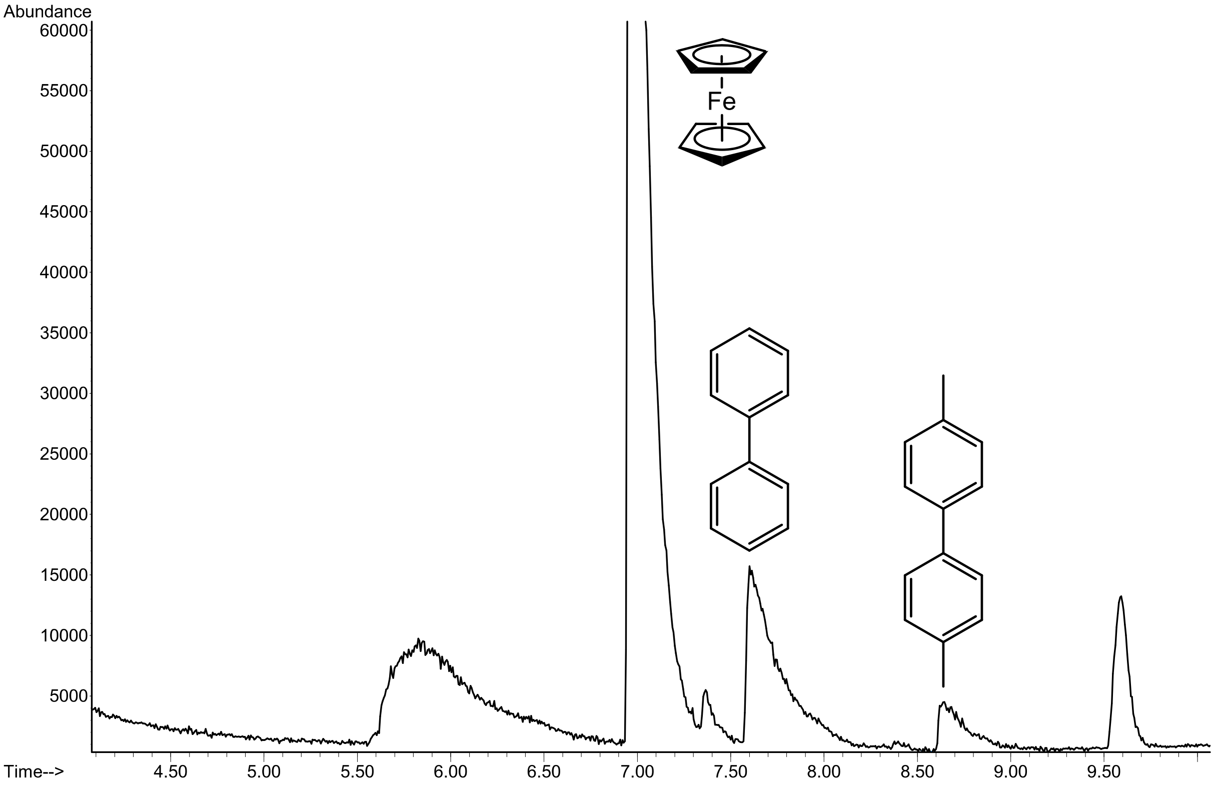

Figure S55. GCMS elugram of the cross-over experiment of (^{Et}PDI)TiPh$_2$ and (^{Et}PDI)Ti(*p*-Tol)$_2$ with [Fc][BAr^F$_4$].

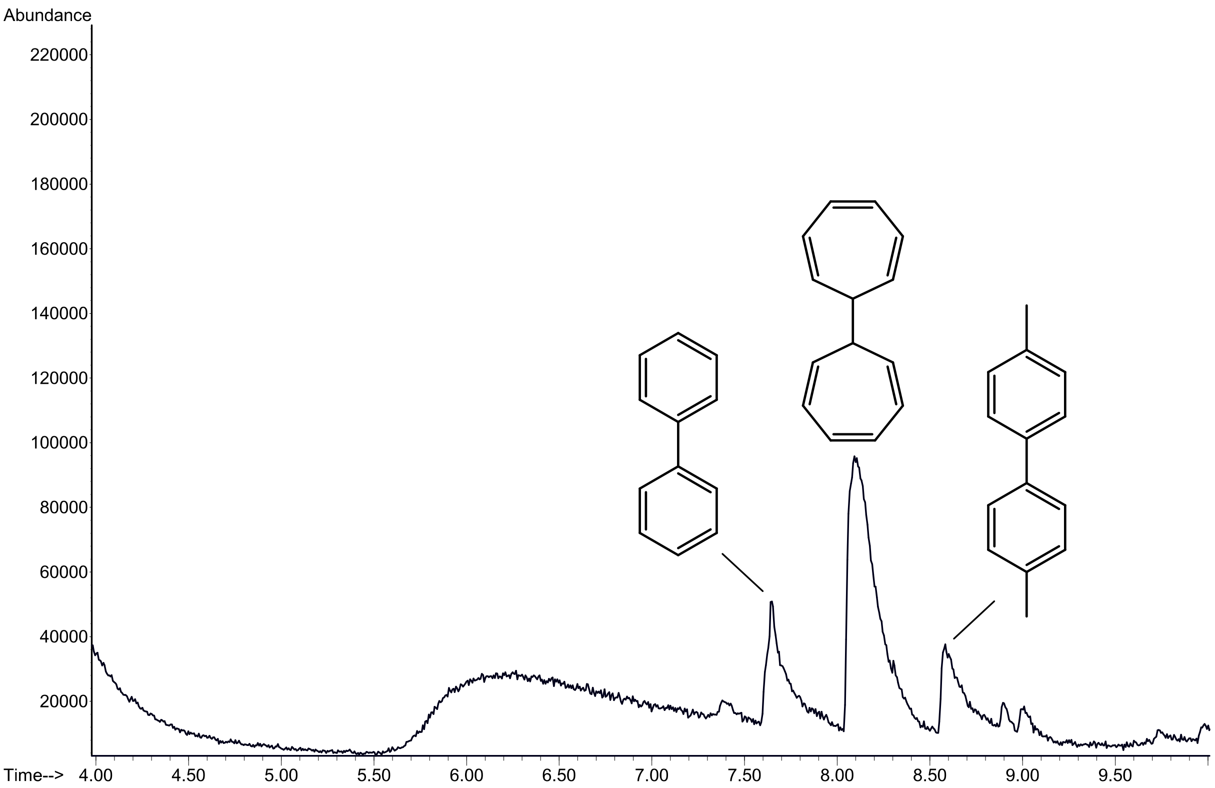

Figure S56. GCMS elugram of the cross-over experiment of (^{Et}PDI)TiPh$_2$ and (^{Et}PDI)Ti(*p*-Tol)$_2$ with [C_7H_7][BAr$^F{}_4$].

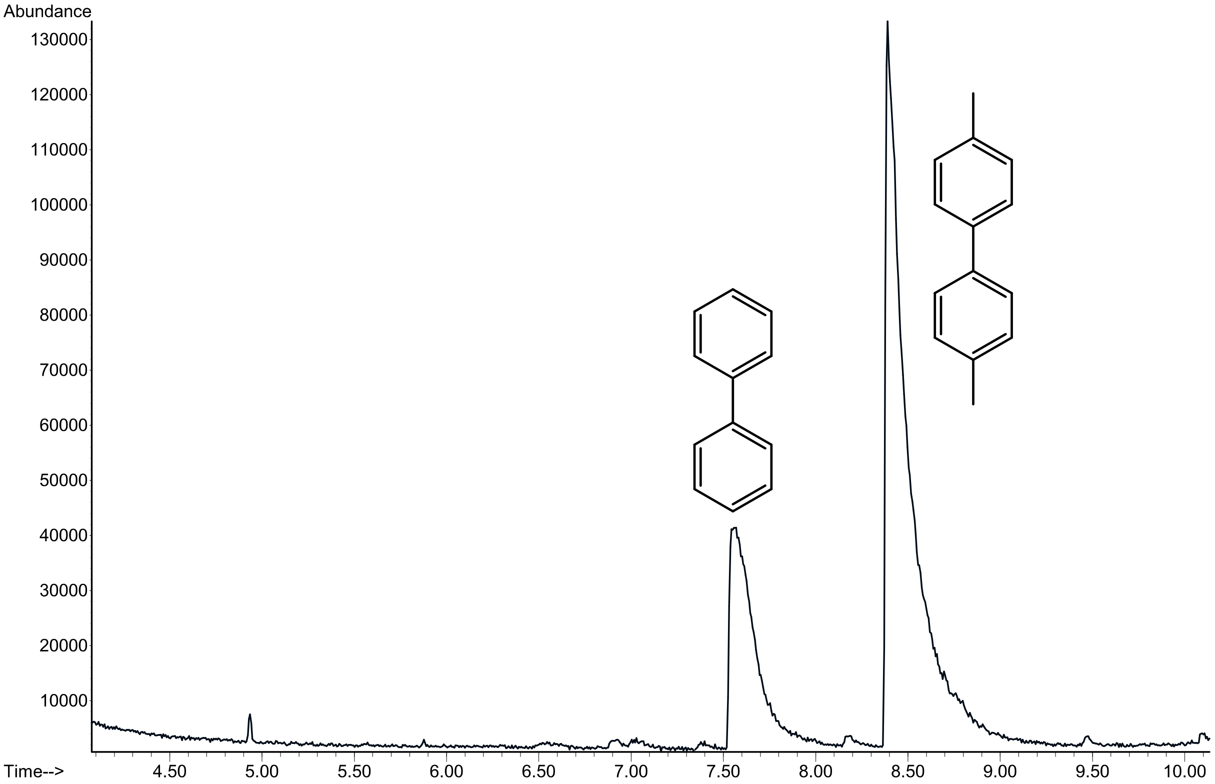

Figure S57. GCMS elugram of the cross-over experiment of (^{Et}PDI)TiPh$_2$ and (^{Et}PDI)Ti(*p*-Tol)$_2$ with I_2.

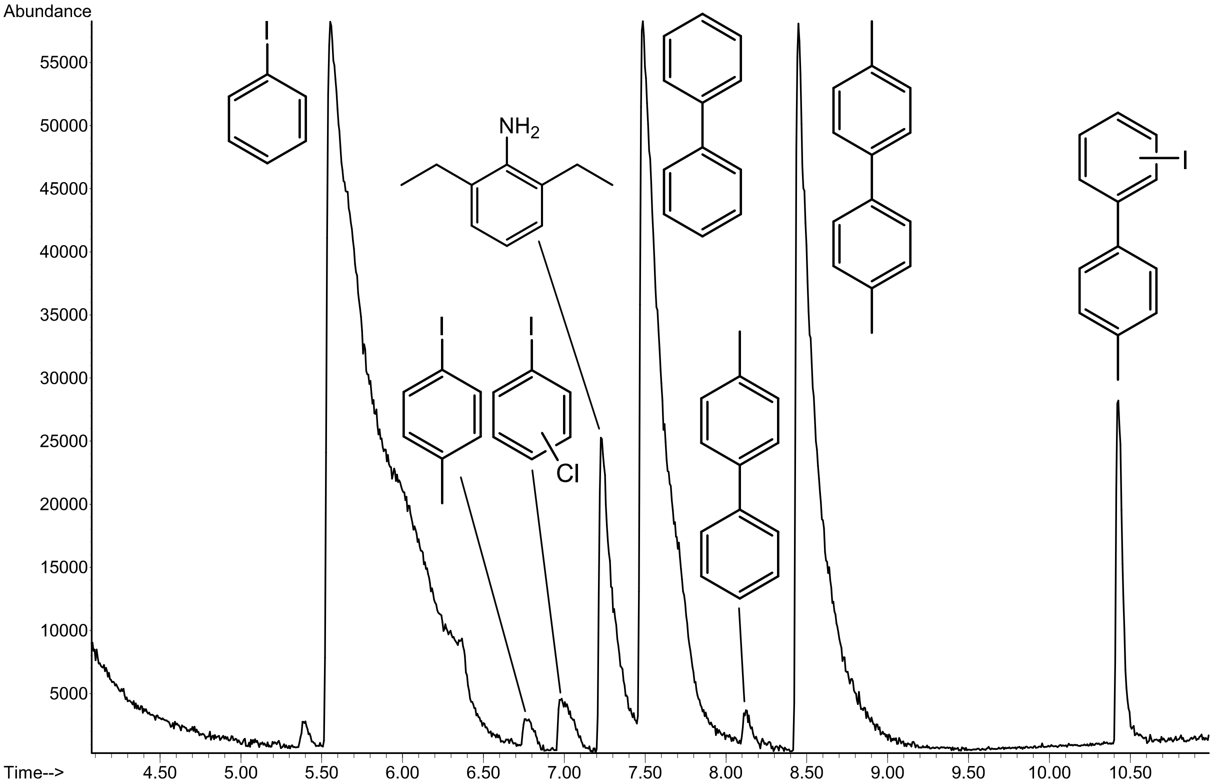

Figure S58. GCMS elugram of the cross-over experiment of (^{Et}PDI)TiPh$_2$ and (^{Et}PDI)Ti(*p*-Tol)$_2$ with PhICl$_2$.

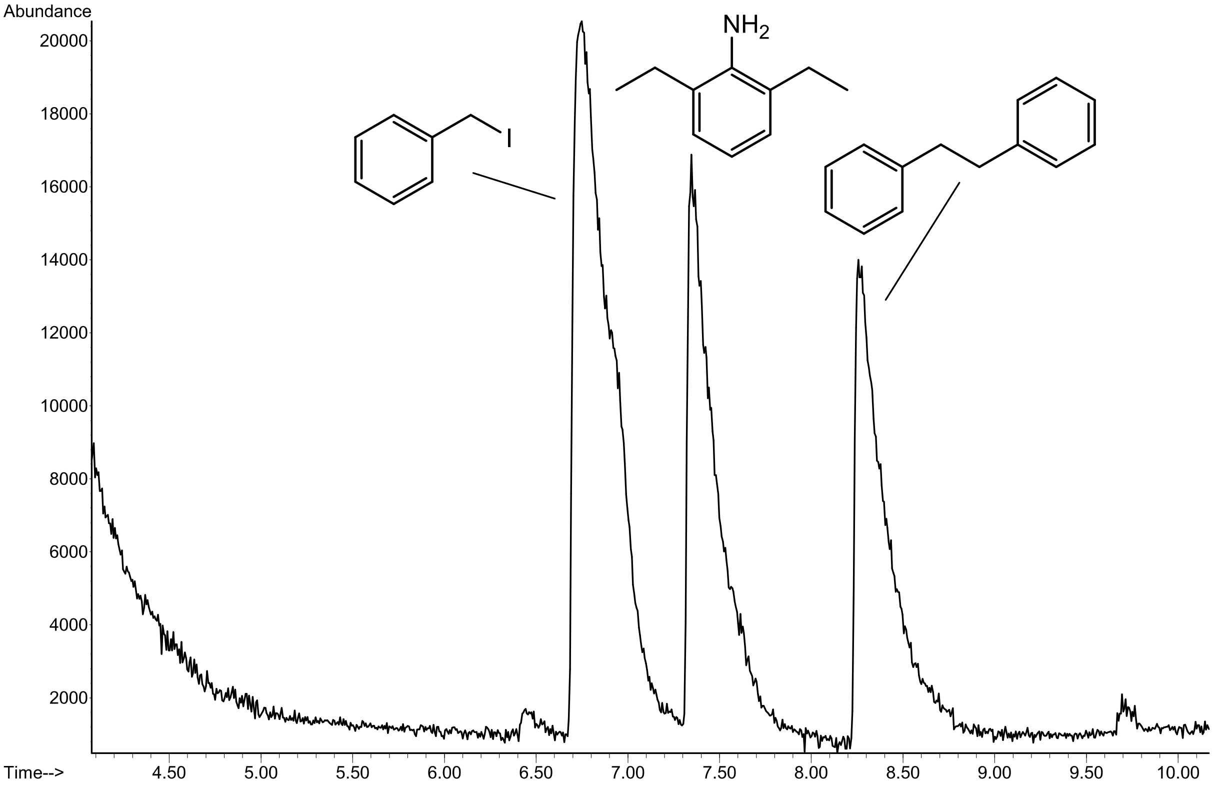

Figure S59. GCMS elugram of the oxidation reaction of (^{Et}PDI)Ti(CH$_2$Ph)$_2$ with I$_2$.

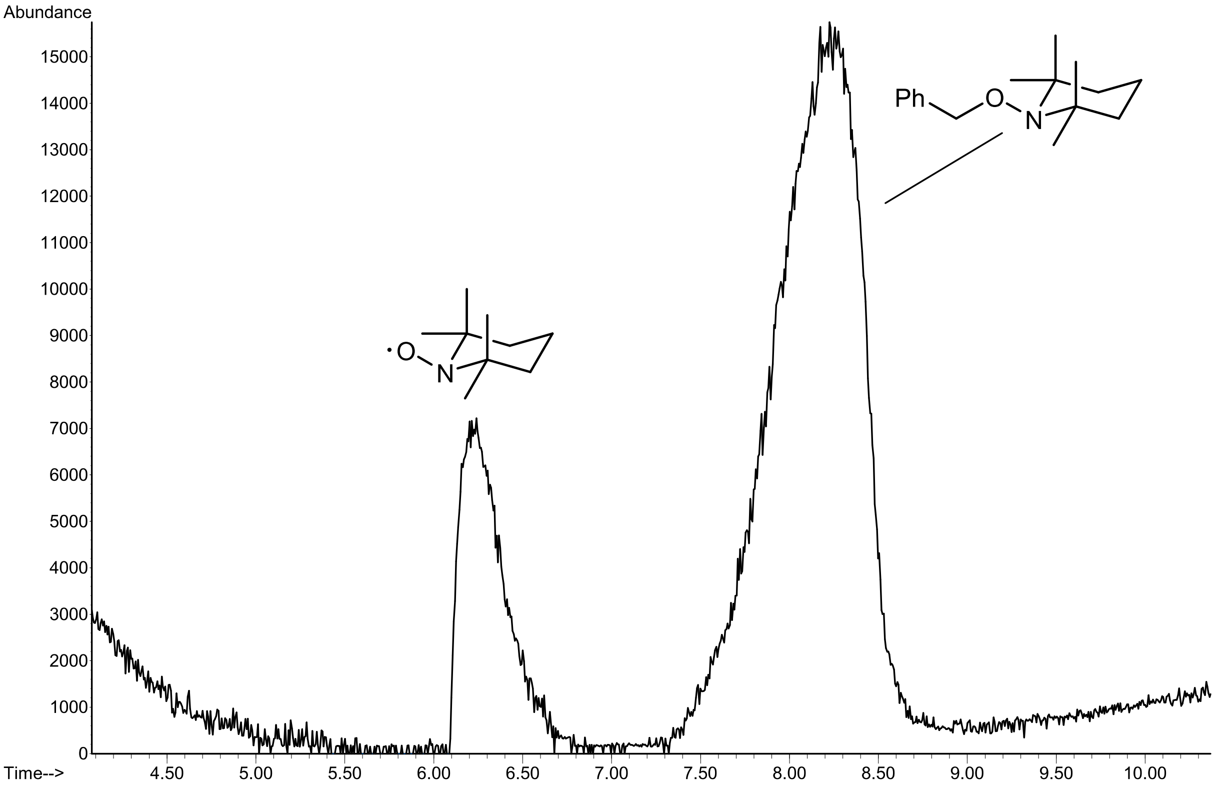

Figure S60. GCMS elugram of the trapping reaction of (^{Et}PDI)Ti($CH_2Ph)_2$ with TEMPO.

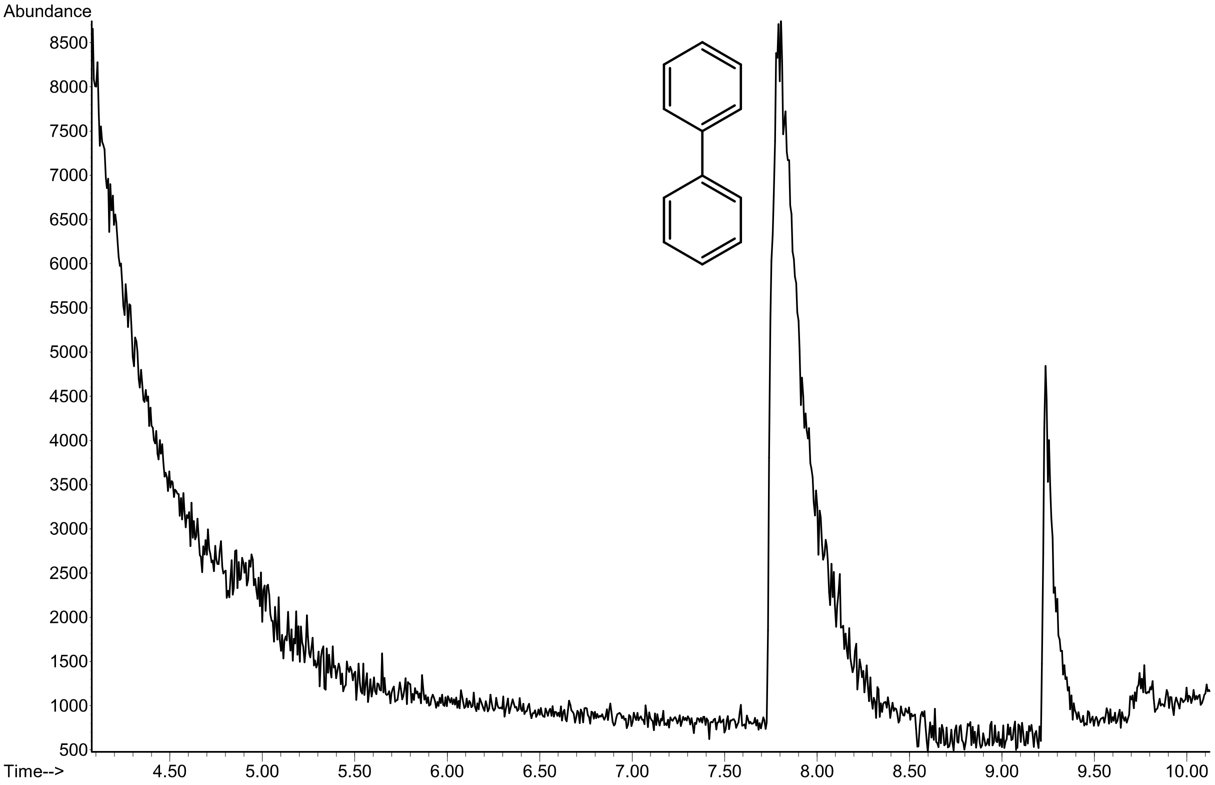

Figure S61. GCMS elugram of the oxidation reaction of (^{iPr}PDA)TiPh$_2$ with AgOTf.

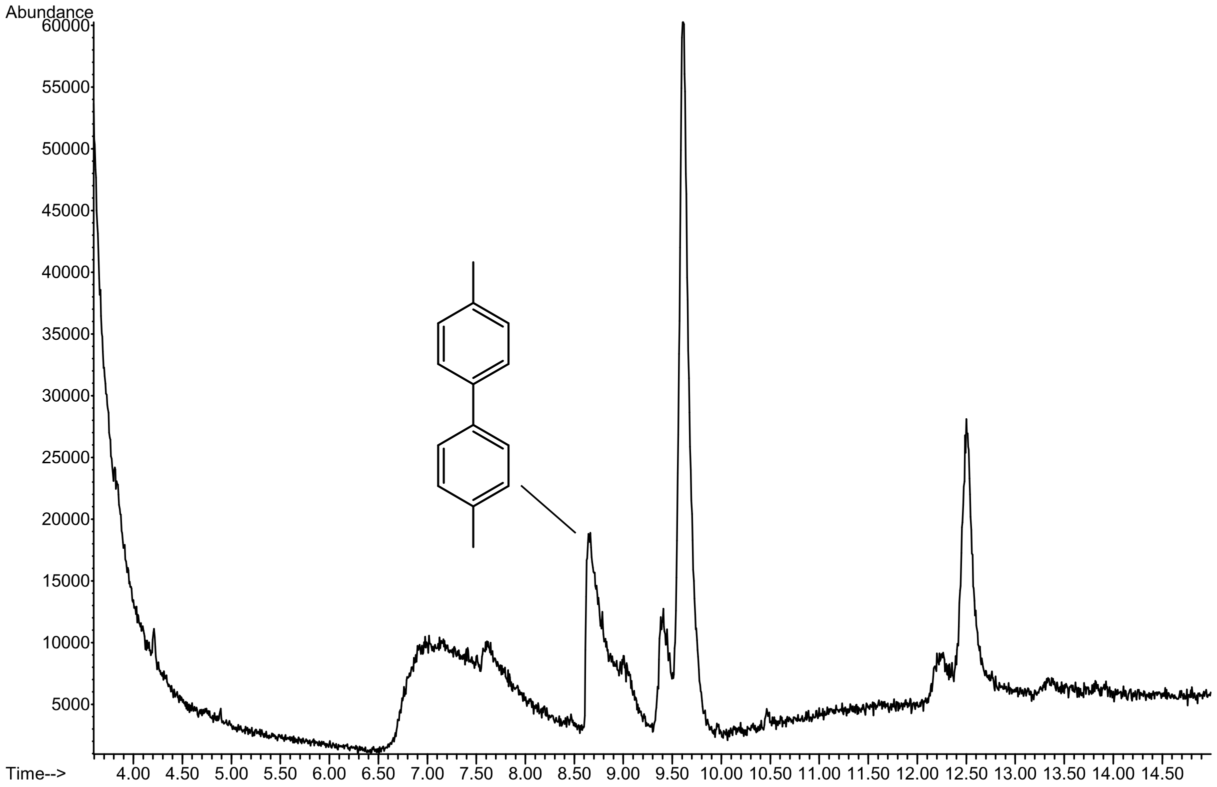

Figure S62. GCMS elugram of the electrochemical oxidation of (^{Et}PDI)Ti(p-Tol)$_2$.

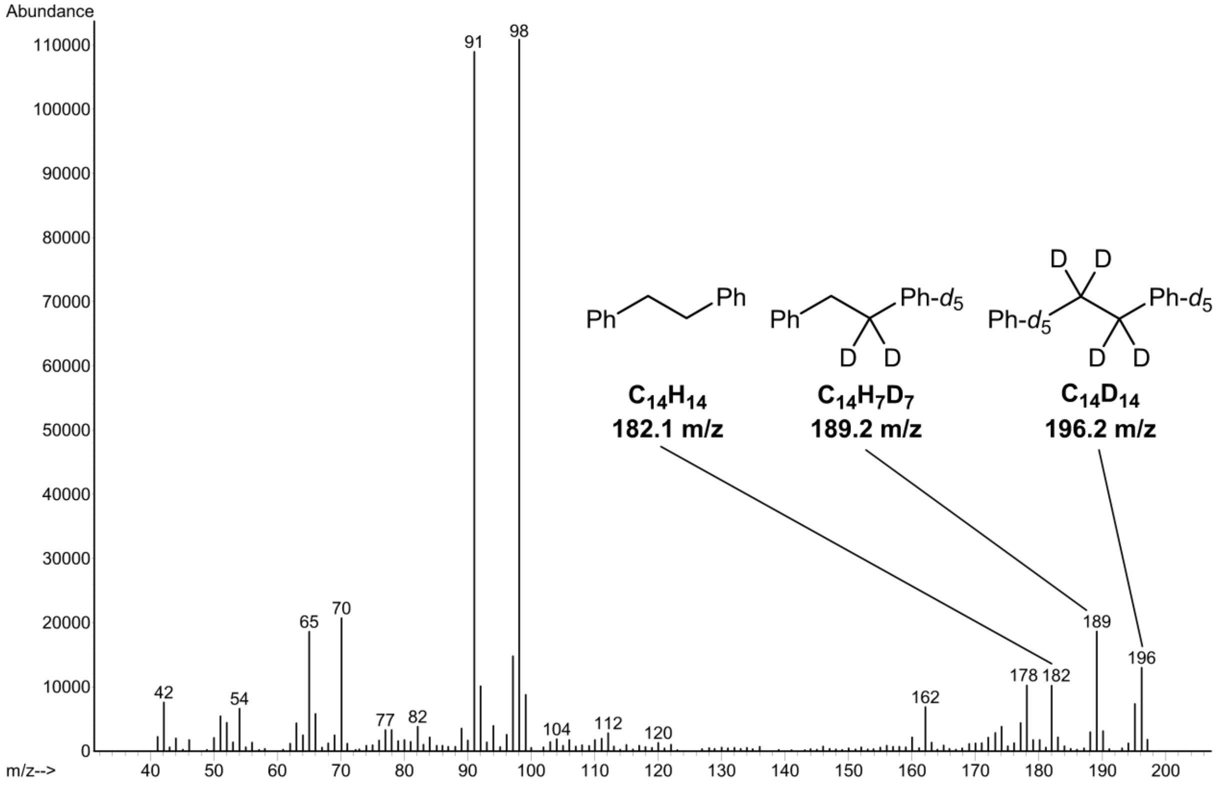

Figure S63. Mass spectrum of the cross-over experiment of (^{iPr}PDA)Ti(CH_2Ph)$_2$ and (^{iPr}PDA)Ti(CD_2Ph-d_5)$_2$ and AgOTf observed at an elution time of 7.94 min.

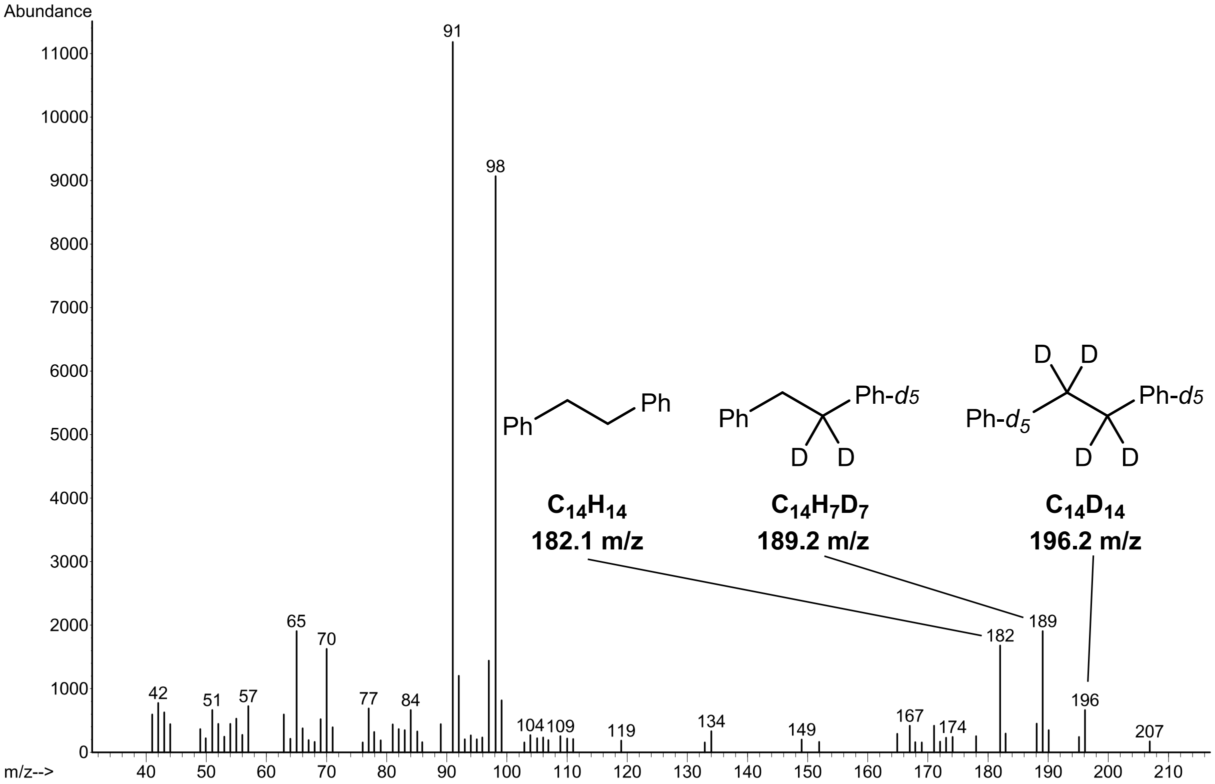

Figure S64. Mass spectrum of the cross-over experiment of (^{iPr}PDA)Ti(CH_2Ph)$_2$ and (^{iPr}PDA)Ti(CD_2Ph-d_5)$_2$ and AgOTf observed at an elution time of 8.22 min.

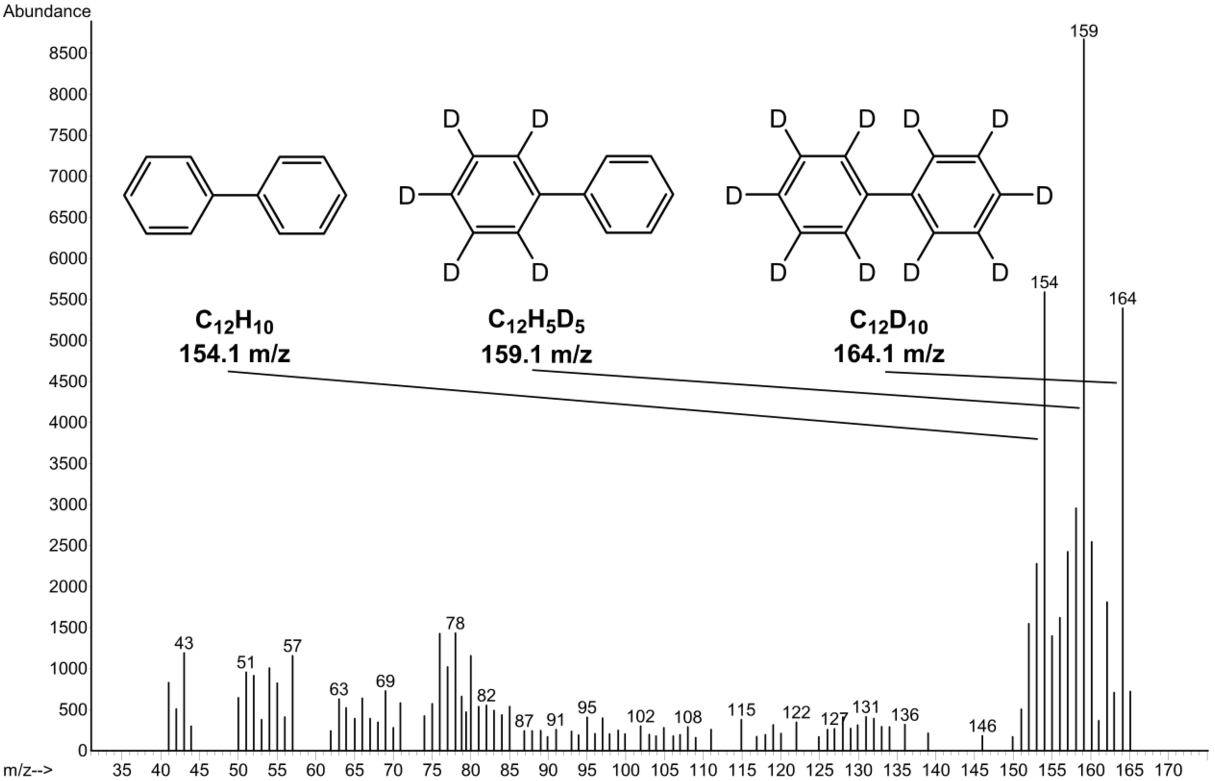

Figure S65. Mass spectrum of the cross-over experiment of (^{iPr}PDA)TiPh$_2$ and (^{iPr}PDA)Ti(Ph-d_5)$_2$ and AgOTf observed at an elution time of 7.53 min.

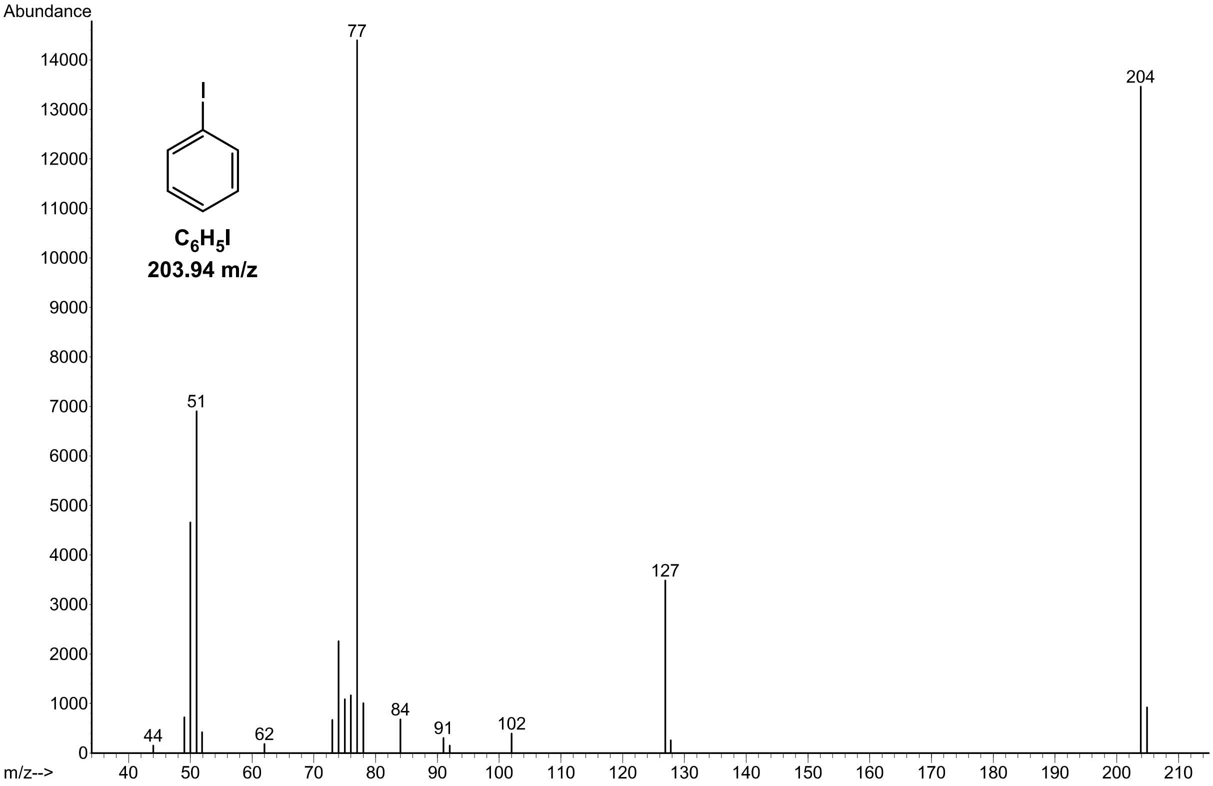

Figure S66. Mass spectrum of iodobenzene observed at an elution time of 5.58 min.

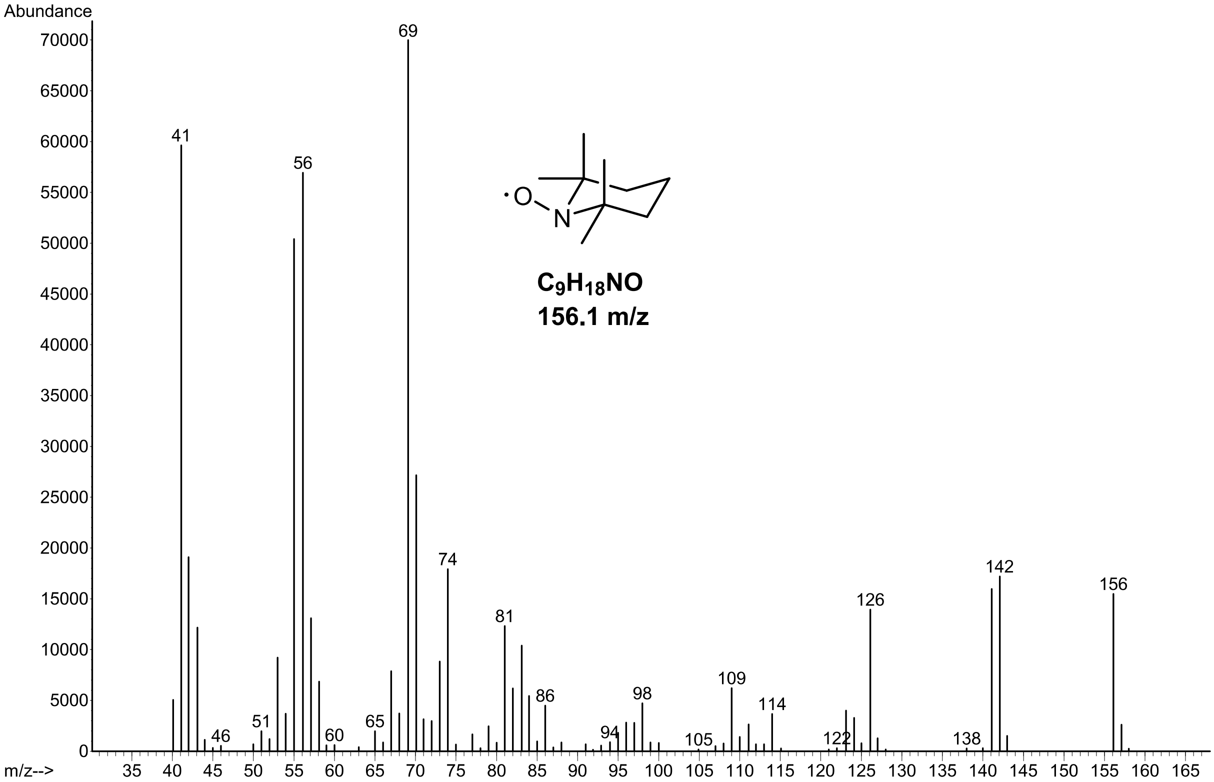

Figure S67. Mass spectrum of TEMPO observed at an elution time of 5.92 min.

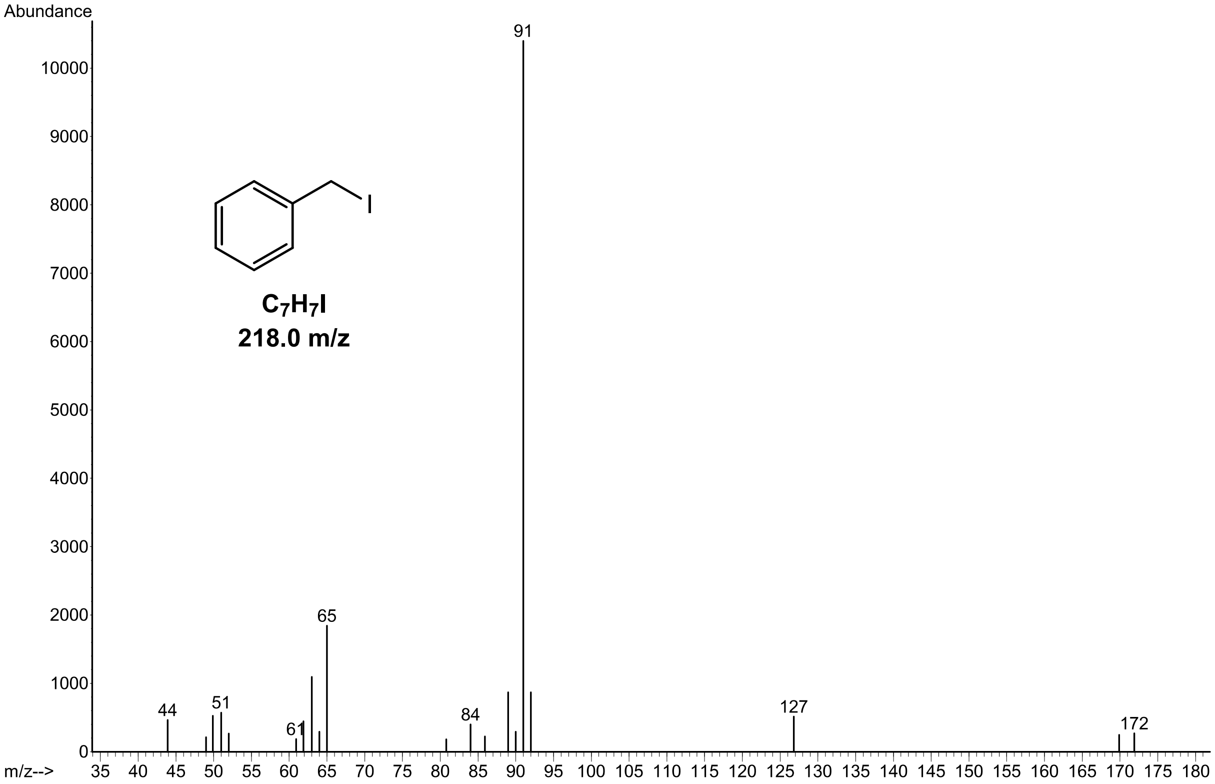

Figure S68. Mass spectrum of iodobenzene observed at an elution time of 6.74 min.

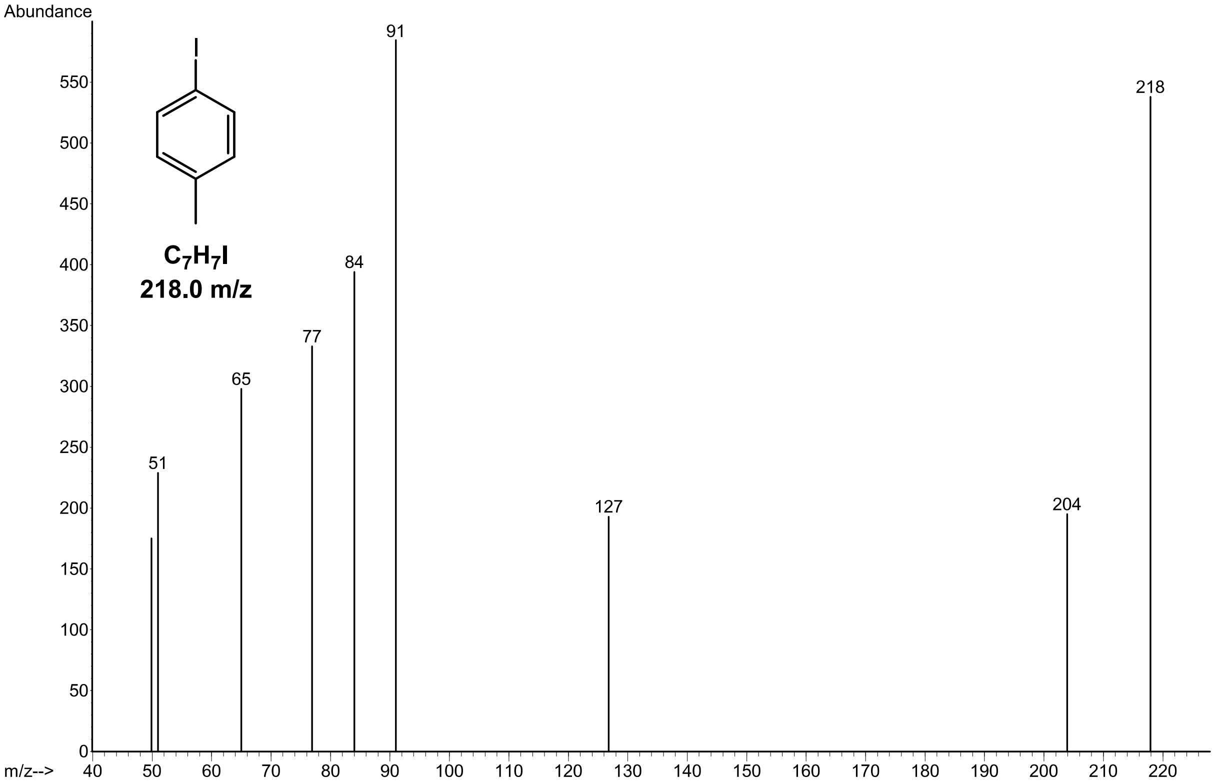

Figure S69. Mass spectrum of *para*-iodotoluene observed at an elution time of 6.78 min.

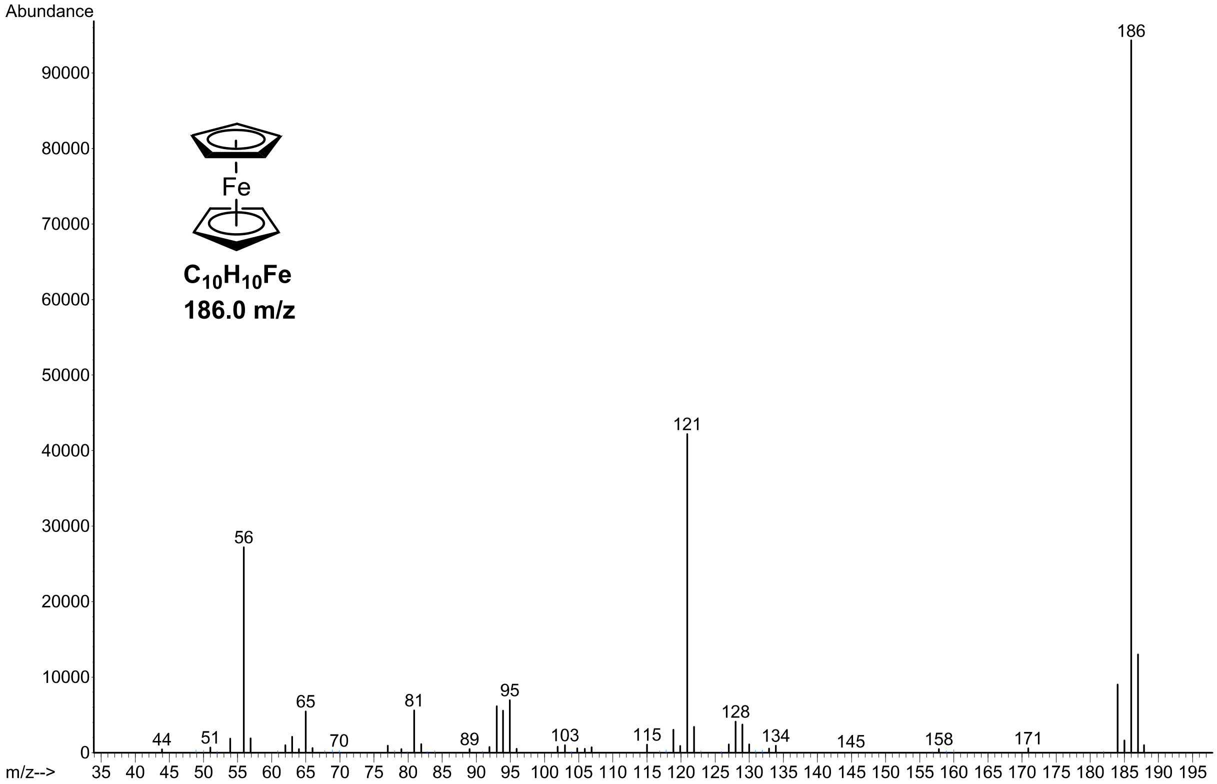

Figure S70. Mass spectrum of ferrocene observed at an elution time of 6.98 min.

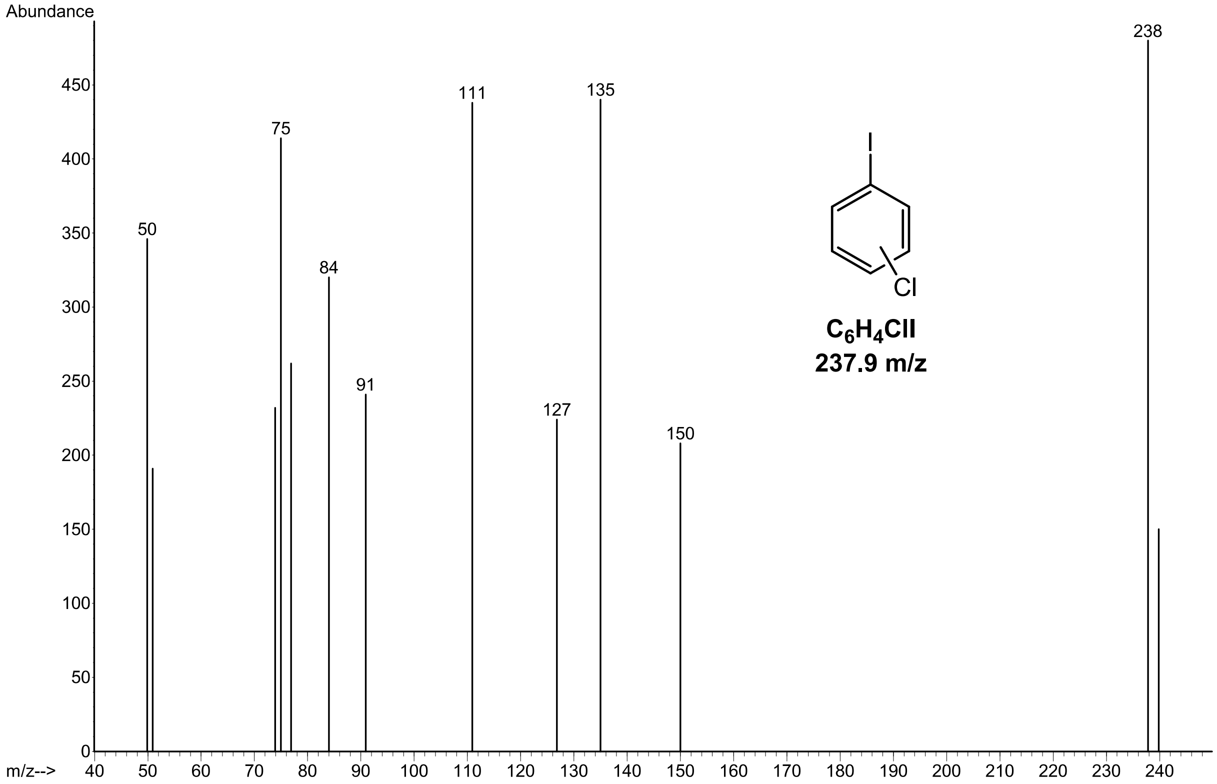

Figure S71. Mass spectrum of chloro iodobenzene observed at an elution time of 7.01 min.

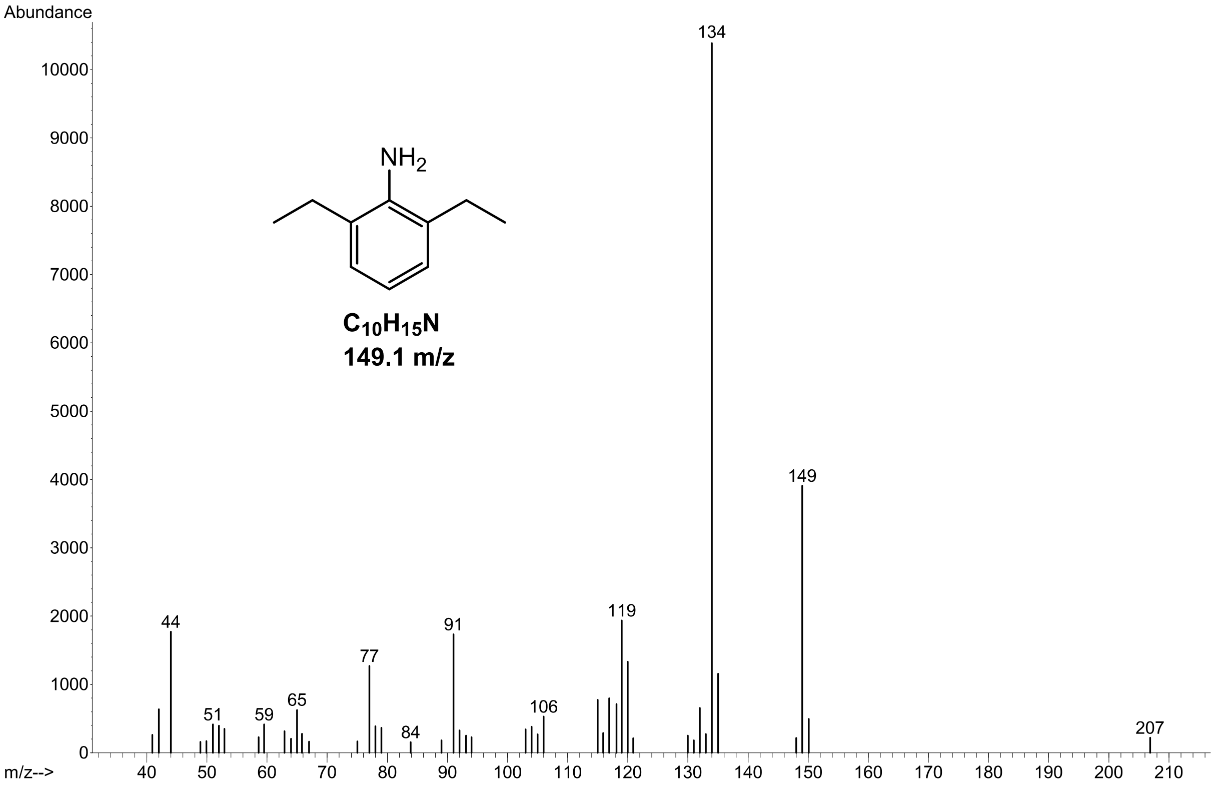

Figure S72. Mass spectrum of 2,6-diethylaniline observed at an elution time of 7.43 min.

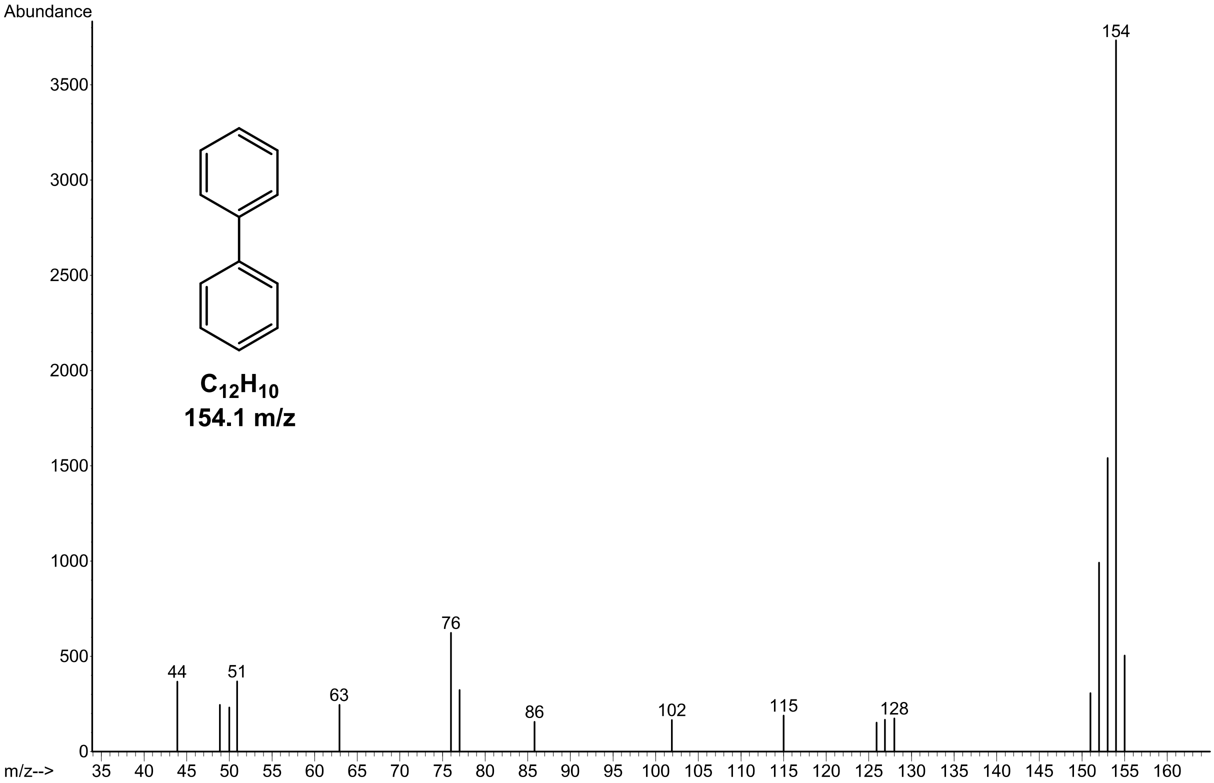

Figure S73. Mass spectrum of biphenyl observed at an elution time of 7.76 min.

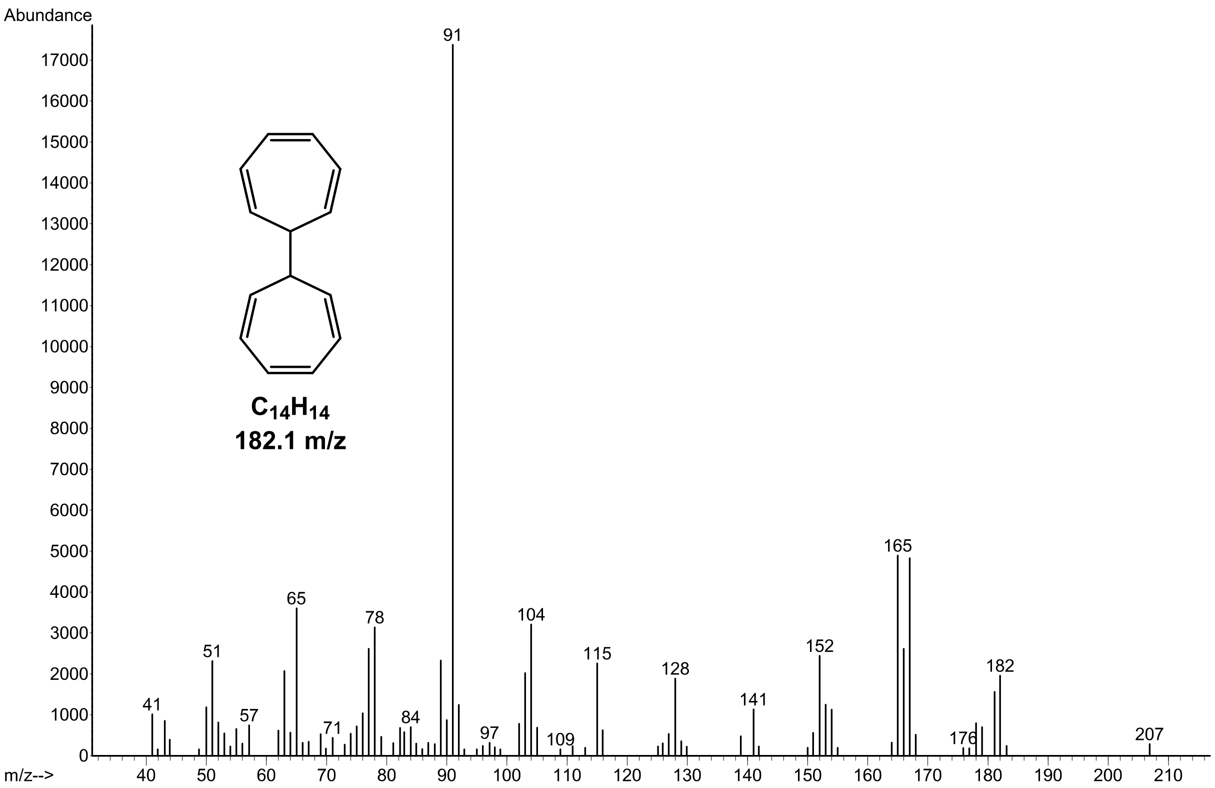

Figure S74. Mass spectrum of bitropyl observed at an elution time of 8.10 min.

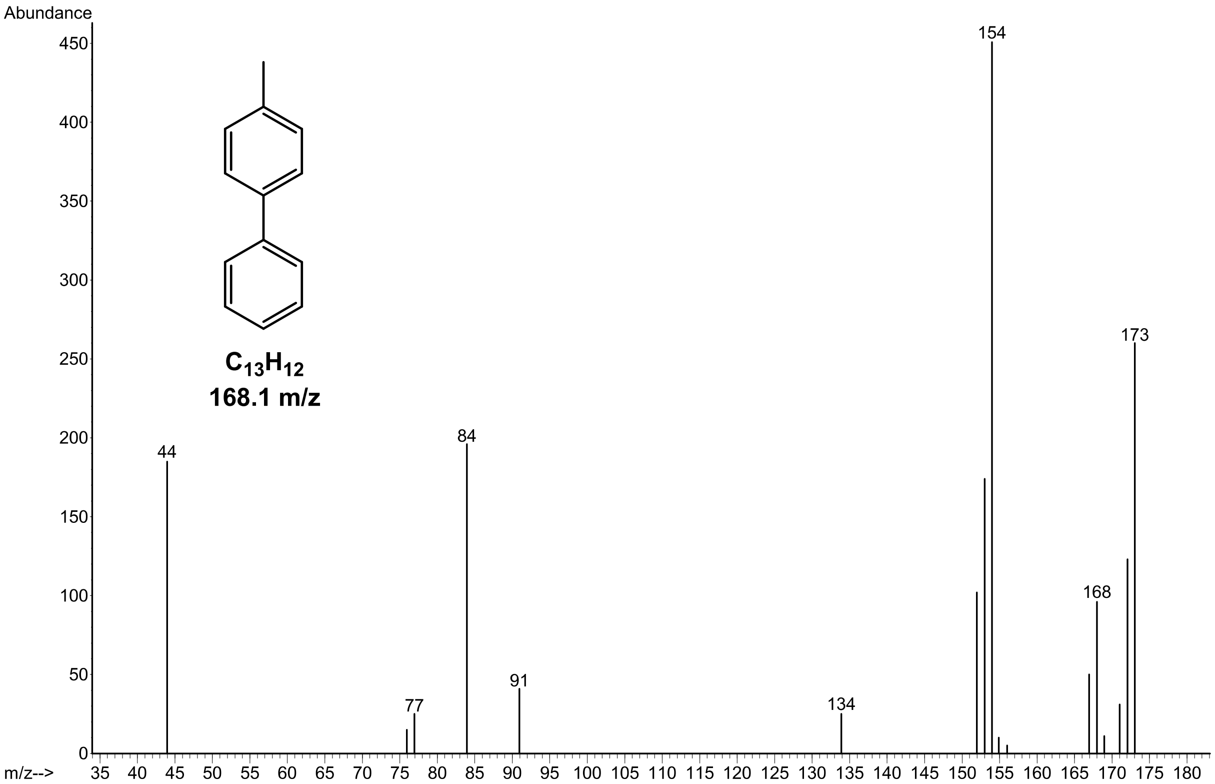

Figure S75. Mass spectrum of 4-methylbiphenyl observed at an elution time of 8.14 min.

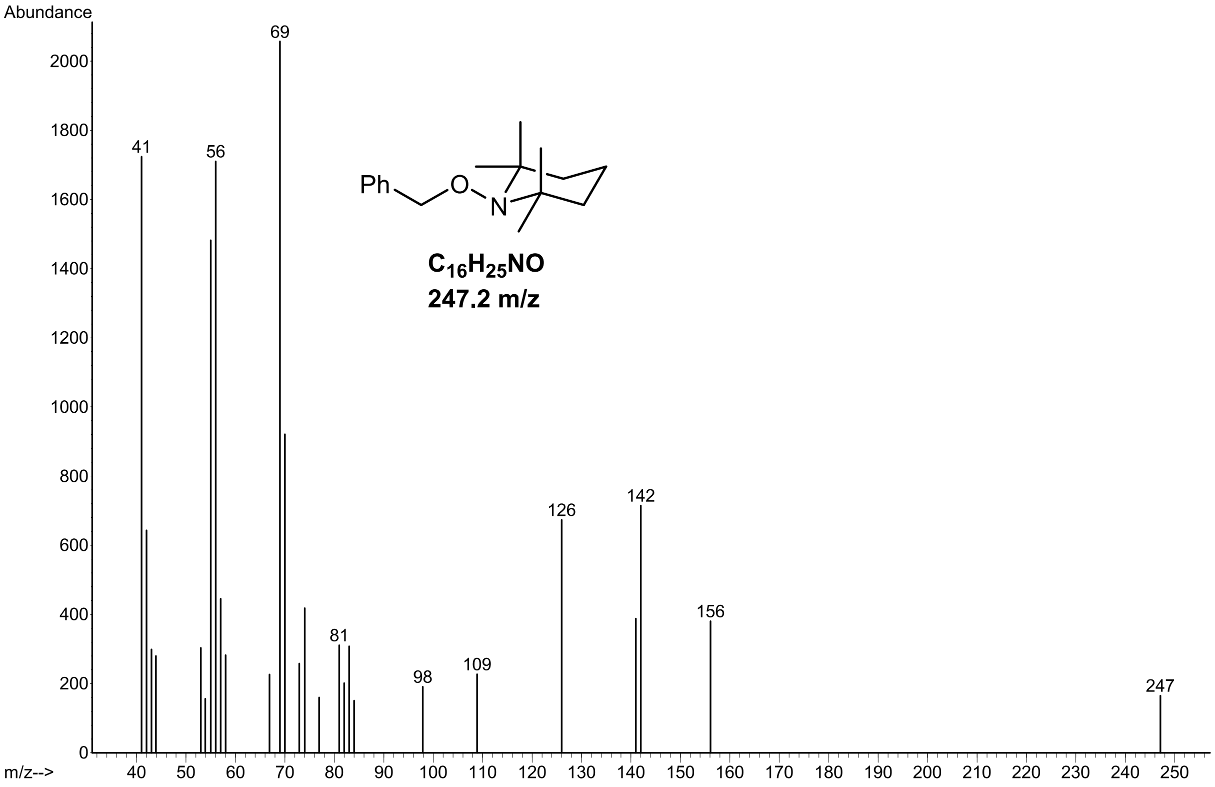

Figure S76. Mass spectrum of TEMPO−CH_2Ph observed at an elution time of 8.19 min.

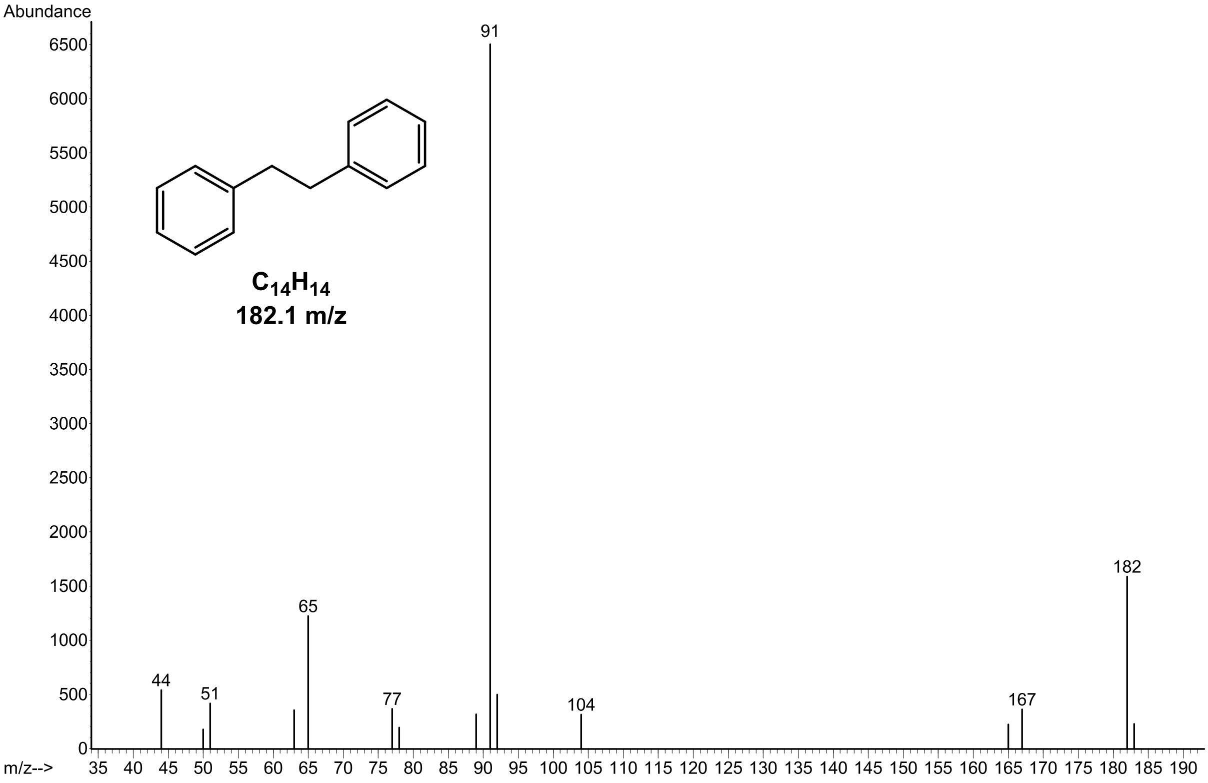

Figure S77. Mass spectrum of bibenzyl observed at an elution time of 8.26 min.

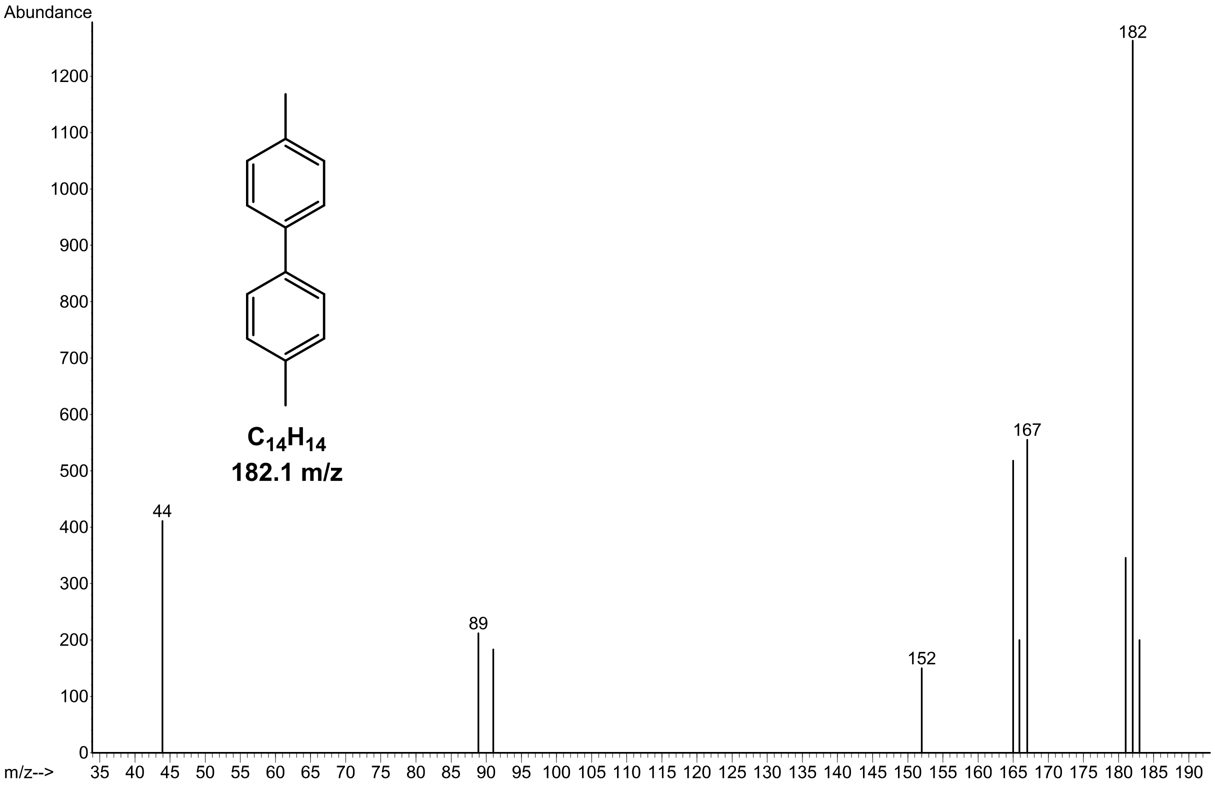

Figure S78. Mass spectrum of bitolyl observed at an elution time of 8.68 min.

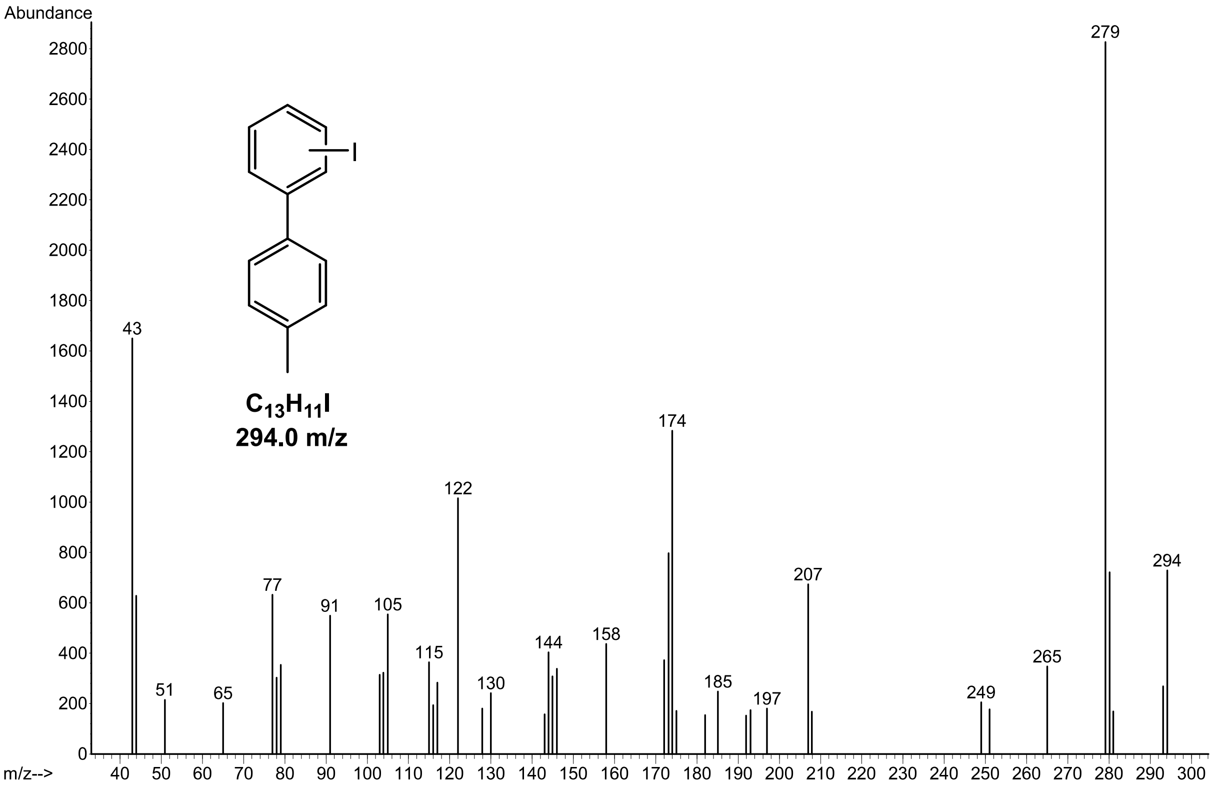

Figure S79. Mass spectrum of iodated methylbiphenyl observed at an elution time of 10.45 min.

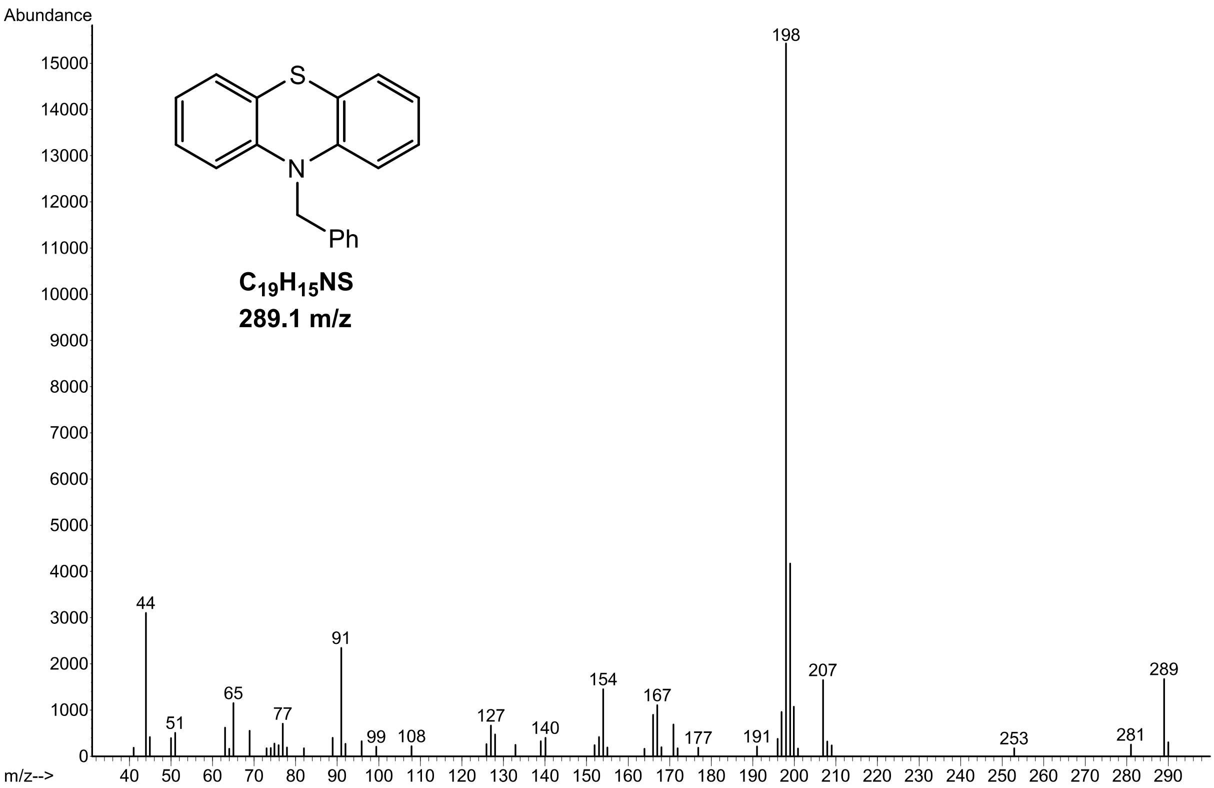

Figure S80. Mass spectrum of 10-benzyl-10H-phenothiazine observed at an elution time of 12.45 min.

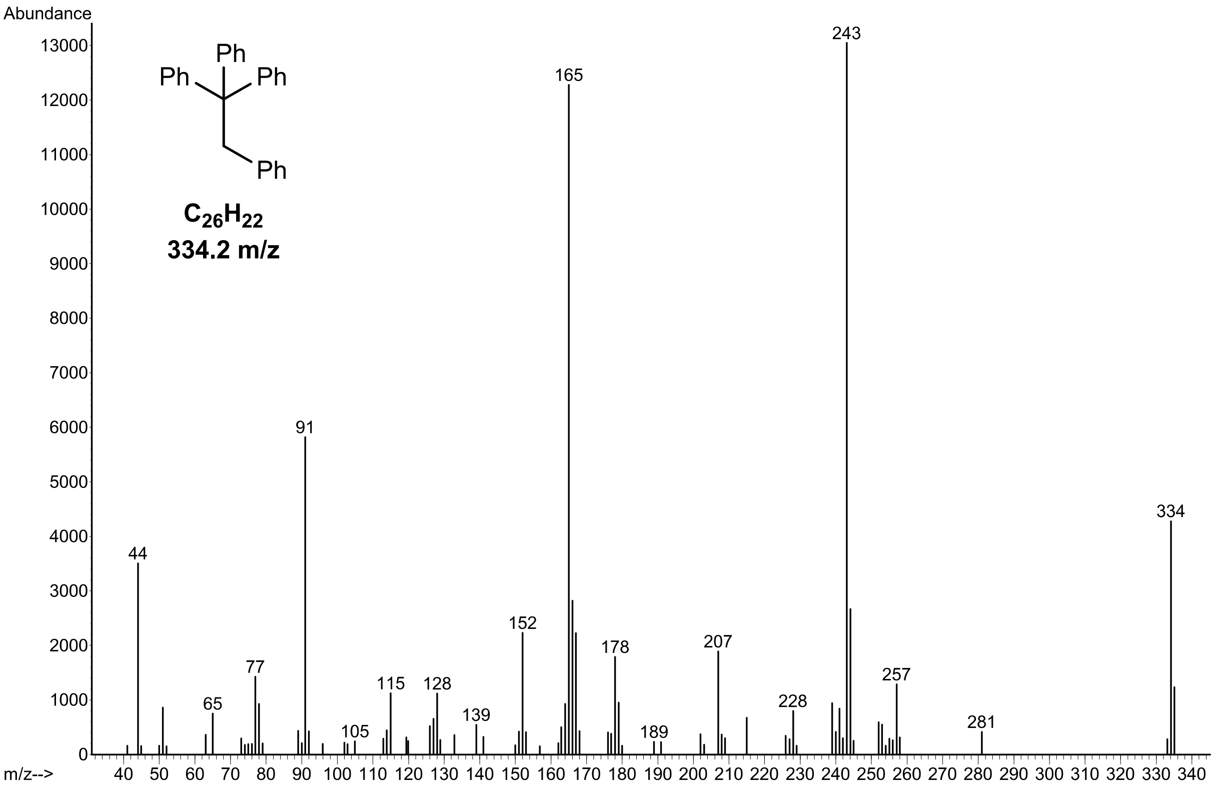

Figure S81. Mass spectrum of 1,1,1,2-tetraphenylethane observed at an elution time of 13.82 min.

8. X-ray crystallographic details

The crystal data of all compounds was collected on a Rigaku XtaLAB Synergy-R diffractometer with a HPA area detector and multi-layer mirror monochromated Cu-K_{α} radiation. Multi-scan absorption correction was applied to the data.[10,11] The structures were solved with SHELXT,[12] refined with the SHELXL[13] using full matrix least squares minimization on F^2 [14] using using Olex2 as the graphical interface.[15] All non-hydrogen atoms were refined anisotropically. The hydrogen atoms were located in idealized positions and refined isotropically with a riding model.

Crystallographic data have been deposited with the Cambridge Crystallographic Data Center as supplementary publication. These data can be obtained free of charge from The Cambridge Crystallographic Data Centre *via* www.ccdc.cam.ac.uk/data_request/cif.

(^{Et}PDI^{Bn})TiI_2 was refined as a 2-component twin. Component 2 was rotated by –165.7141° around [0.05 0.59 0.81] (reciprocal) or [–0.15 0.75 0.65] (direct). The BASF parameter was refined to 1.7%.

Partial substitution was observed at the *para*-atom of the pyridine ring. In the unsubstituted system, this cavity was filled with solvent (toluene). Toluene molecules were refined isotropically with a constrained U_{iso}. The value of this tensor was computed using free variable and SIMU command. The geometry of both toluene molecules (residues 11 and 16) was constrained to the idealized one.

Table S3. Selected bond lengths (in Å) of X-R_2 as determined by single crystal X-ray diffraction and DFT calculations.

X-$(R)_2$								
X	2	2	2	1	2	2	2	1
R	CH_2Ph	CH_2Ph	CH_2Ph	CH_2Ph	*p*-Tol	*p*-Tol	*p*-Tol	Ph
	X-ray	DFT, S = 0	DFT, S = 1	X-ray	X-ray	DFT, S = 0	DFT, S = 1	X-ray
Ti−$C^{ap.}$	2.161(2)	2.156	2.152	2.138(1)	2.159(1)	2.139	2.105	2.151(2)
Ti−$C^{eq.}$	2.184(3)	2.201	2.178	2.125(1)	2.086(2)	2.072	2.088	2.089(2)
Ti−C^{ipso}	2.558(3)	2.598	2.671	3.163(1)	2.530(2)	2.454	2.741	2.699(2)
Ti−N1	1.986(2)	1.980	2.089	2.152(1)	1.989(1)	1.983	2.084	2.147(1)
Ti−N2	2.120(2)	2.077	2.073	2.009(1)	2.075(1)	2.082	2.077	1.980(1)
Ti−N3	2.136(2)	2.146	2.084	1.989(1)	2.1230(1)	2.104	2.063	1.985(1)
C2−N2	1.359(4)	1.354	1.355		1.355(2)	1.345	1.344	
C8−N3	1.335(3)	1.338	1.353		1.3340(2)	1.342	1.340	
C2−C3	1.408(4)	1.402	1.416		1.408(2)	1.407	1.421	
C7−C8	1.429(4)	1.412	1.416		1.420(2)	1.409	1.422	
C3−N1	1.405(3)	1.391	1.363		1.393(2)	1.393	1.369	
C7−N1	1.390(3)	1.381	1.362		1.3880(2)	1.389	1.369	
Δ	0.046	0.041	0.058		0.047	0.041	0.066	

Table S4. Crystallographic data and structure refinement part 1.

	$(^{iPr}PDA)Ti(CH_2Ph)(OTf)$	$(^{iPr}PDA)TiPh_2$	$(^{iPr}PDA)Ti(Ph)(OTf)$	$(^{Et}PDI)Ti(CH_2Ph)_2$
CCDC	2454888	2454892	2454891	2454886
Empirical formula	$C_{39}H_{48}N_3O_3F_3STi$	$C_{45.5}H_{57}N_3Ti$	$C_{38}H_{46}F_3N_3O_3STi$	$C_{43}H_{49}N_3Ti$
Formula weight	743.76	693.84	729.74	655.75
Temperature/K	100.15	99.97(15)	99.97(10)	99.99(11)
Crystal system	monoclinic	orthorhombic	monoclinic	triclinic
Space group	$P2_1/c$	*Pbca*	$C2/c$	$P\bar{1}$
a/Å	26.9438(3)	16.41480(10)	31.5965(3)	11.9292(4)
b/Å	14.67880(10)	19.80770(10)	16.70740(10)	12.5726(4)
c/Å	20.7733(2)	24.53450(10)	29.3336(2)	12.7783(3)
α/°	90	90	90	68.086(3)
β/°	111.7890(10)	90	105.7250(10)	89.265(2)
γ/°	90	90	90	77.620(3)
Volume/Å^3	7628.93(13)	7977.13(7)	14905.5(2)	1731.90(10)
Z	8	8	16	2
ρ_{calc}g/cm^3	1.295	1.155	1.301	1.257
μ/mm^{-1}	2.866	2.061	2.924	2.346
F(000)	3136.0	2984.0	6144.0	700.0
Crystal size/mm^3	0.29 × 0.25 × 0.17	0.257 × 0.181 × 0.14	0.379 × 0.229 × 0.12	0.169 × 0.054 × 0.039
Radiation	CuKα (λ = 1.54184)	Cu Kα (λ = 1.54184)	Cu Kα (λ = 1.54184)	Cu Kα (λ = 1.54184)
2Θ range for data collection/°	6.982 to 140.152	7.206 to 150.302	5.812 to 150.132	7.478 to 149.75
Index ranges	-32 ≤ h ≤ 27, -17 ≤ k ≤ 17, -25 ≤ l ≤ 25	-19 ≤ h ≤ 11, -24 ≤ k ≤ 24, -30 ≤ l ≤ 29	-38 ≤ h ≤ 37, -20 ≤ k ≤ 20, -36 ≤ l ≤ 33	-14 ≤ h ≤ 14, -15 ≤ k ≤ 15, -11 ≤ l ≤ 15
Reflections collected	78252	80241	47022	18315
Independent reflections	14323 [R_{int} = 0.0213, R_{sigma} = 0.0154]	8044 [R_{int} = 0.0258, R_{sigma} = 0.0146]	14526 [R_{int} = 0.0238, R_{sigma} = 0.0280]	6688 [R_{int} = 0.0570, R_{sigma} = 0.0646]
Data/restraints/ parameters	14323/84/947	8044/20/454	14526/0/899	6688/0/430
Goodness-of-fit on F^2	1.003	1.059	1.054	1.032
Final R indexes [I>=2σ (I)]	R_1 = 0.0394, wR_2 = 0.1022	R_1 = 0.0410, wR_2 = 0.1147	R_1 = 0.0374, wR_2 = 0.1027	R_1 = 0.0587, wR_2 = 0.1555
Final R indexes [all data]	R_1 = 0.0408, wR_2 = 0.1029	R_1 = 0.0433, wR_2 = 0.1165	R_1 = 0.0438, wR_2 = 0.1062	R_1 = 0.0761, wR_2 = 0.1656
Largest diff. peak/ hole / e Å^{-3}	0.63/-0.46	0.92/-0.39	0.47/-0.34	0.79/-0.65

Table S5. Crystallographic data and structure refinement part 2.

	$(^{Et}PDI^{Bn})TiI_2$	$(^{Et}PDI)Ti(p\text{-tol})_2$	$(^{Et}PDI)TiI_2$	$(^{Et}PDI)Ti(OTf)_2$
CCDC	2455785	2454887	2454890	2454889
Empirical formula	$C_{107}H_{119.15}I_4N_6Ti_2$	$C_{43}H_{49}N_3Ti$	$C_{29}H_{35}I_2N_3Ti$	$C_{33.5}H_{41}F_6N_3O_6S_2Ti$
Formula weight	2092.63	655.67	727.30	807.71
Temperature/K	100.15	99.88(10)	99.99(16)	102(1)
Crystal system	triclinic	triclinic	monoclinic	monoclinic
Space group	$P\bar{1}$	$P\bar{1}$	$P2_1/c$	$P2_1/c$
a/Å	8.956(9)	10.6295(2)	11.74330(10)	12.33910(10)
b/Å	14.5657(19)	11.9825(2)	18.46910(10)	14.05030(10)
c/Å	18.658(2)	16.5661(3)	13.69010(10)	21.3894(2)
α/°	83.30(5)	70.042(2)	90	90
β/°	78.01(5)	85.791(2)	103.9440(10)	91.0230(10)
γ/°	87.32(7)	65.946(2)	90	90
Volume/Å^3	2364(2)	1805.73(7)	2881.72(4)	3707.65(5)
Z	1	2	4	4
ρ_{calc}g/cm^3	1.470	1.206	1.676	1.447
μ/mm^{-1}	12.070	2.250	19.495	3.691
F(000)	1059.0	700.0	1432.0	1676.0
Crystal size/mm^3	0.426 × 0.083 × 0.06	0.195 × 0.132 × 0.034	0.158 × 0.036 × 0.032	0.38 × 0.233 × 0.151
Radiation	CuKα (λ = 1.54184)	Cu Kα (λ = 1.54184)	Cu Kα (λ = 1.54184)	Cu Kα (λ = 1.54184)
2Θ range for data collection/°	6.112 to 151.598	5.692 to 150.4	7.758 to 150.832	7.166 to 150.496
Index ranges	-11 ≤ h ≤ 11, -17 ≤ k ≤ 17, -22 ≤ l ≤ 23	-13 ≤ h ≤ 13, -9 ≤ k ≤ 14, -20 ≤ l ≤ 20	-13 ≤ h ≤ 14, -21 ≤ k ≤ 22, -17 ≤ l ≤ 16	-15 ≤ h ≤ 14, -17 ≤ k ≤ 15, -26 ≤ l ≤ 25
Reflections collected	9291	25806	46377	27965
Independent reflections	9291 [R_{int} = /, R_{sigma} = 0.0091]	7138 [R_{int} = 0.0206, R_{sigma} = 0.0190]	5740 [R_{int} = 0.0313, R_{sigma} = 0.0163]	7293 [R_{int} = 0.0210, R_{sigma} = 0.0169]
Data/restraints/ parameters	9291/0/526	7138/344/544	5740/0/322	7293/70/525
Goodness-of-fit on F^2	1.046	1.082	1.063	1.027
Final R indexes [I>=2σ (I)]	R_1 = 0.0548, wR_2 = 0.1574	R_1 = 0.0334, wR_2 = 0.0938	R_1 = 0.0219, wR_2 = 0.0556	R_1 = 0.0336, wR_2 = 0.0903
Final R indexes [all data]	R_1 = 0.0556, wR_2 = 0.1581	R_1 = 0.0346, wR_2 = 0.0945	R_1 = 0.0236, wR_2 = 0.0562	R_1 = 0.0348, wR_2 = 0.0912
Largest diff. peak/ hole / e Å^{-3}	1.21/-2.99	0.37/-0.47	0.80/-0.69	0.61/-0.47

9. Cyclic and Differential Pulse Voltammetry

Cyclic voltammetry was conducted using a Reference 600 potentiostat from Gamry Instruments. Measurements were performed under an argon atmosphere at ambient temperature in THF using $[N^nBu_4][BAr^F_4]$ as a conducting salt with a concentration of 0.02 M. $[N^nBu_4][BAr^F_4]$ was chosen for all measurements due to decomposition of $(^{Et}PDI)TiR_2$ (R = *p*-tol, CH_2Ph) when $[N^nBu_4][PF_6]$ was used. Different scan rates of from 50 to 700 mV/s were employed for all measurements. A standard three electrode cell set-up with a glassy carbon working electrode, platinum counter electrode and a platinum wire separated by a Vycor membrane as a reference electrode was used. Potentials were internally referenced against the ferrocene/ferrocenium couple (Fc/Fc^+).

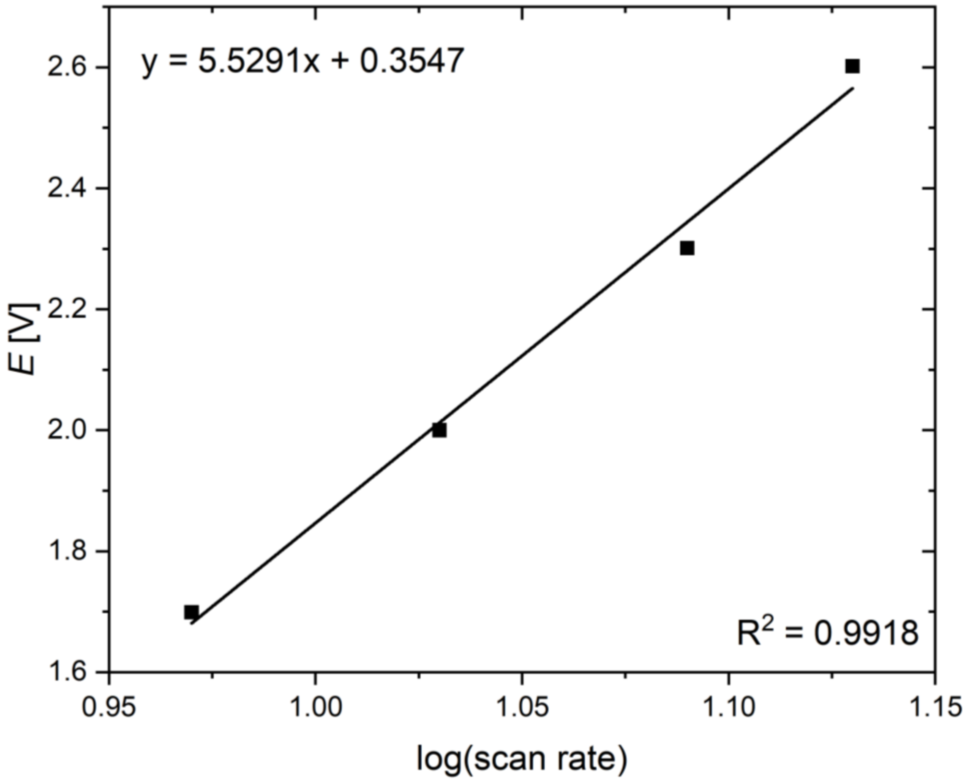

Figure S82. Peak potential vs. log (scan rate) for $(^{iPr}PDA)TiPh_2$ affording a linear relationship.

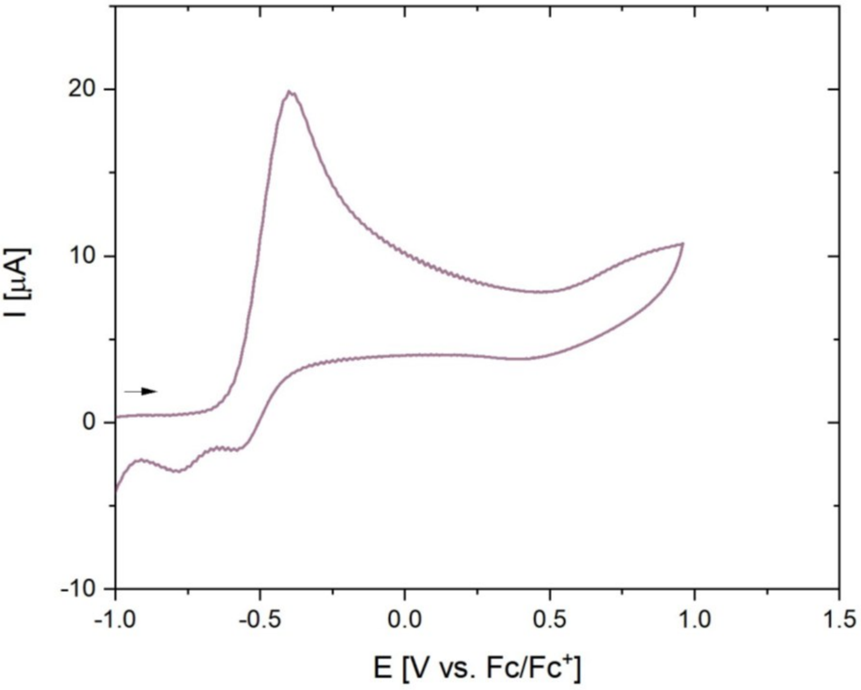

Figure S83. Cyclic voltammetry of (^{Et}PDI)Ti(CH_2Ph)$_2$ (full range).

Differential pulse voltammograms were recorded using a pulse amplitude of 25 mV, a pulse duration of 0.1 s, a step increment of 5.0 mV, and a sampling interval of 1 s.

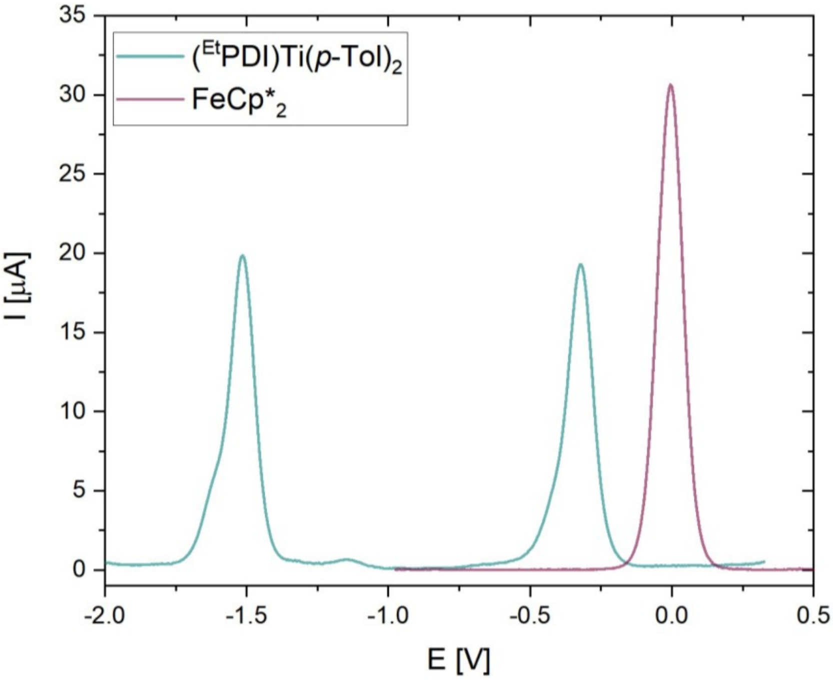

Figure S84. Differential pulse voltammetry of equimolar solutions of (^{Et}PDI)Ti(*p*-Tol)$_2$ and decamethylferrocene (referenced to the latter). The peak currents suggest that the titanium complex undergoes two subsequent single electron oxidations.

10. Quantum Chemical Calculations

DFT calculations were performed with the ORCA 6.0.0 program package.[16,17,18] Geometry optimizations and single-point calculations were carried out using the TPSSh functional in combination with Grimme's dispersion correction D4.[19,20] Alrichs' all-electron Gaussian triple zeta basis set def2-TZVP was employed on all atoms.[21] The RIJCOSX approximation was used to accelerate the calculations.[22,23,24] Final Gibbs free energies were then obtained by using the single-point electronic energies and the thermal corrections from the frequency calculations. The broken symmetry calculations were performed as introduced by Ginsberg[25] and Noodleman et al.[26] The general notation for broken symmetry (m,n)[27] has been adopted, where m (n) denotes the number of spin-up (spindown) electrons at the two interacting fragments.[28] For mechanistic considerations, stationary points were identified as intermediates or transition states by frequency calculations and the presence or absence of an imaginary frequency. Single-point calculations were conducted using the optimized geometries (PBE0-D4/def2-TZVP) with a larger TZVPP basis and a CPCM solvation model with the dielectric constant of benzene. Final Gibbs free energies were then obtained by using the single-point electronic energies and the thermal corrections from the frequency calculations. LOBA calculations[29] on all intermediates and transition states were preformed using Multiwfn.[30]

Table S6. Solvent-corrected Gibbs energies (Final Gibbs; benzene) for TPSSh-D4/def2-TZVP for comparison of singlet and triplet energies of PDI complexes.

Compound	S	Final Gibbs [Hartree]	Final Gibbs [kcal·mol^{-1}]	Relative Final Gibbs [kcal·mol^{-1}]
[(^{Et}PDI)Ti($CH_2Ph)_2$]	0	-2682.173934	-1683088,283445340	-4.114
[(^{Et}PDI)Ti($CH_2Ph)_2$]	1	-2682.167378	-1683084,169201400	0.000
[(^{Et}PDI)Ti(*p*-Tol)$_2$]	0	-2682.168762	-1683085.03767386	-2.511
[(^{Et}PDI)Ti(*p*-Tol)$_2$]	1	-2682.164761	-1683082.52701035	0.000

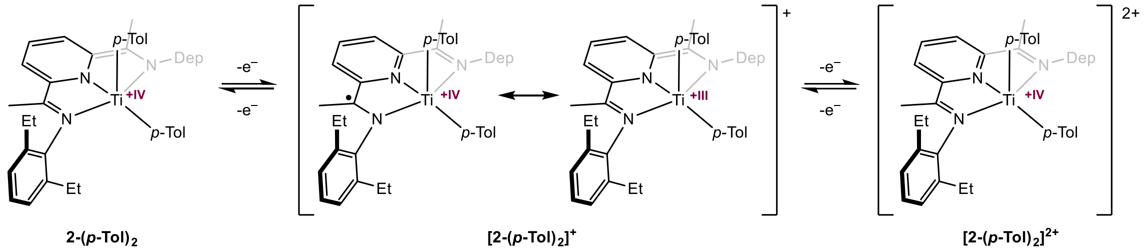

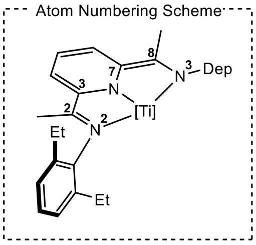

Figure S85. Oxidation of (^{Et}PDI)Ti(*p*-Tol)$_2$ (outer-sphere mechanism).

Table S7. Selected bond lengths (in Å) of [(^{Et}PDI)Ti(*p*-Tol)$_2$]$^{x+}$ as determined by DFT calculations (TPSSh D4 def2-TZVP level of theory); for neutral versions the singlet solution was chosen.

X	0 (±0)	+1	+2
Ti−C$^{ap.}$	2.152	2.103	2.037
Ti−C$^{eq.}$	2.079	2.027	1.987
C$^{eq.}$····C$^{ap.}$	3.672	3.558	3.423
Ti−N1	1.981	2.046	2.150
Ti−N2	2.082	2.115	2.171
Ti−N3	2.110	2.153	2.188
C2−N2	1.346	1.319	1.292
C8−N3	1.342	1.315	1.291
C2−C3	1.407	1.437	1.476
C7−C8	1.410	1.441	1.477
C3−N1	1.395	1.368	1.339
C7−N1	1.390	1.364	1.339
Δ	0.040	0.098	0.161

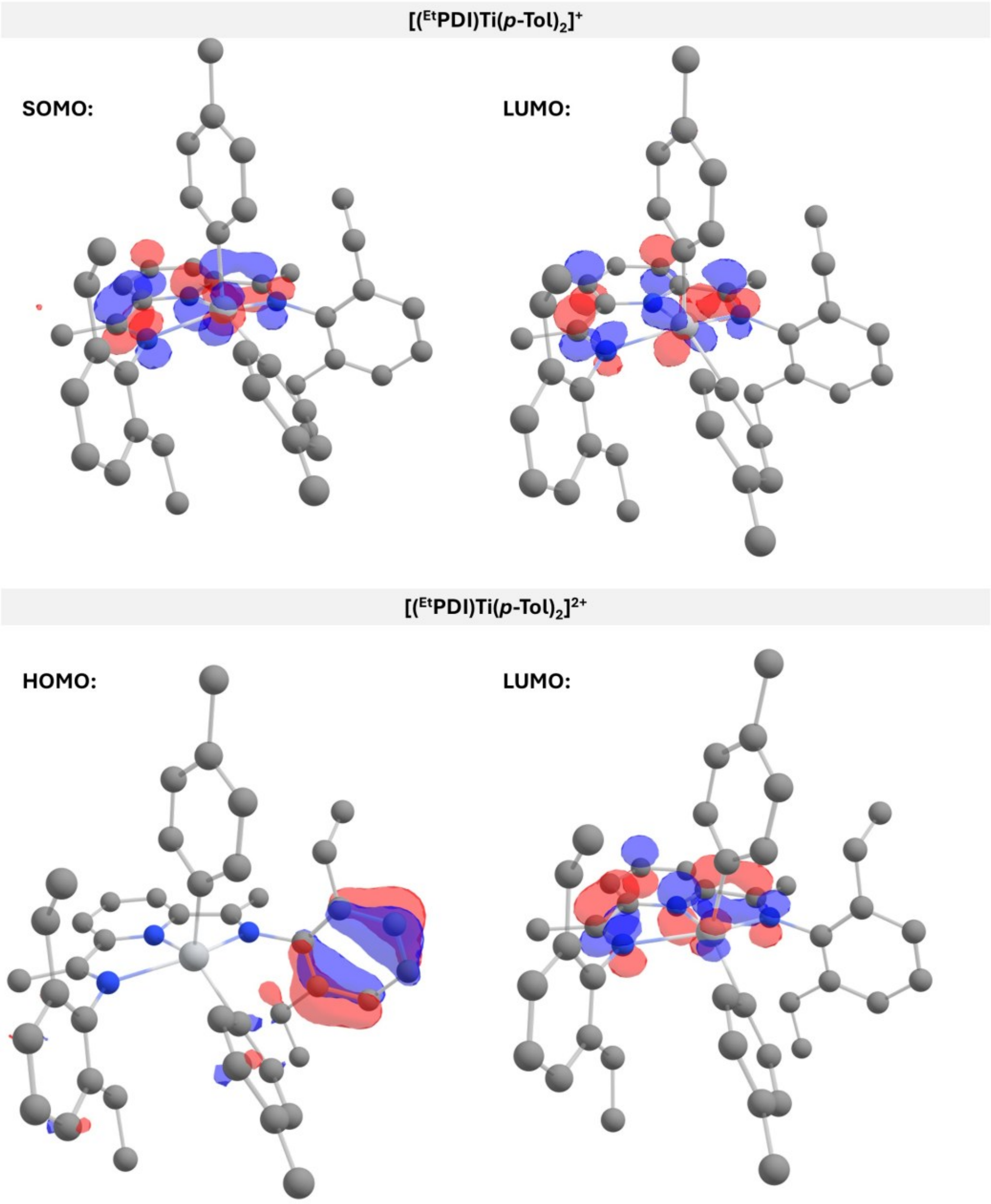

Figure S86. Frontier orbitals of [(^{Et}PDI)Ti(p-Tol)$_2$]$^{n+}$ (n = 1,2). Contour value = 0.06.

Table S8. Solvent-corrected electronic energies (E_{el}; benzene) for PBE0-D4/def2-TZVP, non-thermal ZPE corrections (ZPE), thermal enthalpy correction (H_{therm}), and entropy correction (-TS) for mechanistic calculations; all energies are given in Hartree; relative Gibbs energy is given with reference to [(^{Me}PDI)TiPh$_2$] + 2 I_2.

Compound	E_{el}	E_{ZPE}	H_{therm}	-TS	Final Gibbs	Relative Gibbs Energy [kcal·mol^{-1}]
[(^{Me}PDI)TiPh$_2$]	-2444.628248	0.641069	0.000944	0.101707	-2444.087941	0
[(^{Me}PDI)TiPh$_2$I]I	-3040.141556	0.646718	0.000944	0.114212	-3039.608106	-17.281
[(^{Me}PDI)TiPh$_2$I]I$_3$	-3635.636933	0.647957	0.000944	0.125377	-3635.113409	-25.237
[(^{Me}PDI)TiPh$_2$I]I$_3$-TS	-3635.610755	0.640834	0.000944	0.112591	-3635.081568	-5.256
[(^{Me}PDI)TiI]I$_3$	-3172.733360	0.463947	0.000944	0.104132	-3172.372601	-62.484
[(^{Me}PDI)TiI$_2$]	-2577.296382	0.462601	0.000944	0.094255	-2576.927093	-92.050
I_2	-595.463899	0.000513	0.000944	0.030182	-595.492625	/
Ph$_2$	-462.939444	0.182129	0.000944	0.043795	-462.800166	/

XYZ Coordinates

(^{iPr}PDA)Ti(CH$_2$Ph)$_2$

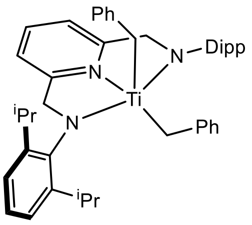

Ti	8.13391959994930	4.44265796905482	4.16873200749178
N	8.27526947834812	6.22553746832418	5.06150354187288
N	6.11741565186980	5.06137612623933	4.65108066663592
N	6.99304198574036	2.82437250462165	4.04519972907101
C	5.88572342764846	6.29342858569924	5.11795617294973
C	5.10302250435897	4.25134247308785	4.31952448974068
C	7.13518512718138	7.05514659570599	5.39475175259766
H	7.12476804385303	7.98700706785920	4.80606250406230
H	7.15146774085808	7.37273460942765	6.44619395092374
C	9.49973996705766	6.81475838563352	5.50921186949448
C	7.45742990642943	1.48191998298737	4.16581049284175
C	9.97306341421837	6.54358552062407	6.81555295313087
C	4.59307397140301	6.76494371948849	5.28709772986352
H	4.42436818847568	7.76250945866704	5.67355474389584
C	7.00008239273677	5.35814004920810	1.57412996720288
C	10.77199668372045	3.46662049002318	3.95169730586944
C	10.22401423872355	7.69043954033451	4.67356768980031
C	3.78662435107048	4.66669639041617	4.43918152347827
H	2.98007642047548	4.00400715445333	4.15192602766397
C	9.84596704182545	3.54560948798296	5.09558970670896
H	10.20681049059319	4.21756458711538	5.87112820156826
H	9.59850634715158	2.56763473614559	5.50318651126018
C	7.65221960558741	0.68277665836534	3.02237792486750
C	7.69756398164221	0.94499884215354	5.45245893202945
C	11.92326866137565	7.91991709270624	6.38423208274864
H	12.86833100445733	8.33342394028146	6.71787691809552
C	11.43236117530385	8.22142866228475	5.12476603150509
H	11.99316114852909	8.88536224863739	4.47571086202502
C	8.12104348158575	-0.62178372062807	3.17265821185398
H	8.27567513566533	-1.23533548708680	2.29145959701886

C	11.18514905665603	7.09707765444517	7.22268780994663
H	11.55774303151652	6.88215669048498	8.21768910851055
C	11.59666121732578	4.55366747289645	3.60845097789519
H	11.58405080190014	5.44159537698344	4.23015220662764
C	5.56117266613217	2.92216591823714	3.82405357268739
H	5.01070886107628	2.11533850758149	4.32417616827332
H	5.30604445439363	2.85166740029416	2.75420632219573
C	8.17517545222790	-0.35994388325723	5.55432070145816
H	8.37766709370263	-0.77824369994561	6.53287028710984
C	7.30669311859803	1.20733423586941	1.64774895614848
H	7.13685060492767	2.27693004787044	1.76706675368561
C	9.68738381959342	8.11806363562841	3.32502509618868
H	8.83807320224137	7.47060224931756	3.11230616249382
C	8.32626145362807	4.93021053781465	2.07414936073092
H	9.07647979178387	5.72123822094148	2.02069422830206
H	8.70686282775469	4.04954723970055	1.55039297123397
C	9.17545815151237	5.71203680357029	7.80425145931426
H	8.42505675568956	5.15781837778308	7.23308666338730
C	3.53257646498967	5.93842038168980	4.93778002234003
H	2.51186424732424	6.28349398225997	5.05428936700372
C	6.43962900008934	6.59756582337833	1.93264148929597
H	7.02075935029865	7.28331774101354	2.53734833963896
C	10.83909773293960	2.31714154280066	3.14376041873733
H	10.22905449184380	1.45798334711419	3.39909374779817
C	6.20749742015555	4.52563623024870	0.76591162612711
H	6.62242074947671	3.59030263497191	0.41159016554039
C	7.40718208080182	1.74663738086936	6.70944058081576
H	7.54236710574181	2.80304281323102	6.46049333189046
C	8.39473414459451	-1.14111411400039	4.42714173182534
H	8.77072269216597	-2.15272218440512	4.53128050968822
C	5.15538099225378	6.95828886581803	1.55487944290461
H	4.75907596239704	7.92059643245140	1.86164987889986
C	4.91835958499059	4.88204548328553	0.38600772420968
H	4.34148462119007	4.20823340758652	-0.23905277198598
C	6.01053993169482	0.56085648289917	1.13588462319212
H	6.15222130209458	-0.51256728799954	0.97914059526427
H	5.71035753888439	1.00426902281707	0.18171484293523
H	5.19100643779730	0.68620680133781	1.84703837471913
C	12.43863174884894	4.49636226857577	2.50649269701244
H	13.07082102542397	5.34576434325036	2.27080436075359
C	8.45054025506102	6.62070934163891	8.81266568069014
H	9.17795459473244	7.16349053385021	9.42335468345584
H	7.82148896081721	6.02635049298933	9.48186474958219
H	7.81891202615101	7.36219365223734	8.31985081722917
C	11.68745866987391	2.26191798593099	2.04686647696524
H	11.73349112376231	1.35717776036672	1.45015325080845
C	4.37405420937650	6.09673101983822	0.78797992172606
H	3.36882163597905	6.37628792484332	0.49464409133980
C	9.18823278101931	9.56994120770800	3.38174552511048
H	8.45106426037419	9.71012596849445	4.17641599958060
H	8.72907188693608	9.85542161547050	2.43081873256812
H	10.01757558980900	10.25602812114844	3.57667742443686
C	10.70261129828408	7.95072730885692	2.19006697813926
H	11.56763083061412	8.60520905192403	2.33053947722276
H	10.24260323590857	8.21158792856446	1.23311921962621
H	11.06183305547225	6.92275163908315	2.12776504704382
C	8.42942444686058	1.02492423089337	0.62330207050260
H	9.34046285335287	1.52947669745901	0.94445442432303
H	8.12958962351962	1.44530218572073	-0.34123750902714
H	8.65971408796892	-0.03214741901135	0.46246362506749
C	5.94717927535011	1.56244083743772	7.15258884896505
H	5.74569619100235	0.51141649060901	7.37846304750916
H	5.24670663882240	1.88127357235304	6.38010478527442
H	5.74589747004870	2.14971033579003	8.05355743593543
C	10.03853878003797	4.70749781199026	8.57793047178188
H	10.60888129856376	4.06055296179576	7.91011137155859
H	9.40516428448969	4.07714219753242	9.20616975662486
H	10.74394314842466	5.21863129807234	9.23840390001928

C	12.48660869327424	3.35298050688076	1.71383423568056
H	13.14837487152024	3.30662327577193	0.85666848114990
C	8.34340426188225	1.40822365985830	7.87213751204941
H	8.16379238688974	2.09349552202920	8.70355051162804
H	9.39299567237575	1.48922816664532	7.58227085401135
H	8.17081344694998	0.39553079000223	8.24675809835531

(^{iPr}PDA)TiPh$_2$

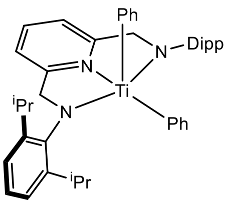

Ti	7.75408145795010	4.54865641482596	4.06596938031786
N	8.11976924619235	6.20029465312387	5.09633672830696
N	5.89574642279989	5.10275111794692	5.03585957642535
N	6.59782322280926	2.94382236784324	4.04073091210540
C	5.80741597633045	6.26525244426420	5.69421432900860
C	4.82403466070187	4.31469741685467	4.87200721855888
C	7.10869312048627	6.98771318481532	5.76966215585146
H	6.99766615209060	7.98060185817850	5.30674128020004
H	7.36762688823443	7.17970166819899	6.82149837766020
C	9.40843253381857	6.81749871921341	5.18301666814585
C	7.06460272671630	1.60240026805259	4.12873576218465
C	10.33572432348362	6.37021550009507	6.14900632514042
C	4.59913419810187	6.69419031735489	6.22421589272363
H	4.54245922549878	7.63220644296439	6.76247414962373
C	7.31821108775459	5.29774065782860	2.12774006275550
C	9.55865565163737	3.51124292157178	4.15454784554924
C	9.74243664698262	7.87326171100039	4.31155417433227
C	3.58167851378679	4.69201783631293	5.35800019810798
H	2.72203228294030	4.04936525223643	5.21441525291127
C	7.54349372542350	0.92490475247554	2.99352670471037
C	7.05086110542080	0.96187938108227	5.38662923935579
C	11.91282955152437	8.03971580085829	5.37324405851860
H	12.88642294167012	8.51164022769398	5.44648280593977
C	10.99749900933123	8.46980429305222	4.42552039254423
H	11.26130225696131	9.28008877048929	3.75387296537197
C	7.99660213964082	-0.38570720800632	3.13498643668198
H	8.36769199168329	-0.91353463073223	2.26305003020990
C	11.57801599174948	6.99548510118549	6.22321931692243
H	12.29990372434129	6.65536088700468	6.95674174838263
C	10.35316753431629	4.39143028507066	3.40591989018511
H	9.95112043374600	5.35039958435203	3.07112235192730
C	5.15394037727122	3.04580926997764	4.15574609698243
H	4.72352264640051	2.19008079243163	4.69055110747701
H	4.67778128205736	3.06010944425386	3.16140532667643
C	7.50853527493435	-0.35083931106795	5.48129518469005
H	7.51082954852546	-0.84562076032573	6.44731352368519
C	7.55460032202294	1.58666425462846	1.63403772449812
H	7.31284029130964	2.63941609411781	1.78642177759209
C	8.77926441980636	8.36802606375201	3.25089824858492
H	7.90812327398770	7.71147658276366	3.26416258869243
C	10.00488826184975	5.24024850206916	7.10255984944263
H	9.18471452939264	4.67911877111510	6.65068495491345
C	3.47470912020363	5.89626103551094	6.04615448113563
H	2.51889858803104	6.20651243282650	6.45222568246150
C	6.17996313355460	6.09945390017287	1.93735885607594
H	5.56735967649472	6.38992115681907	2.78616448770378
C	10.13808966051116	2.30070323904001	4.55531674898952
H	9.55887822897351	1.57714166293253	5.11696356064242
C	8.05942503840824	4.98345058987015	0.97735769088716
H	8.95231810789751	4.37248231779080	1.06039490783043
C	6.61400610034381	1.68893865639688	6.64474345747386
H	6.26495619761107	2.68085600196579	6.35326880384449
C	7.98033628002968	-1.02694364094541	4.36473449433124
H	8.33793656006075	-2.04663941916817	4.45447954412751
C	5.79954859215209	6.55724192705765	0.67792624817833
H	4.91437817077816	7.17673240849304	0.57162465820492
C	7.68824155595462	5.43331315942433	-0.28720855119291
H	8.28909780946629	5.16974770172288	-1.15190813457232

C	6.46926063326787	0.98981418337690	0.72720392031003
H	6.66021594231962	-0.07023034095994	0.53367842138026
H	6.44463756380532	1.51303847781454	-0.23290115011339
H	5.48147861369057	1.07292550573205	1.18754180336163
C	11.67352435143794	4.09522872560366	3.06666846356577
H	12.26355526346030	4.80248345446297	2.49426698393622
C	9.52229937769036	5.77957824181261	8.45744962248734
H	10.31308946525844	6.35779763547512	8.94455118911366
H	9.24448249252054	4.95524972869714	9.12103858381989
H	8.65441655462472	6.43299234155153	8.34568986075203
C	11.45027422564890	1.99098594654426	4.22219281349474
H	11.87837904991676	1.04461786462139	4.53579574828533
C	6.55383422207391	6.22290190915658	-0.44242945184300
H	6.26202447030388	6.57706616243562	-1.42505955835834
C	8.31742239850598	9.80347576968437	3.54073466785930
H	7.86798050248824	9.88898758163905	4.53396452125022
H	7.57854169265815	10.12335777494126	2.80037258608069
H	9.15863884483106	10.50165650870861	3.50050652175342
C	9.38953221412231	8.27967581450719	1.84697334337912
H	10.25664154328789	8.93944897745980	1.75011397532775
H	8.65287443708159	8.57344417662609	1.09527249720129
H	9.70734949264759	7.26115759322465	1.61950736249726
C	8.93031645028608	1.50688794453129	0.96381800580384
H	9.70304613227464	1.94789687598141	1.59638801867632
H	8.91493725316665	2.04227213995001	0.01049606954577
H	9.21524607750141	0.47163615962581	0.75478840951574
C	5.46575837978660	0.96686344168228	7.35917023808649
H	5.77632694990758	-0.02196983962417	7.70790812839584
H	4.60783278692680	0.82804208739672	6.69589951501705
H	5.13672543698438	1.53984776981916	8.23088072531529
C	11.17971794220837	4.27874030940909	7.30402935620968
H	11.58285551347422	3.94332442238564	6.34827005051244
H	10.85012600242707	3.39956368503489	7.86410480090632
H	11.98751625919589	4.74435575489863	7.87618742865075
C	12.22082054738501	2.88767016720348	3.47983342311438
H	13.24549875448183	2.64065433700808	3.22437247447113
C	7.80168333968721	1.89903918522815	7.59386622003791
H	7.49047182745619	2.46027836397418	8.48000902845949
H	8.60014845416799	2.45395867100033	7.09862307722541
H	8.21231074888459	0.94152366054494	7.92620675449822

(^{Et}PDI)Ti(CH$_2$Ph)$_2$ (S = 0)

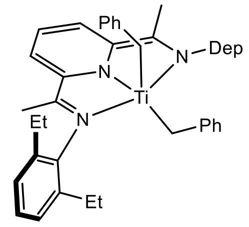

Ti	9.99729182243529	7.43957038778840	3.37990411605300
N	10.84666260763742	8.45876026184686	1.90629515229239
N	10.75907516565188	6.00772762373761	2.07447551578032
N	10.68210519528409	9.24802210604841	4.20841883676834
C	11.19219580743330	7.80932576376970	0.72322114938090
C	11.10616245412331	6.40985919752648	0.83914745688324
C	11.16635297960706	9.80324918554573	2.04137922733813
C	11.03695853593231	10.23813855574386	3.37565758172782
C	7.99580790616560	7.75821676116750	2.69666138728966
C	10.74686038642492	4.61173258396997	2.36933639132394
C	11.80505289073999	9.91645854684933	-0.28216889963648
H	12.15404483066639	10.48546221618389	-1.13393792541720
C	11.60096054186353	10.54023175672379	0.95147772459314
H	11.81126161053092	11.59599690794311	1.06808190939237
C	11.84141286987986	4.09418064920488	3.08585415463036
C	11.63301877124952	8.53730014124030	-0.37545089667410
H	11.86794880615023	8.02189761722589	-1.29838584746945
C	7.73171476948032	8.40001104338338	1.47185828983413
H	8.55765331949323	8.71555550335702	0.84383119070504
C	8.27804334893548	9.56361245025384	5.82495818658390
H	7.57367458550712	9.45197424383277	6.65450646971773
H	8.22208978462014	8.63364915292762	5.25049408349775
C	5.32865392975364	8.27406736129147	1.78565194799693
C	12.98447420258272	5.01115036548145	3.44854380592237

H	12.55975679063631	5.91233457595534	3.90202377957921
H	13.46777475516179	5.35700364652344	2.52646483937871
C	9.65045693984938	3.81171511385238	2.01802614740839
C	11.39589634688391	5.44140083444322	-0.25978262247562
H	12.10467225179380	4.68015853820297	0.07803832277254
H	11.81413654436965	5.94469301497398	-1.13007526927646
H	10.48913408417392	4.91507888524974	-0.57282620788790
C	10.79768846427662	9.50215779699730	5.60352482863392
C	9.66275097909258	9.69289759027775	6.39852835984182
C	10.30046037731779	6.51710606253193	5.21015361825794
C	5.56312058112379	7.63215505173161	3.00164165524080
H	4.71950405818847	7.31946988082357	3.61120584640542
C	6.85995073599130	7.38103454384928	3.43736422940737
H	6.97727035920114	6.87619864195764	4.39336515719325
C	6.43832917149912	8.64897677585708	1.02541114397826
H	6.28592823315974	9.14620177626585	0.07090319046484
C	8.45537115131945	4.38248586187072	1.28912822585305
H	8.45613204332229	4.01864998827430	0.25402582643857
H	8.54637340802482	5.46861880784149	1.23934710342095
C	10.74389220134644	1.93913675014375	3.09919122665373
H	10.74174168678538	0.89322613375253	3.38530008470692
C	11.81826160013827	2.75134964902519	3.44565522807284
H	12.64473693932472	2.33197596700305	4.00616633773731
C	9.36841063282069	5.55774925638259	4.82334449321848
H	8.78750290761560	5.68637421337854	3.89287470998078
C	10.99540927482470	6.28415609170021	6.40425678076486
H	11.70867586955728	7.01430530104023	6.77095863018916
C	3.92962210913000	8.57292377017572	1.31910771049368
H	3.20157348817769	7.92176380000880	1.80752864094803
H	3.83537644026149	8.44551061128752	0.23784073686090
H	3.65367927521318	9.60777328573423	1.54951096351273
C	9.84375147794187	9.95630286620616	7.75549368528692
H	8.96914641547269	10.10620441108031	8.38045308010653
C	9.67568779200441	2.46673697504750	2.39140994549943
H	8.83967381992661	1.82775943468858	2.12873316744991
C	10.78115630439891	5.12346414338754	7.12846388757127
H	11.34163608867554	4.94981202844605	8.04328076399642
C	11.27330210597690	11.63972469135699	3.83809923755070
H	10.42351938262261	11.99840403516021	4.42425538871911
H	11.41950238930507	12.31023272221890	2.99234312440350
H	12.15184419809863	11.70647608652850	4.48673380744582
C	7.82497014531873	10.71655917941587	4.92533718093634
H	8.43946276567508	10.78043664327683	4.02676955888978
H	7.87922348672736	11.67172197724415	5.45507311470904
H	6.79317682121579	10.55749506209348	4.60381122130435
C	14.02966409939783	4.42036277565174	4.38662550141973
H	14.54015847060321	3.56052221805182	3.94387929941073
H	13.57644618806433	4.09977070279653	5.32813653925798
H	14.78840155109909	5.17121412326028	4.61952308142344
C	9.85220145232615	4.15488598791171	6.70831033544958
C	9.12998766744122	4.38800279909528	5.54592474442151
H	8.40695604159411	3.65816063846755	5.19786865778445
C	7.11136912677683	4.03097122710951	1.93417082646391
H	6.29521886604886	4.51164559793085	1.39085567043981
H	7.07074636228306	4.38088046539421	2.96771226223459
H	6.92952743177533	2.95374202517216	1.93572514020306
C	12.09095076850739	9.49947758962966	6.15840657976240
C	11.11338593593618	10.01453749346916	8.31233536247280
H	11.23617728479248	10.22401195743059	9.36893956777967
C	12.22781476322244	9.77128683225203	7.51777168112332
H	13.21330592750101	9.77316415222115	7.96804640045550
C	13.26468032673096	9.12046300228942	5.28515975299498
H	12.96714566413543	8.24075718100973	4.70209725183183
H	13.44568812032408	9.90097663545004	4.53679175178180
C	9.65829598170995	2.89034703074003	7.49865399624502
H	9.40139996494158	3.10908618087816	8.53918980695496
H	10.57573637141449	2.29350985697811	7.50991913847037
H	8.86315680324428	2.27773705036740	7.07067124439123

C	14.56644154141188	8.83208005586823	6.02517406830726
H	15.33484967640169	8.51208234780896	5.31755208338085
H	14.43888344017235	8.03620939815289	6.76410965440521
H	14.94519015605185	9.71595181830510	6.54527771097534

(^{Et}PDI)Ti(CH_2Ph)$_2$ (S = 1)

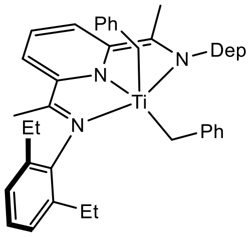

Ti	9.84975464238157	6.08549744369989	3.08072753267857
N	9.47894311600630	4.04809611907808	2.98025712893412
N	11.33237379829846	7.54665028038947	3.18359676612541
N	11.58186064912662	5.13368976962553	3.75588771080365
C	12.52635502689159	7.23828790966832	3.74187319765284
C	9.66498847020922	6.01976531819513	5.99764486458681
C	8.80479607391594	6.52555698430857	4.91084799187318
H	8.64742089727041	7.60366388534615	4.96564082609858
H	7.82614666830804	6.04285172607397	4.87538753487132
C	10.36015001830413	3.18551459667098	3.54210358818368
C	12.66749839497179	5.87748246600059	4.10789727897918
C	10.67137852170257	6.82172403737137	6.56188150491285
H	10.77224647459451	7.84710178597020	6.22664320711509
C	11.54801903992508	3.79570897346361	4.01234353769050
C	13.77492742097626	5.27719155424514	4.72954042374516
H	14.63394594124210	5.87471036016188	5.00172207957893
C	7.77117431862712	7.22278598501931	1.84688331374722
C	9.56230197805187	4.69892090036490	6.46467095662093
H	8.79100413974727	4.05890562858538	6.05367041551530
C	13.73985411971025	3.91447629070290	4.99490896371403
H	14.58334006480019	3.43872131781837	5.48151412681420
C	8.75534864345768	3.33907304574601	0.77327384834560
C	11.78622911168040	8.70337044869295	1.10274470026272
C	8.99549878471608	6.74476394167362	1.18914008637562
H	9.58110560002176	7.53987140993517	0.73106669487518
H	8.81802219435321	5.92642621810147	0.49394400493134
C	8.46401407109166	3.49641302311317	2.13796774032836
C	11.54196466457354	6.32369237779681	7.52020153621701
H	12.31182744781910	6.96860068062082	7.93129406494104
C	7.69911821296551	8.51044390375910	2.41545645177163
H	8.51370321559114	9.20337390689142	2.24124214368064
C	6.66924559851929	6.36744882986810	2.04795725598209
H	6.67296885846491	5.39028112381601	1.58196262851849
C	12.34944683929299	7.40664306159957	0.57096658096703
H	11.64325416376164	6.60443675733157	0.81465303794392
H	13.25948799982190	7.15762955964501	1.12945607247235
C	5.57921162581141	6.76263631415219	2.80968663067480
H	4.74591029145030	6.08125962218779	2.94372639980581
C	6.94560265919332	3.27502224075898	4.13506622592244
H	7.10804402243826	4.32108342210696	4.40565593678170
H	7.70983364150771	2.71436520766816	4.68338596153889
C	11.29503836486719	8.75600150222964	2.42074013288414
C	7.21237361022003	3.12484515736588	2.65951884379191
C	12.62855984301334	3.15865446966941	4.64336948454757
H	12.58437274978915	2.09897879908570	4.85434359197644
C	10.43178739542570	4.20031458625892	7.42433237167634
H	10.32720572311666	3.17394523535816	7.76094028203259
C	10.76114681718781	11.08243161144599	2.15754533314937
H	10.37531963840278	12.01199601036143	2.55726949823413
C	5.54553609929439	8.02727720225777	3.39212793527509
H	4.69588388730513	8.33335480348700	3.99122830771657
C	10.12173820036090	1.71514498473894	3.66517703464896
H	9.82796203606127	1.45800007841521	4.69021886549008
H	11.02824954890810	1.14799077857033	3.43978162615885
H	9.33022141686242	1.38305281287965	2.99400087214317
C	7.75657362421586	2.85372316461587	-0.07134727700640
H	7.96795239843573	2.75181342462616	-1.13106318991746
C	11.72573061102432	9.85761784349670	0.32616490354396
H	12.08259322466646	9.83541196207838	-0.69614445158232
C	10.11815244270286	3.65265583933234	0.20865041157111

H	10.59384818503838	4.43277016657267	0.80745094357054
H	9.99551540439747	4.05380254781074	-0.80202797987504
C	10.79991458155924	9.94802698385390	2.96870928471710
C	6.60569204969599	8.90373604152646	3.17334882433006
H	6.57777316881322	9.90202875944391	3.59665197466353
C	6.24951579580111	2.62742372432855	1.78484621946179
H	5.27800967119333	2.34082822857233	2.16785483871381
C	5.56533431578900	2.83864817282318	4.61100336865119
H	4.77230084650902	3.41827165623799	4.13144326976025
H	5.47972418111723	2.98998524264960	5.68916277045056
H	5.38122805819657	1.78047539121734	4.40644981391709
C	11.43540530327833	5.00548550116614	7.95549842954381
H	12.11908016459012	4.61418659644819	8.69988604829893
C	13.63600195153631	8.22826198006334	3.89360677486501
H	14.33644219698584	8.18675147368804	3.05049091138233
H	14.20559330808229	8.02426074556528	4.80226126567885
H	13.25138140388573	9.24675971951194	3.94420643757053
C	6.51081146209024	2.50328321873240	0.42504753584644
H	5.74236175492253	2.13096523809141	-0.24281302444549
C	11.21009526809155	11.03823589769098	0.84535932230890
H	11.16484873854073	11.92777281949996	0.22737024659879
C	10.35823622247437	9.98179754982984	4.41089778899924
H	9.68325366678970	9.14186216114687	4.58332464324086
H	11.22982970541647	9.77965246227732	5.04566676528311
C	9.69310981654039	11.27302039423297	4.87252289483400
H	8.80348173332306	11.50008042188077	4.27889551094595
H	10.37085836314760	12.12782518557123	4.80233917890292
H	9.38363407624111	11.17985906408801	5.91566478915712
C	11.03785578957400	2.42676278901568	0.15564659883337
H	10.58940337058776	1.62509110540010	-0.43672623781820
H	11.23028321263052	2.03939568624165	1.15725408735897
H	11.99891082224472	2.68864106274845	-0.29443360745654
C	12.65163132337524	7.37993053121355	-0.92288801755366
H	13.41994786573251	8.10786017518682	-1.19671733318963
H	11.75740239149209	7.59354905755957	-1.51443026390565
H	13.01596877686031	6.39136355361306	-1.21136960255399

(^{Et}PDI)Ti(*p*-Tol)$_2$ (S = 0)

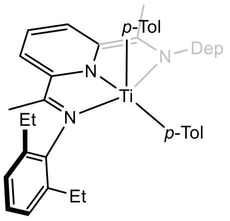

Ti	9.99729182243529	7.43957038778840	3.37990411605300
N	10.84666260763742	8.45876026184686	1.90629515229239
N	10.75907516565188	6.00772762373761	2.07447551578032
N	10.68210519528409	9.24802210604841	4.20841883676834
C	11.19219580743330	7.80932576376970	0.72322114938090
C	11.10616245412331	6.40985919752648	0.83914745688324
C	11.16635297960706	9.80324918554573	2.04137922733813
C	11.03695853593231	10.23813855574386	3.37565758172782
C	7.99580790616560	7.75821676116750	2.69666138728966
C	10.74686038642492	4.61173258396997	2.36933639132394
C	11.80505289073999	9.91645854684933	-0.28216889963648
H	12.15404483066639	10.48546221618389	-1.13393792541720
C	11.60096054186353	10.54023175672379	0.95147772459314
H	11.81126161053092	11.59599690794311	1.06808190939237
C	11.84141286987986	4.09418064920488	3.08585415463036
C	11.63301877124952	8.53730014124030	-0.37545089667410
H	11.86794880615023	8.02189761722589	-1.29838584746945
C	7.73171476948032	8.40001104338338	1.47185828983413
H	8.55765331949323	8.71555550335702	0.84383119070504
C	8.27804334893548	9.56361245025384	5.82495818658390
H	7.57367458550712	9.45197424383277	6.65450646971773
H	8.22208978462014	8.63364915292762	5.25049408349775
C	5.32865392975364	8.27406736129147	1.78565194799693
C	12.98447420258272	5.01115036548145	3.44854380592237
H	12.55975679063631	5.91233457595534	3.90202377957921
H	13.46777475516179	5.35700364652344	2.52646483937871
C	9.65045693984938	3.81171511385238	2.01802614740839
C	11.39589634688391	5.44140083444322	-0.25978262247562
H	12.10467225179380	4.68015853820297	0.07803832277254

H	11.81413654436965	5.94469301497398	-1.13007526927646
H	10.48913408417392	4.91507888524974	-0.57282620788790
C	10.79768846427662	9.50215779699730	5.60352482863392
C	9.66275097909258	9.69289759027775	6.39852835984182
C	10.30046037731779	6.51710606253193	5.21015361825794
C	5.56312058112379	7.63215505173161	3.00164165524080
H	4.71950405818847	7.31946988082357	3.61120584640542
C	6.85995073599130	7.38103454384928	3.43736422940737
H	6.97727035920114	6.87619864195764	4.39336515719325
C	6.43832917149912	8.64897677585708	1.02541114397826
H	6.28592823315974	9.14620177626585	0.07090319046484
C	8.45537115131945	4.38248586187072	1.28912822585305
H	8.45613204332229	4.01864998827430	0.25402582643857
H	8.54637340802482	5.46861880784149	1.23934710342095
C	10.74389220134644	1.93913675014375	3.09919122665373
H	10.74174168678538	0.89322613375253	3.38530008470692
C	11.81826160013827	2.75134964902519	3.44565522807284
H	12.64473693932472	2.33197596700305	4.00616633773731
C	9.36841063282069	5.55774925638259	4.82334449321848
H	8.78750290761560	5.68637421337854	3.89287470998078
C	10.99540927482470	6.28415609170021	6.40425678076486
H	11.70867586955728	7.01430530104023	6.77095863018916
C	3.92962210913000	8.57292377017572	1.31910771049368
H	3.20157348817769	7.92176380000880	1.80752864094803
H	3.83537644026149	8.44551061128752	0.23784073686090
H	3.65367927521318	9.60777328573423	1.54951096351273
C	9.84375147794187	9.95630286620616	7.75549368528692
H	8.96914641547269	10.10620441108031	8.38045308010653
C	9.67568779200441	2.46673697504750	2.39140994549943
H	8.83967381992661	1.82775943468858	2.12873316744991
C	10.78115630439891	5.12346414338754	7.12846388757127
H	11.34163608867554	4.94981202844605	8.04328076399642
C	11.27330210597690	11.63972469135699	3.83809923755070
H	10.42351938262261	11.99840403516021	4.42425538871911
H	11.41950238930507	12.31023272221890	2.99234312440350
H	12.15184419809863	11.70647608652850	4.48673380744582
C	7.82497014531873	10.71655917941587	4.92533718093634
H	8.43946276567508	10.78043664327683	4.02676955888978
H	7.87922348672736	11.67172197724415	5.45507311470904
H	6.79317682121579	10.55749506209348	4.60381122130435
C	14.02966409939783	4.42036277565174	4.38662550141973
H	14.54015847060321	3.56052221805182	3.94387929941073
H	13.57644618806433	4.09977070279653	5.32813653925798
H	14.78840155109909	5.17121412326028	4.61952308142344
C	9.85220145232615	4.15488598791171	6.70831033544958
C	9.12998766744122	4.38800279909528	5.54592474442151
H	8.40695604159411	3.65816063846755	5.19786865778445
C	7.11136912677683	4.03097122710951	1.93417082646391
H	6.29521886604886	4.51164559793085	1.39085567043981
H	7.07074636228306	4.38088046539421	2.96771226223459
H	6.92952743177533	2.95374202517216	1.93572514020306
C	12.09095076850739	9.49947758962966	6.15840657976240
C	11.11338593593618	10.01453749346916	8.31233536247280
H	11.23617728479248	10.22401195743059	9.36893956777967
C	12.22781476322244	9.77128683225203	7.51777168112332
H	13.21330592750101	9.77316415222115	7.96804640045550
C	13.26468032673096	9.12046300228942	5.28515975299498
H	12.96714566413543	8.24075718100973	4.70209725183183
H	13.44568812032408	9.90097663545004	4.53679175178180
C	9.65829598170995	2.89034703074003	7.49865399624502
H	9.40139996494158	3.10908618087816	8.53918980695496
H	10.57573637141449	2.29350985697811	7.50991913847037
H	8.86315680324428	2.27773705036740	7.07067124439123
C	14.56644154141188	8.83208005586823	6.02517406830726
H	15.33484967640169	8.51208234780896	5.31755208338085
H	14.43888344017235	8.03620939815289	6.76410965440521
H	14.94519015605185	9.71595181830510	6.54527771097534

(^{Et}PDI)Ti(*p*-Tol)$_2$ (S = 1)

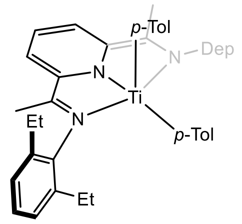

Ti	10.36816300914192	7.37625692412702	3.40799965903619
N	11.37304857649973	8.35032182225528	1.86437846544508
N	10.69335678836723	5.96229935776502	1.92226289061782
N	10.71842066120879	9.30728026133373	4.04501973188226
C	11.61181898110634	7.69267429806982	0.68780533569210
C	11.22658334864784	6.32606073770823	0.74351353048835
C	11.73339779112846	9.66267370597026	2.00478995005286
C	11.36366752908066	10.19123525476025	3.27233123716293
C	8.28263965888839	7.61420677578928	3.25003543247755
C	10.55701506832783	4.57033304939659	2.22439405995412
C	12.59307675468704	9.68595835438562	-0.24192042027162
H	13.06878125915872	10.20681324196122	-1.06459066911156
C	12.36678213854712	10.34885301741259	0.96117767443350
H	12.65668666142708	11.38297470323701	1.09272962076090
C	11.71239321471938	3.88666980483873	2.65819450499856
C	12.20980953992793	8.35717197674678	-0.39227798809505
H	12.37674186825400	7.83199192742089	-1.32316829816199
C	7.76467485951967	7.99044864809457	1.99974660347592
H	8.43137417910536	8.08210632544740	1.14437587365109
C	8.15818465626780	10.73348478281708	4.02914480851152
H	7.17113825790751	10.26608158592592	3.98720361733547
H	8.70274581437804	10.37606934383461	3.15735624264411
C	5.50803206611535	8.15822571341734	2.86932610953522
C	13.05141374541786	4.58048881428859	2.61351187633035
H	12.94710305904002	5.56338806743213	3.08112218121264
H	13.30105032392051	4.78737542554660	1.56528655603929
C	9.31902372293027	3.92445233808890	2.13127930369554
C	11.40248241361609	5.38967222296237	-0.40800283759295
H	11.37921611285356	4.35271110477740	-0.07282270423209
H	12.34738832565501	5.57116706833220	-0.92531872980095
H	10.59636357709186	5.51976700309245	-1.13985704220110
C	10.13386595430965	9.70699904854236	5.28487640055144
C	8.85802876027425	10.29545082050709	5.29081723871333
C	11.05349494111813	6.16241380488173	4.96230699988930
C	6.00756516607061	7.79981569408606	4.12213928473676
H	5.32705560151993	7.73045142177629	4.96653260943798
C	7.36180646511063	7.53613250883030	4.30538592464794
H	7.70023120760782	7.28181270964314	5.30638871759852
C	6.41344550385626	8.25643477268051	1.80952577079629
H	6.05293069758680	8.54595374096216	0.82595065410884
C	8.11945629319112	4.59768042362872	1.51161044824350
H	7.95010381797195	4.14886017658325	0.52405933364394
H	8.34844621463035	5.64793316534588	1.34128609565847
C	10.34144911034303	1.93701636540947	3.06778316255416
H	10.25195176557830	0.91435470006285	3.41713377702916
C	11.57786840728906	2.57364587281835	3.09185871049694
H	12.44654176859030	2.03889707860002	3.45532190181885
C	9.93574861278610	5.34472614867200	5.19707720322461
H	8.97993743386735	5.57322790219460	4.72554777074034
C	12.24638092917209	5.77494552490971	5.58555045540788
H	13.15253059473310	6.35687020781022	5.44231848863013
C	4.03874056887935	8.40724234350854	2.66018786612876
H	3.55517441249217	8.72642659081693	3.58613601249531
H	3.53123673400041	7.49674808518685	2.32259592556801
H	3.86929656073905	9.17484899690128	1.90124098805158
C	8.24664133165327	10.52564702974903	6.52385700567025
H	7.24866718511124	10.95138806286629	6.54210078723300
C	9.23293988927467	2.60150322240937	2.57179239262914
H	8.27970844100759	2.08759195999222	2.51451022111243
C	12.31967465006879	4.63581017513835	6.37594206187131
H	13.26381873246242	4.36322238877814	6.84013407755030
C	11.67622658678079	11.59341792226660	3.68272797624299
H	11.29206139082528	11.79792175592652	4.68138515784182
H	11.23653019784478	12.31687901625479	2.98937230536204
H	12.75817961948098	11.76273633002511	3.68500253080336
C	7.99595965573474	12.25732524623197	3.95311153553778

H	8.96302983434889	12.76423754520903	3.98706382256782
H	7.39720203045255	12.63585445742500	4.78526790596606
H	7.49574489921583	12.53968970576957	3.02295321234005
C	14.20749831473083	3.82502647056470	3.25711448230236
H	14.40668908836462	2.87703283413895	2.74992316260803
H	14.00098369503206	3.61239367930583	4.30834513024725
H	15.11963425687805	4.42429187111198	3.20583130333556
C	11.20064172054476	3.82197782063840	6.57984954295048
C	9.99950514016859	4.19978132838599	5.98555656485448
H	9.11351971398409	3.58764038489523	6.12330721088022
C	6.83427606773916	4.49076132851895	2.33369327532512
H	6.02575904599232	5.03544360928706	1.84141756661744
H	6.97074724239372	4.92642879994836	3.32420494470883
H	6.51533842936006	3.45268340572470	2.45667524866365
C	10.83330687557750	9.46783443170629	6.48308955010204
C	8.88844705961142	10.21763894845974	7.71267461473333
H	8.39032179807670	10.38761912238234	8.66071501866657
C	10.18430137609136	9.71531551016155	7.68916597142100
H	10.69693979992530	9.51621619948666	8.62219517276384
C	12.27766848514717	9.03652858237142	6.42958249577941
H	12.39766675783540	8.35444331777841	5.58785258473854
H	12.88533607106938	9.91841059498244	6.18414067138891
C	11.30403857115374	2.54924370020661	7.37406185231067
H	12.09127885910438	2.61165391590545	8.12872351186140
H	11.54308909524204	1.70556600859057	6.71664640862747
H	10.36238894411045	2.31517844396784	7.87547305605964
C	12.81993623249149	8.39428024541347	7.70172755313671
H	13.84718049740247	8.05716652933173	7.54351858326211
H	12.22193288411844	7.52636555384251	7.98767817060915
H	12.83222804893208	9.09565078325302	8.54005704317751

[(^{Et}PDI)Ti(p-Tol)$_2$]$^+$

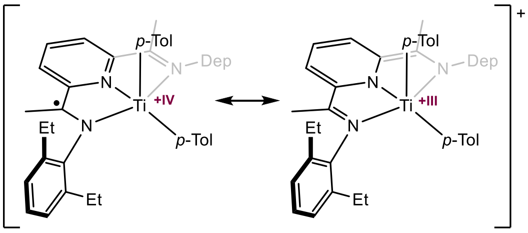

Ti	10.06254026424694	7.34501873763282	3.40228687767280
N	10.97832196847781	8.39805933294488	1.90604703486072
N	10.77489751514302	5.92166731899293	2.01021333094317
N	10.61643463189227	9.25936891858536	4.21630508845215
C	11.28498015471347	7.77104511840839	0.72964402944413
C	11.16649039053959	6.34177570304190	0.82226078058920
C	11.17385546485972	9.74234573046565	2.02698299212079
C	10.96958206100837	10.20738444127038	3.37566287588472
C	8.07421370842031	7.66548022353929	2.79629016090648
C	10.73193382421263	4.51588170066453	2.29332793183034
C	11.84212528028991	9.88257516606070	-0.27482756068529
H	12.16684661002619	10.46363955123380	-1.12797078379760
C	11.58888930111015	10.50675915391642	0.94795994574320
H	11.72652710925268	11.57402372112177	1.06123956480698
C	11.82523023777099	3.97827163248111	2.99626933117233
C	11.70773479635936	8.50097271555829	-0.37596602830821
H	11.93703336544823	7.99519158628666	-1.30430604497386
C	7.82371563789428	8.37212553986995	1.60301377393382
H	8.64543304349591	8.69640274826426	0.97435515804757
C	8.15738150983048	9.72680920508157	5.75509554812489
H	7.42753977473664	9.60139880197618	6.55919375874027
H	8.07849515568934	8.84107390448166	5.12140045185105
C	5.42083853216632	8.29638080438303	1.93706781871541
C	12.99977544434765	4.86408134578358	3.33751144359409
H	12.61418278834583	5.77776794065917	3.80446632016005
H	13.47311517059001	5.19537154464418	2.40480119985044
C	9.61582467180890	3.74995616887413	1.93565302771142
C	11.48224365751755	5.42192995124060	-0.30583245456794
H	11.92457422955417	4.49746026593405	0.06724394707472
H	12.16487593619359	5.88153076755923	-1.01886729126007
H	10.56431442983549	5.15388197216723	-0.83951456560951
C	10.68669472265418	9.54251520370761	5.62072300346692
C	9.52936125382567	9.77311475564601	6.37077700277857
C	10.41274861181816	6.46977174331590	5.19637259276860

C	5.64785134160231	7.59143879585633	3.12201604245678
H	4.80163324798486	7.27503616600534	3.72429922965045
C	6.93763523289073	7.28240079416692	3.53493982264080
H	7.05071071150875	6.73422477021054	4.46529487687918
C	6.53415932371681	8.67593982480608	1.18211541323665
H	6.38792131330259	9.21824511548056	0.25248288547020
C	8.44214051752691	4.34662334653760	1.19354435460843
H	8.47160298767619	4.00776252904236	0.15108225232816
H	8.54057331013564	5.43295618600207	1.17050568318624
C	10.67778015523627	1.84917862753309	3.00231636852308
H	10.65503520168827	0.80176578372710	3.28066922418120
C	11.77511165721379	2.63274035162740	3.34501589248915
H	12.59858732742675	2.18863489682049	3.88997093811104
C	9.46262689248566	5.51670660259259	4.84038347107869
H	8.86316937477933	5.62424450038349	3.91891196520321
C	11.12982427666681	6.26622062323848	6.38347602925518
H	11.86702103496314	6.98877223556221	6.71225017012183
C	4.02951032063626	8.65406468902702	1.49888144771099
H	3.28710581750034	7.99884904520727	1.95838516888393
H	3.92583264216685	8.59110320603361	0.41315092653470
H	3.78677238888626	9.68245490624485	1.78839951129042
C	9.67722435474508	10.04171798650902	7.73254446162248
H	8.78867208175452	10.22509358441175	8.32729301644548
C	9.61532201174330	2.40227179442286	2.30426197901680
H	8.76781822197135	1.78057226812140	2.03973397849988
C	10.89673077038284	5.13724721592929	7.14695588209101
H	11.46272587863262	4.98279940635506	8.06070555212194
C	11.21126456929397	11.63020436268293	3.75483554559179
H	10.80130555003456	11.85046885754859	4.73782573257993
H	10.75664531373543	12.29829850019145	3.02043570517788
H	12.28379506016930	11.84783153926161	3.77356938876500
C	7.78061333602731	10.95742771617224	4.92494340345231
H	8.43263537367017	11.07398721946588	4.05762506395289
H	7.84218168032205	11.87106000026974	5.52187906492185
H	6.75660194847703	10.85953078203582	4.55550512204062
C	14.05394871609361	4.24311547015765	4.24477535632846
H	14.53888895037472	3.38179911434206	3.77791718538203
H	13.62445912777546	3.91672003837515	5.19517783653824
H	14.83187616842917	4.97800013140368	4.46565225753741
C	9.93900615046629	4.17349967246643	6.77089927897615
C	9.21220497620178	4.37508838238355	5.60430553163814
H	8.46652836733240	3.65256011149625	5.29370406751379
C	7.07957733181950	3.98720946658064	1.79084935565038
H	6.28331674949754	4.49007082492761	1.23707446560215
H	7.01012397411472	4.29986130690738	2.83529856342108
H	6.88603092377115	2.91330487237369	1.74721307816612
C	11.96710254917157	9.53569235360618	6.20437190079770
C	10.92980871738044	10.06899369990157	8.32709422917053
H	11.02491627262441	10.27934989524938	9.38611001617318
C	12.06430855399699	9.80784967218301	7.56700974716398
H	13.03589889133620	9.80479901762711	8.04494979229179
C	13.17744493773261	9.17992722200593	5.36880787091803
H	12.93549238512634	8.28341585754066	4.78506925316824
H	13.35760353624661	9.96650743193904	4.62709158906090
C	9.71988219744064	2.95484404168844	7.61954696514396
H	9.44224562958328	3.23370143803749	8.64026590067509
H	10.63574300197499	2.35968107736734	7.68773933530113
H	8.93153298828595	2.32211546596474	7.20944578102087
C	14.46618300338316	8.94215917025920	6.14750013524997
H	15.26228841655426	8.64252954583135	5.46168758843826
H	14.34974136212271	8.14845056075957	6.89050332040942
H	14.80185364013013	9.84374651334615	6.66595376609912

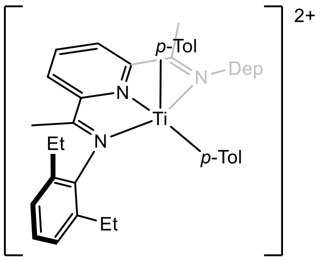

[(^{Et}PDI)Ti(*p*-Tol)$_2$]$^{2+}$

Ti	10.20947578595825	7.24769604620684	3.40843475353651
N	11.21264648837357	8.31301175619568	1.83278820066698

N	10.81335224158572	5.80135812143203	1.90690319975234
N	10.57668975430010	9.29428098702430	4.08823691824368
C	11.53330416972541	7.66414021704317	0.70643090601295
C	11.33278970186395	6.20463075220985	0.79459920437958
C	11.36077483093564	9.64038312036996	1.92313109561784
C	11.02415093242305	10.17546337837331	3.25758573895889
C	8.20357087089965	7.44569351596353	3.11672401681063
C	10.62446382475071	4.38998002327846	2.14415528966126
C	12.15191893929874	9.72624028121047	-0.33074295506669
H	12.51760172378335	10.28363812534665	-1.18395952441692
C	11.82903695858491	10.38833747410807	0.84885262320191
H	11.94206517194104	11.46050158824831	0.93271814329767
C	11.66313299063313	3.71424415829210	2.80602119962271
C	12.01364968804808	8.34470366703767	-0.40703100836352
H	12.27156349549747	7.80916626116607	-1.31040154792021
C	7.91191667894872	8.14829454698577	1.93268252752975
H	8.69860654043076	8.42771257430780	1.23800450314311
C	7.94660114483598	10.16272107097657	5.15167779180107
H	7.09716289693288	9.63315694548835	5.59003505922139
H	8.09758633911748	9.74756114071849	4.15637473801869
C	5.54614606561566	8.18449487767434	2.45848469606244
C	12.94364273298220	4.44134127611094	3.14247754312007
H	12.69021023265439	5.38053194136690	3.64884458368912
H	13.43284103460338	4.73787778066272	2.20569239132611
C	9.43069906309100	3.77377096981004	1.75565412135029
C	11.72494621019149	5.32935937660752	-0.33744676964833
H	11.63527859606402	4.27799692098331	-0.07661802533077
H	12.75033007162113	5.54731900332477	-0.64696915082888
H	11.07670904208305	5.53619898030419	-1.19612999986265
C	10.42022676485909	9.62766814004629	5.48171032345590
C	9.16358967132271	9.93295553086249	6.00921754159034
C	10.78213851412832	6.42717159797793	5.12453776278225
C	5.82967244074817	7.47831220686968	3.63671835879999
H	5.01689782355323	7.21327100178755	4.30531028448775
C	7.12547974148471	7.11382092403733	3.95805276624458
H	7.30047922517098	6.57621000793504	4.88367148570982
C	6.60898859833726	8.50939117850175	1.61044744346945
H	6.41184619564160	9.04868805117566	0.68984521394128
C	8.35519237390005	4.51004184036457	0.99097967302016
H	8.40908200043088	4.20726871243037	-0.06173243862238
H	8.55628736910100	5.58181998236380	1.01191504021907
C	10.28041104305825	1.73035072036109	2.74610893276283
H	10.14325677920407	0.68205845435189	2.98404702769236
C	11.46288486592112	2.37045857604239	3.10284330306923
H	12.23798738126929	1.81402082420392	3.61366403379278
C	9.79802075237326	5.46443178715502	4.90743049623616
H	9.11310429120394	5.52456288366368	4.04613653295088
C	11.61514289694409	6.28162567792461	6.24398074629937
H	12.39257755054025	7.00012919057690	6.45966351266803
C	4.13692726579070	8.56716246602490	2.11986712061888
H	3.51609548427628	7.67542375124714	1.98905045661374
H	4.09020410607150	9.15403193650066	1.20195620620443
H	3.68756716817368	9.15063420899914	2.92857127450787
C	9.07822848846487	10.12275840266406	7.39190899380286
H	8.11208007281258	10.35073284185289	7.82877258100274
C	9.28255179991282	2.42182941563688	2.07683917624872
H	8.37459438764939	1.90600308987001	1.78866321563967
C	11.44510261928424	5.20158014859897	7.08556767185348
H	12.09395460979809	5.09194271991863	7.94857321058808
C	11.24358276380175	11.61367610772159	3.55413659481815
H	11.01401470566608	11.84713666067691	4.59041653573579
H	10.60555181668854	12.21656840976311	2.89947580928206
H	12.27821367541962	11.89352456057108	3.33708190097818
C	7.59769843936690	11.65184177022516	5.03253538635372
H	8.41173495335364	12.21565052009550	4.57017366986821
H	7.40141290434112	12.09277987562944	6.01236505571087
H	6.70350323218461	11.78202904904190	4.41780078049016
C	13.93683171575799	3.66339838267268	3.99651034108121

H	14.29466266137412	2.76706402861392	3.48466471581794
H	13.49604693128502	3.35895206831798	4.94859755035399
H	14.80652900977624	4.28706715590299	4.21446021713747
C	10.44809858737326	4.22788967599630	6.85803063311180
C	9.61836501227476	4.36857105593917	5.75290877475237
H	8.84742454164976	3.63639891335576	5.54479067403051
C	6.93706066053686	4.26059882472887	1.50659595415361
H	6.22400303969004	4.87406305659668	0.95149767557823
H	6.85025710260005	4.51338720519916	2.56531349454635
H	6.63814599933220	3.21795754236333	1.38339829940321
C	11.58969836511844	9.59119085697364	6.26619369055810
C	10.20015596313687	10.03759901757611	8.19839538564165
H	10.11142723299931	10.18037810935990	9.26884182623330
C	11.44975560489154	9.79386242294065	7.63435732345060
H	12.32419982653884	9.76286152012070	8.27157010061004
C	12.93879366627816	9.36572296919840	5.61745884783048
H	12.88166620418324	8.48418678317619	4.96723643800300
H	13.14743267312373	10.20520598010634	4.94358628804963
C	10.30341223621815	3.06790024742379	7.79570750578402
H	10.11612605318453	3.41617957729801	8.81562464125290
H	11.22494513346448	2.47852660577525	7.82465724140590
H	9.48423894533154	2.41274043391632	7.49820370147908
C	14.11556668015373	9.22226201987169	6.57577767171157
H	15.03188693454374	9.03713075209486	6.01116972120684
H	13.97961127422788	8.39099315962019	7.27274773789362
H	14.26740895492555	10.13044153235872	7.16328960444570

Biphenyl

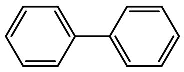

C	0.49675288587499	1.26783198024926	-0.37683811489388
C	1.80881876277606	0.82243961229432	-0.37140794315325
C	1.07752062431740	-1.32111300554834	0.41276831736694
C	-0.23452070798720	-0.87562023513766	0.40581267473363
C	-0.54696879975782	0.42633233180365	0.01147422159215
H	0.27501119895043	2.27611847356597	-0.70830776841886
H	-1.02676826053604	-1.53993425831475	0.73257453129752
C	2.10558989341312	-0.47416313195583	0.02381170731298
H	3.13215899403672	-0.82261004084739	0.02856045255206
C	-3.56875086676757	2.65827715827965	0.36007187874027
C	-2.25662707937583	2.21299434954388	0.36421610040723
C	-1.94424851428507	0.90077602777512	0.00544176524249
C	-2.98811686215604	0.04879587668819	-0.35889293507498
C	-4.30025506688072	0.49395437324130	-0.36461247413584
C	-4.59696229725661	1.80084410501510	-0.00475580223136
H	-5.62359387117966	2.14909982468747	-0.00859996169409
H	1.29848432560458	-2.33385941442989	0.73125062357639
H	-2.76642022651936	-0.96819337462554	-0.66266460242626
H	-3.78964151632997	3.67928898027269	0.65101425444762
H	-1.46425520563607	2.88608024748916	0.67216674463024
H	-5.09497267214076	-0.18236586366783	-0.65942883100214
H	2.60339526183542	1.49026598362148	-0.68534483886887

I_2

I	-6.11994589997836	3.31385000000000	0.00000000000000
I	-3.45927410002164	3.31385000000000	0.00000000000000

[(^{Me}PDI)TiPh$_2$]

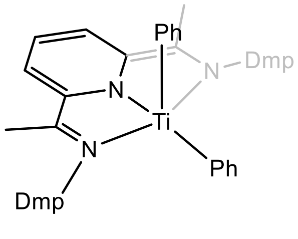

Dmp = 2,6-$Me_2C_6H_3$

Ti	10.00317025190598	7.44330785066128	3.37427227335459
N	10.89564373802132	8.45343619433835	1.94575127599117
N	10.73212167684847	6.01657088904553	2.05326008086874
N	10.64561847162283	9.24318857965853	4.24488072571143
C	11.27427186237287	7.82280447189967	0.76825919657453

C	11.14849762866690	6.42712305800254	0.85070704044613
C	11.17345936531876	9.80280907358785	2.09506409167114
C	10.98936471804494	10.23554476486679	3.42098659071714
C	7.99104933058632	7.73818146293707	2.69997224752090
C	10.67569819559847	4.62248779913513	2.32526054411465
C	11.90295575673089	9.93895173822890	-0.19415985704558
H	12.27792305899677	10.51831375430787	-1.02780253767627
C	11.63455789642446	10.55089028059070	1.02869331436011
H	11.81596251494165	11.61074550946575	1.15642959229006
C	11.79464058690242	4.03267657255317	2.93568449048640
C	11.74852064443008	8.56254994468389	-0.30310032071801
H	12.01860955712993	8.05781073672254	-1.22246934404293
C	7.75365605332997	8.29396548682817	1.43329848700264
H	8.59145550462632	8.58188969427544	0.80624026901805
C	8.22135819124655	9.82072991044791	5.72881486035245
H	7.45835973290702	10.13674674305435	6.44182272145239
H	7.91818469914612	8.85346383788984	5.32315197663139
C	5.36779772789643	8.13433706835450	1.69801489401522
C	13.03248310447188	4.83130846312734	3.18686545920906
H	12.80226814369727	5.77236090769718	3.69063569332371
H	13.53520291230140	5.08971429762418	2.24891451711079
C	9.53378152254335	3.87123777541157	2.03353713411667
C	11.44585721338446	5.47990302237837	-0.25453831968717
H	12.16025801944213	4.71723501234849	0.06787824117318
H	11.86071585798211	5.99182591433411	-1.12089947803375
H	10.54170984915044	4.95234439251413	-0.57394085628727
C	10.71373086041791	9.48462378014813	5.64157463090238
C	9.55989056979417	9.74354698724363	6.38711466464259
C	10.43419362899718	6.32901964788824	5.09766182110722
C	5.56186495635121	7.58788512572464	2.95853962766098
H	4.70771381704397	7.30791066395461	3.56704862123257
C	6.85007972245327	7.39580641152866	3.44149107135234
H	6.95089262301067	6.96634942050668	4.43505508546517
C	6.47116424771098	8.48881428388497	0.93544525675023
H	6.33342889190610	8.92090558030952	-0.05089281337282
C	8.34258212972262	4.45476435324063	1.33791618148249
H	8.16961457265296	3.94730544383844	0.38382486910275
H	8.45291585862858	5.51881723865699	1.14103402595975
C	10.59556076809049	1.94060820046637	3.03141108097822
H	10.55779023237808	0.89581516200817	3.31897743763147
C	11.73244837720665	2.69244065710902	3.28795770418897
H	12.58846167957308	2.23521080639651	3.77313267187702
C	9.31362652386925	5.49724707529035	5.07666798976611
H	8.56918481266244	5.59031348734682	4.27956064673858
C	11.35781806148689	6.12420488964586	6.12521045466843
H	12.24693431973058	6.74115984066704	6.19461834700594
H	4.36427337404854	8.28382484525605	1.31415187219416
C	9.68539810157878	9.93651448902437	7.75875476049413
H	8.79357391971019	10.13843036203013	8.34306321003588
C	9.51491393178105	2.52709103275125	2.40140512569639
H	8.62792677489663	1.93910243271130	2.18670689060814
C	11.16825825481797	5.13385138308438	7.07508839104400
H	11.90159115340447	4.98969216404217	7.86220409947691
C	11.16037449736418	11.63795274488928	3.88281474105146
H	10.26685926391043	11.97665287460734	4.41488339945632
H	11.33660206508734	12.31465703290417	3.04874094360318
H	11.99671267320263	11.73311889351218	4.58119434940251
H	8.21777352435122	10.52216700375252	4.89044165314206
H	13.73727443771372	4.26885162162822	3.80078040418018
C	10.04015460087765	4.31871725043348	7.02681901395796
C	9.10414126099405	4.49786191892291	6.02359129841632
H	8.22542644289272	3.86405932613861	5.96970426954130
H	7.43542332376634	4.32052436741346	1.93355044078727
C	11.97437609859742	9.43447699733500	6.25298855066520
C	10.92149299454865	9.87926375936455	8.37967681552845
H	11.00010501607107	10.02719193623178	9.45087297492983
C	12.05683574304873	9.63239996054953	7.62599304156870
H	13.02801678848106	9.58270118656884	8.10787384254458

C	13.20473801319333	9.15445956925845	5.44874440167689
H	13.04420727574961	8.33406326272652	4.74439339532228
H	13.50638016982772	10.02112673543332	4.85154786823038
H	9.89749687049579	3.54364702165828	7.77199185558557
H	14.04167298923072	8.89936556494378	6.10072801169712

[(^{Me}PDI)TiPh$_2$I]I

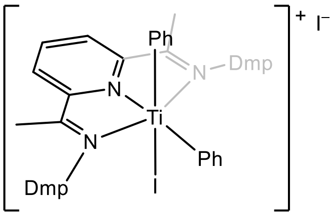

Dmp = 2,6-$Me_2C_6H_3$

Ti	10.79437555549438	7.08299629838011	3.67969390864379
N	11.24437477746444	8.26641896146266	1.96909605092559
N	10.82879236350202	5.68103826727284	1.93751512375220
N	10.85557996303033	9.25750592311477	4.31136426213459
C	11.41038184578055	7.64742785581354	0.79822067889067
C	11.17619664008487	6.20224174304196	0.80762283646928
C	11.35207783198388	9.58997307700173	2.05446649981398
C	11.10486981013433	10.12622633132677	3.39131729449515
C	8.75749396915232	7.39673831256942	3.08275739839034
C	10.37811712706953	4.31997079407072	1.95714301322238
C	11.89340870561347	9.73104436432568	-0.26436015125086
H	12.17274126396095	10.30173988088110	-1.14070147699242
C	11.68181853746260	10.36084946334095	0.94474345771687
H	11.77431520552705	11.43461337843052	1.03232537619909
C	11.19488445747099	3.32237822083355	2.50911548038635
C	11.75366087342712	8.35395839643752	-0.34907736081959
H	11.89184787292136	7.84427273826742	-1.28961224858544
C	8.25965595220923	8.35047430079537	2.18883784508254
H	8.92689378278677	9.04818856611234	1.69513987472167
C	8.24084752168294	9.45021757124334	5.57974798399883
H	7.38858321185396	9.86506659040150	6.11982312084167
H	8.09993295041786	8.36993916984138	5.50956195210058
C	5.99483311075784	7.59568044587471	2.48713816977820
C	12.59757857809684	3.59493026171743	2.94056760462026
H	13.14054440583928	4.19257827120621	2.20583821553673
H	13.13414437449488	2.65633787295247	3.08480049880433
C	9.10718450843877	4.01979288093302	1.44401645633358
C	11.36431583930109	5.45642318568137	-0.45338843133369
H	12.38664674422449	5.65618338231986	-0.80922919444994
H	10.68114951952090	5.81835111576955	-1.22594269539339
H	11.22629108781754	4.38679118241924	-0.32145501610250
C	10.76121523010540	9.72274690699718	5.66289787963216
C	9.50977211468781	9.77975716819863	6.29189046644416
C	10.66292326571711	6.57885205080326	5.63690956708824
C	6.45351991400117	6.63591182653835	3.37467464342817
H	5.75570395997297	5.95434536195828	3.85022660570394
C	7.80873339204045	6.54154200962002	3.66504267357925
H	8.11018966491084	5.78183815399634	4.37650166277736
C	6.90792214305295	8.45611602526641	1.89403444261099
H	6.56662933713935	9.21392309724275	1.19623739432330
C	8.27232781676205	5.00208712152292	0.68422802502127
H	8.46750095385153	4.90518139749357	-0.38916078047896
H	8.45893404742988	6.03603171364176	0.96433250570262
C	9.40117209332824	1.73402867689361	2.18031334908649
H	9.01384211362995	0.72658161551097	2.28142147903263
C	10.68065186600846	2.03490480358647	2.61331482545080
H	11.31033938479013	1.25793113235236	3.03318529831678
C	10.38788643580564	5.28506629908107	5.22553011434282
H	10.27108101232897	5.01396613973928	4.16410302830837
C	10.80589997515826	6.82450694870832	7.00243551894269
H	11.04269992991610	7.81516571400166	7.36840044698324
H	4.93759990951030	7.67403013637990	2.25906496271208
C	9.45573977227382	10.16774509952374	7.62468194723810
H	8.48764024311901	10.21544112423040	8.11154955709578
C	8.63323904158861	2.71866184642270	1.58881339262531
H	7.64417775357355	2.48348257488260	1.21028152572327
C	10.66098769349301	5.78731587074776	7.90488820433696
H	10.77746432767625	5.98001291627751	8.96597554795916
C	11.16199528830400	11.59650949571022	3.59050039904857

H	10.83470537328757	11.88249102621871	4.58631284284469
H	10.52767430558064	12.09179323320584	2.85142778318421
H	12.18252236955653	11.95777177407246	3.43572593168458
H	8.22516742596674	9.83453002923799	4.55974970582620
H	12.63731940725604	4.14962628167390	3.88120350273258
C	10.37575542628632	4.49229130365547	7.46867545466953
C	10.23779970645565	4.22749688164419	6.12032819980060
H	10.01974544888598	3.22800954038113	5.76322095561132
H	7.21209246251373	4.79623783128508	0.83510667485790
C	11.92791547334709	10.10847002120775	6.33790128349385
C	10.60375165203509	10.48809227562763	8.32866154740285
H	10.54401525598345	10.77249924145496	9.37299650136673
C	11.82290594118800	10.46526990457293	7.68116017977141
H	12.72659378365594	10.74394151896054	8.21307817120054
C	13.27414857386351	10.21040681994441	5.69128683209488
H	13.32586080465935	9.73427466406528	4.71578292359639
H	13.55457271871257	11.26272973565953	5.57721804804773
H	10.26797403844113	3.69071830751801	8.19058683124835
H	14.03605562959972	9.74358554010368	6.31857271974261
I	13.53132934796005	7.02274918141960	3.81939042526722
I	14.84704778706413	7.16280285692228	-1.06164772541130

[(^{Me}PDI)TiPh$_2$I]I$_3$

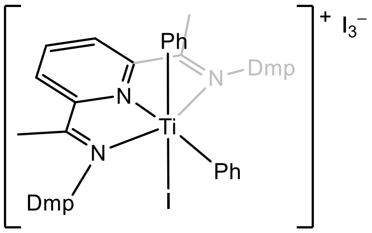

Dmp = 2,6-$Me_2C_6H_3$

Ti	10.80787264616164	7.18886167398236	3.64130746765998
N	11.24611432492890	8.44410517695699	1.98272840857457
N	10.93770728620924	5.85135869458668	1.87073999240953
N	10.78476505826287	9.34476633566296	4.34812953686601
C	11.48065647702651	7.86501866633951	0.80330725979738
C	11.30495063615625	6.40688708513756	0.76677247643947
C	11.27254902830984	9.76918031809992	2.10561533845044
C	10.97598008529982	10.25450699781772	3.45692207041862
C	8.78240453919071	7.47214873589905	3.02262703505501
C	10.50075934826497	4.48331393455203	1.84649524095225
C	11.86942099771131	10.00083460298217	-0.18995578071663
H	12.12625120966087	10.61323205024749	-1.04535268617160
C	11.58695768252385	10.58808372555205	1.03043242839195
H	11.61224134125692	11.66262247235971	1.14777735392017
C	11.29808811241100	3.48840209189826	2.43038624952665
C	11.80681696869449	8.62386062488594	-0.31215640880483
H	12.00065016109528	8.14170853785197	-1.26024890490207
C	8.25494224794289	8.46165088544182	2.18720122293518
H	8.89655536042999	9.21531314077110	1.74412457407607
C	8.21651177278654	9.20107004327549	5.73441597049007
H	7.35084601119197	9.50715637027608	6.32351035934993
H	8.19169947241963	8.11287314829248	5.64839190808863
C	6.02041450995010	7.60199999390181	2.41432568145849
C	12.68025315724243	3.76792707568099	2.91891218010818
H	12.69240130219378	4.45194187882876	3.77029821464938
H	13.29304178385847	4.22550264501923	2.13819617622676
C	9.25838607639825	4.18611750636998	1.26822743860605
C	11.52771565234828	5.69251054121766	-0.50805425576073
H	12.51412487654383	5.95120636949747	-0.90240288958054
H	10.78834778172527	5.98647543288401	-1.25842917655495
H	11.47708684291853	4.61381420936356	-0.38149798830671
C	10.69459708424284	9.75390330202254	5.71772916856168
C	9.47336433392855	9.65320418800457	6.39856603282930
C	10.79477636253952	6.61323243660595	5.57289297683809
C	6.50924683239601	6.60573369674529	3.24276455074831
H	5.83606249588425	5.86670386097745	3.66430369227795
C	7.86473133011509	6.54417786272443	3.53952142094781
H	8.18811157207088	5.74958789289636	4.20088862722977
C	6.90305361986341	8.53368186930794	1.88596350506753
H	6.53755485523351	9.31937702127005	1.23294301269299
C	8.44160737818189	5.17249314325919	0.49608422690455
H	8.63547125993145	5.04554330577155	-0.57473432459592
H	8.64154362205709	6.20773202626650	0.76372220947074

C	9.54154793610532	1.89241207659990	1.98174614693755
H	9.16665445536274	0.87692604020669	2.03314101738509
C	10.79203327341773	2.19685354933765	2.49110007211193
H	11.41167748126298	1.41668148448958	2.91770361973015
C	10.46712147177653	5.34235677825764	5.13226137303008
H	10.28846358567620	5.10739771697714	4.07010859033778
C	11.03087604751490	6.81002808039475	6.93381462554455
H	11.31352167110635	7.78348598173698	7.31413867149782
H	4.96310044042490	7.65250414767368	2.17961382907222
C	9.43833233128318	9.99219668971188	7.74504024460311
H	8.49297022787306	9.92214313653308	8.27206476930652
C	8.79509358797522	2.87674082397585	1.36562569735849
H	7.83593093817938	2.63477516801890	0.92138741816431
C	10.91884707999772	5.74477868375284	7.80587512866937
H	11.10565264108623	5.89514779752291	8.86367000106772
C	10.92435042858116	11.71914680371820	3.69077941160111
H	10.56129053234581	11.95320753131001	4.68806166083667
H	10.26226793417868	12.18102294420134	2.95396564145947
H	11.91452292954418	12.16492311813828	3.56249257282042
H	8.10641397715815	9.59883409737414	4.72558767506460
H	13.16551015872246	2.84001161383318	3.22078960082846
C	10.57871614632727	4.47122562025881	7.34238410415065
C	10.35300890381025	4.25541244591126	5.99800851031168
H	10.09720276228502	3.27333419920952	5.61888285270168
H	7.37809665921614	4.98497728897382	0.64797393162078
C	11.84686900660382	10.22918544645564	6.36193457489155
C	10.57618515421349	10.40839994063106	8.41408293993781
H	10.53208111960951	10.65187944327777	9.46939449115356
C	11.76349676874846	10.52869533489717	7.72037519041678
H	12.65732341076962	10.87550367068333	8.22813186442835
C	13.15539737277287	10.47241520332765	5.67583082965851
H	13.23110010765419	9.99979630032297	4.69959628233416
H	13.32088882111248	11.54792043448665	5.55342898168451
H	10.49960950939913	3.64663205529806	8.04163872423648
H	13.97842814725896	10.09049470508229	6.28271934396305
I	13.58127120666372	7.29494040668453	3.77466618345400
I	13.82822835386490	0.77124191353082	0.26910046783759
I	11.71409304634605	1.88220664158880	-1.36223255976510
I	9.56936087620814	3.11457311412762	-2.95794607307045

[(^{Me}PDI)TiI]I$_3$

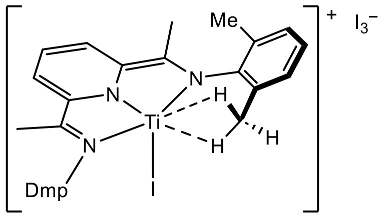

Dmp = 2,6-Me$_2$C$_6$H$_3$

Ti	10.93816987275080	7.62830302622567	3.02946600156616
N	11.99772091687448	8.60549283195493	1.74100216697093
N	11.06883667780370	6.41538454629876	1.40255253041388
N	10.80578015194869	9.55307886794240	3.56620596125260
C	12.51491728466925	7.97055280444069	0.62624841566001
C	11.95086090754029	6.66965273736137	0.46250611439134
C	12.25219152881852	9.95696158002443	1.89999156904537
C	11.51828446469800	10.50405044974172	2.96157274200739
C	10.25600334044100	5.25214879189895	1.47463499396372
C	13.72667286744630	9.94911034991535	-0.00180245047319
H	14.41441279338478	10.45562106562484	-0.66574343864812
C	13.12577004769630	10.62214992804794	1.03905048657818
H	13.32075419415413	11.67548118930160	1.20184279567939
C	10.74860030766647	4.06637861415010	2.03841524993952
C	13.38870217535292	8.60879754865075	-0.22174005980347
H	13.79113795297104	8.07428718786836	-1.07266625080709
C	8.41597188145547	8.18155508782767	3.38584377276572
H	8.90691742161888	8.57929220754470	2.48162899403577
H	7.35118693249619	8.20296212720428	3.14960241709008
C	12.18068644285713	3.87954260878592	2.42548877556335
H	12.26024685910752	3.45243230152083	3.42753963125491
H	12.75286307240640	4.80477957778540	2.41066245373072
C	8.92611994585914	5.36666857060932	1.03158049784499
C	12.26658888846599	5.72712472092416	-0.62528000217954
H	12.76183743140776	4.83522733261546	-0.22790069048806

H	12.91146166426601	6.16904265651272	-1.38116392709259
H	11.34765290971075	5.37219614916569	-1.10132971719910
C	9.98319793789253	9.65543036507014	4.69736451299738
C	8.75181708364138	8.97549289172226	4.61043522027828
C	8.43954946643422	6.57361344670749	0.29334799962906
H	8.54065474106111	6.41072729716760	-0.78501123322791
H	8.99610041934373	7.47874359494660	0.53654251704164
C	8.54389429898736	3.10803103037943	1.78381575340677
H	7.87484046466423	2.26328671310114	1.89954710707907
C	9.86366126989059	3.00222066196260	2.18058296001181
H	10.23208231617807	2.07184870693763	2.59872958499870
C	7.88253980922743	8.99376541845906	5.69126602123578
H	6.92801676409699	8.48478235445633	5.61494206063579
C	8.08204520578623	4.27962521475491	1.20702511377221
H	7.05995395802120	4.34563228976198	0.85180265883182
C	11.46801883093916	11.95126546664899	3.29110708732060
H	12.36776437119206	12.28417787281968	3.81439832257364
H	10.60634250797375	12.17698065160410	3.91878590158560
H	11.38461687801134	12.54058564950157	2.37597347933026
H	8.62594468893063	7.10401042430305	3.51932505620520
H	12.65354754862018	3.17451403125134	1.73395179719935
H	7.37914967966912	6.74720050390277	0.48302623320119
C	10.39146604931072	10.23587657162362	5.91298494541916
C	8.23476538425998	9.64953528099294	6.85647293657402
H	7.55092529809307	9.67009848796402	7.69683673698427
C	9.48471700004613	10.23867340701376	6.96481377445842
H	9.78464097600281	10.68919850817959	7.90508332361952
C	11.77551823967802	10.75549313066957	6.13978748939492
H	11.83234281572554	11.84130594359240	6.02806026456428
H	12.08932835269069	10.52178964692002	7.15857479427579
H	12.49780701121206	10.30529994343626	5.45804593461793
I	12.62640050589550	6.85082257331763	4.86372069688375
I	12.33632687352467	1.76435431420300	-1.21352316977681
I	9.80236260772013	2.96785940936231	-2.03014001343865
I	7.28386171140906	4.22029333732054	-2.78491490074629

[(^{Me}PDI)TiI$_2$]

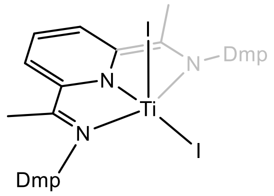

Dmp = 2,6-Me$_2$C$_6$H$_3$

I	0.78707703921964	5.28907874562406	11.14958326949359
I	1.01363807671880	9.44255817686468	9.55496111032084
Ti	1.31061872730216	6.86157754130237	9.05100270744075
N	1.85319906926250	7.11860724206745	7.19930393478444
N	-0.32068382738814	6.28433652880378	7.90850269015382
N	3.35686140246009	6.51019148437589	9.01545369673072
C	4.10419301906945	5.95404192417389	10.09688831744664
C	3.18485371836286	7.27792894644803	6.87233100034997
C	4.02870921835339	6.93527735966894	7.94862334162135
C	4.36616599538574	4.57816787871006	10.05417386487562
C	-1.46906098798150	5.60790347028957	8.41855128868914
C	-0.33727790646351	6.66365255049607	6.63249613278070
C	0.91370678312741	7.13645993872381	6.18742356986039
C	4.54919660058453	6.74912405224676	11.15538427708843
C	5.25582078328327	6.13336918195576	12.18483205861446
H	5.60619077060834	6.74070097575873	13.01288785722805
C	3.55638693346891	7.65476942167299	5.59347692756989
H	4.60352006167308	7.80157458099881	5.36120537011414
C	-1.50376507836230	4.21363726404718	8.28784929059188
C	5.51366523592351	4.77481159408275	12.16673823786456
H	6.06156846108590	4.31451741581459	12.98122740031010
C	5.07220238597156	4.00483704338625	11.10284511429706
H	5.27199846867828	2.93875345695063	11.08207573109660
C	5.51128284496409	6.99570557155608	7.90668970493582
H	5.89461667068708	7.64027170946404	8.70213417562621
H	5.87024665424523	7.37749141080693	6.95327425749379
H	5.94412816303323	6.00444753751872	8.06795181891846
C	-2.51088422625694	6.31086796700474	9.02784026184785
C	-2.60008639260085	3.52828782525338	8.79355458873293

H	-2.63192315494836	2.44758211699649	8.70432759484592
C	1.26981888875288	7.51233891753483	4.90319257684028
H	0.51647728767357	7.54741576220326	4.12658783458995
C	-3.63926493160835	4.20627709477966	9.40991353790299
H	-4.48828739510680	3.65872720305011	9.80336321518992
C	3.88138694595839	3.74582526365311	8.91195886180394
H	2.79915051264804	3.84505191994036	8.78876042702595
H	4.11397477062757	2.69301075060958	9.07587405539282
C	-3.59179635662986	5.58441098990202	9.51912090147395
H	-4.40672411771650	6.11945205443032	9.99527607809798
C	2.59313424931206	7.81006330208698	4.60417726641448
H	2.87748010370710	8.10160056182939	3.60171640330507
C	-1.54753148089576	6.55089729646671	5.77992284900409
H	-1.87781090989729	5.51049177978237	5.71235385022151
H	-1.36582753971632	6.91988896093665	4.77267253474016
H	-2.37806698548327	7.11781571172932	6.20897271639419
H	4.33610815327870	4.04847818153129	7.96364398058573
C	4.26543735804367	8.21478306170137	11.21979050886800
H	4.96082498578841	8.70878002638229	11.90044373961500
H	4.33484068847528	8.69869741306761	10.24460064952157
C	-0.37844030018135	3.48169811782919	7.63140094252247
H	0.57319434717443	3.71002263209090	8.11965169153422
H	-0.26773593958248	3.76147367486383	6.57914874178176
C	-2.47503123870350	7.79690762085507	9.17911792586072
H	-2.10879283293259	8.29804272816257	8.28137834553917
H	-3.46997361997675	8.18258403177798	9.40686646517525
H	3.25251742876262	8.40161951201775	11.58869915084979
H	-0.53951966339074	2.40406488581944	7.67812351099482
H	-1.80870791784885	8.09247963190195	9.99466164702917

Transition state for reductive elimination $[(^{Me}PDI)TiPh_2I]I_3$ → $[(^{Me}PDI)TiI]I_3 + Ph_2$

Imaginary frequency = -338.32 cm^{-1}

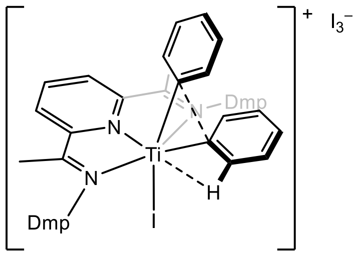

Dmp = 2,6-$Me_2C_6H_3$

Ti	11.19036986419498	7.24717886543662	3.49792503888712
N	11.15783420199070	8.51325232287955	1.83299018462643
N	10.98457810668317	5.94251946650789	1.72598490752347
N	11.00591794775330	9.40356205163315	4.25114161786990
C	11.26124398823377	7.96598409588195	0.61352409979079
C	11.13793876446383	6.51688611003212	0.57498968357736
C	11.33305679555159	9.83502819750029	1.98048406120331
C	11.24863280700674	10.30746879782967	3.36241261766008
C	8.97857202020927	6.73181952633286	3.67130797213032
C	10.58260657929813	4.56616604230550	1.74415059869938
C	11.63866481149912	10.11080508151537	-0.37071782052861
H	11.81804980562178	10.73873964137395	-1.23425090186179
C	11.57313715191602	10.66394212601283	0.89957776650379
H	11.70567883934071	11.72702061052415	1.04563897852576
C	11.41536285778130	3.57776741948545	2.27806812499396
C	11.48692469667375	8.74701740645933	-0.51463752698026
H	11.54888427869870	8.28049238802841	-1.48799420078518
C	8.16510430676971	7.83403958622611	3.37749929927367
H	8.59947815470759	8.81273145747179	3.23490359922198
C	8.52835962391216	10.95948575426829	4.78207690536434
H	8.37302032037199	12.03927285554171	4.85736014349630
H	7.55486676036800	10.48697048729479	4.93310277364010
C	6.19714357995518	6.44634366783295	3.36408607216967
C	12.84619065443740	3.81949781987849	2.63589172644276
H	13.09261248717033	3.39088300386158	3.61082901352343
H	13.12346341126501	4.87202313228700	2.63580721382700
C	9.30118868985530	4.25200684919643	1.25381212561508
C	11.23805787347160	5.81902013067453	-0.72758085725943
H	12.21288802680468	6.04562615432720	-1.17123020625000
H	10.47483380588991	6.15839661460216	-1.43087877329607
H	11.16049924210931	4.74033678836316	-0.62062909955964
C	10.69768700002840	9.82614211369244	5.58396006103129
C	9.50027204523354	10.51312002931309	5.83381500540545

C	10.24330555643916	6.67211442544263	5.14903241094311
C	6.98692312850375	5.34535750977178	3.65829022521133
H	6.53727536372663	4.36538576764335	3.77501378700204
C	8.35634329595364	5.48760923327010	3.81500580625687
H	8.94525921125610	4.61750058784459	4.08240690481613
C	6.79583640350203	7.69116268864675	3.21895591588874
H	6.19406721115764	8.55811162236452	2.96621856900040
C	8.40163243998335	5.25046406464334	0.59713788562727
H	8.59515968848192	5.29102389821379	-0.47896577036430
H	8.50804597964470	6.25499283144852	1.00683284753575
C	9.65164775884335	1.96102099865511	1.94063891860521
H	9.28989007986819	0.94220654493823	2.01486695415827
C	10.92111680199661	2.28088798777637	2.37966339144169
H	11.57114016355382	1.50901018647477	2.77647572091400
C	11.21750317006643	5.65661446897859	5.18272498604095
H	11.71118617188288	5.27304894124319	4.28019936022157
C	9.60315322400447	7.03236324315516	6.34374887413641
H	8.83154968629571	7.79124629594138	6.33158624133757
H	5.12528967646167	6.33663270143297	3.24544452747317
C	9.18082759831642	10.82085925299246	7.15496051017170
H	8.24777659812280	11.33799512121895	7.35354508934684
C	8.86045974880196	2.94005714736366	1.36809029927248
H	7.87579654362735	2.69100806867835	0.98882968756553
C	9.96236073763872	6.41935307164621	7.51952039387688
H	9.48232206217515	6.72433739215108	8.44252092841116
C	11.45990446490520	11.75094953872321	3.63876843138537
H	11.46495270948814	11.96103124553275	4.70477985736380
H	10.68674018788795	12.35850448964365	3.16146298586630
H	12.41974525137396	12.05637679981691	3.21355368021405
H	8.84041013856378	10.74832805830986	3.76301047137064
H	13.48719235921356	3.31830481876066	1.90256667429605
C	10.93072763259005	5.40615702593522	7.54368886518768
C	11.54838365336908	5.01544704245898	6.37613884566772
H	12.28276556746106	4.21882941075047	6.37235775717740
H	7.36152021026704	4.94704020155674	0.71449032681707
C	11.58122971986164	9.51675313871231	6.62915885300207
C	10.02638132815595	10.49796311695155	8.19650040431439
H	9.75968370843743	10.75169771394362	9.21604654502534
C	11.22758702284208	9.86625549525016	7.92433074178371
H	11.91285785710134	9.62936559549514	8.73070052898321
C	12.88283827515849	8.83283603688581	6.38609104189470
H	13.47806570794768	9.33670930402987	5.62287452693231
H	13.46772435507199	8.79036676224766	7.30552284379847
H	11.18417577897459	4.92270272669440	8.47947731938771
H	12.74001925097759	7.80633563192375	6.03794430701445
I	13.94492533258812	7.48026421013310	3.12797980829103
I	13.77354391514401	0.89593140266130	-0.20560577164923
I	11.45207883352172	1.96500677475112	-1.57102921837635
I	9.09172893950897	3.12516281231331	-2.87664449512604

11. References

[1] S. Stoll, A. Schweiger, *J. Magn. Reson.* **2006**, *178*, 42-55.

[2] Fritsche, P.; Geyer, L.; Czernetzki, C.; Hierlmeier, G. Coordination-induced reductive elimination from a titanium(IV) complex. *Chem. Commun.* **2024**, *60*, 9030–9033.

[3] Guérin, F.; McConville, D. H.; Payne, N. C. Conformationally Rigid Diamide Complexes: Synthesis and Structure of Titanium(IV) Alkyl Derivatives. *Organometallics* **1996**, *15*, 5085–5089.

[4] Rahimi, N.; Bruin, B. de; Budzelaar, P. H. M. [Duplikat] Balance between Metal and Ligand Reduction in Diiminepyridine Complexes of Ti. *Organometallics* **2017**, *36*, 3189–3198.

[5] DeMott, J. C.; Bhuvanesh, N.; Ozerov, O. V. Frustrated Lewis pair-like splitting of aromatic C–H bonds and abstraction of halogen atoms by a cationic [(F PNP)Pt] + species. *Chem. Sci.* **2013**, *4*, 642–649.

[6] Primpke, S.; Deinert, A.; Ehlers, F.; Vana, P. A Kinetic Investigation of the Initialization of Catalyzed Chain Growth of Styrene: The Reaction of Cp* 2 ZrCl 2 with Dibenzylmagnesium. *Macromol. Chem. Phys.* **2014**, *215*, 544–554.

[7] Bissinger, P.; Braunschweig, H.; Damme, A.; Kupfer, T.; Krummenacher, I.; Vargas, A. Boron radical cations from the facile oxidation of electron-rich diborenes. *Angew. Chem. Int. Ed.* **2014**, *53*, 5689–5693.

[8] Ivonne Chávez; Angel Alvarez-Carena; Elies Molins*; Anna Roig; Waldemar Maniukiewicz; Alenjandra Arancibia; Verónica Arancibia; Holger Brand; Juan Manuel Manríquez*. Selective oxidants for organometallic compounds containing a stabilising anion of highly reactive cations: (3,5(CF3)2C6H3)4B−)Cp2Fe+ and (3,5(CF3)2C6H3)4B−)Cp*2Fe+. *Journal of Organometallic Chemistry* **2000**, *601*, 126–132.

[9] Poynder, T. B.; Chamorro Orué, A. I.; Tania; Sharp-Bucknall, L.; Flynn, M. T.; Wilson, D. J. D.; Athukorala Arachchige, K. S.; Clegg, J. K.; Dutton, J. L. On the activation of PhICl2 with pyridine. *Chem. Commun.* **2021**, *57*, 4970–4973.

[10] Sheldrick, G. M. SADABS, Bruker AXS, Madison, USA, **2007**.

[11] CrysAlisPro, Scale3 Abspack, Rigaku Oxford Diffraction, **2019**.

[12] Sheldrick, G. M. SHELXT – Integrated space-group and crystal-structure determination. *Acta Cryst. A* **2015**, *71*, 3–8.

[13] Sheldrick, G. M. Crystal structure refinement with SHELXL. *Acta Cryst. C* **2015**, *71*, 3–8.

[14] Sheldrick, G. M. A short history of SHELX. *Acta Cryst. A* **2008**, *64*, 112–122.

[15] Dolomanov, O. V.; Bourhis, L. J.; Gildea, R. J.; Howard, J. A. K.; Puschmann, H. OLEX2: A complete structure solution, refinement and analysis program. *J. Appl. Crystallogr.* **2009**, 42, 339–341.

[16] Neese, F. The ORCA program system. WIREs Comput. Mol. Sci. **2012**, 2, 73–78.

[17] Neese, F.; Wennmohs, F.; Becker, U.; Riplinger, C. The ORCA quantum chemistry program package. *J. Chem. Phys*. **2020**, *152*, 224108.

[18] Neese, F. Software Update: The ORCA Program System—Version 6.0. *WIREs Comput. Mol. Sci.* **2025**, *15*.

[19] Caldeweyher, E.; Bannwarth, C.; Grimme, S. Extension of the D3 dispersion coefficient model. *J. Chem. Phys.* **2017**, *147*, 34112.

[20] Caldeweyher, E.; Ehlert, S.; Hansen, A.; Neugebauer, H.; Spicher, S.; Bannwarth, C.; Grimme, S. A generally applicable atomic-charge dependent London dispersion correction. *J. Chem. Phys.* **2019**, *150*, 154122.

[21] Schäfer, A.; Huber, C.; Ahlrichs, R. Fully optimized contracted Gaussian basis sets of triple zeta valence quality for atoms Li to Kr. *J. Chem. Phys.* **1994**, *100*, 5829-5835.

[22] Kossmann, S.; Neese, F. Comparison of two efficient approximate Hartee–Fock approaches. *Chem. Phys. Lett.* **2009**, *481*, 240-243.

[23] Neese, F.; Wennmohs, F.; Hansen, A.; Becker, U. Efficient, approximate and parallel Hartree–Fock and hybrid DFT calculations. A 'chain-of-spheres' algorithm for the Hartree–Fock exchange. *Chem. Phys.* **2009**, *356*, 98-109.

[24] Neese, F. An improvement of the resolution of the identity approximation for the formation of the Coulomb matrix. *J. Comput. Chem.* **2003**, *24*, 1740-1747.

[25] Ginsberg, A. P. Magnetic exchange in transition metal complexes. 12. Calculation of cluster exchange coupling constants with the X-alpha-scattered wave method. *J. Am. Chem. Soc.* **1980**, *102*, 111-117.

[26] Noodleman, L.; Peng, C. Y.; Case, D. A.; Mouesca, J. M. Orbital interactions, electron delocalization and spin coupling in iron-sulfur clusters. *Coord. Chem. Rev.* **1995**, *144*, 199-244.

[27] Kirchner, B.; Wennmohs, F.; Ye, S.; Neese, F. Theoretical bioinorganic chemistry: the electronic structure makes a difference. *Curr. Opin. Chem. Biol.* **2007**, *11*, 134-141.

[28] Neese, F. Definition of corresponding orbitals and the diradical character in broken symmetry DFT calculations on spin coupled systems. *J. Phys. Chem. Solids* **2004**, *65*, 781-785.

[29] Thom, A. J. W.; Sundstrom, E. J.; Head-Gordon, M. LOBA: a localized orbital bonding analysis to calculate oxidation states, with application to a model water oxidation catalyst. *Phys. Chem. Chem. Phys*. **2009**, *11*, 11297–11304.

[30] Lu, T.; Chen, F. Multiwfn: a multifunctional wavefunction analyzer. *J. Comput. Chem*. **2012**, *33*, 580–592.
